# Stereoretentive cross-coupling of chiral amino acid chlorides and hydrocarbons through mechanistically controlled Ni/Ir photoredox catalysis

**DOI:** 10.1038/s41467-022-32851-7

**Published:** 2022-09-03

**Authors:** Geun Seok Lee, Beomsoon Park, Soon Hyeok Hong

**Affiliations:** Department of Chemistry, Korea Advanced Institute of Science and Technology (KAIST), Daejeon, 34141 Republic of Korea

**Keywords:** Synthetic chemistry methodology, Asymmetric synthesis, Photocatalysis

## Abstract

The direct modification of naturally occurring chiral amino acids to their amino ketone analogs is a significant synthetic challenge. Here, an efficient and robust cross-coupling reaction between chiral amino acid chlorides and unactivated C(sp^3^)–H hydrocarbons is achieved by a mechanistically designed Ni/Ir photoredox catalysis. This reaction, which proceeds under mild conditions, enables modular access to a wide variety of chiral amino ketones that retain the stereochemistry of the starting amino acids. In-depth mechanistic analysis reveals that the strategic generation of an N-acyllutidinium intermediate is critical for the success of this reaction. The barrierless reduction of the N-acyllutidinium intermediate facilitates the delivery of chiral amino ketones with retention of stereochemistry. This pathway avoids the formation of a detrimental nickel intermediate, which could be responsible for undesirable decarbonylation and transmetalation reactions that limit the utility of previously reported methods.

## Abstract (web summary)

Chiral α-amino ketones are privileged motifs in bioorganic and medicinal chemistry. Here, the authors develop an efficient method to synthesize these structures via stereoretentive direct cross-coupling of amino acid chlorides with simple aliphatic substrates.

## Introduction

Chiral α-amino acids and their derivatives are among the most widely utilized compounds in organic and medicinal chemistry^1^. In particular, α-amino ketones are privileged structures^2,3^ that often serve as key intermediates in synthetic transformations and as core moieties in a wide array of drugs and biologically active molecules^4,5^. However, the streamlined synthesis of chiral α-amino ketones directly from naturally optically active α-amino acids remains challenging, compared to the synthesis of other amino acid derivatives such as α-amino aldehydes^6^. This difficulty to be overcome is their high tendency to racemize even under mild reaction conditions.

**Fig. 1:**
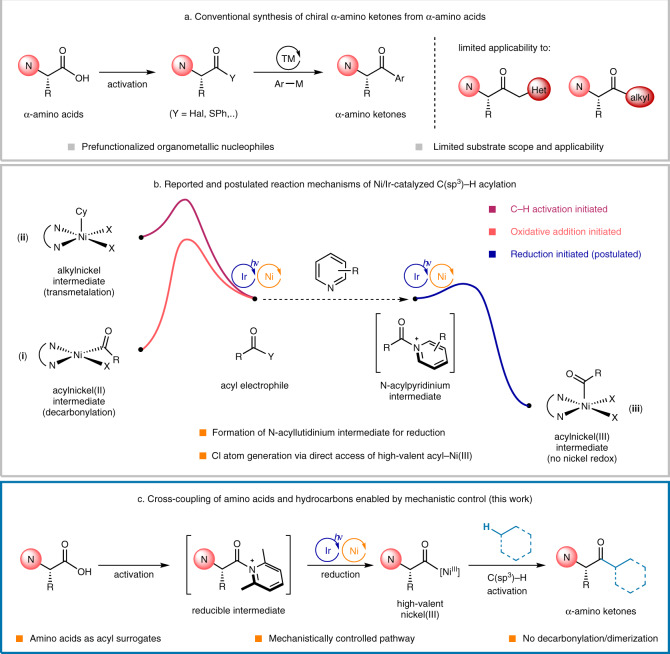
Challenges and strategy for the cross-coupling of chiral amino acids and hydrocarbons. **a** Conventional synthesis of chiral α-amino ketones from α-amino acids. **b** Reported and postulated reaction mechanisms of Ni/Ir-catalyzed C(sp^3^)–H acylation. Cy = cyclohexyl as a representative alkyl group of a C(sp^3^)–H substrate. **c** Cross-coupling of amino acids and hydrocarbons enabled by mechanistic control.

Over the last few decades, transition-metal-catalyzed cross-coupling reactions of activated chiral α-amino acid derivatives with organometallic reagents, such as organozinc^7,8^ or organoboron^9^ species, have been developed, as represented by the Liebeskind–Srogl reaction employing thioesters (Fig. 1a)^10–12^. Although efficient, these protocols have significant limitations in terms of atom economy and applicability because of the necessity of using organometallic nucleophiles. The scope of organometallic coupling partners has mainly been restricted to aromatic compounds, making structural diversification challenging. This drawback restricts the applicability of chiral α-amino ketones. For example, α,α′-diheteroatom-substituted amino ketones^13^, which could have promising biological activity^2,3^, are not readily available because of the limited synthetic accessibility of α-heteroatom-substituted organometallic reagents^14,15^. Recently, the Huo group reported the asymmetric acylation of α-amino C(sp^3^)–H bonds with carboxylic acids to afford chiral α-amino ketone products with high enantioselectivities (85–96% enantiomeric excess (ee)) via Ni/Ir photoredox catalysis using a chiral bis(oxazoline) ligand^16^.

The coupling of naturally occurring chiral α-amino acids and simple C(sp^3^)–H bonds under mild reaction conditions is an ideal synthetic method for accessing structurally diverse chiral α-amino ketones, offering enriched synthetic applicability in a practical and sustainable manner. Recently, Ni/photoredox dual catalysis^17–20^ has enabled the direct C–H acylation^21–23^ of simple hydrocarbons with acyl electrophiles for the atom-economical synthesis of ketones^24,25^ by generating a halogen atom for C–H abstraction^26–29^. Thorough mechanistic investigations revealed that two different reaction pathways could operate depending on the redox nature of the Ni complex and acyl electrophile. These pathways differ in terms of the order in which oxidative addition of the acyl electrophile and C–H activation of the hydrocarbon occur, resulting in an oxidative-addition-initiated pathway involving an acylnickel(II) intermediate (**i**)^16,30–32^ and a C–H-activation-initiated pathway involving an alkylnickel intermediate (**ii**)^33^ (Fig. 1b). However, such nickel intermediates can participate in undesirable decarbonylation^11,34–37^ and/or transmetalation^38–40^ reactions, leading to losses in optical purity and decreased reaction efficiencies (Table 2). Therefore, neither of these pathways effectively produces chiral α-amino acid derivatives. It has been established that the presence of an α-heteroatom substituent accelerates the decarbonylation of acyl radical intermediates^41^, which imposes a challenging mechanistic hurdle for the use of optically active α-amino acid derivatives in the streamlined synthesis of chiral α-amino ketones directly from hydrocarbons.

To address this challenge, a completely different reaction pathway was devised, in which the formation of both problematic species, acylnickel(II) and alkylnickel, could be bypassed. It was postulated that reducing the acyl electrophile prior to the redox reaction of the nickel species would furnish an acylnickel(III) intermediate directly (**iii**). The acylnickel(III) intermediate could then react further to form the product through photocatalytic C–H abstraction (Fig. 1b, blue trace). Beneficially, this pathway does not require modulation of the nickel oxidation state after acyl radical formation. Thus, the C–H activation and reductive elimination processes are accelerated, which kinetically inhibits the undesirable decarbonylation reaction. However, the reduction barriers of acid chlorides (e.g., benzoyl chloride **2z**, *E*^o^ = −1.53 V vs SCE) are too high for these compounds to be reduced by Ir[dF(CF_3_)ppy]_2_(dtbbpy)PF_6_ (*E*^o^_red_ = −1.37 V vs SCE), the optimized photocatalyst for reported C–H acylation reactions^42,43^. To solve the problem, more electron-deficient N-acylpyridinium compounds^44,45^, which can be formed by reacting acid chlorides with pyridine derivatives, were applied^46^.

Herein, using the designed nickel redox modulation strategy with in situ generated N-acylpyridinium intermediates, we achieved the direct cross-coupling of chiral amino acid chlorides with unactivated C(sp^3^)–H hydrocarbons to produce chiral amino ketones (Fig. 1c).

## Results

### Reaction optimization

*N*-Phthaloyl-L-phenylalanine (**1a**) and cyclohexane were chosen as model substrates to investigate the reaction conditions (Table 1). Compound **1a** was treated with an in situ generated Vilsmeier reagent^47,48^ to furnish amino acid chloride **2a**. After the simple evaporation of volatiles, **2a** was used for the reaction without further purification. The reaction conditions were optimized to obtain target product **3a** in 81% NMR yield (74% isolated yield) using 3 equiv of cyclohexane and 2 equiv of 2,6-lutidine (entry 1). Notably, no loss of stereochemical integrity occurred as the product was obtained in 99% ee (identical to that of **1a**, Supplementary Table 1). A good yield was also obtained with only 1 equiv of cyclohexane (entry 2) and the yield became nearly quantitative with 5 equiv of cyclohexane (entry 3), indicating that the proportion of a C–H substrate can be flexibly tuned depending on its availability and cost. Reducing the amount of 2,6-lutidine led to a slight decrease in the reaction efficiency (entry 4). To assess the reactivities of different pyridine derivatives, structurally diverse pyridines were tested. With pyridine itself, the reaction efficiency greatly decreased, likely due to undesired radical addition to pyridine (entry 5)^49^. When 2,4,6-collidine was introduced instead of 2,6-lutidine, a similar yet slightly diminished reactivity was observed (entry 6). The use of 2,6-di-*tert*-butylpyridine led to a huge loss of reaction efficiency, demonstrating that a sterically more hindered pyridine derivative lowers the reactivity (entry 7)^50^. 2,4,6-Triphenylpyridine, which may form an acyl analog of the Katritzky salt^51,52^, generated the targeted product in a moderate yield (entry 8). When 2,6-lutidine was replaced with common inorganic bases such as cesium carbonate, sodium bicarbonate, or potassium phosphate, the reaction efficiency decreased significantly (entries 9–11). In addition, under base-free conditions, only a low yield of the desired product was observed (entry 12), indicating the essential role of 2,6-lutidine. Control experiments without light, the nickel catalyst, or the photocatalyst failed to produce the desired product, implying that these components are necessary for the reaction to proceed (entry 13).

**Table 1:** Optimization of reaction conditions^a^


Entry	Variation from standard conditions	Yield^b^ (%)
1	None	81 (74^c^)
2	1 equiv of cyclohexane	58
3	5 equiv of cyclohexane	90
4	1 equiv of 2,6-lutidine	64
5	pyridine instead of 2,6-lutidine	36
6	2,4,6-collidine instead of 2,6-lutidine	76
7	2,6-di-*tert*-butylpyridine instead of 2,6-lutidine	27
8	2,4,6-triphenylpyridine instead of 2,6-lutidine	53
9	Cs_2_CO_3_ instead of 2,6-lutidine	19
10	NaHCO_3_ instead of 2,6-lutidine	17
11	K_3_PO_4_ instead of 2,6-lutidine	20
12	No base	13
13	No light/No nickel and dtbbpy/No photocatalyst	0

^a^Reaction conditions: **1a** (0.20 mmol), oxalyl chloride (1.8 equiv), DMF (cat., 0.2 μL), and CH_2_Cl_2_ (2.0 mL), 2 h. After evaporation of remaining solvent, NiCl_2_·glyme (5 mol %), dtbbpy (10 mol %), 2,6-lutidine (2.0 equiv), cyclohexane (3.0 equiv), Ir[dF(CF_3_)ppy]_2_(dtbbpy)PF_6_ (1 mol %), and benzene (8.0 mL), irradiated 12 h with a Penn PhD M2 photoreactor.

^b^Yields were determined by NMR spectroscopy using 1,1,2,2-tetrachloroethane as an internal standard.

^c^Isolated yield, 99% ee. NPhth = phthalimidyl.

### Substrate scope

**Fig. 2:**
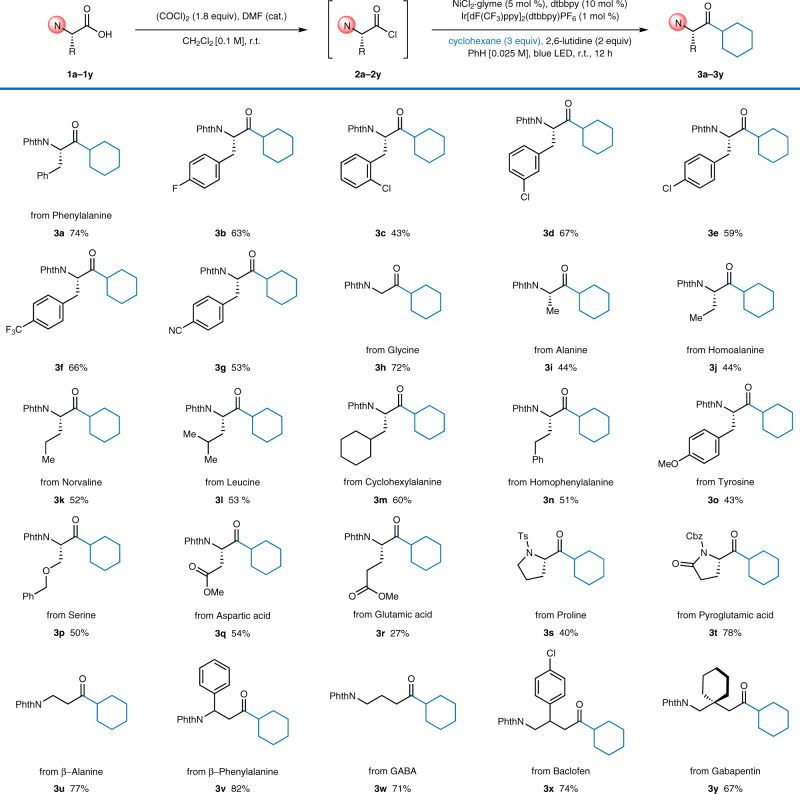
Amino acid scope. Reaction conditions: **1a–1y** (0.20 mmol), oxalyl chloride (1.8 equiv), DMF (cat., 0.2 μL), and CH_2_Cl_2_ (2.0 mL). After evaporation of the remaining solvent, NiCl_2_·glyme (5 mol %), dtbbpy (10 mol %), 2,6-lutidine (2.0 equiv), cyclohexane (3.0 equiv), Ir[dF(CF_3_)ppy]_2_(dtbbpy)PF_6_ (1 mol %), and benzene (8.0 mL) were added, and the solution was irradiated for 12 h with a Penn PhD M2 photoreactor. All yields are isolated yields.

With the optimized conditions in hand, the amino acid scope of the developed method was investigated (Fig. 2). The reaction was effective with a wide range of amino acids, demonstrating excellent functional group compatibility. Initially, several phenylalanine derivatives were tested (**3a**–**3g**). To our delight, even fluorine- or chlorine-substituted derivatives (**3b**–**3e**) were well tolerated, providing opportunities for further functionalization. Electron-withdrawing trifluoromethyl and cyano groups on the phenyl ring of the phenylalanine did not affect the reaction efficiency (**3f**, **3g**). Other amino acids also successfully delivered the corresponding chiral amino ketones. The simplest amino acid, glycine, exhibited a good yield (72%, **3h**). Alkyl-chain-bearing amino acids such as alanine, homoalanine, norvaline, leucine, and cyclohexylalanine, which possess high steric hindrance and hydrophobicity^53^, reacted smoothly under the standard reaction conditions (**3i**–**3m**). Homophenylalanine, an important bioactive non-natural chiral amino acid^54^, effectively produced **3n** in moderate yield (51%). Notably, amino acids bearing polar side chains could also be employed after appropriate protection. O*-*Methylated tyrosine reacted smoothly (43%, **3o**). Furthermore, benzyl-protected serine gave the desired ketone in moderate yield (50%, **3p**). Aspartic acid and glutamic acid methyl esters furnished chiral amino ketones bearing ester side chains, albeit in lower yields (54%, **3q**; 27%, **3r**). The reactions of cyclic amino acids with different protecting groups also furnished the target products (40%, **3s**; 78%, **3t**). Due to their innate instability and high tendency to undergo racemization, peptidyl acid chlorides bearing more than two amino acid residues could not be employed^47,48^. In addition, α-amino acid homologues were tested, as they are known to exhibit significantly different biochemical properties^55^. Two β-amino acids (β-alanine and β-phenylalanine) delivered the target products in very good yields (77%, **3u**; 82%, **3v**). Moreover, γ-amino acids, such as γ-aminobutyric acid (GABA), baclofen, and gabapentin, all effectively gave the corresponding products in good yields (67–74%, **3w**–**3y**).

**Fig. 3:**
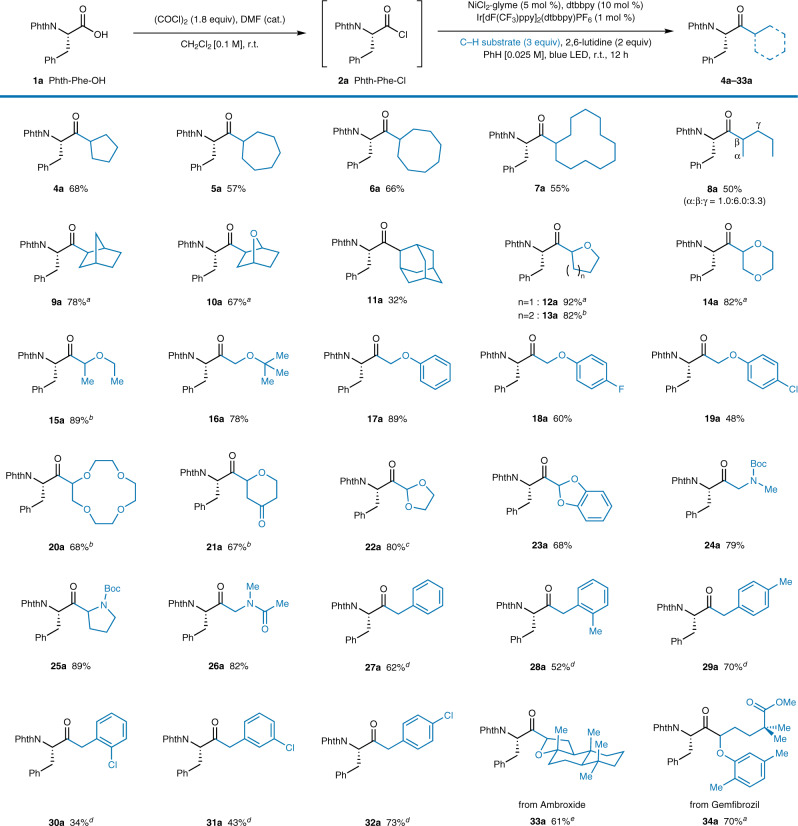
C–H substrate scope. Reaction conditions: **1a** (0.20 mmol), oxalyl chloride (1.8 equiv), DMF (cat., 0.2 μL), and CH_2_Cl_2_ (2.0 mL). After evaporation of the remaining solvent, NiCl_2_·glyme (5 mol %), dtbbpy (10 mol %), 2,6-lutidine (2.0 equiv), C–H substrate (3.0 equiv), Ir[dF(CF_3_)ppy]_2_(dtbbpy)PF_6_ (1 mol %), and benzene (8.0 mL) were added, and the solution was irradiated for 12 h with a Penn PhD M2 photoreactor. All yields are isolated yields. ^*a*^d.r. = 1:1. ^*b*^d.r. = 2:1. ^*c*^r.r. = 4:1; d.r. for the minor regioisomer = 1:1. ^*d*^10 equiv of C–H substrate. ^*e*^d.r. = 3:1.

Next, the scope of C(sp^3^)–H substrates was examined (Fig. 3). The reactions with simple cyclic alkanes, from cyclopentane to cyclododecane, proceeded smoothly (**4a**–**7a**). An acyclic alkane, pentane, also smoothly produce the desired aminoketone as a mixture of regioisomers (**8a**, α:β:γ = 1.0:6.0:3.3, 5% terminal selectivity after statistical correction). Bicyclic compounds such as norbornane and 7-oxanorbornane also efficiently furnished the target products (78%, **9a**; 67%, **10a**). Adamantane was acylated exclusively at the secondary position (32%, **11a**), consistent with a previous report^33^. Cyclic ethers including tetrahydrofuran, tetrahydropyran, and 1,4-dioxane gave very good to excellent yields (**12a**–**14a**), showing exclusive selectivity for the more reactive ethereal C–H bonds. Acyclic ethereal substrates such as diethyl ether and methyl *tert*-butyl ether also reacted smoothly (89%, **15a**; 78%, **16a**). Various anisole derivatives reacted well under the optimized conditions (**17a**–**19a**), showing tolerance for halogen functionalities on the aromatic ring. To our delight, an amino acid fragment was directly introduced into the well-known ionophore 12-crown-4 in a synthetically applicable yield (68%, **20a**), thus realizing one-pot production of a crown ether with a chiral α-amino ketone moiety. When pyranone was used as the coupling partner, the 1,4-dicarbonyl **21a** was obtained in 67% yield. Acetals were also successfully functionalized to provide α-ketoacetal products (80%, **22a**; 68%, **23a**)^56,57^.

The direct cross-coupling of amino acids and amino alkyl substrates produced unsymmetrical α,α′-diaminoketones in very good yields (79–89%, **24a**–**26a**). Although some 1,3-diaminoketone compounds have been reported to exhibit bioactive properties^2^, synthetic access to this substrate class has been limited^13^. To the best of our knowledge, the developed reaction is unique in providing direct synthetic access to α′-oxy- or α′-amino-substituted chiral amino ketones. Because α-heteroatom-substituted organometallic reagents cannot be readily prepared, transition-metal-catalyzed cross-coupling reactions (Fig. 1a) are not readily applicable for synthesizing such compounds^14,15^. Toluene, *o-*xylene, and *p*-xylene delivered the desired products in moderate yields (52–70%, **27a**–**29a**); however, benzylic functionalization required more equivalents (10 equiv) of the C–H substrate. Aryl chlorides were also tolerated as the C–H substrate, as in the case of amino acids (**30a**–**32a**). Complex bioactive C–H substrates were also tolerated producing the corresponding amino-acid-coupled complexes. Ambroxide (61%, **33a**) and gemfibrozil (70%, **34a**) reacted smoothly to provide the coupled products in a regioselective fashion, which demonstrates the streamlined late-stage introduction of a chiral amino acid moiety, taking advantage of the excellent functional group compatibility of the developed protocol. Having observed that our reaction disfavors tertiary C–H bonds when adamantane was used, we attempted an intermolecular competition experiment using an equivalent amount of cyclohexane and 2,3-dimethylbutane (Supplementary Fig. 40). The reaction exclusively furnished the secondary C–H functionalized product **3a**, albeit in a lowered yield (63%, 99% ee). We presume this unusual secondary selectivity arises from steric hindrance lowering the reactivity at the tertiary position. Finally, the stereochemical integrity of selected products (**3a**, **3k**, **3l**, **3n**, **3o**, **3s**, **16a**, **17a**, **26a**, and **27a** as chosen based on the limited availability of racemic compounds) was investigated. The stereochemistry was fully maintained during the reaction, except with benzylic substrates, which led to a small decrease in enantiopurity (from 99% to 98% ee, Supplementary Table 1).

### Mechanistic investigations

After demonstrating the wide applicability of the developed reaction conditions, the underlying reaction mechanism was investigated through comprehensive computational and experimental studies. First, control experiments were performed to compare the developed reaction conditions with the two previously reported protocols, which featured oxidative addition first^16,30–32^ or C–H activation first^33^ (Table 2). The optimized reaction conditions (entry 1) afforded the desired product in high yields without any enantiomeric loss, whereas the previously reported reaction conditions exhibited low yields accompanied by a significant decrease in enantioselectivity (entries 2^31^ and 3^33^). Even when N-acylsuccinimide **35a** was employed under the reported conditions^33^ for the C–H-activation-initiated pathway, no product was generated (entry 4). It has been reported that such acyclic secondary-alkyloyl-derived N-acylsuccinimide substrates are incompatible, presumably due to their sensitivity to sterics^33^. Notably, a dimerization byproduct (**2a-dimer**) was detected in both entries 2 and 3, but no such product was generated under the developed reaction conditions, implying that undesirable decarbonylation or transmetalation processes were successfully suppressed by the proposed strategy.

**Table 2:** Control experiments under reported conditions for Ni/Ir-catalyzed C(sp^3^)–H acylation^a^


Entry	Substrate	Conditions	Yield (3a)	Yield (2a-dimer)
1	**2a**	Standard conditions	74% (99% ee)	0%
2	**2a**	Ni(COD)_2_ (4 mol %), dtbbpy (5.2 mol %), [Ir(dFCF_3_ppy)_2_(dtbbpy)]PF_6_ (0.5 mol %) K_3_PO_4_ (2 equiv), Na_2_WO_4_• 2H_2_O (1 equiv), PhH [0.1 M], r.t., 48 h, blue LED	25% (93% ee)	26% (1:1 d.r.)
3	**2a**	NiCl_2_ • glyme (5 mol %), dtbbpy (10 mol %), [Ir(dFCF_3_ppy)_2_(dtbbpy)]PF_6_ (1 mol %) K_2_CO_3_ (2 equiv), PhH [0.1 M], r.t., 16 h, blue LED	28% (91% ee)	10% (1:1 d.r.)
4	**35a**	NiCl_2_ • glyme (5 mol %), dtbbpy (10 mol %), [Ir(dFCF_3_ppy)_2_(dtbbpy)]PF_6_ (1 mol %) K_2_CO_3_ (2 equiv), PhH [0.1 M], r.t., 16 h, blue LED	N.D.	N.D.

^a^All yields are isolated yields. Diastereomeric ratios were determined by ^1^H NMR spectroscopy. N.D. = not detected.

It is well documented that N-acylpyridiniums can be generated from acyl chlorides and pyridines^58^. Some stabilized N-acylpyridiniums, such as N-acylpyridinium and *N*,*N*-dimethylaminopyridinium salts, have been isolated and fully characterized^44,59^. However, attempts to isolate the N-acyllutidinium intermediate **2z-lut** were unsuccessful, presumably because its stability was decreased by the steric hindrance of the pyridine ring.

**Fig. 4:**
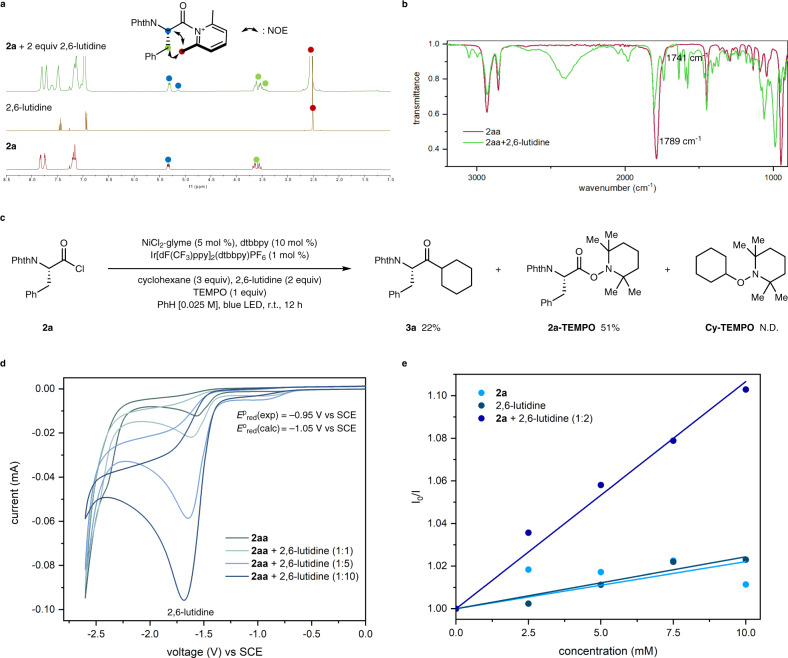
Mechanistic studies. **a** NMR studies. **b** IR studies. **c** Radical scavenger experiment. Yields were determined using ^1^H NMR spectroscopy with 1,1,2,2-tetrachloroethane as an internal standard. **d** Cyclic voltammetry studies. **e** Stern–Volmer quenching experiment.

Instead, in situ NMR studies were conducted to investigate the formation of **2a-lut**. The ^1^H NMR spectrum of a 1:2 mixture of **2a** and 2,6-lutidine is shown in Fig. 4a. Here, an upfield shift of the **2a** resonances and a downfield shift of the 2,6-lutidine methyl peak was witnessed. This observation is in good agreement with the analogous lutidinium salt generated from ethyl chlorooxoacetate and 2,6-lutidine, reported by the Wu group^46^. In addition, pronounced peak broadening and even separation of the α-carbonyl proton resonance were detected, likely due to the steric bulkiness of the 2,6-lutidine moiety leading to the formation of rotameric species. An NOE study of this mixture indicated NOE signals between the carbonyl α-proton, benzylic proton and the lutidine methyl group, indicating their presence in the same molecule. Further studies were conducted by monitoring the IR carbonyl stretch of cyclohexane carbonyl chloride (**2aa**) with and without the addition of 2,6-lutidine (Fig. 4b). Here, **2aa** was chosen as the phthalimide group present in **2a** led to complex carbonyl absorptions. The characteristic carbonyl stretch of **2aa** was observed at 1789 cm^−1^. When 1 equiv of 2,6-lutidine was introduced, the IR spectrum clearly showed a new absorption band in the carbonyl region at 1741 cm^−1^. This is indicative of the formation of a new carbonyl species, like the postulated N-acyllutidinium intermediate.

After confirming the generation of an N-acyllutidinium compound, the single-electron reduction of the N-acyllutidinium intermediate was investigated. When 2,2,6,6-tetramethylpiperidine-*N*-oxide (TEMPO, 1 equiv) was introduced, acyl-TEMPO (**2a-TEMPO**) was formed in 51% yield, indirectly confirming the generation of acyl radicals during the reaction (Fig. 4c). Next, cyclic voltammetry experiments were conducted with **2aa** as the model substrate (Fig. 4d). The measured reduction potential of **2aa** was –2.45 V vs SCE, which indicates that the direct reduction of **2aa** is not feasible with the iridium photocatalyst (Ir[dF(CF_3_)ppy]_2_(dtbbpy)PF_6_, *E*^o^_red_ = −1.37 V vs SCE)^60,61^. After the addition of 2,6-lutidine, the reduction wave of **2aa** was diminished, accompanied by the formation of a new irreversible reduction wave at *E*^p^ = −0.95 V vs SCE. This new reduction likely originated from the lutidinium intermediate **2aa-lut**. The observed value, which falls within the range suitable for reduction by Ir[dF(CF_3_)ppy]_2_(dtbbpy)PF_6_, is in good agreement with the computed reduction potential of **2aa-lut** (−1.05 V vs SCE). This change in the reduction potential suggests that the generation of acyl radicals is facilitated by the formation of an N-acyllutidinium intermediate, as initially postulated. Furthermore, the reduction potential of the lutidinium species when coordinated to nickel catalyst (^**3**^**IV-2aa**) was computed to be less negative (–0.85 V vs SCE), indicating that the nickel catalyst may facilitate the reduction process. In addition, Stern–Volmer quenching experiments were conducted with **2a** and 2,6-lutidine. **2a** or 2,6-lutidine alone could not effectively quench the iridium photocatalyst. However, a 1:2 mixture of **2a** and 2,6-lutidine showed highly efficient quenching of the excited photocatalyst (Fig. 4e). Overall, these mechanistic studies confirm that the proposed N-acyllutidinium intermediate is indeed generated and can be effectively reduced to furnish the postulated acylnickel species.

**Fig. 5:**
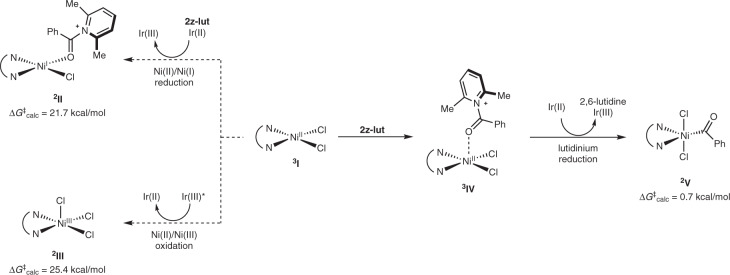
Computed redox properties of Ni(II) and N-acyllutidinium species. The reduction of Ni-coordinated N-acyllutidinium species ^**3**^**IV** was computed to be practically barrierless (0.7 kcal/mol), outcompeting other redox pathways involving the nickel precatatlyst ^**3**^**I**.

Furthermore, to obtain an improved understanding of the reaction mechanism, computational studies were performed using density functional theory (DFT) at the B3LYP-D3/6-311++G**/SDD^62,63^ level of theory (Supplementary Data 1). First, the redox barriers (Δ*G*^‡^) of initial catalytic species (dtbbpy)Ni^II^Cl_2_^**3**^**I** were investigated using Marcus theory^64,65^ to clarify its behavior (Fig. 5). The barrier for reduction of ^**3**^**I** to Ni(I) species ^**2**^**II**, a key process in the oxidative-addition-initiated pathway, was computed to be 21.7 kcal/mol. Similarly, the barrier for the oxidation of ^**3**^**I** to high-valent Ni(III) species ^**2**^**III** in the C–H-activation-initiated pathway was determined to be 25.4 kcal/mol. In contrast, the proposed single-electron reduction of **2z-lut** after binding to nickel species ^**3**^**IV** was found to be practically barrierless, directly delivering acylnickel(III) intermediate ^**2**^**V**. These results clearly indicate that the reduction of **2z-lut** is favored over the redox processes of the relevant nickel species, and this serves as a thermodynamic sink to drive the reaction.

**Fig. 6:**
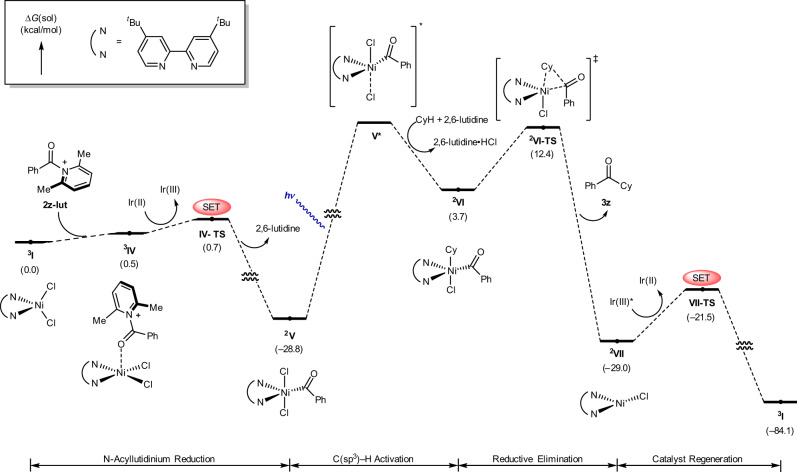
Computed free energy profile for the overall reaction with substrate 2z. The computed reaction profile demonstrates facile reduction of ^**3**^**IV** followed by photolytic C–H activation and reductive elimination to deliver amino ketone product **3z**. Energies are given in kcal/mol values.

A full energy profile for the proposed pathway was constructed through extensive computational studies (Fig. 6). Initially, the N-acyllutidinium intermediate undergoes facile coordination to nickel precatalyst ^**3**^**I** to give ^**3**^**IV**. Initially, the N-acyllutidinium intermediate undergoes facile coordination to nickel precatalyst ^**3**^**I** to give ^**3**^**IV**, followed by an essentially barrierless reduction (0.7 kcal/mol) to form ^**2**^**V** (–28.8 kcal/mol), which serves as a thermodynamic sink, rendering this process irreversible. The photolysis of ^**2**^**V** may lead to its excited state V*, which undergoes chlorine-mediated hydrogen atom transfer to give alkylnickel species ^**2**^**VI** (3.7 kcal/mol). Excited state V* and its C–H abstracting transition state could not be located in a straightforward manner using DFT. However, it is well documented that such processes are downstream transformations when assisted by light as an energy source^66,67^. This process has served as a fundamental step for the development of a variety of C(sp^3^)–H functionalization reactions through the implementation of photoirradiated Ni/Ir dual catalysis^68^. Kinetic isotope effect (KIE) experiments using **2a** as a substrate resulted in *k*_H_/*k*_D_ values of 1.08 (parallel reactions) and P_H_/P_D_ values of 1.83 (intermolecular competition reactions)^69^ (Supplementary Fig. 55–57). This indicates that the product-determining C–H activation (^**2**^**V** to ^**2**^**VI**) is not the turnover-limiting step^33^. Reductive elimination through ^**2**^**VI-TS** to produce the desired product was found to have an activation barrier of 8.7 kcal/mol^70^. Finally, the oxidation of Ni(I) species ^**2**^**VII** to initial precatalyst ^**3**^**I** through **VII-TS** (–21.5 kcal/mol) completes the catalytic cycle. In this case, the overall reaction barrier (8.7 kcal/mol) is very low, which accounts for the kinetic inhibition of side reactions such as decarbonylation. These results indicate that modulation of the redox state of the nickel species is rather sluggish. Thus, the designed strategy, which bypasses the redox processes of the nickel species, is crucial for direct C(sp^3^)–H coupling between chiral α-amino acid chlorides and hydrocarbons.

**Fig. 7:**
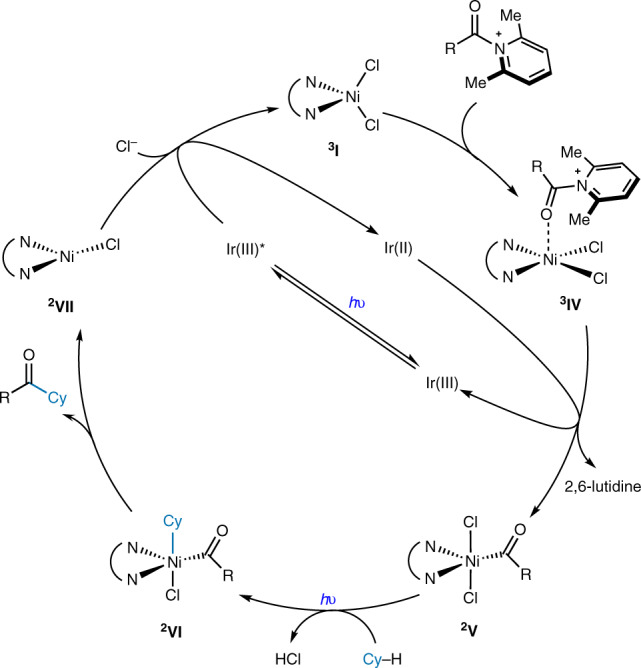
Proposed reaction mechanism. The iridium photocatalyst serves to reduce the N-acyllutidnium bound to the Ni(II) species (^**3**^**IV**), furnishing Ni(III) species ^**2**^**V**. ^**2**^**V** undergoes light-assisted C–H activation and reduction elimination to deliver the ketone product and Ni(I) species ^**2**^**VII**. Finally, ^**2**^**VII** is oxidized back the the initial catalyst ^**3**^**I**.

Combining the experimental and computational findings, a plausible mechanism is proposed, as shown in Fig. 7. The N-acyllutidinium intermediate binds to the nickel(II) precatalyst, generating ^**3**^**IV**. Subsequent single electron reduction of ^**3**^**IV** furnishes acyl group-bound ^**2**^**V** along with the liberation of 2,6-lutidine. This high-valent nickel species undergoes hydrogen atom transfer mediated by a chlorine radical liberated through direct photolysis to yield alkyl acyl nickel species ^**2**^**VI**. This species then undergoes reductive elimination to deliver the desired ketone product, followed by reoxidation of the resulting Ni(I) species ^**2**^**VII** to its initial state ^**3**^**I**.

In conclusion, cross-coupling between chiral amino acid chlorides and unactivated C(sp^3^)–H substrates was realized using nickel/photoredox dual catalysis under mild reaction conditions. Through strategic modulation of the reaction mechanism, a variety of chiral amino acids were transformed into the corresponding amino ketones without the loss of stereochemical integrity. This method overcomes the limitations associated with decarbonylative racemization in previously reported methodologies for Ni/photoredox catalysis. Comprehensive mechanistic studies revealed that the N-acyllutidinium intermediate, generated from the acid chloride and 2,6-lutidine, is crucial for driving the reaction to the successful reduction-initiated pathway. This pathway commences with the single-electron reduction of the N-acyllutidinium species, thus directly furnishing an acylnickel(III) intermediate and preventing undesirable side reactions. Computational analysis revealed that modulating the nickel oxidation state is a sluggish process that may lead to acyl radical decarbonylation. Thus, circumventing this process with the developed reduction-initiated strategy is key for maintaining the optical purity of the product. Various functionalized chiral amino ketones were efficiently synthesized using the developed reaction. The present findings demonstrate successful mechanistic control to realize a challenging coupling reaction in Ni/photoredox catalysis, which can provide further insight into the development of new synthetic methods.

## Methods

### General procedure for the N-acyllutidinium-mediated acylation

To an 8 mL vial equipped with a PTFE-coated stirrer bar were added the corresponding N-protected amino acid (0.20 mmol, 1.0 equiv), oxalyl chloride (30.9 μL, 0.36 mmol, 1.8 equiv), a catalytic amount of DMF (0.2 μL), and CH_2_Cl_2_ (2.0 mL). The resulting mixture was stirred for 2–16 h at room temperature before it was concentrated under reduced pressure and azeotropically dried with benzene (2 mL × 2) to afford the desired N-protected amino acid chloride which was used directly for the next step.

To the same vial containing the corresponding acid chloride were added NiCl_2_·glyme (2.2 mg, 0.01 mmol, 0.05 equiv), dtbbpy (4,4′-di-*tert*-butyl-2,2′-dipyridyl) (5.37 mg, 0.02 mmol, 0.10 equiv), Ir[dF(CF_3_)ppy]_2_(dtbbpy)PF_6_ (2.2 mg, 0.002 mmol, 0.01 equiv), 2,6-lutidine (46.6 μL, 0.40 mmol, 2.0 equiv), the corresponding C–H substrate (0.60 mmol, 3.0 equiv), and benzene (8.0 mL). The resulting mixture was stirred for 12 h under blue LED irradiation in a Penn PhD M2 photoreactor (1200 stir rpm, 6800 fan rpm, 100% light intensity). The reaction mixture was then diluted with HCl (1 M aq., 5 mL), extracted with CH_2_Cl_2_ (3 × 5 mL), dried (anhydrous Na_2_SO_4_), filtered, and concentrated under reduced pressure. When N-Boc-protected amine substrates were used as the C–H substrate, the work-up procedure was omitted. The reaction mixture was filtered through a short pad of Celite®, eluted with CH_2_Cl_2_, and concentrated under reduced pressure. The resulting residue was purified by flash column chromatography (silica gel, hexanes/EtOAc or hexanes/Et_2_O gradient elution) to afford the desired aminoketone product.

## Supplementary information

Supplementary Information

Description of Additional Supplementary Files

Supplementary Data 1

## Data Availability

Detailed experimental procedures, computational details, and characterization data for new compounds are available from the Supplementary Information. Cartesian coordinates of the calculated structures are available from the Supplementary Data 1. The authors declare that the data supporting the manuscript are included in the manuscript and supplementary materials.
