# Supplementary material for: Stereoretentive cross-coupling of chiral amino acid chlorides and hydrocarbons through mechanistically controlled Ni/Ir photoredox catalysis
Source: Nat Commun. 2022 Sep 3;13:5200. doi: 10.1038/s41467-022-32851-7 (PMC9440902; doi:10.1038/s41467-022-32851-7)
Supplement: Supplementary file 1 — Supplementary Information [file 41467_2022_32851_MOESM1_ESM.pdf]

Supplementary Information for

Stereoretentive Cross-Coupling of Chiral Amino Acid Chlorides and Hydrocarbons Through Mechanistically Controlled Ni/Ir Photoredox Catalysis

Geun Seok Lee,[1‡] Beomsoon Park,[1‡] and Soon Hyeok Hong[1]*

[1]Department of Chemistry, Korea Advanced Institute of Science and Technology (KAIST), Daejeon 34141, Republic of Korea.

[‡]These authors contributed equally.

E-mail: soonhyeok.hong@kaist.ac.kr

Table of contents

1 Supplementary Methods (S2)

1.1 General Information (S2)

1.2 Substrate Preparation (S2)

1.3 General Procedure for the N-Acyllutidinium-Mediated Acylation (S3)

1.4 Characterization Data (S3)

1.5 Chiral HPLC Data (S19)

2 Supplementary Discussion (S31)

2.1 Control Experiments in Previously Reported Ni/Ir-Catalyzed Acylation Conditions (S31)

2.2 Intermolecular Competition Experiment (S35)

2.3 NMR Studies for the Equilibrium of N-Acyllutidinium Intermediate (S36)

2.4 IR Studies for the Equilibrium of N-Acyllutidinium Intermediate (S37)

2.5 Radical Scavenger Experiment (S38)

2.6 Cyclic Voltammetry Experiments (S39)

2.7 Stern-Volmer Quenching Experiments (S41)

2.8 Kinetic Isotope Effect Measurements (S43)

2.9 Computational Details (S44)

3 Supplementary Notes (S48)

3.1 ^{1}H, ^{13}C and ^{19}F NMR Spectra (S48)

4 Suppelementary References (S113)

1. Supplementary Methods

1.1. General Information

Unless otherwise noted, all reactions were performed under inert conditions. Nuclear magnetic resonance (NMR) spectra were recorded on a Bruker AVANCE 300 (300 MHz), Bruker AVANCE 400 (400 MHz), Bruker AVANCE III Nanobay (400 MHz), Bruker AVANCE NEO Nanobay (400 MHz), Bruker AVANCE III HD (400 MHz), or Bruker AVANCE NEO (500 Mhz) and the residual solvent signal was used as a reference. High-resolution mass spectrometry (HRMS) was performed at the Korea Basic Science Institute (KBSI) using the EI or FAB method. Chemical shifts are reported in ppm, and coupling constants are given in Hz. Gas chromatography (GC) was carried out using a 7890A or 7890B GC system (Agilent Technologies) equipped with an HP-5 column and a flame ionization detector (FID). High-performance liquid chromatography (HPLC) analysis was performed using a 1260 Infinity LC system (Agilent Technologies) equipped with CHIRALPAK IA-3, CHIRALPAK IB, CHIRALPAK IB-3, or CHIRALPAK OD-H column. Infrared (IR) spectra were acquired on Bruker Alpha ATR FT-IR Spectrometer, or Thermo Scientific Nicolet iS50 Spectrometer. Melting point were measured using Buchi Melting Point M-565. Reactions were monitored by thin-layer chromatography (TLC) on EMD Silica Gel 60 F254 plates and visualized either using UV light (254 nm) or by staining with potassium permanganate and heating. Tetrahydrofuran (THF), diethyl ether, benzene, and toluene were dried using a PureSolv solvent purification system. All chemicals were purchased from commercial sources (Sigma-Aldrich, Alfa Aesar, TCI) and used without further purification. The photocatalyst Ir[dF(CF_3)ppy]$_2$(dtbbpy)PF_6 was synthesized following a literature procedure.[1] All photocatalytic reactions were conducted under irradiation by Penn PhD Photoreactor M2 equipped with 420 nm Light Source (Sigma-Aldrich) using the maximum light intensity.

1.2. Substrate Preparation

N-Protected amino acids **1a–1y**[2] were prepared following the described procedure. All C−H substrates except *N*-Boc-pyrrolidine and Gemfibrozil methyl ester were purchased from commercial sources and used after degassing for liquids and directly for solids. *N*-Boc-pyrrolidine was synthesized by Boc protection of pyrrolidine following literature procedures.[3] Gemfibrozil methyl ester was synthesized by esterification of Gemfibrozil.[4]

Preparation of N-Protected Amino Acids 1a–1y

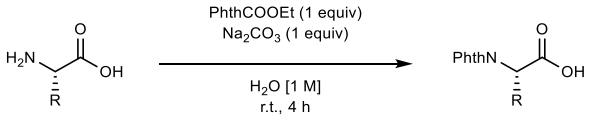

N-Protected amino acids were prepared following a slightly modified literature procedure.[5] To a flask equipped with a stirrer-bar were added the corresponding amino acid (1.0 mmol, 1.0 equiv), Na_2CO_3 (1.0 mmol, 1.0 equiv), and water (1.0 mL). The resulting mixture was stirred at room temperature for 4 h and acidified with aqueous HCl until precipitates were slowly generated. When no precipitate was generated further, the resulting mixture was extracted with EtOAc (3 mL), concentrated under reduced pressure. The resulting residue was then purified by flash column chromatography (silica gel, hexanes/EtOAc gradient elution) to afford protected amino acids **1a**–**1y**.

1.3. General Procedure for the N-Acyllutidinium-Mediated Acylation

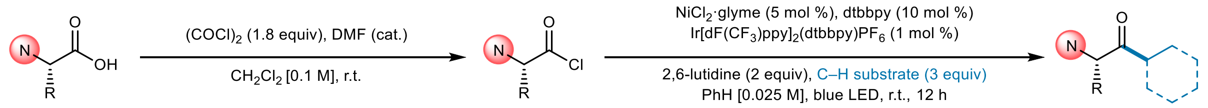

To an 8 mL vial equipped with a PTFE-coated stirrer bar were added the corresponding N-protected amino acid (0.20 mmol, 1.0 equiv), oxalyl chloride (30.9 µL, 0.36 mmol, 1.8 equiv), a catalytic amount of DMF (0.2 µL), and CH_2Cl_2 (2.0 mL). The resulting mixture was stirred for 2–16 h at room temperature before it was concentrated under reduced pressure and azeotropically dried with benzene (2 mL × 2) to afford the desired N-protected amino acid chloride which was used directly for the next step.

To the same vial containing the corresponding acid chloride were added $NiCl_2$·glyme (2.2 mg, 0.01 mmol, 0.05 equiv), dtbbpy (4,4′-di-*tert*-butyl-2,2′-dipyridyl) (5.37 mg, 0.02 mmol, 0.10 equiv), $Ir[dF(CF_3)ppy]_2(dtbbpy)PF_6$ (2.2 mg, 0.002 mmol, 0.01 equiv), 2,6-lutidine (46.6 µL, 0.40 mmol, 2.0 equiv), the corresponding C–H substrate (0.60 mmol, 3.0 equiv), and benzene (8.0 mL). The resulting mixture was stirred for 12 h under blue LED irradiation in a Penn PhD M2 photoreactor (1200 stir rpm, 6800 fan rpm, 100% light intensity). The reaction mixture was then diluted with HCl (1 M aq., 5 mL), extracted with CH_2Cl_2 (3 × 5 mL), dried (anhydrous Na_2SO_4), filtered, and concentrated under reduced pressure. When N-Boc-protected amine substrates were used as the C–H substrate, the work-up procedure was omitted. The reaction mixture was filtered through a short pad of Celite®, eluted with CH_2Cl_2, and concentrated under reduced pressure. The resulting residue was purified by flash column chromatography (silica gel, hexanes/EtOAc or hexanes/Et_2O gradient elution) to afford the desired aminoketone product.

1.4. Characterization data

Newly synthesized compounds (**1c, 1g**) were characterized by ^{1}H NMR, ^{13}C NMR and HRMS. The identity of the reported N-protected amino acids (**1a, 1b, 1d**–**1f, 1h**–**1s, 1u**–**1y**) were confirmed by spectral comparison with literature data. The commercially available **1t** was used as purchased. Newly synthesized compounds (**3a**–**3y, 4a**–**34a**) were characterized by ^{1}H NMR, ^{13}C NMR, ^{19}F NMR, and HRMS.

References to characterization data for the reported compounds:

(1a, 1d, 1f, 1o)[6]; **1b**[7]; **(1e, 1n, 1j)**[8]; **(1h, 1q)**[9]; **(1i, 1m)**[10]; **(1k, 1l)**[11]; **1p**[12]; **1s**[13]; **(1u, 1v)** [14]; **1w**[15]**; 1x**[16]**; 1y**[17].

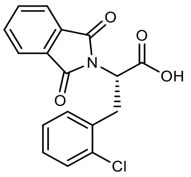

(*S*)-3-(2-Chlorophenyl)-2-(1,3-dioxoisoindolin-2-yl)propanoic acid (1c)

White solid, 141.3 mg (0.43 mmol, 43% yield); m.p. 160–162 °C; ^{1}H NMR (500 MHz, $CDCl_3$): δ = 7.81 – 7.78 (m, 2H), 7.73 – 7.66 (m, 2H), 7.31 (d, *J* = 7.9 Hz, 1H), 7.14 – 7.06 (m, 2H), 7.05 – 6.98 (m, 1H), 5.41 (dd, *J* = 11.4, 4.5 Hz, 1H), 3.83 – 3.75 (m, 1H), 3.66 – 3.57 (m, 1H); ^{13}C NMR (125 MHz, $CDCl_3$): δ = 173.8, 167.3, 134.4, 134.2, 131.5, 131.3, 129.8, 128.7, 126.9, 123.6, 51.0, 32.8; IR (cm^{-1}) 3309, 2179, 1976, 1760, 1701, 1398, 1224, 1085, 941, 723, 531; HRMS-EI (m/z) $[M]^+$ calcd for $C_{17}H_{12}ClNO_4$, 329.0455; found: 329.0456.

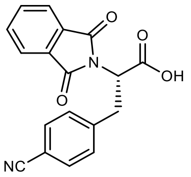

(*S*)-3-(4-Cyanophenyl)-2-(1,3-dioxoisoindolin-2-yl)propanoic acid (1g)

White solid, 159.9 mg (0.50 mmol, 50% yield); m.p. 163–165 °C; ^{1}H NMR (500 MHz, $CDCl_3$): δ = 7.82 – 7.77 (m, 2H), 7.74 – 7.69 (m, 2H), 7.50 (d, *J* = 8.2 Hz, 2H), 7.30 (d, *J* = 8.2 Hz, 2H), 5.23 (t, *J* = 8.3 Hz, 1H), 3.65 (d, *J* = 8.3 Hz, 2H); ^{13}C NMR (125 MHz, $CDCl_3$): δ = 173.8, 167.3, 142.0, 134.5, 132.5, 131.2, 129.7, 123.8, 118.6, 111.1, 52.3, 34.6; IR (cm^{-1}) 3188.

2225, 1781, 1701, 1607, 1398, 1158, 1119, 958, 723, 531; HRMS-EI (m/z) $[M]^+$ calcd for $C_{17}H_{12}ClNO_4$, 320.0797; found: 320.0793.

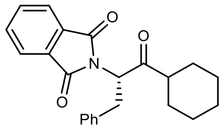

(*S*)-2-(1-Cyclohexyl-3-phenyl-1-oxopropan-2-yl)isoindoline-1,3-dione (3a)

White solid, 53.7 mg (0.148 mmol, 74% yield); m.p. 75–77 °C; ^{1}H NMR (400 MHz, $CDCl_3$): δ = 7.82 – 7.74 (m, 2H), 7.72 – 7.66 (m, 2H), 7.20 – 7.06 (m, 5H), 5.14 (dd, *J* = 11.1, 5.1 Hz, 1H), 3.58 – 3.31 (m, 2H), 2.54 (tt, *J* = 11.4, 3.4 Hz, 1H), 1.88 – 1.18 (m, 10H); ^{13}C NMR (125 MHz, $CDCl_3$): δ = 207.6, 167.8, 137.1, 134.2, 131.5, 128.9, 128.5, 126.8, 123.5, 58.7, 46.7, 33.7, 29.1, 28.2, 25.7, 25.4; IR (cm^{-1}) 2930, 2854, 1774, 1707, 1382, 717, 696, 529; HRMS-EI (m/z) $[M]^+$ calcd for $C_{23}H_{23}NO_3$, 361.1678; found: 361.1680.

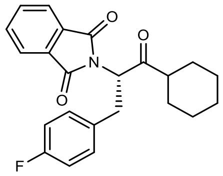

(*S*)-2-(1-Cyclohexyl-3-(4-fluorophenyl)-1-oxopropan-2-yl)isoindoline-1,3-dione (3b)

White solid, 47.8 mg (0.126 mmol, 63% yield); m.p. 86–88 °C; ^{1}H NMR (500 MHz, $CDCl_3$): δ = 7.82 – 7.74 (m, 2H), 7.74 – 7.67 (m, 2H), 7.12 – 7.06 (m, 2H), 6.87 – 6.80 (m, 2H), 5.09 (dd, *J* = 11.1, 5.1 Hz, 1H), 3.57 – 3.29 (m, 2H), 2.52 (tt, *J* = 11.4, 3.4 Hz, 1H), 1.87 – 1.72 (m, 4H), 1.66 – 1.59 (m, 1H), 1.53 – 1.40 (m, 1H), 1.34 – 1.17 (m, 4H); ^{13}C NMR (125 MHz, $CDCl_3$): δ = 207.4, 167.8, 161.7 (d, *J* = 245.0 Hz), 134.3, 132.8 (d, *J* = 3.2 Hz), 131.4, 130.4, 130.4, 123.6, 115.4 (d, *J* = 21.4 Hz), 58.5, 46.7, 32.9, 29.1, 28.1, 25.7, 25.7, 25.3; ^{19}F NMR (470 MHz, $CDCl_3$): δ = –116.0; IR (cm^{-1}) 2933, 2852, 1976, 1779, 1709, 1600, 1387, 1217, 974, 716, 526; HRMS-EI (m/z) $[M]^+$ calcd for $C_{23}H_{22}FNO_3$, 379.1584; found: 379.1582.

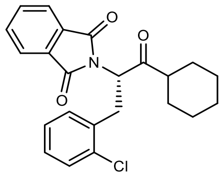

(*S*)-2-(1-Cyclohexyl-3-(2-chlorophenyl)-1-oxopropan-2-yl)isoindoline-1,3-dione (3c)

White solid, 34.3 mg (0.086 mmol, 43% yield); m.p. 125–127 °C; ^{1}H NMR (400 MHz, $CDCl_3$): δ = 7.85 – 7.74 (m, 2H), 7.73 – 7.64 (m, 2H), 7.32 – 7.27 (m, 1H), 7.13 – 7.01 (m, 2H), 7.01 – 6.91 (m, 1H), 5.30 (dd, *J* = 11.3, 4.3 Hz, 1H), 3.78 – 3.64 (m, 1H), 3.54 – 3.42 (m, 1H), 2.58 (tt, *J* = 11.4, 3.4 Hz, 1H), 1.90 – 1.81 (m, 2H), 1.81 – 1.71 (m, 2H), 1.65 – 1.58 (m, 1H), 1.50 – 1.19 (m, 5H); ^{13}C NMR (100 MHz, $CDCl_3$): δ = 207.3, 167.8, 134.9, 134.3, 134.2, 131.5, 131.3, 129.7, 128.5, 126.8, 123.5, 56.5, 46.7, 31.9, 29.1, 28.2, 25.7, 25.7, 25.4; IR (cm^{-1}) 2935, 2856, 2158, 1976, 1775, 1707, 1471, 1386, 761, 719, 676, 528; HRMS-EI (m/z) $[M]^+$ calcd for $C_{23}H_{22}ClNO_3$, 395.1288; found: 395.1285.

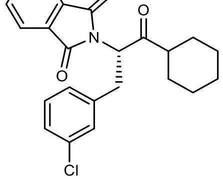

(*S*)-2-(1-Cyclohexyl-3-(3-chlorophenyl)-1-oxopropan-2-yl)isoindoline-1,3-dione (3d)

White solid, 52.8 mg (0.134 mmol, 67% yield); m.p. 93–95 °C; ^{1}H NMR (400 MHz, $CDCl_3$): δ = 7.82 – 7.75 (m, 2H), 7.75 – 7.67 (m, 2H), 7.13 – 7.10 (m, 1H), 7.10 – 7.06 (m, 2H), 7.05 – 6.99 (m, 1H), 5.09 (dd, *J* = 10.9, 5.1 Hz, 1H), 3.55 – 3.46 (m, 1H), 3.43 – 3.30 (m, 1H), 2.51 (tt, *J* = 11.4, 3.3 Hz, 1H), 1.89 – 1.72 (m, 4H), 1.65 – 1.58 (m, 1H), 1.54 – 1.40 (m, 1H), 1.38

– 1.16 (m, 4H); ^{13}C NMR (100 MHz, CDCl$_3$): δ = 207.2, 167.8, 139.2, 134.3, 134.3, 131.4, 129.8, 129.1, 127.1, 127.0, 123.6; IR (cm^{-1}) 2933, 2850, 1774, 1706, 1597, 1444, 1386, 1080, 872, 782, 717, 543, 529; HRMS-EI (m/z) [M]$^+$ calcd for $C_{23}H_{22}ClNO_3$, 395.1288; found: 395.1291.

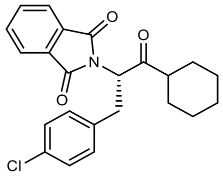

(*S*)-2-(1-Cyclohexyl-3-(4-chlorophenyl)-1-oxopropan-2-yl)isoindoline-1,3-dione (3e)

White solid, 46.5 mg (0.118 mmol, 59% yield); m.p. 90–92 °C; ^{1}H NMR (500 MHz, CDCl$_3$): δ = 7.86 – 7.75 (m, 2H), 7.75 – 7.66 (m, 2H), 7.17 – 7.11 (m, 2H), 7.10 – 7.04 (m, 2H), 5.10 (dd, *J* = 11.1, 5.1 Hz, 1H), 3.56 – 3.30 (m, 2H), 2.52 (tt, *J* = 11.4, 3.3 Hz, 1H), 1.89 – 1.72 (m, 4H), 1.65 – 1.62 (m, 1H), 1.51 – 1.18 (m, 5H); ^{13}C NMR (125 MHz, CDCl$_3$): δ = 207.3, 167.8, 135.6, 134.3, 132.6, 131.4, 130.2, 128.7, 123.6, 58.3, 46.7, 33.1, 29.1, 28.1, 25.7, 25.6, 25.3; IR (cm^{-1}) 2930, 2853, 1776, 1708, 1491, 1383, 1089, 877, 754, 719, 530, 504; HRMS-EI (m/z) [M]$^+$ calcd for $C_{23}H_{22}ClNO_3$, 395.1288; found: 395.1289.

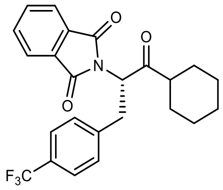

(*S*)-2-(1-Cyclohexyl-1-oxo-3-(4-(trifluoromethyl)phenyl)propan-2-yl)isoindoline-1,3-dione (3f)

White solid, 57.0 mg (0.133 mmol, 66% yield); m.p. 129–131 °C; ^{1}H NMR (400 MHz, CDCl$_3$): δ = 7.84 – 7.78 (m, 2H), 7.77 – 7.67 (m, 2H), 7.46 – 7.41 (m, 2H), 7.29 – 7.26 (m, 2H), 5.15 (dd, *J* = 10.9, 5.2 Hz, 1H), 3.67 – 3.41 (m, 2H), 2.52 (tt, *J* = 11.4, 3.3 Hz, 1H), 1.90 – 1.69 (m, 4H), 1.66 – 1.59 (m, 1H), 1.56 – 1.40 (m, 1H), 1.40 – 1.13 (m, 4H); ^{13}C NMR (100 MHz, CDCl$_3$): δ = 207.1, 167.8, 141.4, 134.4, 131.4, 129.3, 125.47 (q, *J* = 3.7 Hz), 123.6, 58.0, 46.6, 33.5, 29.2, 28.1, 25.7, 25.6, 25.3; ^{19}F NMR (375 MHz, CDCl$_3$): δ = –62.5; IR (cm^{-1}) 2933, 2856, 1775, 1710, 1616, 1384, 1323, 1163, 1122, 1066, 880, 743, 722, 530; HRMS-EI (m/z) [M]$^+$ calcd for $C_{24}H_{22}F_3NO_3$, 429.1553; found: 429.1552.

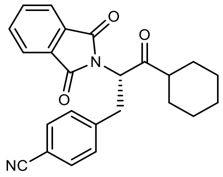

(*S*)-4-(3-Cyclohexyl-2-(1,3-dioxoisoindolin-2-yl)-3-oxopropyl)benzonitrile (3g)

White solid, 41.2 mg (0.107 mmol, 53% yield); m.p. 65–67 °C; ^{1}H NMR (400 MHz, CDCl$_3$): δ = 7.83 – 7.77 (m, 2H), 7.76 – 7.70 (m, 2H), 7.49 – 7.43 (m, 2H), 7.30 – 7.22 (m, 2H), 5.11 (dd, *J* = 10.8, 5.2 Hz, 1H), 3.70 – 3.25 (m, 2H), 2.49 (tt, *J* = 11.4, 3.2 Hz, 1H), 1.87 – 1.72 (m, 4H), 1.67 – 1.57 (m, 1H), 1.53 – 1.42 (m, 1H), 1.35 – 1.15 (m, 4H); ^{13}C NMR (100 MHz, CDCl$_3$): δ = 206.9, 167.7, 142.9, 134.5, 132.3, 131.2, 129.8, 123.7, 118.7, 110.8, 57.6, 46.6, 33.9, 29.3, 28.0, 25.7, 25.6, 25.2; IR (cm^{-1}) 2930, 2855, 2227, 1773, 1709, 1608, 1383, 1124, 1078, 879, 719, 555, 529; HRMS-EI (m/z) [M]$^+$ calcd for $C_{24}H_{22}N_2O_3$, 386.1633; found: 386.1630.

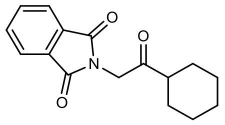

2-(2-Cyclohexyl-2-oxoethyl)isoindoline-1,3-dione (3h)

White solid, 39.0 mg (0.144 mmol, 72% yield); m.p. 120–122 °C; ^{1}H NMR (500 MHz, $CDCl_3$): δ = 7.89 – 7.82 (m, 2H), 7.76 – 7.67 (m, 2H), 4.53 (s, 2H), 2.51 (tt, *J* = 11.4, 3.5 Hz, 1H), 2.00 – 1.92 (m, 2H), 1.82 (dt, *J* = 12.7, 3.6 Hz, 2H), 1.71 – 1.65 (m, 1H), 1.50 – 1.40 (m, 2H), 1.37 – 1.21 (m, 3H); ^{13}C NMR (125 MHz, $CDCl_3$): δ = 204.9, 167.8, 134.1, 132.2, 123.5, 48.4, 45.0, 28.2, 25.7, 25.5; IR (cm^{-1}) 2926, 2849, 1775, 1712, 1464, 1420, 1300, 1115, 1071, 947, 714, 523; HRMS-EI (m/z) $[M]^+$ calcd for $C_{16}H_{17}NO_3$, 271.1208; found: 271.1211.

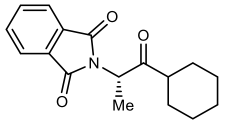

(*S*)-2-(1-Cyclohexyl-1-oxopropan-2-yl)isoindoline-1,3-dione (3i)

White oil, 25.1 mg (0.088 mmol, 44% yield); ^{1}H NMR (500 MHz, $CDCl_3$): δ = 7.89 – 7.83 (m, 2H), 7.77 – 7.71 (m, 2H), 4.95 (q, *J* = 7.3 Hz, 1H), 2.55 (tt, *J* = 11.4, 3.3 Hz, 1H), 1.90 – 1.72 (m, 4H), 1.71 – 1.59 (m, 4H), 1.49 – 1.28 (m, 2H), 1.28 – 1.17 (m, 3H); ^{13}C NMR (125 MHz, $CDCl_3$): δ = 208.2, 167.9, 134.2, 132.0, 123.5, 52.7, 46.1, 29.0, 28.3, 25.7, 25.6, 25.4, 14.3; IR (cm^{-1}) 2930, 2854, 1777, 1707, 1449, 1383, 1053, 988, 878, 717, 529; HRMS-EI (m/z) $[M]^+$ calcd for $C_{17}H_{19}NO_3$, 285.1365; found: 285.1361.

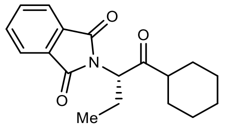

(*S*)-2-(1-Cyclohexyl-1-oxobutan-2-yl)isoindoline-1,3-dione (3j)

White oil, 26.4 mg (0.088 mmol, 44% yield); ^{1}H NMR (500 MHz, $CDCl_3$): δ = 7.90 – 7.84 (m, 2H), 7.77 – 7.71 (m, 2H), 4.78 (dd, *J* = 9.1, 6.6 Hz, 1H), 2.54 (tt, *J* = 11.4, 3.3 Hz, 1H), 2.27 – 2.17 (m, 2H), 1.85 – 1.74 (m, 3H), 1.66 – 1.62 (m, 2H), 1.45 – 1.19 (m, 5H), 0.93 (t, *J* = 7.4 Hz, 3H); ^{13}C NMR (125 MHz, $CDCl_3$): δ = 207.9, 168.2, 134.2, 131.8, 123.5, 59.4, 46.6, 28.8, 28.4, 25.7, 25.6, 25.5, 21.0, 11.2; IR (cm^{-1}) 2930, 2855, 1774, 1708, 1467, 1450, 1382, 1052, 988, 878, 743, 717, 530; HRMS-EI (m/z) $[M]^+$ calcd for $C_{18}H_{21}NO_3$, 299.1521; found: 299.1520.

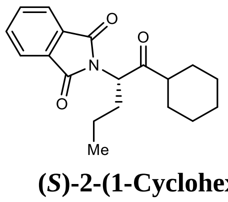

(*S*)-2-(1-Cyclohexyl-1-oxopentan-2-yl)isoindoline-1,3-dione (3k)

White oil, 32.7 mg (0.104 mmol, 52% yield); ^{1}H NMR (500 MHz, $CDCl_3$): δ = 7.88 – 7.83 (m, 2H), 7.77 – 7.72 (m, 2H), 4.87 (dd, *J* = 11.3, 4.3 Hz, 1H), 2.55 (tt, *J* = 11.4, 3.4 Hz, 1H), 2.31 – 2.20 (m, 1H), 2.13 – 2.02 (m, 1H), 1.85 – 1.74 (m, 4H), 1.68 – 1.59 (m, 2H), 1.47 – 1.14 (m, 6H), 0.94 (t, *J* = 7.4 Hz, 3H); ^{13}C NMR (125 MHz, $CDCl_3$): δ = 208.1, 168.2, 134.2, 131.8, 123.5, 57.7, 46.5, 29.5, 28.8, 28.5, 25.7, 25.6, 25.5, 19.8, 13.5; IR (cm^{-1}) 2930, 2855, 1774, 1708, 1467, 1450, 1382, 1052, 743, 717, 530; HRMS-EI (m/z) $[M]^+$ calcd for $C_{19}H_{23}NO_3$, 313.1678; found: 313.1680.

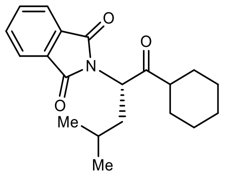

(*S*)-2-(1-Cyclohexyl-4-methyl-1-oxopentan-2-yl)isoindoline-1,3-dione (3l)

White oil, 35.0 mg (0.107 mmol, 53% yield); ^{1}H NMR (500 MHz, $CDCl_3$): δ = 7.88 – 7.82 (m, 2H), 7.77 – 7.70 (m, 2H), 4.95 (dd, *J* = 11.8, 4.1 Hz, 1H), 2.55 (tt, *J* = 11.4, 3.4 Hz, 1H), 2.40 – 2.29 (m, 1H), 1.87 – 1.71 (m, 4H), 1.64 (d, *J* = 9.5 Hz, 1H), 1.54 – 1.30 (m, 3H), 1.28 – 1.13 (m, 4H), 0.95 (dd, *J* = 11.2, 6.6 Hz, 6H); ^{13}C NMR (125 MHz, $CDCl_3$): δ = 208.2, 168.2, 134.2, 131.9, 123.5, 56.6, 46.5, 36.1, 28.7, 28.6, 25.7, 25.6, 25.5, 25.4, 23.4, 21.0; IR (cm^{-1}) 2929, 2855, 1774, 1708, 1468, 1450, 1382, 1063, 717, 530; HRMS-EI (m/z) $[M]^+$ calcd for $C_{20}H_{25}NO_3$, 327.1834; found: 327.1835.

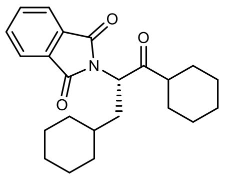

(*S*)-2-(1,3-Dicyclohexyl-1-oxopropan-2-yl)isoindoline-1,3-dione (3m)

White viscous solid, 44.0 mg (0.120 mmol, 60% yield); ^{1}H NMR (500 MHz, $CDCl_3$): δ = 7.90 – 7.82 (m, 2H), 7.78 – 7.70 (m, 2H), 4.99 (dd, *J* = 11.7, 4.1 Hz, 1H), 2.54 (tt, *J* = 11.4, 3.4 Hz, 1H), 2.32 – 2.25 (m, 1H), 1.94 – 1.85 (m, 2H), 1.84 – 1.72 (m, 4H), 1.72 – 1.55 (m, 6H), 1.44 – 1.31 (m, 2H), 1.26 – 1.11 (m, 6H), 1.04 – 0.86 (m, 2H); ^{13}C NMR (125 MHz, $CDCl_3$): δ = 208.3, 168.3, 134.1, 131.9, 123.5, 56.0, 46.5, 34.7, 34.7, 33.9, 31.8, 28.7, 28.6, 26.4, 26.2, 25.9, 25.7, 25.6, 25.5; IR (cm^{-1}) 2922, 2851, 1775, 1709, 1467, 1448, 1382, 1067, 888, 717; HRMS-EI (m/z) $[M]^+$ calcd for $C_{23}H_{29}NO_3$, 367.2147; found: 367.2145.

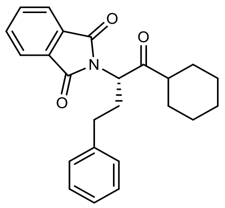

(*S*)-2-(1-Cyclohexyl-1-oxo-4-phenylbutan-2-yl)isoindoline-1,3-dione (3n)

White viscous solid, 38.1 mg (0.102 mmol, 51% yield); ^{1}H NMR (500 MHz, $CDCl_3$): δ = 7.86 – 7.79 (m, 2H), 7.77 – 7.70 (m, 2H), 7.25 – 7.17 (m, 2H), 7.17 – 7.12 (m, 2H), 7.12 – 7.06 (m, 1H), 4.92 – 4.83 (m, 1H), 2.70 – 2.59 (m, 3H), 2.54 – 2.40 (m, 2H), 1.83 – 1.69 (m, 4H), 1.63 – 1.61 (m, 2H), 1.45 – 1.24 (m, 1H), 1.24 – 1.14 (m, 3H); ^{13}C NMR (125 MHz, $CDCl_3$): δ = 207.7, 168.2, 140.4, 134.2, 131.8, 128.4, 128.4, 126.1, 123.5, 57.6, 46.4, 33.0, 28.9, 28.8, 28.4, 25.7, 25.6, 25.4; IR (cm^{-1}) 2928, 2853, 1774, 1708, 1467, 1450, 1383, 757, 717, 698, 529; HRMS-EI (m/z) $[M]^+$ calcd for $C_{24}H_{25}NO_3$, 375.1834; found: 375.1831.

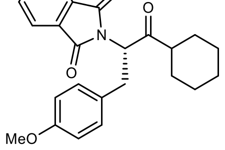

(*S*)-2-(1-Cyclohexyl-3-(4-methoxyphenyl)-1-oxopropan-2-yl)isoindoline-1,3-dione (3o)

White viscous solid, 33.5 mg (0.086 mmol, 43% yield); ^{1}H NMR (500 MHz, $CDCl_3$): δ = 7.81 – 7.73 (m, 2H), 7.73 – 7.66 (m, 2H), 7.04 (d, *J* = 8.2 Hz, 2H), 6.69 (d, *J* = 8.4 Hz, 2H), 5.09 (dd, *J* = 11.1, 5.0 Hz, 1H), 3.69 (s, 3H), 3.50 – 3.44 (m, 1H), 3.40 – 3.33 (m, 1H), 2.54 (tt, *J* = 11.4, 3.4 Hz, 1H), 1.89 – 1.68 (m, 4H), 1.65 – 1.60 (m, 1H), 1.51 – 1.43 (m, 1H), 1.39 – 1.28

(m, 1H), 1.26 – 1.17 (m, 3H); ^{13}C NMR (125 MHz, $CDCl_3$): δ = 207.7, 167.9, 158.3, 134.1, 131.6, 129.8, 129.0, 123.5, 113.9, 58.9, 55.1, 46.7, 32.8, 29.0, 28.3, 25.7, 25.7, 25.4; IR (cm^{-1}) 2930, 2854, 1774, 1708, 1612, 1512, 1382, 1246, 1177, 1032, 716, 529; HRMS-EI (m/z) [M]$^+$ calcd for $C_{24}H_{25}NO_4$, 391.1784; found: 391.1784.

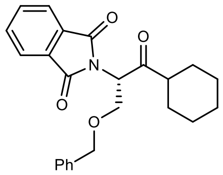

(*S*)-2-(3-(Benzyloxy)-1-cyclohexyl-1-oxopropan-2-yl)isoindoline-1,3-dione (3p)

White viscous solid, 39.1 mg (0.100 mmol, 50% yield); ^{1}H NMR (500 MHz, $CDCl_3$): δ = 7.91 – 7.83 (m, 2H), 7.79 – 7.71 (m, 2H), 7.26 – 7.16 (m, 5H), 5.18 (dd, *J* = 8.9, 5.3 Hz, 1H), 4.58 – 4.43 (m, 2H), 4.20 – 4.14 (m, 1H), 4.13 – 4.03 (m, 1H), 2.51 (tt, *J* = 11.4, 3.4 Hz, 1H), 1.89 – 1.54 (m, 5H), 1.49 – 1.36 (m, 1H), 1.34 – 1.11 (m, 4H); ^{13}C NMR (125 MHz, $CDCl_3$): δ = 207.2, 167.9, 137.6, 134.2, 131.9, 128.4, 127.7, 127.6, 123.6, 73.0, 66.4, 56.6, 46.9, 28.9, 27.9, 25.7, 25.7, 25.3; IR (cm^{-1}) 2930, 2854, 1776, 1710, 1450, 1384, 1269, 1097, 714, 529; HRMS-EI (m/z) [M]$^+$ calcd for $C_{24}H_{25}NO_4$, 391.1784; found: 391.1779.

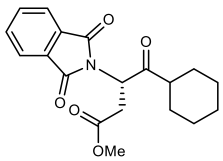

Methyl (*S*)-4-cyclohexyl-3-(1,3-dioxoisoindolin-2-yl)-4-oxobutanoate (3q)

White viscous solid, 37.3 mg (0.109 mmol, 54% yield); ^{1}H NMR (500 MHz, $CDCl_3$): δ = 7.93 – 7.85 (m, 2H), 7.79 – 7.70 (m, 2H), 5.34 (dd, *J* = 8.1, 6.3 Hz, 1H), 3.65 (s, 3H), 3.33 – 3.24 (m, 1H), 2.92 – 2.84 (m, 1H), 2.43 (tt, *J* = 11.3, 3.4 Hz, 1H), 1.92 – 1.85 (m, 1H), 1.75 – 1.60 (m, 4H), 1.52 – 1.41 (m, 1H), 1.32 – 1.11 (m, 4H); ^{13}C NMR (125 MHz, $CDCl_3$): δ = 206.6, 171.0, 167.5, 134.5, 131.7, 123.8, 52.8, 52.1, 46.1, 32.6, 29.5, 27.6, 25.8, 25.6, 25.1; IR (cm^{-1}) 2930, 2854, 1777, 1710, 1384, 1173, 978, 876, 719, 529; HRMS-EI (m/z) [M]$^+$ calcd for $C_{19}H_{21}NO_5$, 343.1420; found: 343.1416.

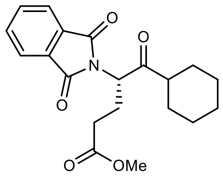

Methyl (*S*)-5-cyclohexyl-4-(1,3-dioxoisoindolin-2-yl)-5-oxopentanoate (3r)

White oil, 19.4 mg (0.054 mmol, 27% yield); ^{1}H NMR (500 MHz, $CDCl_3$): δ = 7.90 – 7.84 (m, 2H), 7.78 – 7.73 (m, 2H), 4.92 (dd, *J* = 10.7, 4.5 Hz, 1H), 3.62 (s, 3H), 2.59 – 2.36 (m, 4H), 1.89 – 1.72 (m, 3H), 1.65 – 1.62 (m, 2H), 1.50 – 1.37 (m, 1H), 1.36 – 1.16 (m, 5H); ^{13}C NMR (125 MHz, $CDCl_3$): δ = 207.3, 172.8, 168.1, 134.3, 131.7, 123.6, 56.7, 51.8, 46.5, 30.7, 28.9, 28.3, 25.7, 25.6, 25.4, 23.0; IR (cm^{-1}) 2929, 2854, 1776, 1710, 1438, 1383, 1172. 719, 530; HRMS-EI (m/z) [M]$^+$ calcd for $C_{20}H_{23}NO_5$, 357.1576; found: 357.1576.

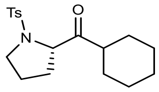

(*S*)-Cyclohexyl(1-tosylpyrrolidin-2-yl)methanone (3s)

White viscous solid, 26.6 mg (0.079 mmol, 40% yield); ^{1}H NMR (500 MHz, $CDCl_3$, 10:1 mixture of rotamers, reported only for the major rotamer): δ = 7.74 – 7.70 (m, 2H), 7.35 – 7.28 (m, 2H), 4.42 – 4.32 (m, 1H), 3.46 – 3.31 (m, 2H), 2.79 (tt, *J* = 11.3, 3.4 Hz, 1H), 2.42 (s, 3H), 1.92 – 1.78 (m, 6H), 1.73 – 1.63 (m, 2H), 1.51 – 1.11 (m, 6H); ^{13}C NMR (125 MHz, $CDCl_3$, mixture of rotamers): δ = 212.1, 143.7, 135.0, 134.5, 130.0, 129.9, 129.7, 127.8, 127.6, 67.6, 65.5, 49.2, 48.8, 47.2, 31.7, 29.5,

29.2, 28.3, 25.8, 25.7, 25.5, 24.7, 24.5, 21.6, 21.3; IR (cm^{-1}) 2928, 2854, 1718, 1597, 1448, 1344, 1156, 1093, 815, 662, 588, 546; HRMS-EI (m/z) [M]$^+$ calcd for $C_{18}H_{25}NO_3S$, 335.1555; found: 335.1553.

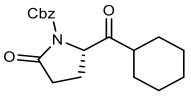

Benzyl (*S*)-2-(cyclohexanecarbonyl)-5-oxopyrrolidine-1-carboxylate (3t)

White solid, 51.1 mg (0.155 mmol, 78% yield); m.p. 102–104 °C; ^{1}H NMR (400 MHz, $CDCl_3$): δ = 7.39 – 7.28 (m, 5H), 5.24 – 5.14 (m, 2H), 4.87 (dd, *J* = 9.7, 2.8 Hz, 1H), 2.62 – 2.38 (m, 3H), 2.31 – 2.17 (m, 1H), 1.90 – 1.60 (m, 6H), 1.50 – 1.36 (m, 1H), 1.28 – 1.06 (m, 4H); ^{13}C NMR (100 MHz, $CDCl_3$): δ = 209.1, 173.2, 150.9, 134.9, 128.6, 128.5, 128.4, 68.4, 62.1, 48.1, 30.8, 28.7, 27.8, 25.6, 25.6, 25.3, 20.6; IR (cm^{-1}) 2930, 2853, 1781, 1713, 1698, 1449, 1386, 1288, 1190, 755, 698, 590, 499; HRMS-EI (m/z) [M]$^+$ calcd for $C_{19}H_{23}NO_4$, 329.1627; found: 329.1629.

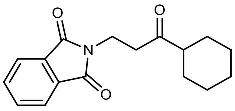

2-(3-Cyclohexyl-3-oxopropyl)isoindoline-1,3-dione (3u)

White solid, 44.1 mg (0.155 mmol, 77% yield); m.p. 140–142 °C; ^{1}H NMR (500 MHz, $CDCl_3$): δ = 7.85 – 7.78 (m, 2H), 7.75 – 7.65 (m, 2H), 3.99 – 3.84 (m, 2H), 2.91 – 2.79 (m, 2H), 2.32 (tt, *J* = 11.3, 3.4 Hz, 1H), 1.86 – 1.79 (m, 2H), 1.79 – 1.69 (m, 2H), 1.68 – 1.60 (m, 1H), 1.35 – 1.17 (m, 5H); ^{13}C NMR (125 MHz, $CDCl_3$): δ = 211.2, 168.1, 134.0, 132.1, 123.2, 50.8, 38.4, 33.2, 28.3, 25.8, 25.6; IR (cm^{-1}) 2923, 2850, 1771, 1711, 1695, 1447, 1368, 1188, 983, 717, 532; HRMS-EI (m/z) [M]$^+$ calcd for $C_{17}H_{19}NO_3$, 285.1365; found: 285.1362.

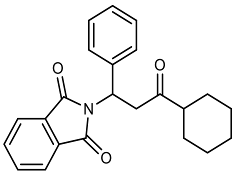

2-(3-Cyclohexyl-3-oxo-1-phenylpropyl)isoindoline-1,3-dione (3v)

White oil, 58.9 mg (0.163 mmol, 82% yield); ^{1}H NMR (400 MHz, $CDCl_3$): δ = 7.80 – 7.74 (m, 2H), 7.67 – 7.62 (m, 2H), 7.53 – 7.49 (m, 2H), 7.34 – 7.28 (m, 2H), 7.27 – 7.22 (m, 1H), 5.84 (dd, *J* = 9.8, 5.4 Hz, 1H), 4.09 – 3.97 (m, 1H), 3.35 – 3.24 (m, 1H), 2.36 (tt, *J* = 11.0, 3.5 Hz, 1H), 1.89 – 1.67 (m, 4H), 1.68 – 1.57 (m, 1H), 1.35 – 1.04 (m, 5H); ^{13}C NMR (100 MHz, $CDCl_3$): δ = 210.6, 168.3, 139.4, 133.9, 131.8, 128.7, 128.0, 127.8, 123.2, 50.9, 50.2, 42.0, 28.3, 28.3, 25.8, 25.6; IR (cm^{-1}) 2928, 2853, 1772, 1704, 1495, 1387, 1352, 1331, 1073, 718, 697, 530; HRMS-EI (m/z) [M]$^+$ calcd for $C_{23}H_{23}NO_3$, 361.1678; found: 361.1678.

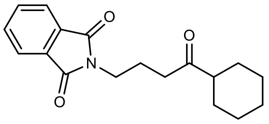

2-(4-Cyclohexyl-4-oxobutyl)isoindoline-1,3-dione (3w)

White solid, 42.3 mg (0.141 mmol, 71% yield); m.p. 87–89 °C; ^{1}H NMR (500 MHz, $CDCl_3$): δ = 7.89 – 7.79 (m, 2H), 7.74 – 7.66 (m, 2H), 3.69 (t, *J* = 6.8 Hz, 2H), 2.50 (t, *J* = 7.2 Hz, 2H), 2.34 – 2.27 (m, 1H), 1.93 (p, *J* = 7.0 Hz, 2H), 1.83 – 1.71 (m, 4H), 1.68 – 1.57 (m, 1H), 1.34 – 1.08 (m, 5H); ^{13}C NMR (125 MHz, $CDCl_3$): δ = 212.8, 168.5, 133.9, 132.1, 123.2, 50.7, 37.7, 37.4, 28.5, 25.8, 25.7, 22.6; IR (cm^{-1}) 2933, 2850, 1769, 1696, 1614, 1439, 1394, 1354, 1068, 1004, 876, 712, 526; HRMS-EI (m/z) [M]$^+$ calcd for $C_{18}H_{21}NO_3$, 299.1521; found: 299.1520.

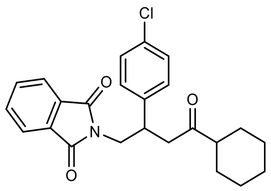

2-(2-(4-Chlorophenyl)-4-cyclohexyl-4-oxobutyl)isoindoline-1,3-dione (3x)

White solid, 60.8 mg (0.149 mmol, 74% yield); m.p. 143–145 °C; ^{1}H NMR (400 MHz, $CDCl_3$): δ = 7.84 – 7.75 (m, 2H), 7.73 – 7.65 (m, 2H), 7.23 – 7.17 (m, 4H), 3.86 – 3.76 (m, 3H), 2.94 – 2.76 (m, 2H), 2.29 – 2.12 (m, 1H), 1.73 – 1.54 (m, 4H), 1.28 – 0.99 (m, 6H); ^{13}C NMR (100 MHz, $CDCl_3$): δ = 211.3, 168.2, 139.9, 134.0, 132.7, 131.8, 129.1, 128.7, 123.3, 51.0, 44.7, 43.0, 38.7, 28.3, 28.0, 25.7, 25.6, 25.5; IR (cm^{-1}) 2926, 2852, 1773, 1704, 1613, 1494, 1392, 1123, 1068, 1012, 826, 710, 673, 529; HRMS-EI (m/z) [M]$^+$ calcd for $C_{24}H_{24}ClNO_3$, 409.1445; found: 409.1447.

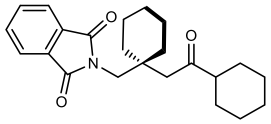

2-((1-(2-Cyclohexyl-2-oxoethyl)cyclohexyl)methyl)isoindoline-1,3-dione (3y)

White solid, 49.4 mg (0.135 mmol, 67% yield); m.p. 91–93 °C; ^{1}H NMR (500 MHz, $CDCl_3$): δ = 7.88 – 7.79 (m, 2H), 7.75 – 7.66 (m, 2H), 3.81 (s, 2H), 2.54 (s, 2H), 2.31 (tt, *J* = 11.2, 3.4 Hz, 1H), 1.90 – 1.83 (m, 2H), 1.81 – 1.73 (m, 2H), 1.71 – 1.56 (m, 6H), 1.51 – 1.44 (m, 1H), 1.41 – 1.13 (m, 9H); ^{13}C NMR (125 MHz, $CDCl_3$): δ = 213.0, 169.3, 133.9, 132.1, 123.2, 51.8, 45.9, 44.5, 38.2, 33.7, 28.7, 26.0, 25.9, 25.8, 21.6; IR (cm^{-1}) 2921, 2848, 1768, 1700, 1434, 1390, 1049, 913, 723, 628, 531; HRMS-EI (m/z) [M]$^+$ calcd for $C_{23}H_{29}NO_3$, 367.2147; found: 367.2150.

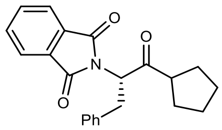

(*S*)-2-(1-Cyclopentyl-1-oxo-3-phenylpropan-2-yl)isoindoline-1,3-dione (4a)

White solid, 47.5 mg (0.137 mmol, 68% yield); m.p. 62–64 °C; ^{1}H NMR (400 MHz, $CDCl_3$): δ = 7.83 – 7.73 (m, 2H), 7.73 – 7.64 (m, 2H), 7.21 – 7.08 (m, 5H), 5.11 (dd, *J* = 11.3, 4.9 Hz, 1H), 3.62 – 3.37 (m, 2H), 3.09 – 2.97 (m, 1H), 1.95 – 1.46 (m, 8H); ^{13}C NMR (100 MHz, $CDCl_3$): δ = 207.5, 167.8, 137.2, 134.2, 131.5, 128.8, 128.5, 126.7, 123.5, 59.8, 47.4, 33.7, 30.1, 29.2, 26.2, 26.1; IR (cm^{-1}) 2959, 2865, 1777, 1706, 1380, 1096, 1068, 712, 703, 529; HRMS-EI (m/z) [M]$^+$ calcd for $C_{22}H_{21}NO_3$, 347.1519; found: 347.1521.

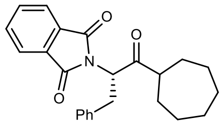

(*S*)-2-(1-Cycloheptyl-1-oxo-3-phenylpropan-2-yl)isoindoline-1,3-dione (5a)

White solid, 42.9 mg (0.114 mmol, 57% yield); m.p. 82–84 °C; ^{1}H NMR (400 MHz, $CDCl_3$): δ = 7.83 – 7.73 (m, 2H), 7.73 – 7.63 (m, 2H), 7.21 – 7.07 (m, 5H), 5.17 (dd, *J* = 11.0, 5.1 Hz, 1H), 3.59 – 3.36 (m, 2H), 2.72 (tt, *J* = 9.1, 4.3 Hz, 1H), 1.91 – 1.29 (m, 12H); ^{13}C NMR (100 MHz, $CDCl_3$): δ = 208.2, 167.8, 137.2, 134.2, 131.5, 128.9, 128.5, 126.8, 123.5, 58.9, 47.8, 33.7, 30.4, 29.8, 28.3, 26.5, 26.4.; IR (cm^{-1}) 2927, 2855, 1771, 1705, 1466, 1383, 981, 720, 698, 528; HRMS-EI (m/z) [M]$^+$ calcd for $C_{24}H_{25}NO_3$, 375.1837; found: 375.1834.

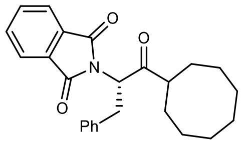

(*S*)-2-(1-Cyclooctyl-1-oxo-3-phenylpropan-2-yl)isoindoline-1,3-dione (6a)

White solid, 51.4 mg (0.132 mmol, 66% yield); m.p. 88–90 °C; ^{1}H NMR (400 MHz, $CDCl_3$): δ = 7.82 – 7.72 (m, 2H), 7.72 – 7.63 (m, 2H), 7.24 – 6.96 (m, 5H), 5.17 (dd, *J* = 11.0, 5.2 Hz, 1H), 3.69 – 3.22 (m, 2H), 2.98 – 2.62 (m, 1H), 1.88 – 1.30 (m, 14H); ^{13}C NMR (100 MHz, $CDCl_3$): δ 208.4, 167.8, 137.2, 134.2, 131.5, 128.8, 128.6, 126.8, 123.5, 58.9, 46.1, 33.7, 28.4, 28.0, 26.7, 26.4, 26.3, 25.4, 25.3; IR (cm^{-1}) 2921, 2854, 1774, 1708, 1468, 1382, 872, 717, 699, 530; HRMS-EI (m/z) $[M]^+$ calcd for $C_{25}H_{27}NO_3$, 389.1989; found: 389.1991.

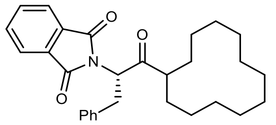

(*S*)-2-(1-Cyclododecyl-1-oxo-3-phenylpropan-2-yl)isoindoline-1,3-dione (7a)

White solid, 48.6 mg (0.109 mmol, 55% yield); m.p. 96–98 °C; ^{1}H NMR (500 MHz, $CDCl_3$): δ = 7.82 – 7.74 (m, 2H), 7.73 – 7.66 (m, 2H), 7.22 – 7.07 (m, 5H), 5.13 (dd, *J* = 11.2, 5.1 Hz, 1H), 3.62 – 3.39 (m, 2H), 2.87 – 2.78 (m, 1H), 1.72 – 1.04 (m, 22H); ^{13}C NMR (125 MHz, $CDCl_3$): δ = 208.3, 167.8, 137.2, 134.2, 131.6, 128.9, 128.6, 126.7, 123.4, 59.2, 42.8, 33.6, 27.2, 25.3, 24.0, 23.7, 23.5, 23.5, 23.1, 23.0, 22.7, 22.2; IR (cm^{-1}) 2928, 2857, 1773, 1707, 1498, 1387, 1362, 1130, 748, 717, 105, 529; HRMS-EI (m/z) $[M]^+$ calcd for $C_{29}H_{35}NO_3$, 445.2619; found: 445.2617.

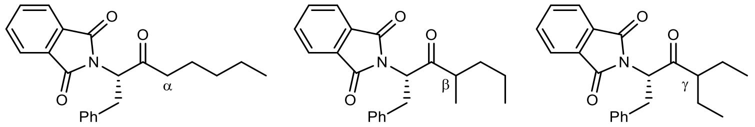

(α:β:γ = 1.0:6.0:3.3, 5% terminal selectivity after statistical correction)

(*S*)-2-(3-Oxo-1-phenyloctan-2-yl)isoindoline-1,3-dione (8aα)
(*S*)-2-(4-Methyl-3-oxo-1-phenylheptan-2-yl)isoindoline-1,3-dione (8aβ)
(*S*)-2-(4-Ethyl-3-oxo-1-phenylhexan-2-yl)isoindoline-1,3-dione (8aγ)

White oil, 35.2 mg (0.108 mmol, 50% yield); ^{1}H NMR (500 MHz, $CDCl_3$, mixture of regioisomers): δ = 7.81 – 7.74 (m, 2H), 7.74 – 7.65 (m, 2H), 7.21 – 7.08 (m, 5H), 5.15 (dd, *J* = 11.2, 5.1 Hz, 0.3H), 5.12 – 5.07 (m, 0.6H), 4.99 (dd, *J* = 11.3, 4.9 Hz, 0.1H), 3.63 – 3.37 (m, 2H), 2.81 – 2.71 (m, 0.6H), 2.61 (p, *J* = 6.3 Hz, 0.3H), 2.48 (t, *J* = 7.3 Hz, 0.2H), 1.77 – 1.58 (m, 1.5H), 1.56 – 1.41 (m, 0.5H), 1.37 – 1.20 (m, 3H), 1.09 (dd, *J* = 12.0, 6.9 Hz, 2H), 0.99 – 0.81 (m, 4H); ^{13}C NMR (125 MHz, $CDCl_3$, mixture of regioisomers): δ = 208.8, 208.4, 208.0, 204.8, 167.9, 167.8, 167.8, 137.1, 137.1, 134.2, 134.2, 134.1, 131.5, 131.5, 128.9, 128.8, 128.8, 128.6, 128.6, 128.5, 126.8, 126.8, 123.5, 123.4, 60.4, 60.4, 59.4, 59.4, 50.5, 41.9, 41.6, 39.1, 35.7, 35.0, 33.7, 33.6, 33.4, 33.2, 31.2, 30.3, 24.3, 23.7, 23.2, 22.4, 20.3, 20.3, 17.1, 16.2, 14.1, 14.0, 11.7, 11.5; IR (cm^{-1}) 2961, 1775, 1709, 1497, 1455, 1381, 970, 718, 698, 529; HRMS-EI (m/z) $[M]^+$ calcd for $C_{22}H_{23}NO_3$. 349.1678; found: 349.1675.

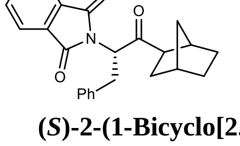

(*S*)-2-(1-Bicyclo[2.2.1]heptan-2-yl-1-oxo-3-phenylpropan-2-yl)isoindoline-1,3-dione (9a)

White solid, 58.5 mg (0.157 mmol, 78% yield); m.p. 91–93 °C; ^{1}H NMR (400 MHz, $CDCl_3$, 1:1 mixture of diastereomers): δ = 7.80 – 7.74 (m, 2H), 7.72 – 7.65 (m, 2H), 7.23 – 7.06 (m, 5H), 5.22 – 5.05 (m, 1H), 3.62 – 3.39 (m, 2H), 2.61 – 2.52 (m, 1.5H), 2.46 – 2.42 (m, 0.5H), 2.31 – 2.26 (m, 1H), 1.98 – 1.90 (m, 0.5H), 1.84 – 1.76 (m, 0.5H), 1.55 – 1.07 (m, 7H); ^{13}C NMR (125 MHz, $CDCl_3$, mixture of diastereomers): δ = 206.3, 206.2, 168.0, 167.8, 137.2, 137.1, 134.2, 134.1, 131.6, 131.5, 128.8, 128.8, 128.6, 128.5, 126.8, 126.7, 123.5, 123.4, 59.5, 50.5, 50.2, 40.7, 40.0, 36.5, 36.1, 36.0, 36.0, 34.0, 33.8, 33.6, 33.2, 29.9, 29.5, 28.6, 28.6; IR (cm^{-1}) 2949, 2866, 1777, 1707, 1454, 1383, 1098, 877, 749, 717, 700, 529; HRMS-EI (m/z) $[M]^+$ calcd for $C_{24}H_{23}NO_3$. 373.1678; found: 373.1676.

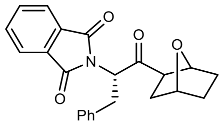

(*S*)-2-(1-(7-Oxabicyclo[2.2.1]heptan-2-yl)-1-oxo-3-phenylpropan-2-yl)isoindoline-1,3-dione (10a)

Yellow oil, 50.1 mg (0.134 mmol, 67% yield); ^{1}H NMR (500 MHz, $CDCl_3$, 1:1 mixture of diastereomers): δ = 7.80 – 7.68 (m, 4H), 7.21 – 7.06 (m, 5H), 5.18 – 5.08 (m, 1H), 4.95 – 4.90 (m, 0.5H), 4.73 – 4.68 (m, 0.5H), 4.67 – 4.56 (m, 1H), 3.61 – 3.34 (m, 2H), 2.85 – 2.70 (m, 1H), 2.31 – 2.19 (m, 0.5H), 1.99 – 1.89 (m, 0.5H), 1.74 – 1.66 (m, 2H), 1.62 (dd, *J* = 12.0, 9.4 Hz, 0.5H), 1.54 (dd, *J* = 12.0, 8.8 Hz, 0.5H), 1.42 – 1.30 (m, 2H); ^{13}C NMR (125 MHz, $CDCl_3$, mixture of diastereomers): δ = 203.9, 203.3, 167.7, 167.7, 136.9, 136.8, 134.4, 134.3, 131.4, 131.3, 128.9, 128.9, 128.6, 128.6, 126.9, 126.8, 123.6, 123.6, 77.9, 77.2, 76.4, 76.3, 59.1, 58.7, 51.8, 34.7, 33.9, 33.8, 33.3, 30.2, 29.5, 29.3; IR (cm^{-1}) 2952, 1774, 1707, 1496, 1467, 1381, 1099, 873, 717, 700, 529; HRMS-EI (m/z) $[M]^+$ calcd for $C_{23}H_{21}NO_4$, 375.1473; found: 375.1471.

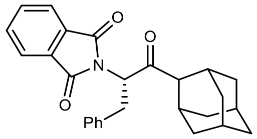

(*S*)-2-(1-(Adaman-2-yl)-1-oxo-3-phenylpropan-2-yl)isoindoline-1,3-dione (11a)

White solid, 26.4 mg (0.064 mmol, 32% yield); m.p. 101–103 °C; ^{1}H NMR (500 MHz, $CDCl_3$): δ = 7.80 – 7.73 (m, 2H), 7.73 – 7.64 (m, 2H), 7.21 – 7.10 (m, 5H), 5.21 (dd, *J* = 10.8, 5.5 Hz, 1H), 3.59 – 3.46 (m, 2H), 2.80 (s, 1H), 2.42 (d, *J* = 4.0 Hz, 1H), 2.26 (s, 1H), 1.99 – 1.57 (m, 12H); ^{13}C NMR (125 MHz, $CDCl_3$): δ = 205.9, 168.1, 137.1, 134.1, 131.6, 128.8, 128.6, 126.8, 123.4, 58.6, 53.2, 38.5, 38.4, 37.2, 34.1, 33.1, 32.8, 29.8, 29.0, 27.6, 27.3; IR (cm^{-1}) 2896, 2851, 1773, 1710, 1453, 1383, 1361, 1099, 744, 735, 715. 700, 529; HRMS-EI (m/z) $[M]^+$ calcd for $C_{27}H_{27}NO_3$, 413.1993; found: 413.1991.

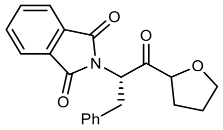

2-((2*S*)-1-Oxo-3-phenyl-1-(tetrahydrofuran-2-yl)propan-2-yl)isoindoline-1,3-dione (12a)

White viscous solid, 63.9 mg (0.183 mmol, 92% yield); ^{1}H NMR (500 MHz, $CDCl_3$, 1:1 mixture of diastereomers): δ = 7.80 – 7.74 (m, 2H), 7.70 – 7.64 (m, 2H), 7.22 – 7.06 (m, 5H), 5.45 (dd, *J* = 11.7, 4.3 Hz, 0.5H), 5.22 (dd, *J* = 10.6, 5.1 Hz, 0.5H), 4.63 (dd, *J* = 7.6, 6.6 Hz, 0.5H), 4.40 (dd, *J* = 8.2, 5.6 Hz, 0.5H), 3.99 – 3.88 (m, 1H), 3.87 – 3.79 (m, 0.5H), 3.78 – 3.72 (m, 1H), 3.64 – 3.48 (m, 1H), 3.30 (dd, *J* = 14.2, 10.6 Hz, 0.5H), 2.23 – 1.78 (m, 4H); ^{13}C NMR (125 MHz, $CDCl_3$, mixture of diastereomers): δ = 205.9, 205.5, 168.0, 167.6, 137.2, 137.0, 134.1, 134.0, 131.7, 131.6, 129.1, 128.9, 128.8, 128.6, 128.5, 128.5, 126.7, 126.7, 123.5, 123.4, 82.1, 81.8, 69.5, 69.5, 59.0, 57.4, 33.8, 33.1, 28.6, 28.5, 25.5; IR (cm^{-1}) 3262, 3029, 1773, 1707, 1386, 1102, 718, 699, 530; HRMS-EI (m/z) $[M]^+$ calcd for $C_{21}H_{19}NO_4$, 349.1315; found: 349.1314.

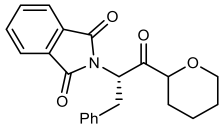

2-((2*S*)-1-Oxo-3-phenyl-1-(tetrahydro-2H-pyran-2-yl)propan-2-yl)isoindoline-1,3-dione (13a)

White oil, 59.4 mg (0.164 mmol, 82% yield); ^{1}H NMR (500 MHz, $CDCl_3$, 2:1 mixture of diastereomers): δ = 7.78 – 7.74 (m, 2H), 7.72 – 7.62 (m, 2H), 7.22 – 7.08 (m, 5H), 5.53 (dd, *J* = 11.5, 4.4 Hz, 0.7H), 5.40 (dd, *J* = 11.1, 4.6 Hz, 0.3H), 4.10 – 4.03 (m, 1.6H), 3.94 – 3.87 (m, 0.7H), 3.70 – 3.49 (m, 2H), 3.40 (dd, *J* = 14.2, 11.1 Hz, 0.3H), 3.32 – 3.26 (m, 0.4H), 1.99 – 1.83 (m, 2H), 1.62 – 1.42 (m, 4H); ^{13}C NMR (125 MHz, $CDCl_3$, mixture of diastereomers): δ = 204.3, 203.7, 168.1, 167.8, 137.3, 137.2, 134.0, 134.0, 131.7, 131.7, 129.0, 128.8, 128.5, 128.5, 126.7, 126.6, 123.4, 123.3, 81.4, 80.7, 68.8, 68.7, 58.6, 57.8, 33.5, 33.1, 27.9, 27.5, 25.5, 25.5, 22.7; IR (cm^{-1}) 3029, 2941, 1774, 1707, 1385, 1088, 718, 699, 530; HRMS-EI (m/z) $[M]^+$ calcd for $C_{22}H_{21}NO_4$, 363.1467; found: 363.1471.

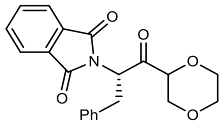

2-((2*S*)-1-(1,4-Dioxan-2-yl)-1-oxo-3-phenylpropan-2-yl)isoindoline-1,3-dione (14a)

Yellow solid, 60.2 mg (0.165 mmol, 82% yield); m.p. 47–49 °C; ^{1}H NMR (400 MHz, $CDCl_3$, 1:1 mixture of diastereomers): δ = 7.82 – 7.73 (m, 2H), 7.71 – 7.65 (m, 2H), 7.21 – 7.10 (m, 5H), 5.50 (dd, *J* = 11.3, 4.6 Hz, 0.5H), 5.31 (dd, *J* = 10.7, 4.9 Hz, 0.5H), 4.37 (dd, *J* = 9.5, 3.3 Hz, 0.5H), 4.16 (dd, *J* = 9.2, 3.3 Hz, 0.5H), 4.08 – 3.97 (m, 1H), 3.93 – 3.86 (m, 0.5H), 3.81 (td, *J* = 11.7, 11.2, 2.7 Hz, 0.5H), 3.75 – 3.49 (m, 5.5H), 3.32 (dd, *J* = 14.2, 10.7 Hz, 0.5H); ^{13}C NMR (100 MHz, $CDCl_3$, mixture of diastereomers): δ = 202.1, 201.4, 167.9, 167.6, 136.8, 136.8, 134.2, 134.1, 131.6, 131.5, 129.0, 128.8, 128.6, 128.5, 126.9, 126.8, 123.5, 123.4, 78.4, 78.1, 67.3, 67.2, 66.9, 66.8, 66.3, 66.2, 58.7, 57.5, 33.4, 33.1; IR (cm^{-1}) 2917, 2856, 1775, 1708, 1382, 1101, 902, 876, 717, 700, 529; HRMS-EI (m/z) [M]$^+$ calcd for $C_{21}H_{19}NO_5$, 365.1260; found: 365.1263.

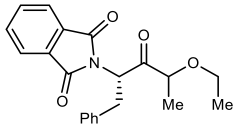

2-((2*S*)-4-Ethoxy-3-oxo-1-phenylpentan-2-yl)isoindoline-1,3-dione (15a)

White oil, 62.6 mg (0.178 mmol, 89% yield); ^{1}H NMR (500 MHz, $CDCl_3$, 2:1 mixture of diastereomers): δ = 7.80 – 7.73 (m, 2H), 7.72 – 7.65 (m, 2H), 7.22 – 7.08 (m, 5H), 5.44 – 5.35 (m, 1H), 4.14 (q, *J* = 6.7 Hz, 0.7H), 4.01 (q, *J* = 6.7 Hz, 0.3H), 3.65 – 3.52 (m, 2.7H), 3.48 – 3.39 (m, 1H), 3.37 – 3.26 (m, 0.3H), 1.39 (d, *J* = 6.7 Hz, 1H), 1.32 (d, *J* = 6.8 Hz, 2H), 1.23 (t, *J* = 7.0 Hz, 2H), 1.13 (t, *J* = 6.9 Hz, H); ^{13}C NMR (125 MHz, $CDCl_3$, mixture of diastereomers): δ = 205.6, 205.3, 167.8, 167.7, 137.2, 137.0, 134.1, 131.6, 128.9, 128.5, 128.5, 126.8, 126.7, 123.4, 123.4, 79.4, 79.0, 65.6, 65.4, 58.1, 58.0, 33.5, 33.4, 16.6, 15.3, 15.2; IR (cm^{-1}) 3030, 1774, 1707, 1384, 1104, 874, 717, 698, 530; HRMS-EI (m/z) [M]$^+$ calcd for $C_{21}H_{21}NO_4$, 351.1473; found: 351.1471.

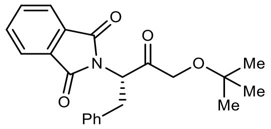

(*S*)-2-(4-(*tert*-butoxy)-3-oxo-1-phenylbutan-2-yl)isoindoline-1,3-dione (16a)

White viscous solid, 57.2 mg (0.157 mmol, 78% yield); ^{1}H NMR (400 MHz, $CDCl_3$): δ = 7.72 – 7.67 (m, 2H), 7.65 – 7.58 (m, 2H), 7.13 – 7.02 (m, 5H), 5.25 (dd, *J* = 11.0, 4.7 Hz, 1H), 4.11 – 3.91 (m, 2H), 3.62 – 3.24 (m, 2H), 1.01 (s, 9H); ^{13}C NMR (125 MHz, $CDCl_3$): δ = 203.9, 167.7, 137.1, 134.1, 131.7, 129.0, 128.5, 126.7, 123.4, 74.3, 67.5, 58.3, 33.5, 27.0; IR (cm^{-1}) 3262, 1772, 1741, 1694, 1386, 1217, 1101, 942, 719, 701, 633, 530; HRMS-EI (m/z) [M]$^+$ calcd for $C_{22}H_{23}NO_4$, 365.1630; found: 365.1627.

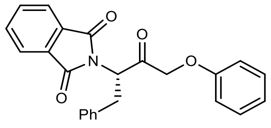

(*S*)-2-(3-Oxo-4-phenoxy-1-phenylbutan-2-yl)isoindoline-1,3-dione (17a)

Brown solid, 68.9 mg (0.179 mmol, 89% yield); m.p. 104–106 °C; ^{1}H NMR (500 MHz, $CDCl_3$): δ = 7.81 – 7.73 (m, 2H), 7.73 – 7.64 (m, 2H), 7.29 – 7.21 (m, 2H), 7.21 – 7.09 (m, 5H), 6.99 – 6.92 (m, 1H), 6.85 – 6.79 (m, 2H), 5.35 (dd, *J* = 10.8, 5.0 Hz, 1H), 4.79 – 4.63 (m, 2H), 3.67 – 3.60 (m, 1H), 3.47 – 3.38 (m, 1H); ^{13}C NMR (125 MHz, $CDCl_3$): δ = 201.2, 167.6, 157.4, 136.5, 134.2, 131.5, 129.7, 129.0, 128.6, 126.9, 123.5, 121.9, 114.4, 71.6, 58.2, 33.5; IR (cm^{-1}) 2924, 1775, 1729, 1708, 1598, 1586, 1495, 1380, 1228, 1083, 831, 754, 717, 527; HRMS-EI (m/z) [M]$^+$ calcd for $C_{24}H_{19}NO_4$, 385.1312; found: 385.1314.

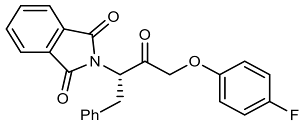

(*S*)-2-(4-(4-Fluorophenoxy)-3-oxo-1-phenylbutan-2-yl)isoindoline-1,3-dione (18a)

Brown solid, 48.2 mg (0.120 mmol, 60% yield); m.p. 150–152 °C; ^{1}H NMR (500 MHz, $CDCl_3$): δ = 7.82 – 7.74 (m, 2H), 7.73 – 7.66 (m, 2H), 7.21 – 7.10 (m, 5H), 6.98 – 6.85 (m, 2H), 6.80 – 6.70 (m, 2H), 5.30 (dd, *J* = 10.8, 5.0 Hz, 1H), 4.75 – 4.60 (m, 2H), 3.65 – 3.34 (m, 2H) ; ^{13}C NMR (125 MHz, $CDCl_3$): δ = 200.9, 167.6, 157.8 (d, *J* = 239.9 Hz), 153.6, 153.6, 136.4, 134.3, 131.4, 129.0, 128.6, 127.0, 123.6, 116.1 (d, *J* = 23.2 Hz), 115.6 (d, *J* = 8.2 Hz), 72.2, 58.1, 33.6; ^{19}F NMR (470 MHz, $CDCl_3$): δ = –122.35; IR (cm^{-1}) 2905, 2035, 1708, 1747, 1711, 1600, 1505, 1381, 1201, 1099, 964, 877, 821, 749, 718, 529; HRMS-EI (m/z) [M]$^+$ calcd for $C_{24}H_{18}FNO_4$, 403.1217; found: 403.1220.

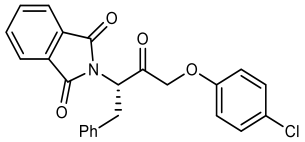

(*S*)-2-(4-(4-Chlorophenoxy)-3-oxo-1-phenylbutan-2-yl)isoindoline-1,3-dione (19a)

Orange solid, 40.5 mg (0.097 mmol, 48% yield); m.p. 179–181 °C; ^{1}H NMR (400 MHz, $CDCl_3$): δ = 7.81 – 7.73 (m, 2H), 7.73 – 7.63 (m, 2H), 7.22 – 7.10 (m, 7H), 6.78 – 6.70 (m, 2H), 5.29 (dd, *J* = 10.7, 5.1 Hz, 1H), 4.74 – 4.62 (m, 2H), 3.65 – 3.29 (m, 2H); ^{13}C NMR (100 MHz, $CDCl_3$): δ = 200.7, 167.5, 156.0, 136.3, 134.3, 131.4, 129.6, 129.0, 128.6, 127.0, 126.9, 123.6, 115.7, 71.8, 58.0, 33.6; IR (cm^{-1}) 3063, 2916, 1778, 1746, 1706, 1591, 1491, 1379, 1241, 1060, 876, 818, 725, 529; HRMS-EI (m/z) [M]$^+$ calcd for $C_{24}H_{18}ClNO_4$, 419.0928; found: 419.0924.

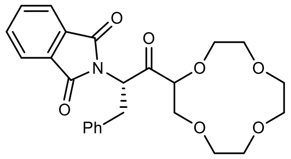

2-((2*S*)-1-(1,4,7,10-Tetraoxacyclododecan-2-yl)-1-oxo-3-phenylpropan-2-yl)isoindoline-1,3-dione (20a)

White oil, 61.4 mg (0.135 mmol, 68% yield); ^{1}H NMR (500 MHz, $CDCl_3$, 2:1 mixture of diastereomers): δ = 7.77 – 7.70 (m, 2H), 7.69 – 7.58 (m, 2H), 7.18 – 7.04 (m, 5H), 5.40 (dd, *J* = 11.2, 4.5 Hz, 0.3H), 5.23 (dd, *J* = 10.7, 5.1 Hz, 0.7H), 4.50 (dd, *J* = 7.3, 2.7 Hz, 0.7H), 4.27 (dd, *J* = 6.8, 2.9 Hz, 0.3H), 4.01 – 3.77 (m, 2H), 3.70 – 3.30 (m, 14H); ^{13}C NMR (125 MHz, $CDCl_3$, mixture of diastereomers): δ = 204.7, 204.1, 167.7, 167.5, 137.1, 136.9, 134.1, 134.1, 131.7, 131.6, 129.0, 129.0, 128.5, 128.5, 126.7, 126.7, 123.4, 123.4, 84.3, 83.5, 71.9, 71.4, 71.1, 71.0, 70.9, 70.7, 70.6, 70.5, 70.3, 70.2, 70.1, 58.6, 57.8, 33.4, 33.0; IR (cm^{-1}) 2913, 2865, 1774, 1709, 1455, 1383, 1124, 719, 701, 530; HRMS-EI (m/z) [M]$^+$ calcd for $C_{25}H_{27}NO_7$, 453.1785; found: 453.1788.

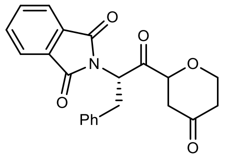

2-((2*S*)-1-Oxo-1-(4-oxotetrahydro-2H-pyran-2-yl)-3-phenylpropan-2-yl)isoindoline-1,3-dione (21a)

White solid, 50.4 mg (0.134 mmol, 67% yield); m.p. 142–144 °C; ^{1}H NMR (500 MHz, $CDCl_3$, 2:1 mixture of diastereomers): δ = 7.81 – 7.74 (m, 2H), 7.72 – 7.66 (m, 2H), 7.23 – 7.06 (m, 5H), 5.58 (dd, *J* = 11.0, 5.0 Hz, 0.7H), 5.38 (dd, *J* = 10.5, 5.1 Hz, 0.3H), 4.40 (dd, *J* = 11.1, 3.6 Hz, 0.7H), 4.35 (ddd, *J* = 11.6, 7.1, 2.1 Hz, 0.7H), 4.22 (dd, *J* = 10.6, 3.8 Hz, 0.3H), 4.16 (ddd, *J* = 11.6, 7.0, 2.4 Hz, 0.3H), 3.76 (td, *J* = 11.7, 3.1 Hz, 0.7H), 3.69 – 3.47 (m, 2H), 3.37 – 3.27 (m, 0.3H), 2.74 – 2.64 (m, 1H), 2.64 – 2.51 (m, 2H), 2.43 – 2.35 (m, 0.7H), 2.33 – 2.25 (m, 0.3H); ^{13}C NMR (125 MHz, $CDCl_3$, mixture of diastereomers): δ = 204.3, 204.2, 201.5, 201.0, 167.9, 167.5, 136.6, 134.3, 134.2, 131.6, 131.4, 129.1, 128.8, 128.6, 128.5, 127.0, 126.9, 123.6, 123.5, 79.6, 79.4, 67.0, 66.9, 58.5, 57.3, 43.0, 43.0, 41.8, 41.8, 33.7, 33.4; IR (cm^{-1}) 2925, 2863, 1773, 1725, 1705, 1498, 1455, 1382, 1099, 871, 717, 696, 528; HRMS-EI (m/z) [M]$^+$ calcd for $C_{22}H_{19}NO_5$, 377.1261; found: 377.1263.

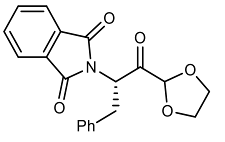

21a major

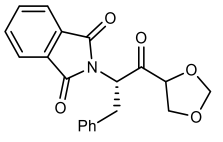

21a' minor

2-((2*S*)-1-(1,3-Dioxolan-2-yl)-1-oxo-3-phenylpropan-2-yl)isoindoline-1,3-dione (22a)

Yellow oil, 44.6 mg (0.127 mmol, 64% yield); ^{1}H NMR (500 MHz, $CDCl_3$): δ = 7.81 – 7.76 (m, 2H), 7.73 – 7.63 (m, 2H), 7.19 – 7.11 (m, 5H), 5.34 – 5.27 (m, 2H), 4.04 – 3.86 (m, 4H), 3.62 – 3.34 (m, 2H); ^{13}C NMR (125 MHz, $CDCl_3$): δ = 199.4, 167.5, 136.5, 134.2, 131.5, 129.0, 128.5, 126.9, 123.5, 100.9, 65.7, 65.5, 56.9, 33.5; IR (cm^{-1}) 2190, 1803, 1773, 1707, 1386, 1172, 1073, 718, 700, 530; HRMS-EI (m/z) [M]$^+$ calcd for $C_{20}H_{17}NO_5$, 351.1107; found: 351.1107.

2-((2*S*)-1-(1,3-Dioxolan-4-yl)-1-oxo-3-phenylpropan-2-yl)isoindoline-1,3-dione (22a')

Yellow oil, 10.9 mg (0.031 mmol, 16% yield); ^{1}H NMR (500 MHz, $CDCl_3$, 1:1 mixture of diastereomers): δ = 7.84 – 7.75 (m, 2H), 7.72 – 7.63 (m, 2H), 7.21 – 7.09 (m, 5H), 5.43 (dd, *J* = 11.3, 4.7 Hz, 0.5H), 5.20 (dd, *J* = 10.4, 5.1 Hz, 0.5H), 5.03 (s, 0.5H), 4.97 (s, 0.5H), 4.87 (s, 0.5H), 4.79 (s, 0.5H), 4.67 (dd, *J* = 8.1, 4.4 Hz, 0.5H), 4.45 (dd, *J* = 7.6, 4.1 Hz, 0.5H), 4.27 (dd, *J* = 8.7, 4.1 Hz, 0.5H), 4.14 (dd, *J* = 8.8, 4.4 Hz, 0.5H), 4.04 – 3.91 (m, 1H), 3.71 – 3.63 (m, 0.5H), 3.54 – 3.45 (m, 1H), 3.28 – 3.19 (m, 0.5H); ^{13}C NMR (125 MHz, $CDCl_3$, mixture of diastereomers): δ = 204.4, 203.6, 167.7, 167.5, 136.7, 136.6, 134.2, 134.1, 131.5, 129.1, 128.9, 128.6, 128.5, 126.9, 126.8, 123.6, 123.4, 96.4, 96.2, 78.4, 78.3, 67.6, 67.3, 58.2, 57.4, 33.8, 33.1; IR (cm^{-1}) 1775, 1707, 1383, 1086, 926, 717, 700, 529; HRMS-EI (m/z) [M]$^+$ calcd for $C_{20}H_{17}NO_5$, 351.1110; found: 351.1107.

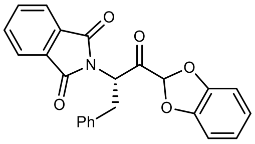

(*S*)-2-(1-(Benzo[d][1,3]dioxol-2-yl)-1-oxo-3-phenylpropan-2-yl)isoindoline-1,3-dione (23a)

White viscous solid, 53.9 mg (0.135 mmol, 68% yield); ^{1}H NMR (500 MHz, $CDCl_3$): δ = 7.73 – 7.61 (m, 4H), 7.19 – 7.07 (m, 5H), 6.81 – 6.76 (m, 1H), 6.73 – 6.67 (m, 1H), 6.65 – 6.57 (m, 2H), 6.27 (s, 1H), 5.37 (dd, *J* = 10.6, 5.0 Hz, 1H), 3.68 – 3.26 (m, 2H); ^{13}C NMR (125 MHz, $CDCl_3$): δ = 197.0, 167.0, 146.2, 145.8, 136.1, 134.1, 131.3, 129.1, 128.6, 127.0, 123.4, 122.4, 122.2, 109.3, 109.0, 105.9, 56.3, 33.6; IR (cm^{-1}) 3064, 2918, 2850, 1774, 1711, 1604, 1481, 1382, 1234, 1095, 716, 699, 530; HRMS-EI (m/z) [M]$^+$ calcd for $C_{24}H_{17}NO_5$, 399.1106 found: 399.1107.

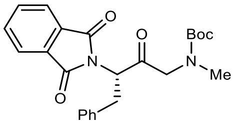

***tert*-Butyl (*S*)-(3-(1,3-dioxoisoindolin-2-yl)-2-oxo-4-phenylbutyl)(methyl)carbamate (24a)**

White solid, 67.0 mg (0.158 mmol, 79% yield); m.p. 105–107 °C; ^{1}H NMR (500 MHz, $CDCl_3$, 1:1 mixture of rotamers): δ = 7.82 – 7.65 (m, 4H), 7.19 – 7.06 (m, 5H), 5.16 – 4.96 (m, 1H), 4.32 – 4.25 (m, 0.5H), 4.13 – 3.91 (m, 1.5H), 3.60 – 3.51 (m, 1H), 3.42 – 3.27 (m, 1H), 2.89 – 2.83 (m, 3H), 1.45 – 1.37 (m, 9H); ^{13}C NMR (125 MHz, $CDCl_3$, mixture of rotamers): δ = 201.4, 200.4, 167.5, 156.0, 155.4, 136.5, 136.4, 134.4, 134.2, 131.5, 131.4, 129.0, 128.9, 128.6, 128.5, 127.0, 126.8, 123.6, 123.5, 80.5, 80.3, 58.7, 58.3, 56.4, 55.9, 35.8, 35.6, 33.6, 28.3, 28.2; IR (cm^{-1}) 2977, 2931, 2886, 1777, 1742, 1712, 1692, 1384, 1246, 1171, 1089, 986, 874, 719, 528; HRMS-FAB (m/z) [M]$^+$ calcd for $C_{24}H_{26}N_2O_5$, 422.1839 found: 422.1842.

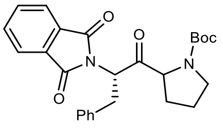

***tert*-Butyl 2-((*S*)-2-(1,3-dioxoisoindolin-2-yl)-3-phenylpropanoyl)pyrrolidine-1-carboxylate (25a)**

White oil, 79.8 mg (0.178 mmol, 89% yield); ^{1}H NMR (500 MHz, $CDCl_3$, mixture of diastereomers and rotamers): δ = 7.80 – 7.55 (m, 4H), 7.19 – 6.95 (m, 5H), 5.48 – 5.13 (m, 1H), 5.00 – 4.39 (m, 1H), 3.62 – 3.21 (m, 4H), 2.51 – 1.70 (m, 4H), 1.61 – 1.22 (m, 9H); ^{13}C NMR (125 MHz, $CDCl_3$, mixture of diastereomers and rotamers): δ = 205.0, 204.0, 200.0, 198.6, 167.7, 167.5, 167.3, 154.5, 153.5, 137.0, 136.7, 136.4, 136.2, 134.3, 134.3, 134.2, 134.1, 131.5, 131.4, 131.2, 129.0, 129.0,

128.8, 128.8, 128.7, 128.6, 128.6, 128.4, 128.4, 126.9, 126.9, 126.7, 126.6, 123.6, 123.5, 123.4, 123.3, 80.7, 79.9, 62.4, 61.9, 61.8, 59.3, 59.1, 58.0, 56.9, 46.9, 46.8, 33.9, 33.5, 33.1, 33.0, 31.6, 30.5, 29.6, 29.3, 28.4, 28.4, 24.7, 23.8, 22.7; IR (cm^{-1}) 2976, 1775, 1710, 1380, 1158, 717, 700, 530; HRMS-EI (m/z) $[M]^+$ calcd for $C_{24}H_{28}N_2O_5$, 449.2073 found: 449.2076.

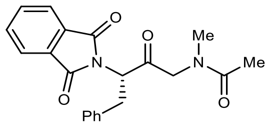

(*S*)-*N*-(3-(1,3-Dioxoisoindolin-2-yl)-2-oxo-4-phenylbutyl)-*N*-methylacetamide (26a)

White solid, 59.7 mg (0.164 mmol, 82% yield); m.p. 138–140 °C; ^1H NMR (500 MHz, $CDCl_3$, 5:1 mixture of rotamers): δ = 7.83 – 7.65 (m, 4H), 7.21 – 7.08 (m, 5H), 5.11 (dd, *J* = 11.1, 5.0 Hz, 0.8H), 5.06 (dd, *J* = 10.8, 5.3 Hz, 0.2H), 4.49 (d, *J* = 17.4 Hz, 0.8H), 4.32 (d, *J* = 19.1 Hz, 0.2H), 4.20 – 4.13 (m, 0.2H), 4.06 (d, *J* = 17.4 Hz, 0.8H), 3.65 – 3.32 (m, 2H), 3.04 (s, 2.5H), 2.90 (s, 0.5H), 2.11 (s, 2.5H), 1.96 (s, 0.5H); ^{13}C NMR (125 MHz, $CDCl_3$, mixture of rotamers): δ = 200.5, 199.9, 171.4, 171.3, 167.6, 167.5, 136.4, 135.9, 134.7, 134.3, 131.4, 131.2, 129.0, 128.9, 128.7, 128.6, 127.1, 126.9, 123.8, 123.5, 58.9, 58.5, 57.8, 54.8, 37.4, 34.9, 33.9, 33.6, 21.1, 21.1; IR (cm^{-1}) 2916, 1773, 1736, 1710, 1631, 1468, 1383, 1218, 989, 877, 718, 702, 529, 484; HRMS-EI (m/z) $[M]^+$ calcd for $C_{21}H_{20}N_2O_4$, 364.1421 found: 364.1423.

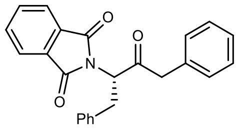

(*S*)-2-(3-Oxo-1,4-diphenylbutan-2-yl)isoindoline-1,3-dione (27a)

White solid, 45.7 mg (0.124 mmol, 62% yield); m.p. 113–115 °C; ^1H NMR (400 MHz, $CDCl_3$): δ = 7.78 – 7.73 (m, 2H), 7.72 – 7.67 (m, 2H), 7.30 – 7.20 (m, 3H), 7.17 – 7.04 (m, 7H), 5.07 (dd, *J* = 11.0, 5.0 Hz, 1H), 3.79 (s, 2H), 3.64 – 3.33 (m, 2H); ^{13}C NMR (125 MHz, $CDCl_3$): δ = 202.3, 167.6, 136.8, 134.2, 133.1, 131.5, 129.4, 128.9, 128.9, 128.7, 128.6, 128.5, 127.2, 126.8, 123.5, 59.6, 46.6, 33.7; IR (cm^{-1}) 3062, 1775, 1731, 1707, 1604, 1495, 1383, 1097, 1063, 981, 719, 707, 530, 515; HRMS-EI (m/z) $[M]^+$ calcd for $C_{24}H_{19}NO_3$, 369.1362 found: 369.1365.

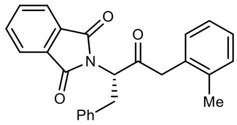

(*S*)-2-(3-Oxo-1-phenyl-4-(*o*-tolyl)butan-2-yl)isoindoline-1,3-dione (28a)

White solid, 39.9 mg (0.104 mmol, 52% yield); m.p. 82–84 °C; ^1H NMR (500 MHz, $CDCl_3$): δ = 7.81 – 7.74 (m, 2H), 7.74 – 7.66 (m, 2H), 7.19 – 6.99 (m, 9H), 5.07 (dd, *J* = 11.1, 5.0 Hz, 1H), 3.82 (s, 2H), 3.64 – 3.40 (m, 2H), 2.21 (s, 3H); ^{13}C NMR (125 MHz, $CDCl_3$): δ = 202.2, 167.7, 137.0, 136.8, 134.2, 132.0, 131.5, 130.5, 130.4, 128.9, 128.6, 127.5, 126.8, 126.2, 123.5, 60.0, 45.0, 33.8, 19.6; IR (cm^{-1}) 3026, 2922, 1769, 1732, 1709, 1605, 1465, 1380, 1098, 1063, 974, 874, 744, 722, 700, 529, 510; HRMS-EI (m/z) $[M]^+$ calcd for $C_{25}H_{21}NO_3$, 383.1524 found: 383.1521.

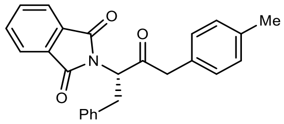

(*S*)-2-(3-Oxo-1-phenyl-4-(*p*-tolyl)butan-2-yl)isoindoline-1,3-dione (29a)

White solid, 53.6 mg (0.140 mmol, 70% yield); m.p. 135–137 °C; ^1H NMR (500 MHz, $CDCl_3$): δ = 7.80 – 7.73 (m, 2H), 7.73 – 7.63 (m, 2H), 7.16 – 6.99 (m, 9H), 5.07 (dd, *J* = 11.1, 5.0 Hz, 1H), 3.76 (s, 2H), 3.62 – 3.32 (m, 2H), 2.29 (s, 3H); ^{13}C NMR (125 MHz, $CDCl_3$): δ = 202.5, 167.6, 136.9, 134.2, 131.5, 130.0, 129.4, 129.2, 128.9, 128.5, 126.8, 123.5, 59.5, 46.3, 33.7, 21.1; IR (cm^{-1}) 2906, 1775, 1710, 1380, 1337, 1095, 1065, 977, 796, 719, 104, 530, 481; HRMS-EI (m/z) $[M]^+$ calcd for $C_{25}H_{21}NO_3$, 383.1522 found: 383.1521.

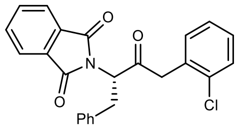

(*S*)-2-(4-(2-Chlorophenyl)-3-oxo-1-phenylbutan-2-yl)isoindoline-1,3-dione (30a)

White solid, 27.5 mg (0.068 mmol, 34% yield); m.p. 83–85 °C; ^{1}H NMR (500 MHz, $CDCl_3$): δ = 7.80 – 7.74 (m, 2H), 7.72 – 7.66 (m, 2H), 7.36 – 7.31 (m, 1H), 7.22 – 7.07 (m, 8H), 5.13 (dd, *J* = 11.1, 5.0 Hz, 1H), 4.05 – 3.83 (m, 2H), 3.67 – 3.35 (m, 2H); ^{13}C NMR (125 MHz, $CDCl_3$): δ = 201.3, 167.6, 136.7, 134.3, 134.3, 134.1, 131.9, 131.7, 131.5, 129.5, 128.9, 128.8, 128.5, 127.0, 126.8, 123.5, 60.0, 44.3, 33.8; IR (cm^{-1}) 3062, 2923, 1770, 1711, 1603, 1379, 1340, 1099, 974, 875, 721, 702, 529, 518; HRMS-EI (m/z) [M]$^+$ calcd for $C_{24}H_{18}ClNO_3$, 403.0974 found: 403.0975.

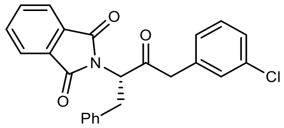

(*S*)-2-(4-(3-Chlorophenyl)-3-oxo-1-phenylbutan-2-yl)isoindoline-1,3-dione (31a)

White solid, 35.0 mg (0.087 mmol, 43% yield); m.p. 143–145 °C; ^{1}H NMR (500 MHz, $CDCl_3$): δ = 7.80 – 7.74 (m, 2H), 7.74 – 7.68 (m, 2H), 7.22 – 7.06 (m, 8H), 7.03 – 6.98 (m, 1H), 5.12 – 5.04 (m, 1H), 3.76 (d, *J* = 2.9 Hz, 2H), 3.63 – 3.34 (m, 2H); ^{13}C NMR (125 MHz, $CDCl_3$): δ = 201.6, 167.5, 136.6, 134.9, 134.4, 134.4, 131.4, 129.9, 129.6, 128.9, 128.6, 127.6, 127.4, 126.9, 123.6, 59.7, 46.0, 33.8; IR (cm^{-1}) 2921, 2852, 1770, 1706, 1600, 1385, 1343, 1091, 977, 872, 786, 722, 701, 527, 512; HRMS-EI (m/z) [M]$^+$ calcd for $C_{24}H_{18}ClNO_3$, 403.0971 found: 403.0975.

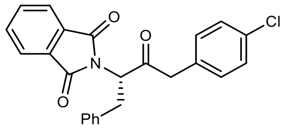

(*S*)-2-(4-(4-Chlorophenyl)-3-oxo-1-phenylbutan-2-yl)isoindoline-1,3-dione (32a)

White solid, 58.7 mg (0.146 mmol, 73% yield); m.p. 152–154 °C; ^{1}H NMR (500 MHz, $CDCl_3$): δ = 7.81 – 7.74 (m, 2H), 7.73 – 7.69 (m, 2H), 7.24 – 7.20 (m, 2H), 7.17 – 7.08 (m, 5H), 7.06 – 7.03 (m, 2H), 5.06 (dd, *J* = 10.9, 5.1 Hz, 1H), 3.75 (s, 2H), 3.60 – 3.33 (m, 2H); ^{13}C NMR (125 MHz, $CDCl_3$): δ = 201.8, 167.6, 136.6, 134.4, 133.2, 131.5, 131.4, 130.8, 128.9, 128.8, 128.6, 126.9, 123.6, 59.7, 45.7, 33.8; IR (cm^{-1}) 1775, 1730, 1707, 1492, 1384, 1089, 983, 877, 746, 720, 529, 496; HRMS-EI (m/z) [M]$^+$ calcd for $C_{24}H_{18}ClNO_3$, 403.0978 found: 403.0975.

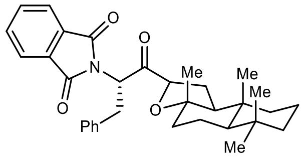

2-((*S*)-1-Oxo-3-phenyl-1-((2*R*,3a*S*,5a*R*,9a*R*,9b*S*)-3a,6,6,9a-tetramethyldodecahydronaphtho[2,1-b]furan-2-yl)propan-2-yl)isoindoline-1,3-dione (33a)

White oil, 62.8 mg (0.122 mmol, 61% yield); ^{1}H NMR (500 MHz, $CDCl_3$, 3:1 mixture of diastereomers): δ 7.84 – 7.74 (m, 2H), 7.73 – 7.64 (m, 2H), 7.22 – 7.06 (m, 5H), 5.46 (dd, *J* = 11.2, 4.6 Hz, 0.75H), 5.19 (dd, *J* = 10.5, 5.3 Hz, 0.25H), 4.65 (dd, *J* = 10.2, 3.2 Hz, 0.75H), 4.35 (t, *J* = 8.1 Hz, 0.25H), 3.68 – 3.19 (m, 2H), 2.08 – 1.73 (m, 3H), 1.69 – 0.67 (m, 22H), 0.45 (dd, *J* = 12.3, 2.7 Hz, 1H); ^{13}C NMR (125 MHz, $CDCl_3$, mixture of diastereomers): δ 207.9, 206.2, 167.7, 167.5, 137.3, 137.1, 134.1, 134.1, 133.9, 131.9, 131.8, 129.1, 129.0, 128.9, 128.5, 128.4, 128.4, 126.6, 126.6, 123.5, 123.4, 123.3, 82.8, 82.2, 80.5, 79.9, 60.2, 59.0, 58.3, 57.7, 57.0, 56.9, 42.4, 42.2, 39.9, 39.5, 39.3, 39.3, 36.3, 36.2, 34.2, 33.5, 33.4, 33.1, 33.0, 32.9, 27.3, 26.2, 22.4, 21.2, 21.0, 21.0, 20.6, 20.4, 18.3, 18.2, 15.2, 14.8; IR (cm^{-1}) 2929, 1773, 1713, 1455, 1386, 1104, 952, 717, 699; HRMS-EI (m/z) [M]$^+$ calcd for $C_{33}H_{39}NO_4$, 513.2878 found: 513.2879.

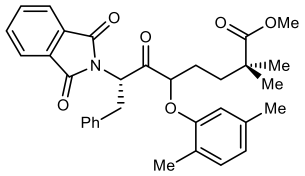

Methyl (7*S*)-5-(2,5-dimethylphenoxy)-7-(1,3-dioxoisoindolin-2-yl)-2,2-dimethyl-6-oxo-8-phenyloctanoate (34a)

White viscous solid, 76.0 mg (0.140 mmol, 70% yield); ^{1}H NMR (500 MHz, $CDCl_3$, 1:1 miture of diastereomers): δ = 7.82 – 7.75 (m, 1H), 7.74 – 7.67 (m, 1H), 7.59 – 7.53 (m, 2H), 7.18 – 7.01 (m, 5.5H), 6.78 (d, *J* = 7.5 Hz, 0.5H), 6.69 (d, *J* = 7.5 Hz, 0.5H), 6.48 (s, 0.5H), 6.36 (d, *J* = 7.5 Hz, 0.5H), 6.24 (s, 0.5H), 5.38 – 5.29 (m, 1H), 4.81 – 4.67 (m, 1H), 3.63 (s, 1.5H), 3.53 (s, 1.5H), 3.51 – 3.32 (m, 2H), 2.28 (s, 1.5H), 2.22 (s, 1.5H), 2.14 (s, 1.5H), 2.03 (s, 1.5H), 1.93 – 1.53 (m, 4H), 1.16 (s, 1.5H), 1.15 (s, 1.5H), 1.11 (s, 1.5H), 1.09 (s, 1.5H); ^{13}C NMR (125 MHz, $CDCl_3$, mixture of diastereomers): δ = 204.5, 203.9, 177.9, 177.8, 167.6, 167.2, 155.3, 155.0, 136.8, 136.7, 136.6, 136.5, 134.3, 133.9, 133.6, 131.5, 131.3, 130.9, 129.1, 128.9, 128.9, 128.5, 126.8, 123.9, 123.6, 123.5, 123.2, 122.9, 122.0, 121.6, 112.5, 111.2, 81.1, 80.8, 57.6, 57.1, 51.8, 51.7, 42.0, 41.9, 35.8, 35.7, 33.8, 33.3, 28.1, 27.4, 25.1, 25.0, 24.9, 21.3, 21.1, 15.9, 15.7; IR (cm^{-1}) 2924, 1776, 1712, 1614, 1584, 1508, 1382, 1254, 1196, 1127, 718, 700, 530; HRMS-EI (m/z) [M]$^+$ calcd for $C_{33}H_{35}NO_6$, 541.2462 found: 541.2464.

1.5. Chiral HPLC Data

After acid chloride generation from the described procedure and quenching the reaction by the addition of an excess amount of methanol (1 mL), the amino acid methyl ester was synthesized. The enantiomeric excesses (ee's) of the starting materials (**2a**, **2k**, **2l**, **2n, 2o, 2s**) were determined via HPLC traces using the amino acid methyl ester, and the ee's of the products were determined after isolation of the targeted products (**3a**, **3k**, **3l**, **3n**, **3o**, **3s**, **16a**, **17a**, **26a**, **27a**) by silica column chromatography. The results are summarized in Supplementary Table 1, along with their HPLC chromatograms (Supplementary Fig. 1–32).

By comparing the ee of **2-OMe** and **3**, we concluded that the ee of the starting material is preserved when alkyl substrates and ethereal substrates were used, except for benzylic C–H substrates, which required 10 equiv of the substrates to facilitate the reaction.

Supplementary Table 1. Comparison of Enantiomeric Excesses of Amino Acid Methyl Esters and Target Products.

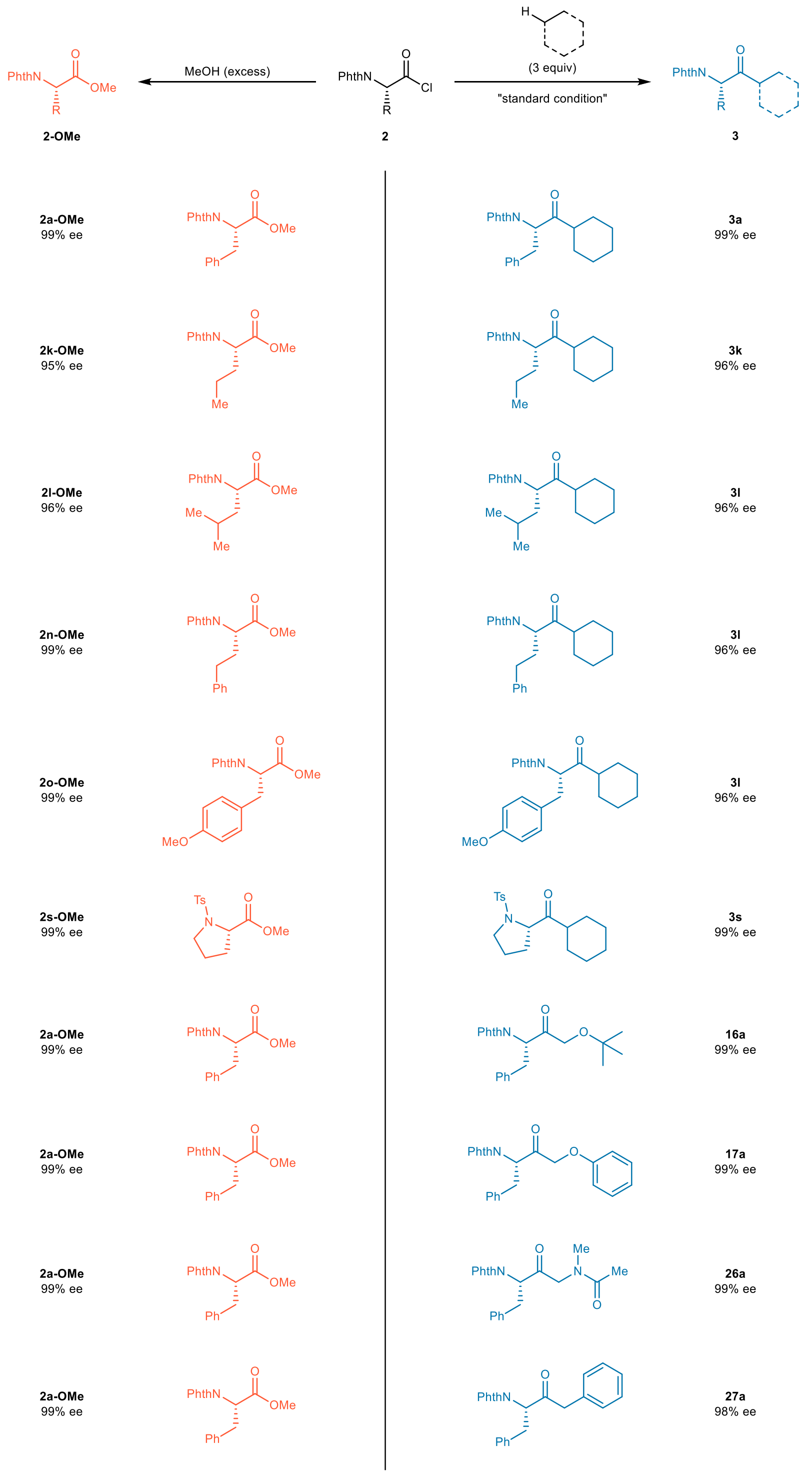

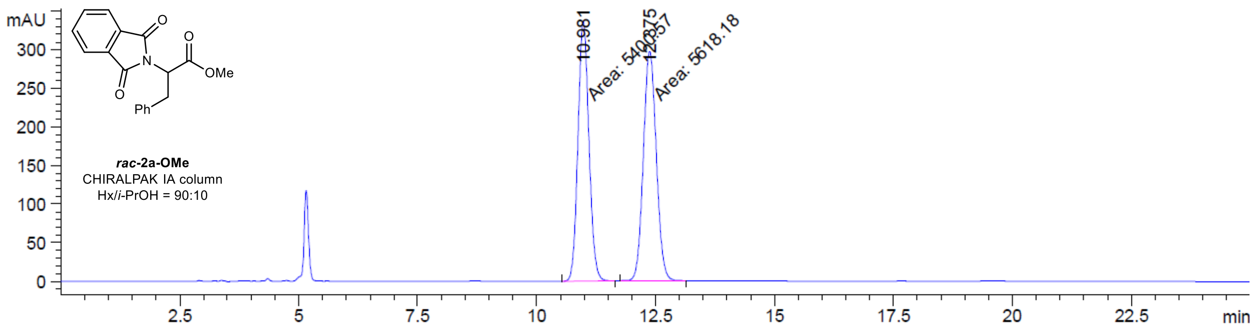

Peak #	RetTime [min]	Type	Width [min]	Area [mAU*s]	Height [mAU]	Area %
1	10.981	MM	0.2670	5400.57129	337.09979	49.0125
2	12.375	MM	0.3146	5618.18262	297.66446	50.9875

Supplementary Fig. 1. HPLC data of *rac*-2a-OMe.

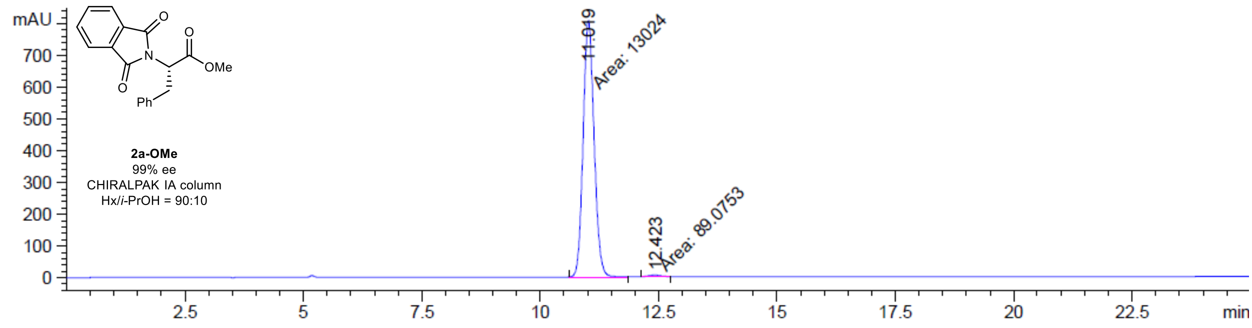

Peak #	RetTime [min]	Type	Width [min]	Area [mAU*s]	Height [mAU]	Area %
1	11.019	MM	0.2681	1.30240e4	809.52899	99.3207
2	12.423	MM	0.3102	89.07529	4.78657	0.6793

Supplementary Fig. 2. HPLC data of 2a-OMe.

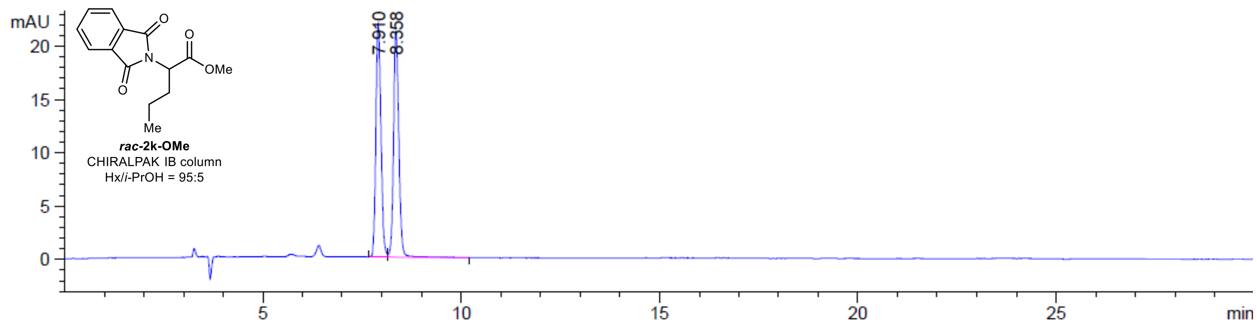

Peak #	RetTime [min]	Type	Width [min]	Area [mAU*s]	Height [mAU]	Area %
1	7.910	BV	0.1428	191.79808	21.76078	49.1959
2	8.358	VB	0.1499	198.06798	21.03254	50.8041

Supplementary Fig. 3. HPLC data of *rac*-2k-OMe.

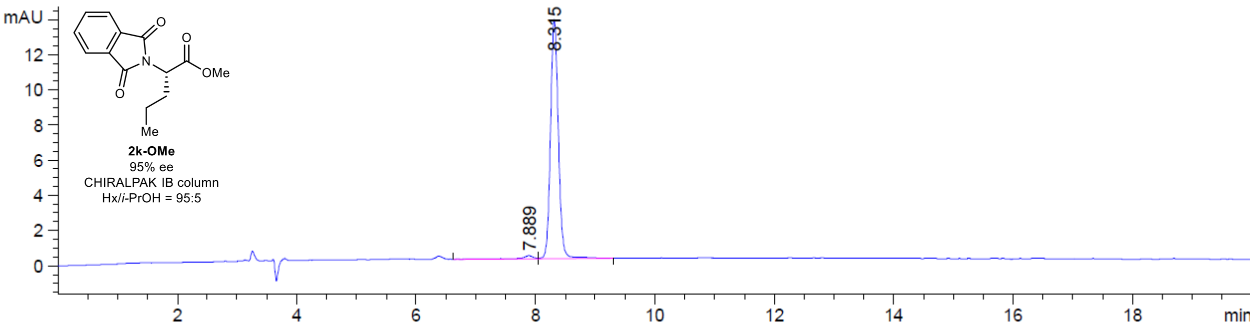

Peak #	RetTime [min]	Type	Width [min]	Area [mAU*s]	Height [mAU]	Area %
1	7.889	BV	0.2321	2.98673	1.81369e-1	2.3366
2	8.315	VB	0.1399	124.83967	13.50183	97.6634

Supplementary Fig. 4. HPLC data of 2k-OMe.

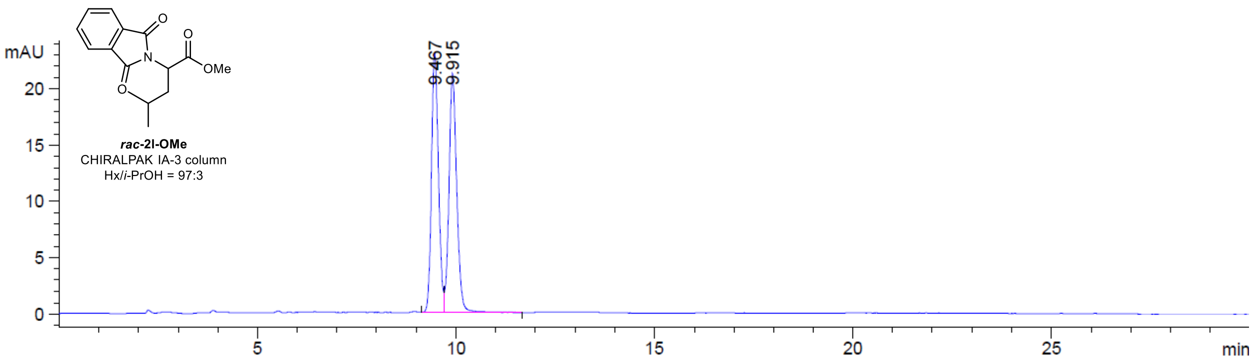

Peak #	RetTime [min]	Type	Width [min]	Area [mAU*s]	Height [mAU]	Area %
1	9.467	VV	0.1908	279.20599	22.80335	48.8340
2	9.915	VB	0.2087	292.53952	21.23294	51.1660

Supplementary Fig. 5. HPLC data of *rac*-2l-OMe.

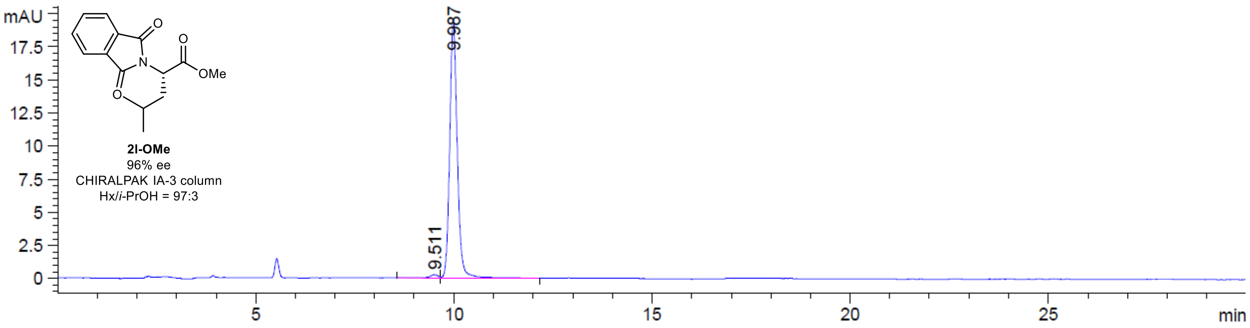

Peak #	RetTime [min]	Type	Width [min]	Area [mAU*s]	Height [mAU]	Area %
1	9.500	MM	0.3317	3.10132	1.55808e-1	1.8328
2	9.984	MM	0.2193	166.11063	12.62407	98.1672

Supplementary Fig. 6. HPLC data of 2l-OMe.

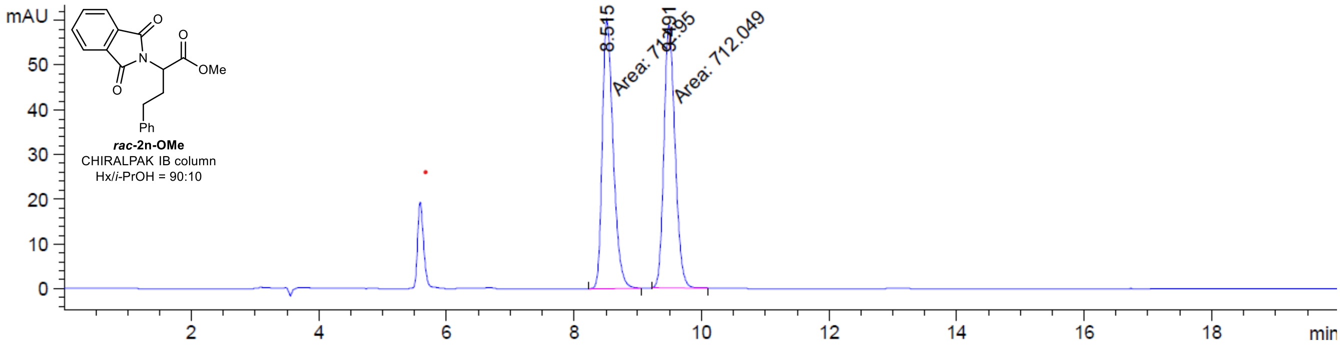

Peak #	RetTime [min]	Type	Width [min]	Area [mAU*s]	Height [mAU]	Area %
1	8.515	MM	0.1964	712.94995	60.48867	50.0316
2	9.491	MM	0.2010	712.04907	59.03448	49.9684

Supplementary Fig. 7. HPLC data of *rac*-2n-OMe.

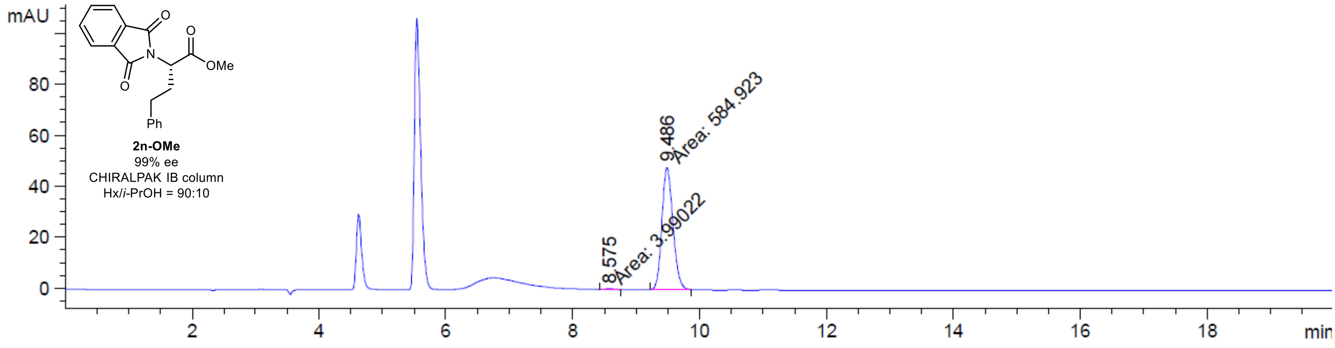

Peak #	RetTime [min]	Type	Width [min]	Area [mAU*s]	Height [mAU]	Area %
1	8.575	MM	0.1886	3.99022	3.52675e-1	0.6776
2	9.486	MM	0.2019	584.92279	48.27449	99.3224

Supplementary Fig. 8. HPLC data of 2n-OMe.

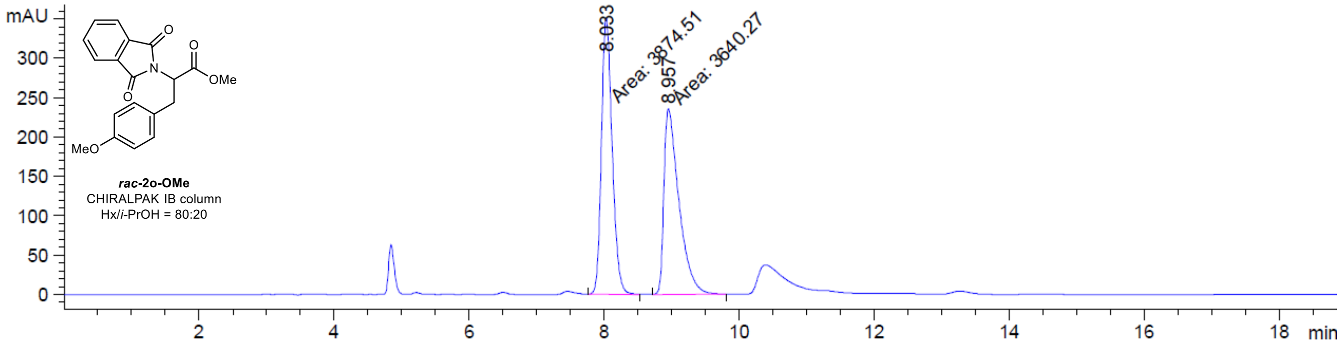

Peak #	RetTime [min]	Type	Width [min]	Area [mAU*s]	Height [mAU]	Area %
1	8.033	MM	0.1832	3874.51440	352.43948	51.5586
2	8.957	MM	0.2574	3640.26929	235.70370	48.4414

Supplementary Fig. 9. HPLC data of *rac*-2o-OMe.

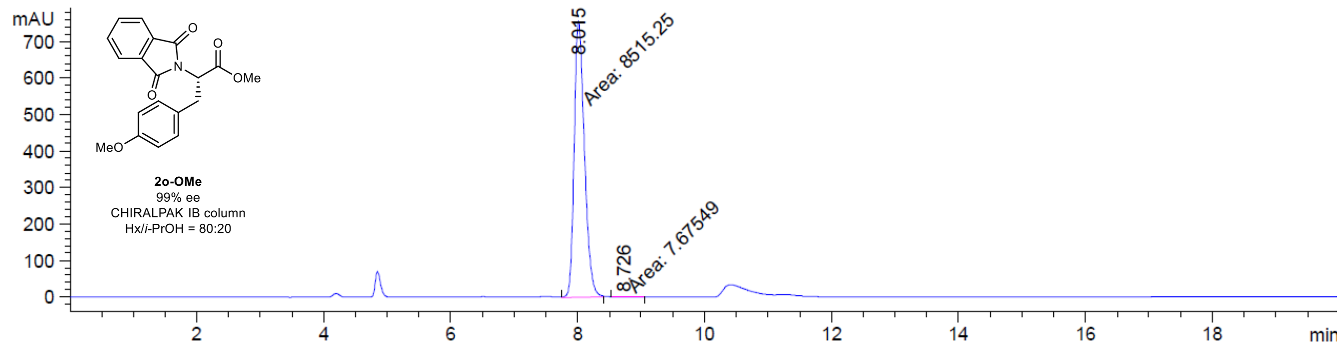

Peak #	RetTime [min]	Type	Width [min]	Area [mAU*s]	Height [mAU]	Area %
1	8.015	MM	0.1869	8515.25000	759.38739	99.9099
2	8.726	MM	0.2578	7.67549	4.96215e-1	0.0901

Supplementary Fig. 10. HPLC data of 2o-OMe.

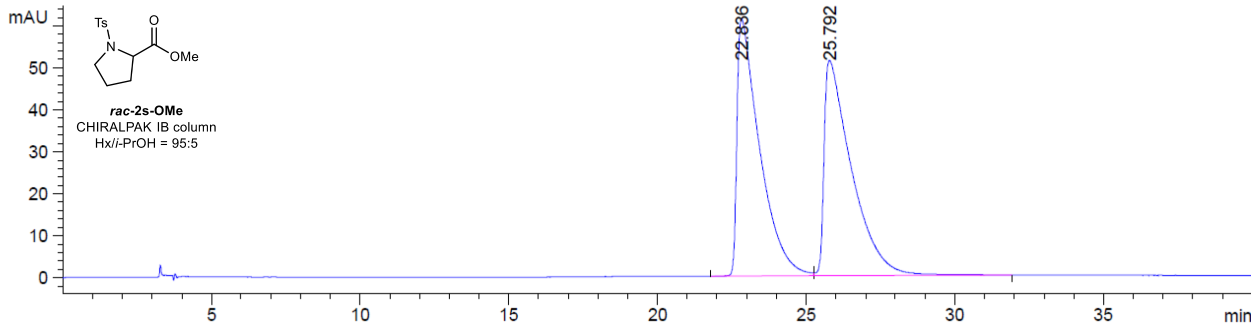

Peak #	RetTime [min]	Type	Width [min]	Area [mAU*s]	Height [mAU]	Area %
1	22.836	BV	0.7533	3237.17651	61.18142	49.5978
2	25.792	VB	0.9007	3289.68481	51.26220	50.4022

Supplementary Fig. 11. HPLC data of *rac*-2s-OMe.

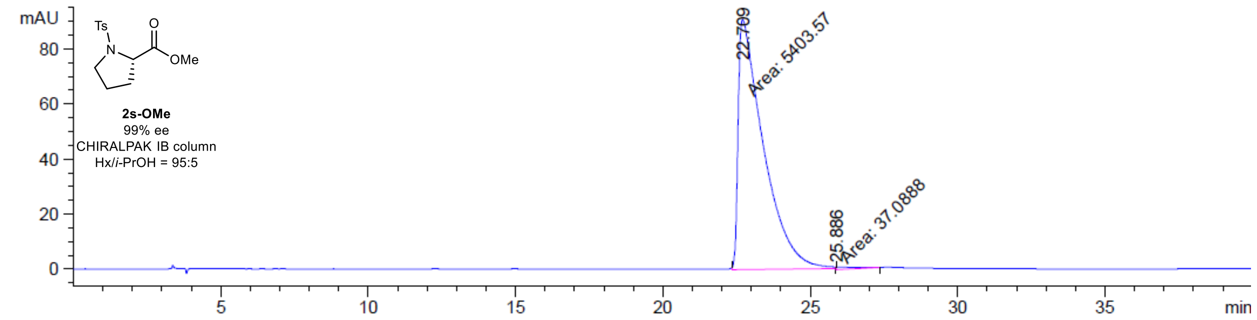

Peak #	RetTime [min]	Type	Width [min]	Area [mAU*s]	Height [mAU]	Area %
1	22.709	MM	0.9891	5403.56689	91.05006	99.3183
2	25.886	MM	0.5976	37.08878	1.03447	0.6817

Supplementary Fig. 12. HPLC data of 2s-OMe.

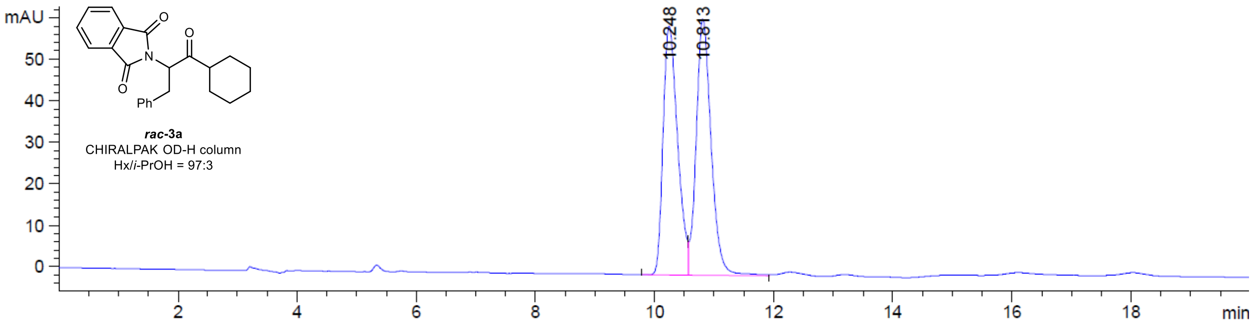

Peak #	RetTime [min]	Type	Width [min]	Area [mAU*s]	Height [mAU]	Area %
1	10.248	BV	0.2616	1011.45166	59.70695	48.3299
2	10.813	VV	0.2689	1081.35413	61.55577	51.6701

Supplementary Fig. 13. HPLC data of *rac*-3a.

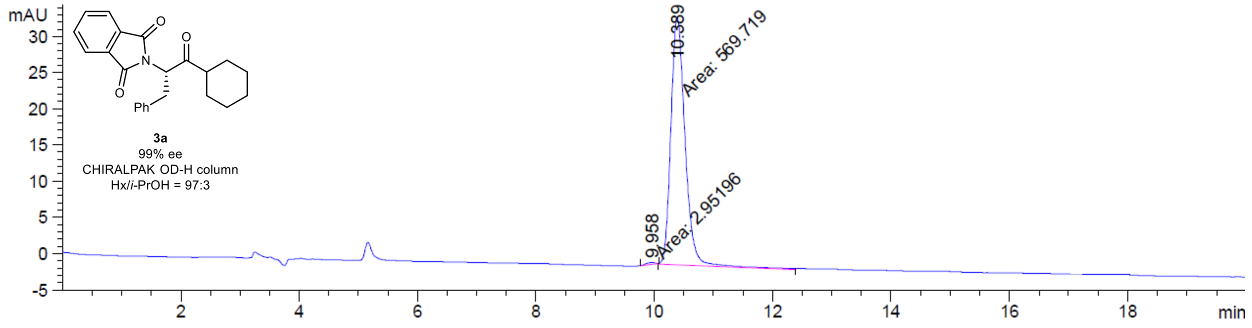

Peak #	RetTime [min]	Type	Width [min]	Area [mAU*s]	Height [mAU]	Area %
1	9.958	MM	0.1738	2.95196	2.83157e-1	0.5155
2	10.389	MM	0.2774	569.71948	34.23190	99.4845

Supplementary Fig. 14. HPLC data of 3a.

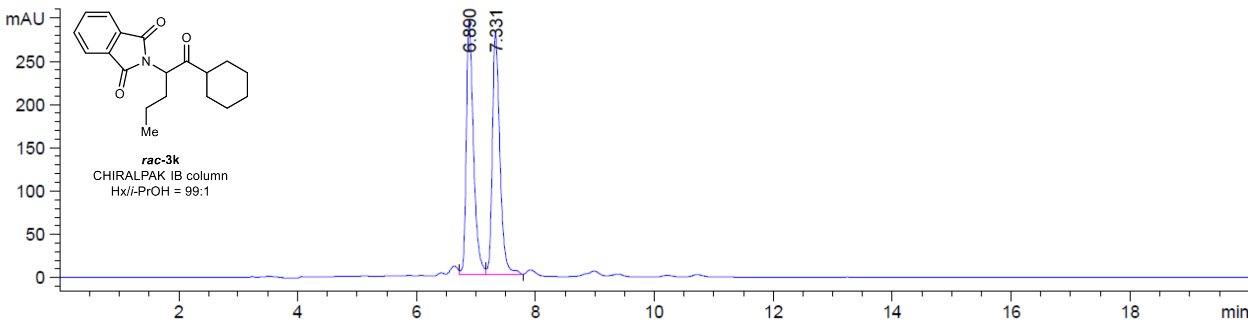

Peak #	RetTime [min]	Type	Width [min]	Area [mAU*s]	Height [mAU]	Area %
1	6.890	VV	0.1318	2459.79443	287.60660	49.9932
2	7.331	VB	0.1354	2460.46631	277.57343	50.0068

Supplementary Fig. 15. HPLC data of *rac*-3k.

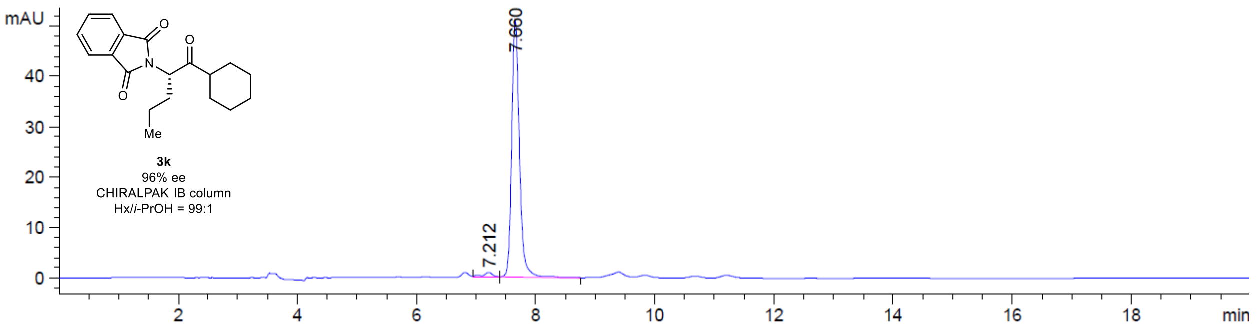

Peak #	RetTime [min]	Type	Width [min]	Area [mAU*s]	Height [mAU]	Area %
1	7.212	VB	0.1655	10.44204	9.12814e-1	2.2408
2	7.660	BB	0.1382	455.54849	50.05475	97.7592

Supplementary Fig. 16. HPLC data of 3k.

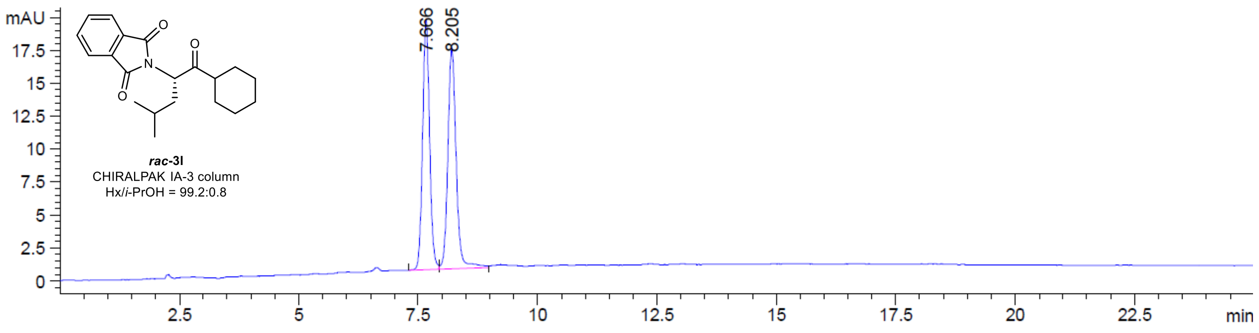

Peak #	RetTime [min]	Type	Width [min]	Area [mAU*s]	Height [mAU]	Area %
1	7.666	BV	0.1595	192.11444	18.75720	48.7329
2	8.205	VV	0.1814	202.10440	16.67777	51.2671

Supplementary Fig. 17. HPLC data of *rac*-3l.

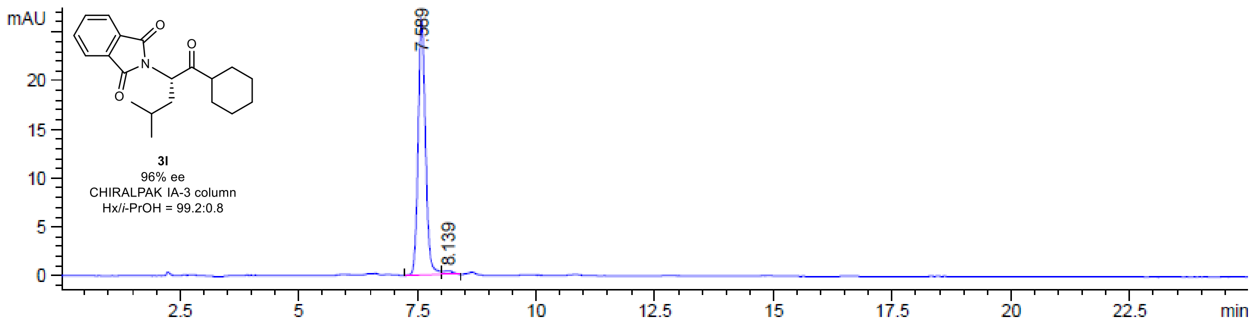

Peak #	RetTime [min]	Type	Width [min]	Area [mAU*s]	Height [mAU]	Area %
1	7.589	BV	0.1655	279.17279	25.94572	98.2058
2	8.139	VB	0.2090	5.10039	3.69614e-1	1.7942

Supplementary Fig. 18. HPLC data of 3l.

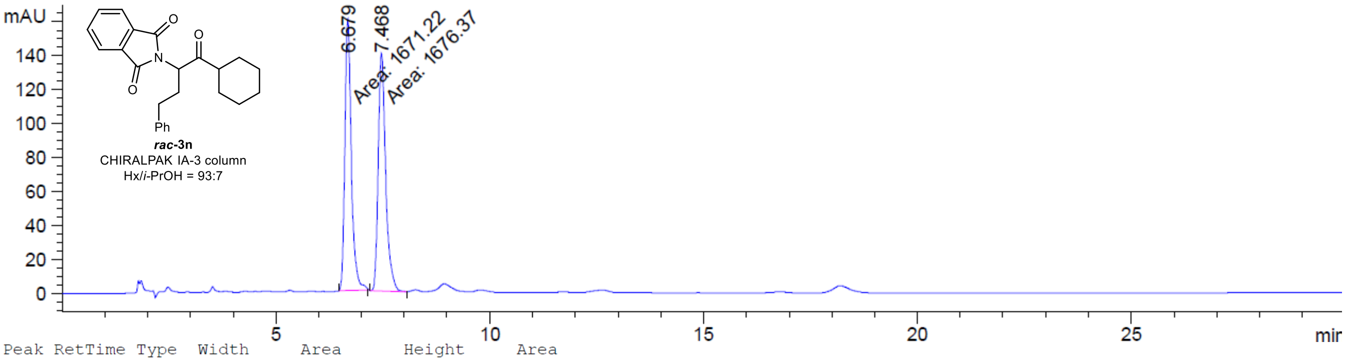

Peak #	RetTime [min]	Type	Width [min]	Area [mAU*s]	Height [mAU]	Area %
1	6.679	MM	0.1733	1671.22205	160.77118	49.9232
2	7.468	MM	0.1984	1676.36707	140.82825	50.0768

Supplementary Fig. 19. HPLC data of *rac*-3n.

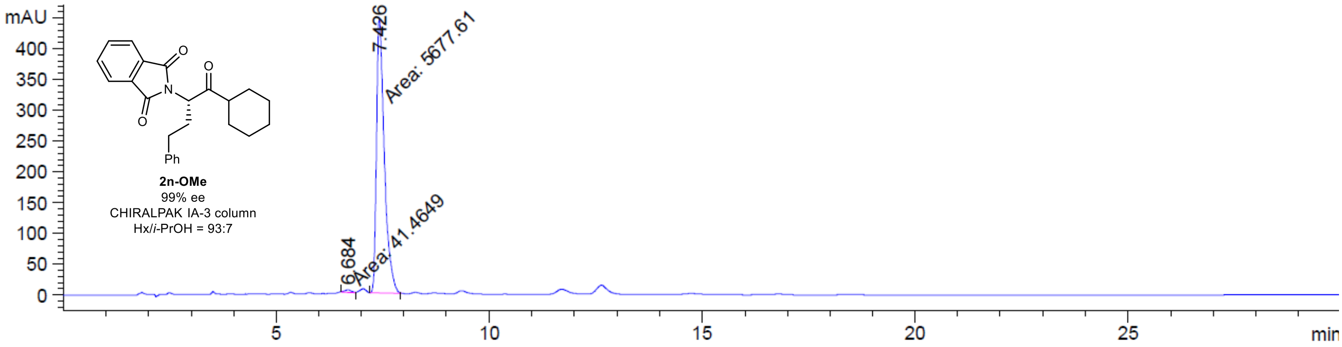

Peak #	RetTime [min]	Type	Width [min]	Area [mAU*s]	Height [mAU]	Area %
1	6.684	MM	0.1776	41.46488	3.89141	0.7250
2	7.426	MM	0.2120	5677.61328	446.28772	99.2750

Supplementary Fig. 20. HPLC data of 3n.

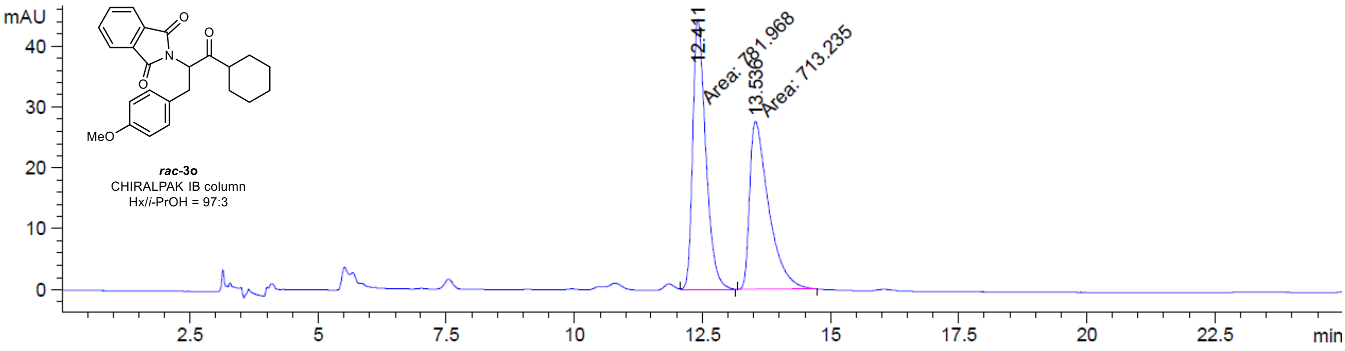

Peak #	RetTime [min]	Type	Width [min]	Area [mAU*s]	Height [mAU]	Area %
1	12.411	MM	0.2929	781.96777	44.49501	52.2984
2	13.536	MM	0.4307	713.23541	27.60274	47.7016

Supplementary Fig. 21. HPLC data of *rac*-3o.

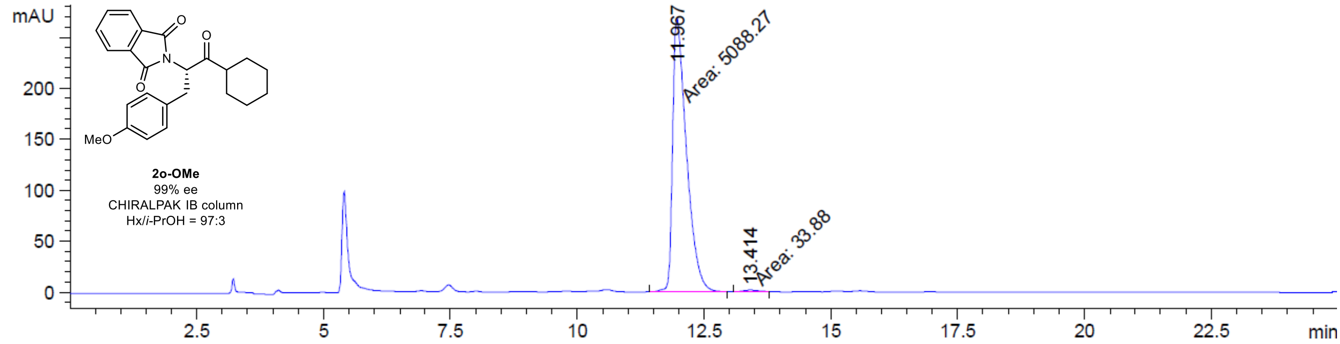

Peak #	RetTime [min]	Type	Width [min]	Area [mAU*s]	Height [mAU]	Area %
1	11.967	MM	0.3166	5088.27100	267.89728	99.3386
2	13.414	MM	0.3518	33.88003	1.60496	0.6614

Supplementary Fig. 22. HPLC data of 3o.

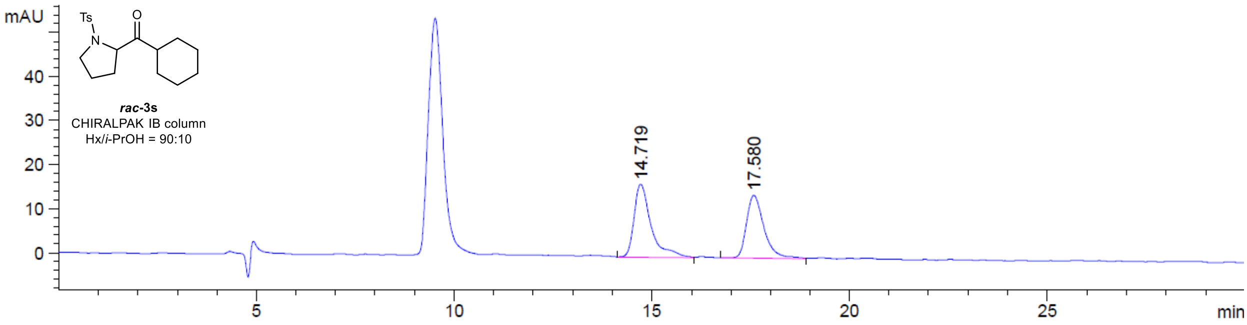

Peak #	RetTime [min]	Type	Width [min]	Area [mAU*s]	Height [mAU]	Area %
1	14.719	BV	0.4460	496.79291	16.56000	52.9697
2	17.580	BV	0.4717	441.08798	14.30511	47.0303

Supplementary Fig. 23. HPLC data of *rac*-3s.

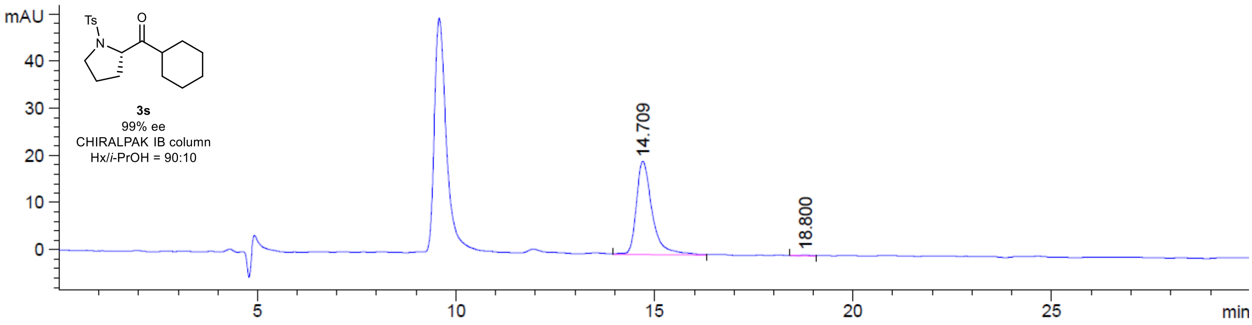

Peak #	RetTime [min]	Type	Width [min]	Area [mAU*s]	Height [mAU]	Area %
1	14.709	VV	0.4208	550.20227	19.76602	99.3101
2	18.800	BV	0.3525	3.82223	1.54568e-1	0.6899

Supplementary Fig. 24. HPLC data of 3s.

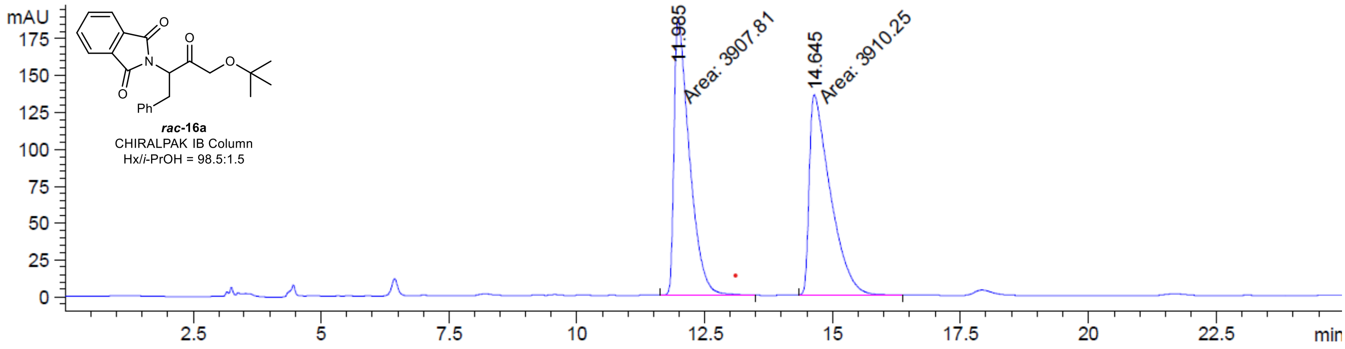

Peak #	RetTime [min]	Type	Width [min]	Area [mAU*s]	Height [mAU]	Area %
1	11.985	MM	0.3474	3907.80615	187.45976	49.9844
2	14.645	MM	0.4782	3910.25073	136.28975	50.0156

Supplementary Fig. 25. HPLC data of *rac*-16a.

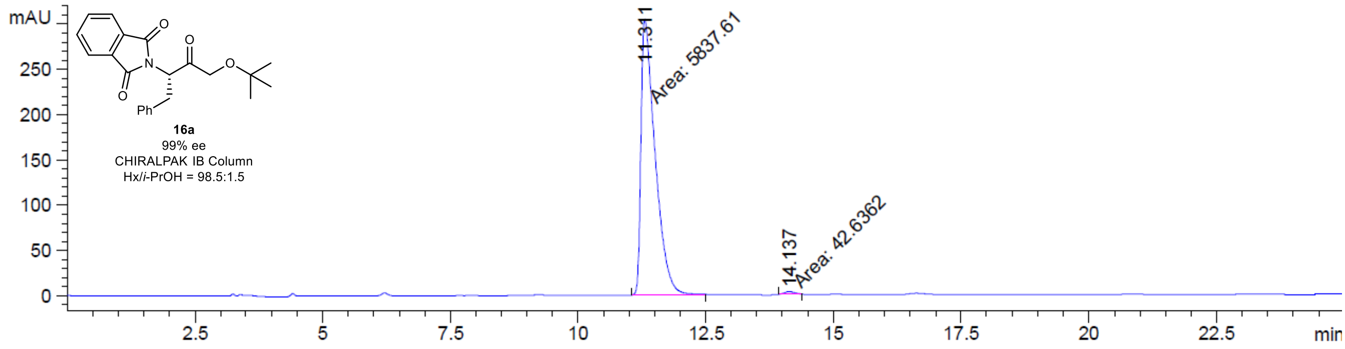

Peak #	RetTime [min]	Type	Width [min]	Area [mAU*s]	Height [mAU]	Area %
1	11.311	MM	0.3196	5837.61230	304.45767	99.2749
2	14.137	MM	0.2522	42.63615	2.81805	0.7251

Supplementary Fig. 26. HPLC data of 16a.

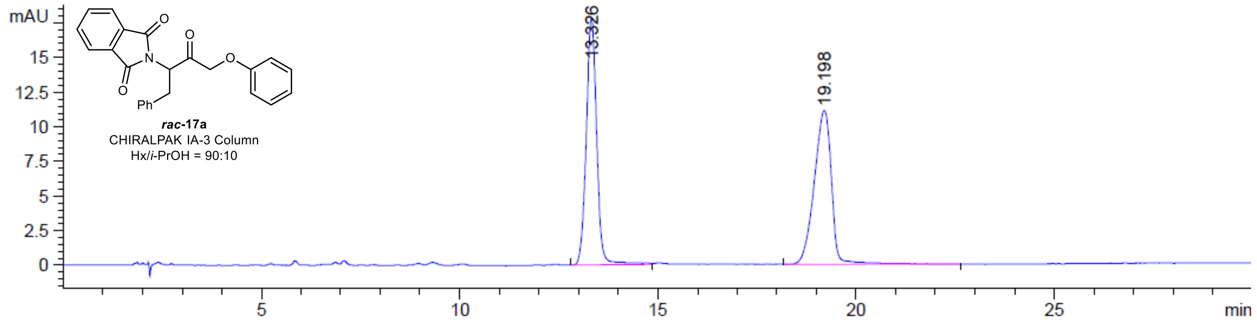

Peak #	RetTime [min]	Type	Width [min]	Area [mAU*s]	Height [mAU]	Area %
1	13.326	BV	0.2877	328.85968	17.78726	49.4348
2	19.198	BB	0.4733	336.37915	11.10818	50.5652

Supplementary Fig. 27. HPLC data of *rac*-17a.

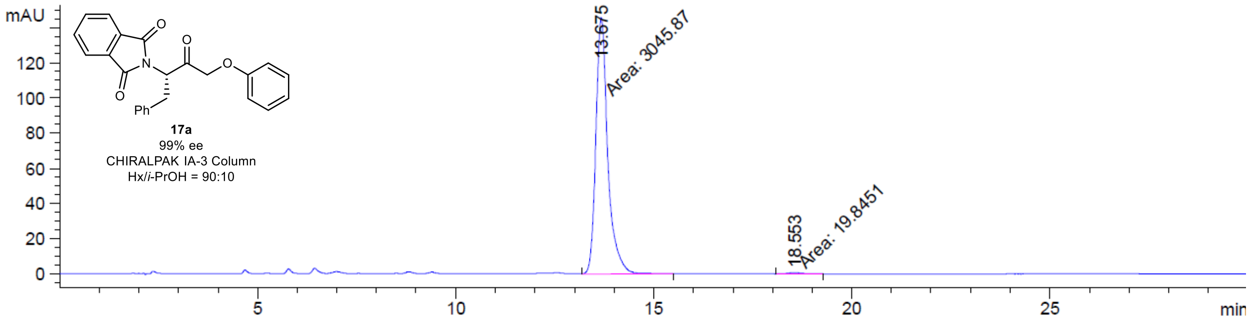

Peak #	RetTime [min]	Type	Width [min]	Area [mAU*s]	Height [mAU]	Area %
1	13.675	MM	0.3480	3045.86890	145.88727	99.3527
2	18.553	MM	0.4339	19.84510	7.62354e-1	0.6473

Supplementary Fig. 28. HPLC data of 17a.

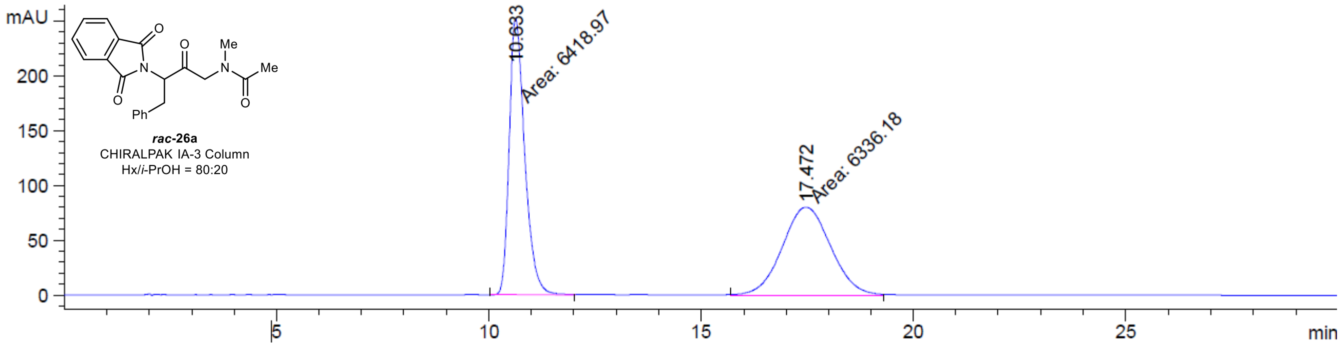

Peak #	RetTime [min]	Type	Width [min]	Area [mAU*s]	Height [mAU]	Area %
1	10.633	MM	0.4246	6418.96777	251.95949	50.3245
2	17.472	MM	1.3199	6336.18457	80.00816	49.6755

Supplementary Fig. 29. HPLC data of *rac*-26a.

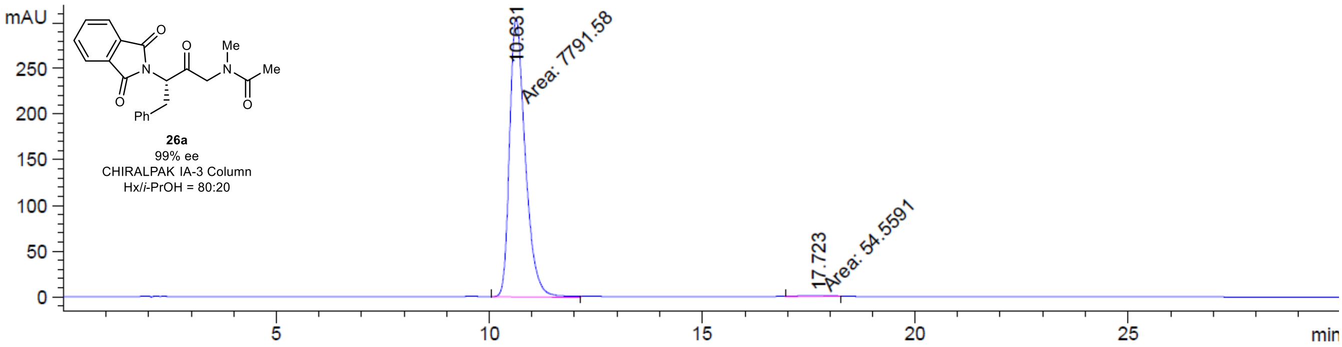

Peak #	RetTime [min]	Type	Width [min]	Area [mAU*s]	Height [mAU]	Area %
1	10.631	MM	0.4258	7791.57910	304.96057	99.3046
2	17.723	MM	0.9425	54.55915	9.64835e-1	0.6954

Supplementary Fig. 30. HPLC data of *rac*-26a.

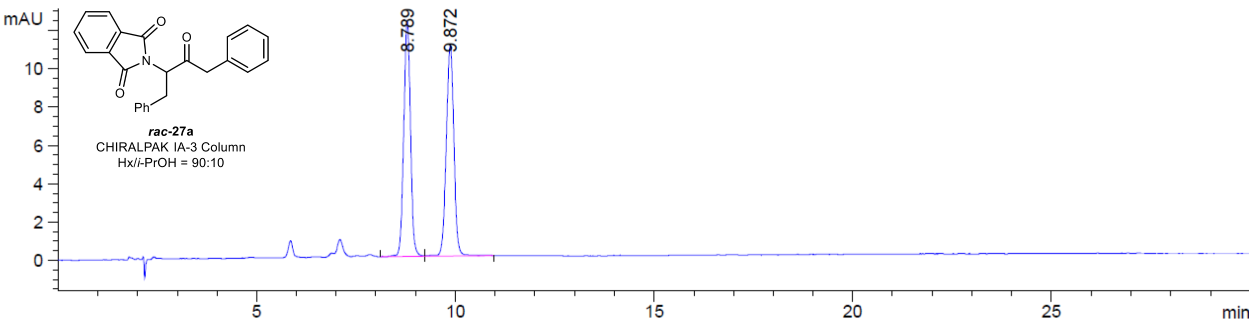

Peak #	RetTime [min]	Type	Width [min]	Area [mAU*s]	Height [mAU]	Area %
1	8.789	BV	0.1771	143.27980	12.19266	49.4685
2	9.872	VB	0.2043	146.35883	10.92695	50.5315

Supplementary Fig. 31. HPLC data of *rac*-27a.

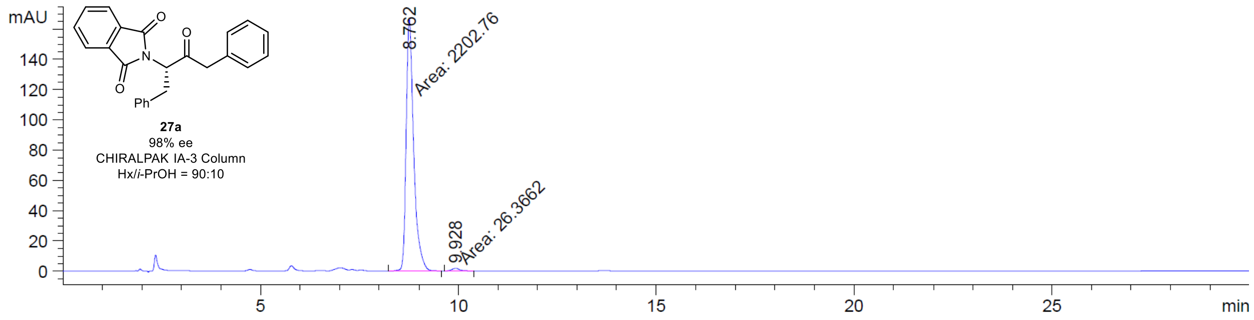

Peak #	RetTime [min]	Type	Width [min]	Area [mAU*s]	Height [mAU]	Area %
1	8.762	MM	0.2188	2202.76123	167.77756	98.8172
2	9.928	MM	0.2513	26.36622	1.74876	1.1828

Supplementary Fig. 32. HPLC data of 27a.

2. Supplementary Discussion

2.1. Control Experiments in Previously Reported Ni/Ir-Catalyzed Acylation Conditions

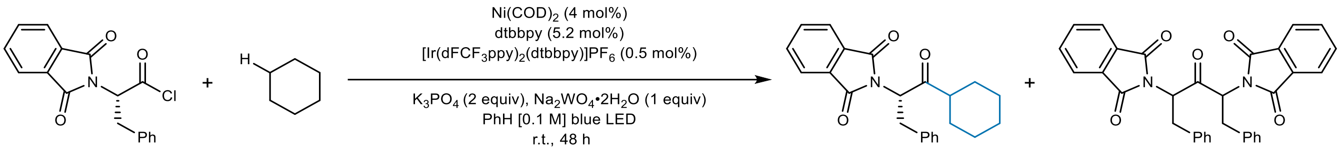

2a (3 equiv) **3a** 93% ee 25% isolated yield **2a-dimer** 1:1 d.r. 26% isolated yield

Supplementary Fig. 33. Oxidative addition initiated pathway.[18]

To a 8 mL vial equipped with a stirrer-bar were added K_3PO_4 (84.9 mg, 0.40 mmol, 2.0 equiv), Na_2WO_4•$2H_2O$ (66.0 mg, 0.20 mmol, 1 equiv), Ni(COD) (2.2 mg, 0.008 mmol, 0.04 equiv), dtbbpy (4,4′-di-*tert*-butyl-2,2′-dipyridyl) (2.8 mg, 0.0104 mmol, 0.052 equiv), Ir[dF(CF_3)ppy]$_2$(dtbbpy)PF_6 (1.1 mg, 0.001 mmol, 0.005 equiv), cyclohexane (64.8 μL, 0.60 mmol, 3.0 equiv), **2a** (62.7 mg, 0.20 mmol, 1.0 equiv), and benzene (2.0 mL). The resulting mixture was stirred for 48 h under blue LED irradiation in a Penn PhD M2 photoreactor (1200 stir rpm, 6800 fan rpm, 100% light intensity). The reaction mixture was then filtered through a pad of silica, washed with CH_2Cl_2 and concentrated under reduced pressure. The resulting residue was purified by flash column chromatography (silica gel, hexanes/EtOAc or hexanes/Et_2O gradient elution) to afford **3a** (18.0 mg, 25%) and dimerized byproduct (**2a-dimer**, 13.8 mg, 26%). The ee of the isolated product was analyzed by HPLC.

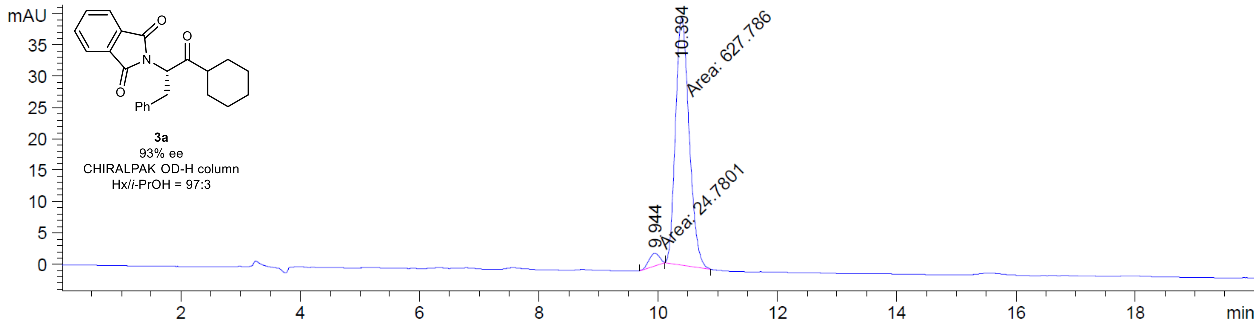

Peak #	RetTime [min]	Type	Width [min]	Area [mAU*s]	Height [mAU]	Area %
1	9.944	MM	0.2007	24.78012	2.05818	3.7973
2	10.394	MM	0.2646	627.78607	39.54578	96.2027

Supplementary Fig. 34. HPLC data of 3a from oxidative addition initiated pathway.

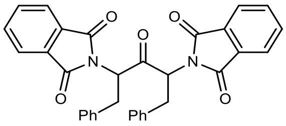

2,2'-(3-Oxo-1,5-diphenylpentane-2,4-diyl)bis(isoindoline-1,3-dione) (2a-dimer)

White solid; m.p. 167–169 °C; ^{1}H NMR (500 MHz, $CDCl_3$, 1:1 mixture of diastereomers): δ = 7.79 – 7.66 (m, 4H), 7.65 – 7.58 (m, 4H), 7.14 – 6.96 (m, 10H), 5.27 (dd, *J* = 10.5, 5.5 Hz, 1H), 4.96 (dd, *J* = 11.1, 4.7 Hz, 1H), 3.61 – 3.55 (m, 1H), 3.55 – 3.44 (m, 2H), 3.32 – 3.25 (m, 1H); ^{13}C NMR (125 MHz, $CDCl_3$): δ = 200.0, 198.6, 167.5, 167.3, 136.4, 136.2, 134.3, 134.2, 131.4, 131.2, 129.0, 128.8, 128.6, 128.4, 126.9, 126.7, 123.6, 123.3, 58.0, 56.9, 33.9, 33.6; IR (cm^{-1}) 2929, 1774, 1709, 1605, 1468, 1379, 1100, 717, 699; HRMS-EI (m/z) [M]$^+$ calcd for $C_{33}H_{24}N_2O_5$, 528.1685; found: 528.1682.

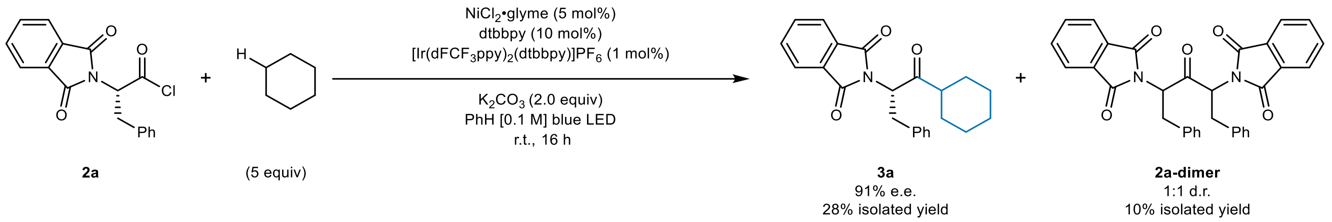

Supplementary Fig. 35. C–H activation initiated pathway using 2a.[3]

To a 8 mL vial equipped with a stirrer-bar were added K_2CO_3 (55.3 mg, 0.40 mmol, 2.0 equiv), $NiCl_2$•glyme (2.2 mg, 0.01 mmol, 0.05 equiv), dtbbpy (4,4′-di-*tert*-butyl-2,2′-dipyridyl) (5.4 mg, 0.02 mmol, 0.10 equiv), Ir[dF(CF_3)ppy]$_2$(dtbbpy)PF_6 (2.2 mg, 0.002 mmol, 0.01 equiv), cyclohexane (108 µL, 1.0 mmol, 5.0 equiv), **2a** (62.7 mg, 0.20 mmol, 1.0 equiv), and benzene (2.0 mL). The resulting mixture was stirred for 16 h under blue LED irradiation in a Penn PhD M2 photoreactor (1200 stir rpm, 6800 fan rpm, 100% light intensity). The reaction mixture was then diluted with HCl (1 M aq., 5 mL), extracted with CH_2Cl_2 (3 × 5 mL), dried (anhydrous Na_2SO_4), filtered and concentrated under reduced pressure. The resulting residue was purified by flash column chromatography (silica gel, hexanes/EtOAc or hexanes/Et_2O gradient elution) to afford **3a** (20.4 mg, 28%) and dimerized byproduct (**2a-dimer**, 5.5 mg, 10%). The ee of the isolated product was analyzed by HPLC.

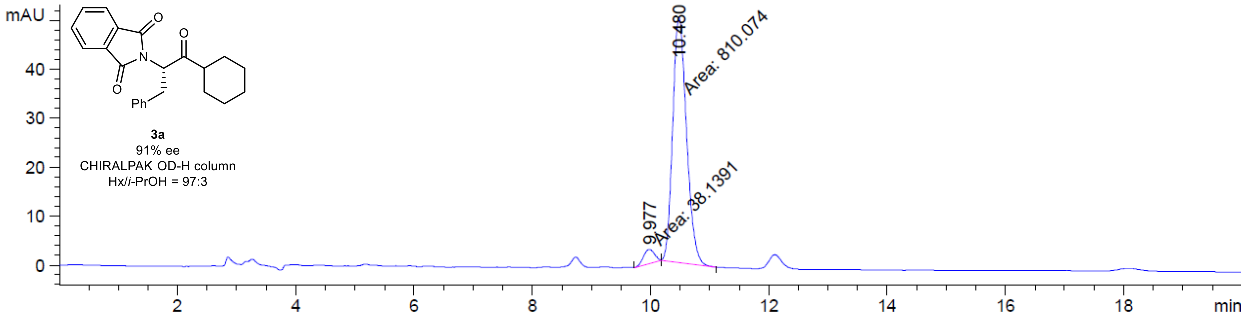

Peak #	RetTime [min]	Type	Width [min]	Area [mAU*s]	Height [mAU]	Area %
1	9.977	MM	0.2166	38.13913	2.93435	4.4964
2	10.480	MM	0.2689	810.07391	50.21828	95.5036

Supplementary Fig. 36. HPLC data of 3a from C–H activation initiated pathway.

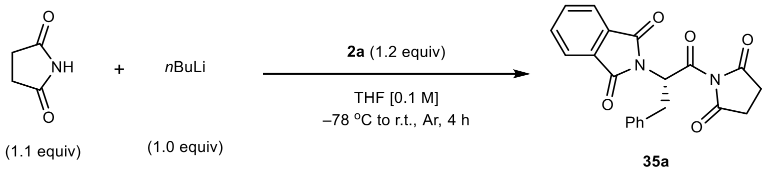

Compound **35a** was prepared following a slightly modified literature procedure.[19] To a 50 mL round bottom flask equipped with a stirrer-bar were added succinimide (43.6 mg, 0.44 mmol, 1.1 equiv), and THF (4 mL). To the reaction mixture, 2.5 M solution of *n*-butyllithium in hexanes (0.16 mL, 0.40 mmol, 1.0 equiv) was added slowly at –78 °C under Ar atmosphere. After 30 min of stirring, **2a** (150.6 mg, 0.48 mmol, 1.2 equiv) dissolved in THF was added to the reaction mixture. After 10 min the solution was allowed to reach 0 °C. The reaction mixture was then diluted with $NaHCO_3$ (5 mL), extracted with CH_2Cl_2 (3 × 5 mL), dried (anhydrous Na_2SO_4), filtered and concentrated under reduced pressure. The resulting residue was purified by flash column chromatography (silica gel, hexanes/EtOAc gradient elution), and recrystallization in dichloromethane/hexanes to afford **35a** (85.6 mg, 57%). The ee of the isolated product was analyzed by HPLC.

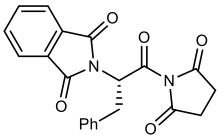

(*S*)-2-(1-(2,5-Dioxopyrrolidin-1-yl)-1-oxo-3-phenylpropan-2-yl)isoindoline-1,3-dione (35a)

White solid, 85.6 mg (0.23 mmol, 57% yield); m.p. 170–172 °C; ^{1}H NMR (500 MHz, $CDCl_3$): δ = 7.83 – 7.74 (m, 2H), 7.72 – 7.67 (m, 2H), 7.25 – 7.12 (m, 5H), 5.93 (t, *J* = 7.9 Hz, 1H), 3.52 (d, *J* = 7.9 Hz, 2H), 2.73 (s, 4H); ^{13}C NMR (125 MHz, $CDCl_3$): δ = 173.3, 168.5, 167.1, 135.9, 134.3, 131.4, 129.2, 128.6, 127.1, 123.7, 57.0, 34.4, 29.6, 28.5; IR (cm^{-1}) 1799, 1777, 1755, 1708, 1383, 1317, 1178, 1083, 718, 702, 617, 521; HRMS-EI (m/z) [M]$^{+}$ calcd for $C_{21}H_{16}N_2O_5$, 376.1059; found: 376.1055.

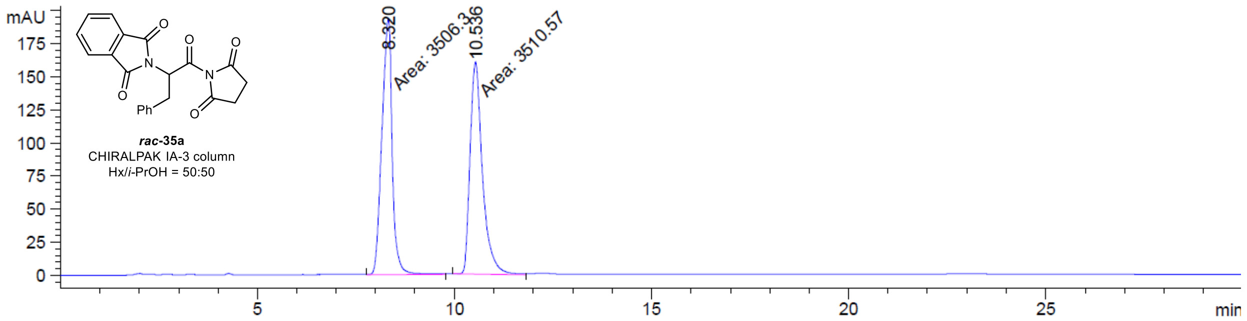

Peak #	RetTime [min]	Type	Width [min]	Area [mAU*s]	Height [mAU]	Area %
1	8.321	MM	0.3031	3943.94604	216.88805	50.7708
2	10.535	MM	0.3576	3824.19336	178.23180	49.2292

Supplementary Fig. 37. HPLC data of *rac*-35a.

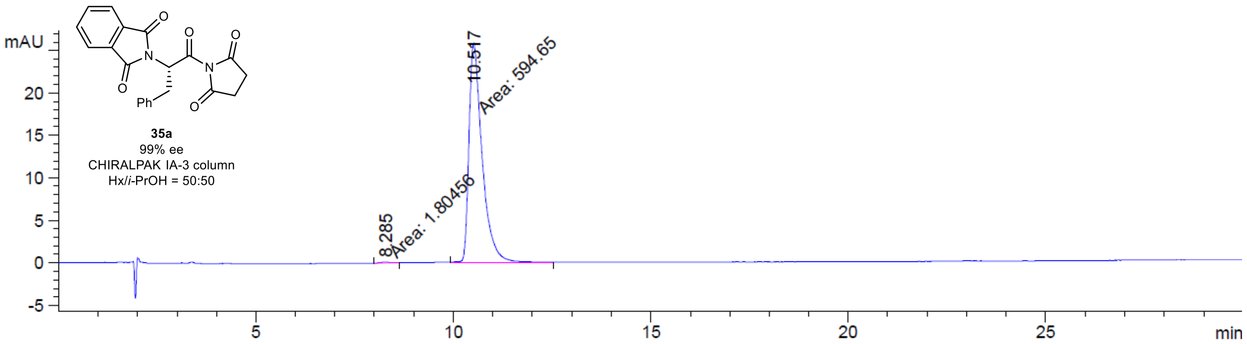

Peak #	RetTime [min]	Type	Width [min]	Area [mAU*s]	Height [mAU]	Area %
1	8.285	MM	0.3333	1.80456	9.02363e-2	0.3025
2	10.517	MM	0.3841	594.64990	25.80243	99.6975

Supplementary Fig. 38. HPLC data of 35a.

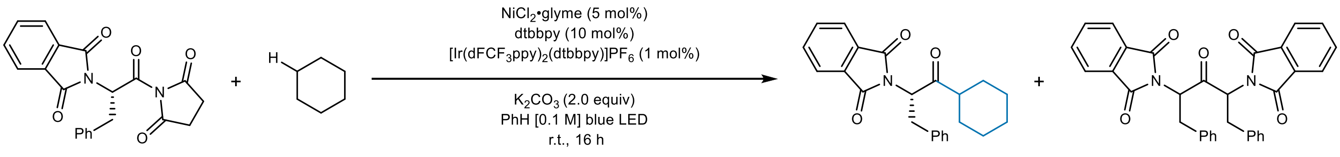

35a (5 equiv) 3a N.D. 2a-dimer N.D.

Supplementary Fig. 39. C–H activation initiated pathway using 35a.[3]

To a 8 mL vial equipped with a stirrer-bar were added K_2CO_3 (55.3 mg, 0.40 mmol, 2.0 equiv), $NiCl_2$•glyme (2.2 mg, 0.01 mmol, 0.05 equiv), dtbbpy (4,4′-di-*tert*-butyl-2,2′-dipyridyl) (5.4 mg, 0.02 mmol, 0.10 equiv), Ir[dF(CF_3)ppy]$_2$(dtbbpy)PF_6

(2.2 mg, 0.002 mmol, 0.01 equiv), cyclohexane (108 μL, 1.0 mmol, 3.0 equiv), **35a** (75.2 mg, 0.20 mmol, 1.0 equiv), and benzene (2.0 mL). The resulting mixture was stirred for 16 h under blue LED irradiation in a Penn PhD M2 photoreactor (1200 stir rpm, 6800 fan rpm, 100% light intensity). The reaction mixture was then diluted with HCl (1 M aq., 5 mL), extracted with CH_2Cl_2 (3 × 5 mL), dried (anhydrous Na_2SO_4), filtered and concentrated under reduced pressure. The resulting residue was analyzed by crude NMR after the addition of 1,1,2,2-tetrachloroethane (6 μL) as an internal standard.

2.2. Intermolecular Competition Experiment

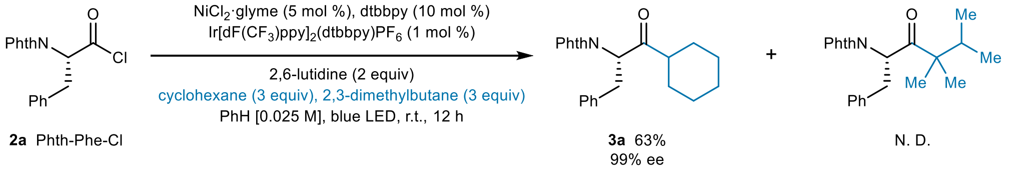

Supplementary Fig. 40. Intermolecular competition experiment.

To an 8 mL vial equipped with a PTFE-coated stirrer bar were added **2a** (62.7 mg, 0.20 mmol, 1.0 equiv), $NiCl_2$·glyme (2.2 mg, 0.01 mmol, 0.05 equiv), dtbbpy (5.37 mg, 0.02 mmol, 0.10 equiv), Ir[dF(CF_3)ppy]$_2$(dtbbpy)PF_6 (2.2 mg, 0.002 mmol, 0.01 equiv), 2,6-lutidine (46.6 µL, 0.40 mmol, 2.0 equiv), cyclohexane (64.8 µL, 0.60 mmol, 3.0 equiv), 2,3-dimethylbutane (78.3 µL, 0.60 mmol, 3.0 equiv), and benzene (8.0 mL). The resulting mixture was stirred for 12 h under blue LED irradiation in a Penn PhD M2 photoreactor (1200 stir rpm, 6800 fan rpm, 100% light intensity). The reaction mixture was then diluted with HCl (1 M aq., 5 mL), extracted with CH_2Cl_2 (3 × 5 mL), dried (anhydrous Na_2SO_4), filtered, and concentrated under reduced pressure. The reaction mixture was filtered through a short pad of Celite®, eluted with CH_2Cl_2, and concentrated under reduced pressure. The resulting residue was purified by flash column chromatography (silica gel, hexanes/Et_2O gradient elution) to afford 63% of **3a**. No product derived from 2,3-dimethylbutane was detected. The ee of the obtained product **3a** was analyzed by chiral HPLC.

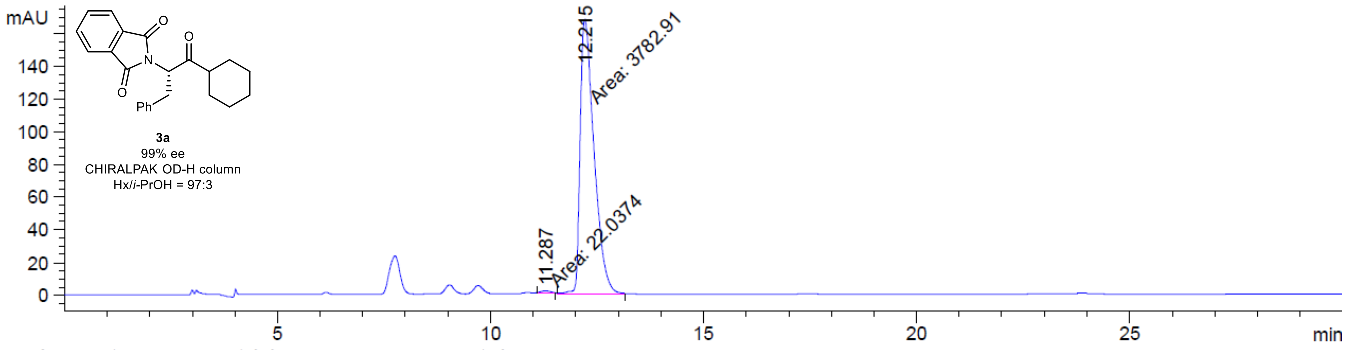

Peak #	RetTime [min]	Type	Width [min]	Area [mAU*s]	Height [mAU]	Area %
1	11.287	MM	0.2591	22.03741	1.41775	0.5792
2	12.215	MM	0.3746	3782.90845	168.29033	99.4208

Supplementary Fig. 41. HPLC data of 3a from C–H intermolecular competition experiment.

2.3. NMR Studies for the Equilibrium of N-Acyllutidinium Intermediate

^{1}H NMR Study with the 2a and 2,6-Lutidine

In an NMR tube, amino acid chloride **2a** (31.4 mg, 0.1 mmol) and the corresponding amount of 2,6-lutidine were dissolved in $CDCl_3$ (1 mL). Before each experiment, the sample was mixed and allowed to equilibrate for 10 min before spectra were obtained. Throughout the investigation, a clear tendency of the chemical shift was observed in ^{1}H NMR reasoned from the equilibrium present in $CDCl_3$ (Supplementary Fig. 42). The observation, upfield shift of **2a** and downfield shift of 2,6-lutidine methyl peak, was in good accordance with the NMR studies reported by the Wu group.[20]

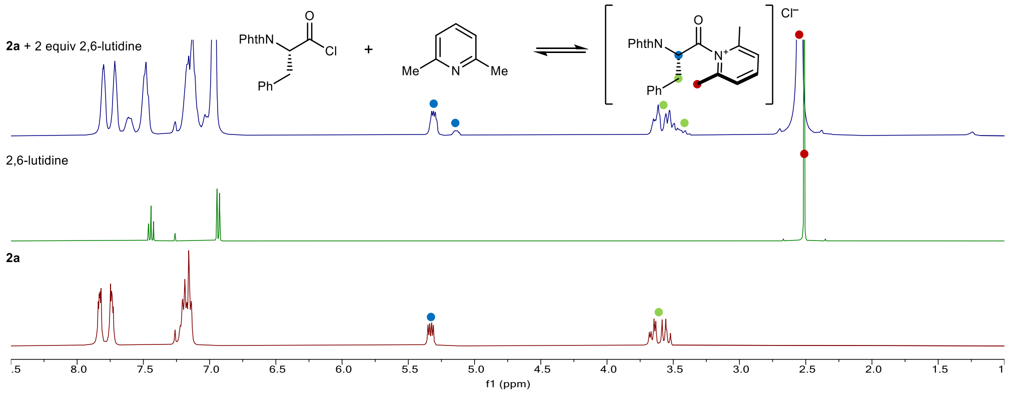

Supplementary Fig. 42. ^{1}H NMR spectra of 2a and 2,6-lutidine in $CDCl_3$.

Nuclear Overhauser Effect Experiment of the N-Acyllutidinium Intermediate

In an NMR tube, the amino acid chloride **2a** (31.4 mg, 0.1 mmol) and 2,6-lutidine (57.9 µL, 0.5 mmol, 5.0 equiv) were dissolved in $CDCl_3$ (1 mL). The sample was mixed and allowed to equilibrate for 10 min before spectra were obtained (Supplementary Fig. 43).

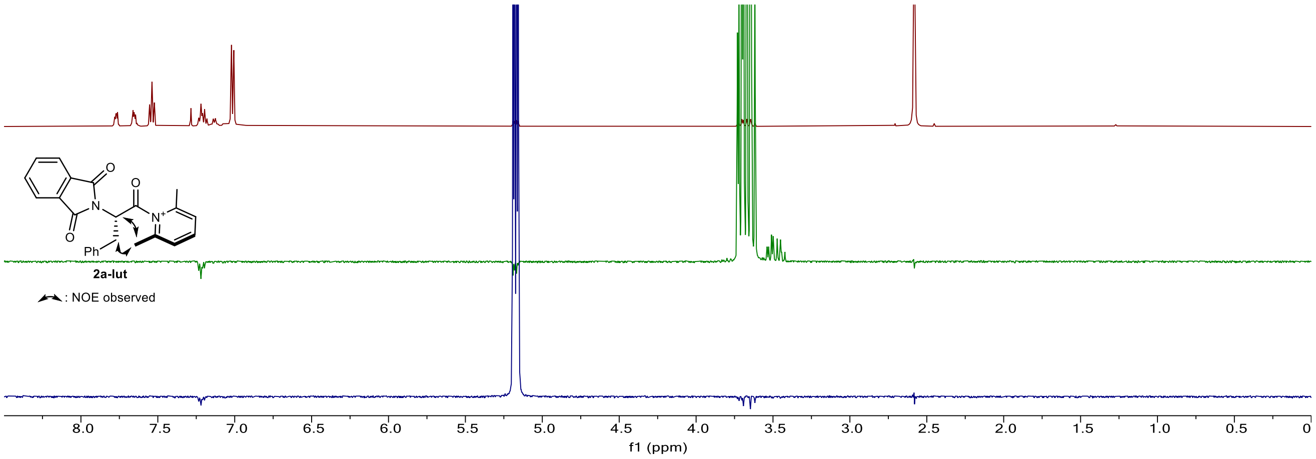

Supplementary Fig. 43. 1D selective gradient NOESY spectra (selective to 3.68 ppm: green, selective to 5.18 ppm: blue) of 2a and 2,6-lutidine (1:5) in $CDCl_3$.

2.4. IR Studies for the Equilibrium of N-Acyllutidinium Intermediate

IR spectroscopy was performed with acyl chloride **2aa** (13.4 µL, 0.1 mmol) before and after adding an equimolar amount of 2,6-lutidine. The intensity of the carbonyl peak (1789 cm^{-1}) of **2aa** was decreased with the appearance of a new absorption band at 1741 cm^{-1} (Supplementary Fig. 44–45).

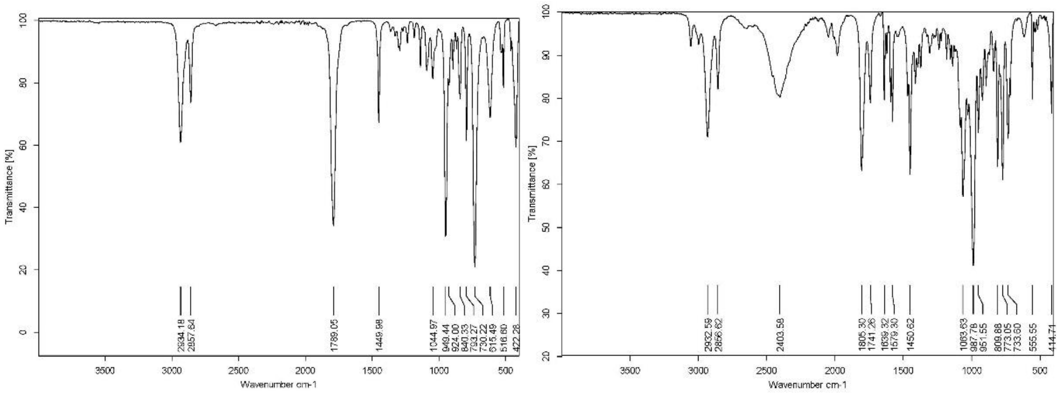

Supplementary Fig. 44. IR spectra of 2aa (left) and that with 1 equiv 2,6-lutidine (right).

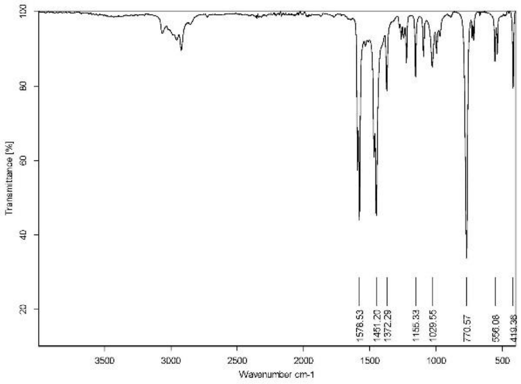

Supplementary Fig. 45. IR spectra of 2,6-lutidine.

2.5. Radical Scavenger Experiment

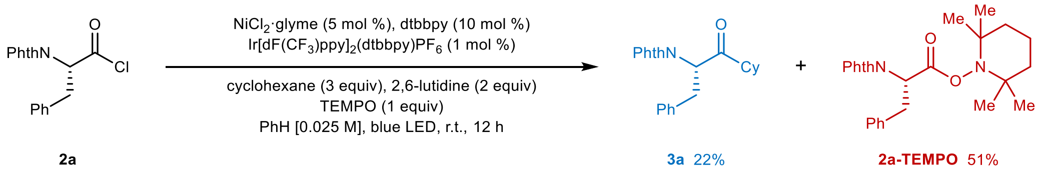

Supplementary Fig. 46. Radical scavenger experiment.

To a 8 mL vial equipped with a stirrer-bar were added **2a** (62.7 mg, 0.20 mmol), $NiCl_2$·glyme (2.2 mg, 0.01 mmol, 0.05 equiv), dtbbpy (4,4′-di-*tert*-butyl-2,2′-dipyridyl) (5.37 mg, 0.02 mmol, 0.10 equiv), Ir[dF(CF_3)ppy]$_2$(dtbbpy)PF_6 (2.2 mg, 0.002 mmol, 0.01 equiv), 2,6-lutidine (46.6 µL, 0.40 mmol, 2.0 equiv), cyclohexane (64.8 µL, 0.60 mmol, 3.0 equiv), (2,2,6,6-tetramethylpiperidin-1-yl)oxyl (TEMPO, 31.2 mg, 0.20 mmol, 1.0 equiv), and benzene (8.0 mL). The resulting mixture was stirred for 12 h under blue LED irradiation in a Penn PhD M2 photoreactor (1200 stir rpm, 6800 fan rpm, 100% light intensity). The reaction mixture was then diluted with HCl (1 M aq., 5 mL), extracted with CH_2Cl_2 (3 × 5 mL), dried (anhydrous Na_2SO_4), filtered, and concentrated under reduced pressure. The resulting residue was analyzed by crude NMR after the addition of 1,1,2,2-tetrachloroethane (3 µL) as an internal standard (Supplementary Fig. 47).

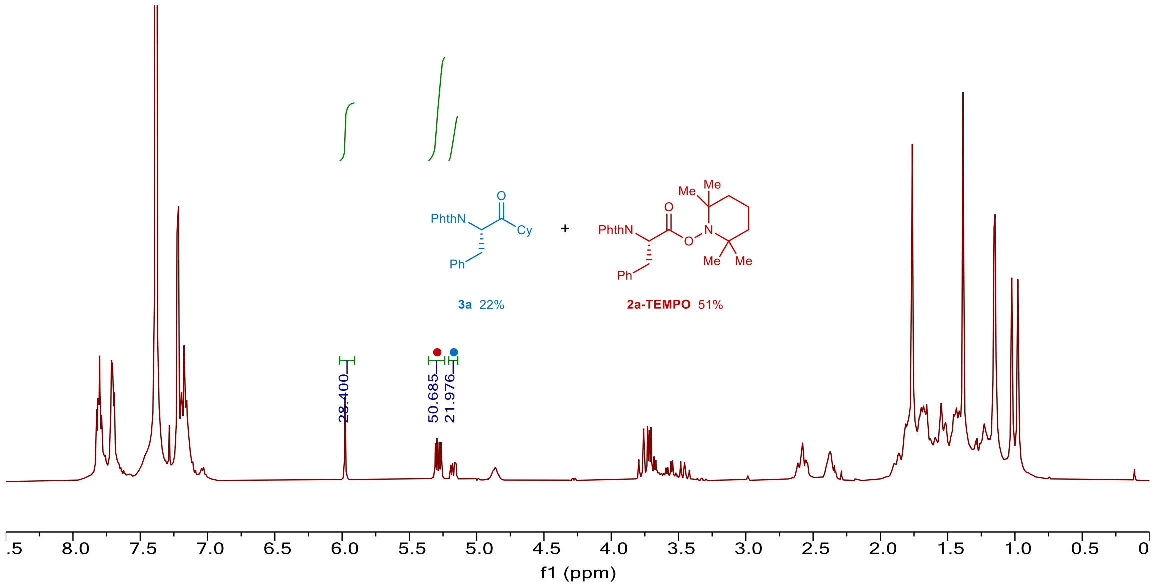

Supplementary Fig. 47. Crude spectra of the radical scavenger experiment.

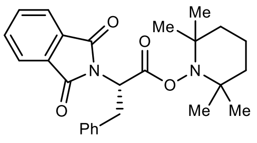

2,2,6,6-Tetramethylpiperidin-1-yl (*S*)-2-(1,3-dioxoisoindolin-2-yl)-3-phenylpropanoate (2a-TEMPO)

White solid; m.p. 152–154 °C; ^{1}H NMR (500 MHz, $CDCl_3$): δ = 7.82 – 7.74 (m, 2H), 7.73 – 7.66 (m, 2H), 7.25 – 7.14 (m, 4H), 7.13 (p, J = 4.1 Hz, 1H), 5.25 (dd, J = 11.7, 5.1 Hz, 1H), 3.81 – 3.62 (m, 2H), 1.70 – 1.34 (m, 6H), 1.11 (d, J = 4.1 Hz, 6H), 0.99 (s, 3H), 0.94 (s, 3H); ^{13}C NMR (125 MHz, $CDCl_3$, mixture of rotamer): δ = 168.3, 167.6, 136.8, 134.2, 131.6, 128.8, 128.6, 126.8, 123.4, 60.5, 60.4, 52.8, 39.2, 39.1, 34.4, 31.9, 31.8, 20.5, 20.3, 16.9; IR (cm^{-1}) 2929, 1771, 1712, 1389, 1171, 946, 874, 745, 722, 698, 529; HRMS-EI (m/z) $[M]^+$ calcd for $C_{26}H_{30}N_2O_4$, 434.2206; found: 434.2202.

2.6. Cyclic Voltammetry Experiments

Cyclic Voltammetry (CV) experiments were carried out (Supplementary Fig. 48–51). Due to a complex cyclic voltammogram of **2a**, cyclohexane carbonyl chloride (**2aa**) was used as a model substrate. The reduction wave of **2aa**/**2aa**$^{-}$ was observed to be –2.45 V vs SCE. With the addition of lutidine, its original reduction wave gradually decreased while a new reduction wave gradually increased at –0.95 V vs SCE (Supplementary Fig. 49–51). This data corresponds well with the computed reduction potential of N-acyllutidinium intermediate (–1.05 V vs SCE, see section 14. Computational Details).

Calibration of the Reference Electrode

<Electrode Composition>

Working electrode: glassy carbon.

Reference electrode: Ag/$AgNO_3$.

Counter electrode: graphite

<Procedure>

Ferrocene (**Fc**, 0.001 M) and tetrabutylammonium perchlorate (TBAP, 0.1 M) in acetonitrile (20 mL) were transferred to an electrochemical cell equipped with the above three-electrode system. The CV was obtained using a potentiostat with a scan rate 0.1 V/s and a scan range of +0.5 to –0.5 V.

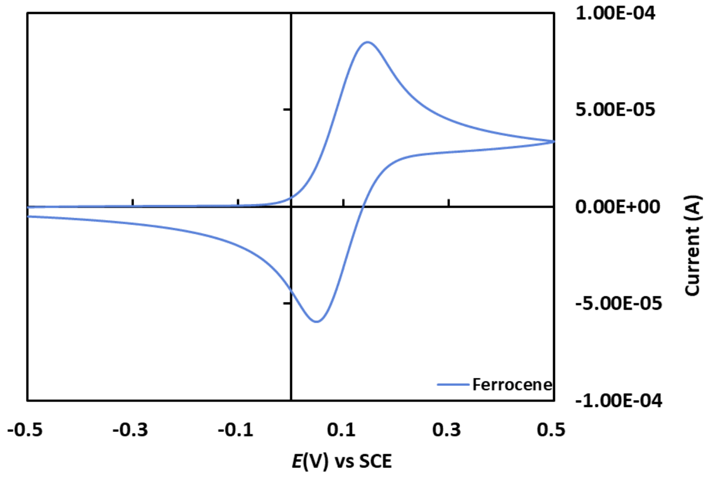

Supplementary Fig. 48. Cyclic voltammogram of Fc/Fc^{+}.

Measured: $E_{1/2}$[**Fc/Fc^{+}**] = 0.081 vs Ag/$AgNO_3$

Reference: $E_{1/2}$[**Fc/Fc^{+}**] = 0.380 vs SCE (Saturated Calomel Electrode)

The conversion constant was measured to be +0.299 V.

2aa and 2,6-lutidine

<Electrode Composition>

Working electrode: glassy carbon.

Reference electrode: Ag/$AgNO_3$.

Counter electrode: graphite

<Procedure>

The changes in the reduction wave of **2aa** (10 mM) were measured after adding the corresponding amount of 2,6-lutidine (no addition, 10 mM, 50 mM, and 100 mM). The substrates were prepared in a tetrabutylammonium perchlorate (TBAP, 0.1

M) solution in acetonitrile (20 mL). The CVs were measured in an electrochemical cell equipped with the three-electrode system. The CVs were obtained using a potentiostat with a scan rate of 0.05 V/s and a scan range of +0.0 to –2.9 V.

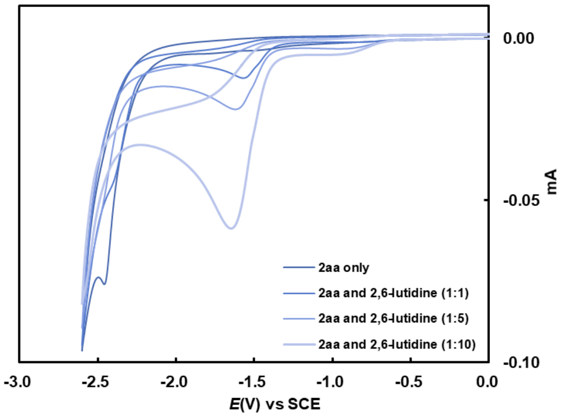

Supplementary Fig. 49. Cyclic voltammogram with variable ratio of 2aa and 2,6-lutidine.

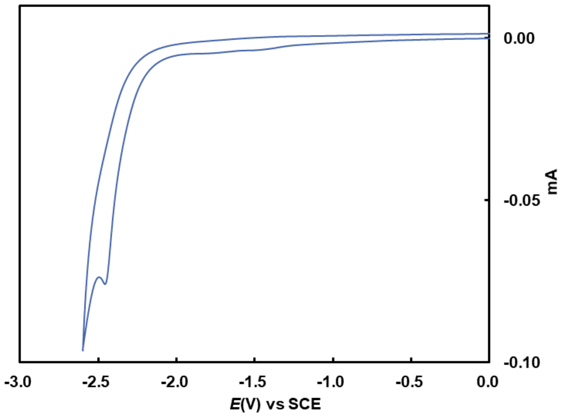

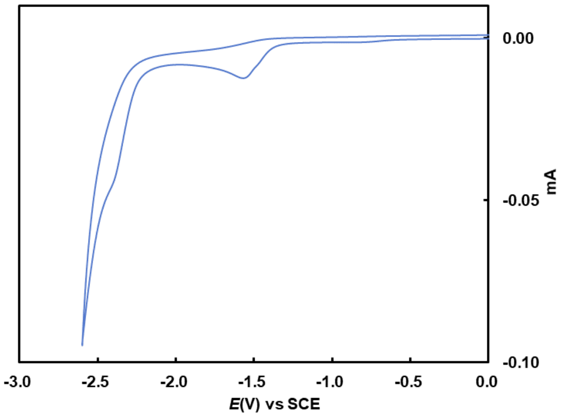

Supplementary Fig. 50. Cyclic voltammogram of 2aa (left), and that of 2aa and 2,6-lutidine (1:1, right).

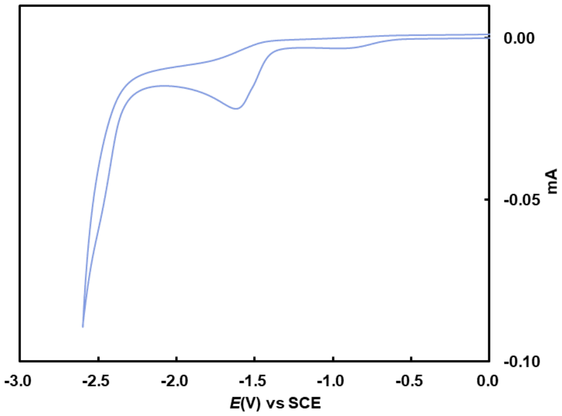

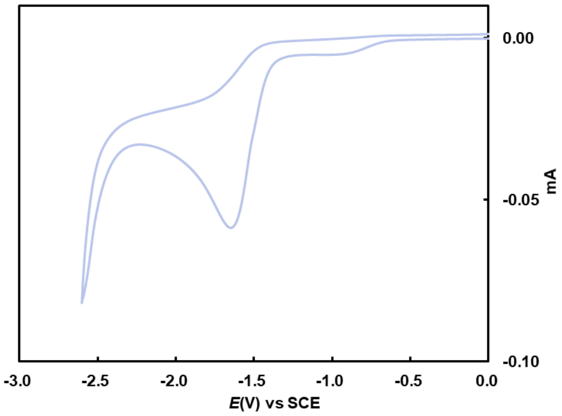

Supplementary Fig. 51. Cyclic voltammogram of 2aa (1:5, left), and that of 2aa and 2,6-lutidine (1:10, right).

2.7. Stern-Volmer Quenching Experiments

Stern-Volmer quenching experiments were conducted with 25 μM Ir[dF(CF_3)ppy]$_2$(dtbbpy)PF_6 in benzene. **2a**, 2,6,-lutidine was used as quenchers. The samples were excited at 380 nm, and emission peaks were observed at 475 nm to obtain the Stern-Volmer regression.

The fluorescence spectra and the Stern-Volmer regression data are shown below (Supplementary Fig. 52–54).

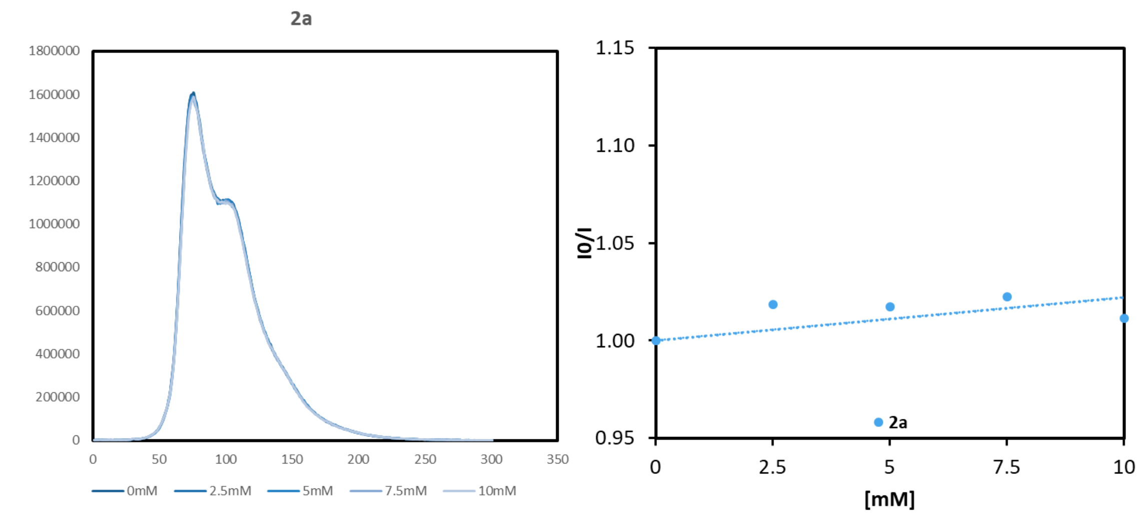

Supplementary Fig. 52. Stern-Volmer quenching experiment with 2a.

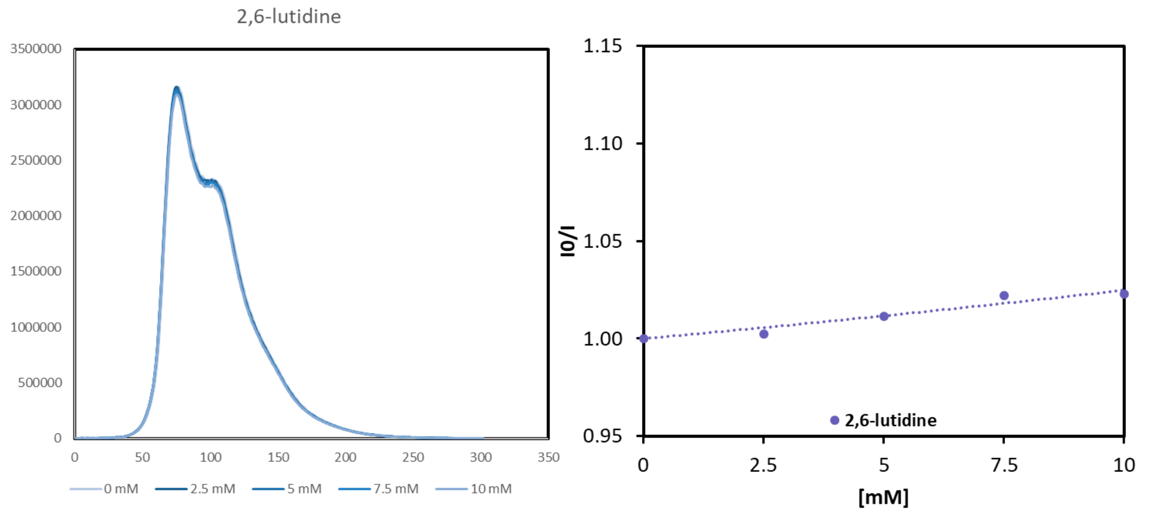

Supplementary Fig. 53. Stern-Volmer quenching experiment with 2,6-lutidine.

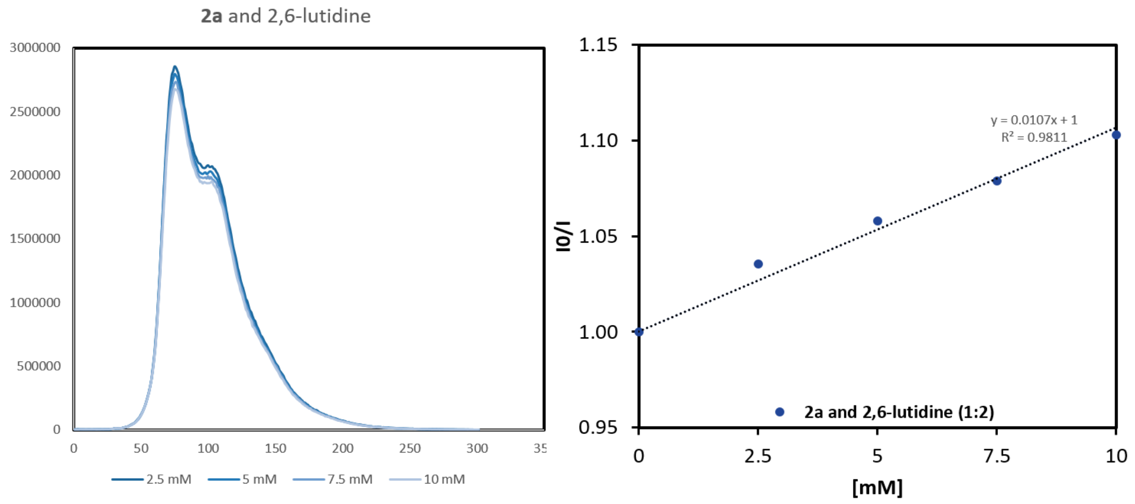

Supplementary Fig. 54. Stern-Volmer quenching experiment with 2a and 2,6-lutidine (1:2).

The quenching experiment revealed **2a** and 2,6-lutidine in benzene are not good quenchers individually for the iridium photocatalyst. In contrast, when a mixture of **2a** and 2,6-lutidine (1:2 ratio) was added, an enhanced quenching of the excited photocatalyst was observed ($\mathbf{K}_{SV}$ = 10.7 M^{-1}).

2.8. Kinetic Isotope Effect Measurements

KIE experiments were conducted using cyclohexane and cyclohexane-d_{12}. Both initial rate difference and intermolecular competition experiments were performed.

Initial Rate Difference from Parallel Reactions

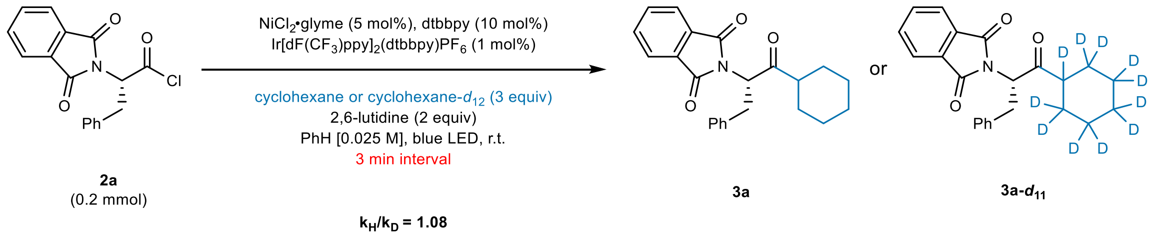

Supplementary Fig. 55. KIE experiment of parallel reactions.

To a 8 mL vial equipped with a stirrer-bar were added **2a** (62.7 mg, 0.20 mmol), $NiCl_2$·glyme (2.2 mg, 0.01 mmol, 0.05 equiv), dtbbpy (4,4′-di-*tert*-butyl-2,2′-dipyridyl) (5.37 mg, 0.02 mmol, 0.10 equiv), $Ir[dF(CF_3)ppy]_2(dtbbpy)PF_6$ (2.2 mg, 0.002 mmol, 0.01 equiv), 2,6-lutidine (46.6 μL, 0.40 mmol, 2.0 equiv), cyclohexane or cyclohexane-d_{12} (0.60 mmol, 3.0 equiv), dodecane (internal standard, 20 μL), and benzene (8.0 mL). The resulting mixture was stirred in a 34W blue LED lamp irradiation with fan cooling. Aliquots of the reaction mixture were taken for 3 min interval and analyzed by gas chromatography to determine the product yield by comparing signal ratio. The initial rates were compared and the kinetic isotope effects (KIE) were measured to be 1.08 (Supplementary Fig. 56).

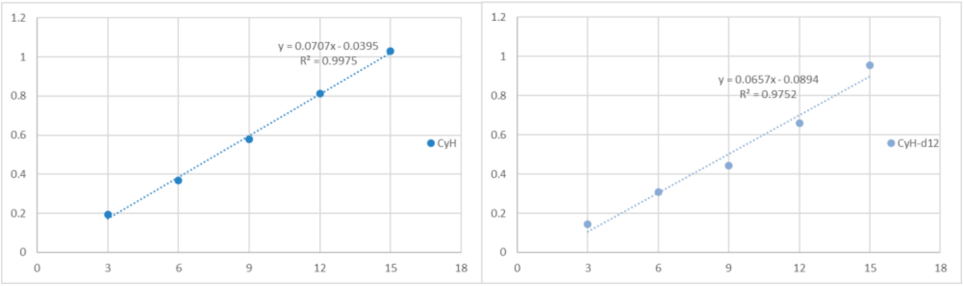

Supplementary Fig. 56. KIE measurement with 2a (left: cyclohexane, right: cyclohexane-d_{12}).

Intermolecular Competition Experiment

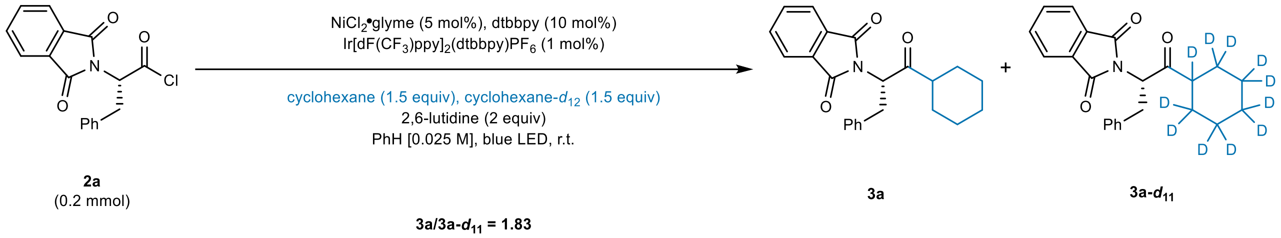

Supplementary Fig. 57. KIE experiment of intermolecular competition reaction.

To a 8 mL vial equipped with a stirrer-bar were added **2a** (62.7 mg, 0.20 mmol), $NiCl_2$·glyme (2.2 mg, 0.01 mmol, 0.05 equiv), dtbbpy (4,4′-di-*tert*-butyl-2,2′-dipyridyl) (5.37 mg, 0.02 mmol, 0.10 equiv), $Ir[dF(CF_3)ppy]_2(dtbbpy)PF_6$ (2.2 mg, 0.002 mmol, 0.01 equiv), 2,6-lutidine (46.6 μL, 0.40 mmol, 2.0 equiv), cyclohexane (32 μL, 0.30 mmol, 1.5 equiv), cyclohexane-d_{12} (32 μL, 0.30 mmol, 1.5 equiv), and benzene (8.0 mL). The resulting mixture was stirred in a 34W blue LED lamp irradiation with fan cooling for 12 h. The reaction mixture was then diluted with HCl (1 M aq., 5 mL), extracted with CH_2Cl_2 (3 × 5 mL), dried (anhydrous Na_2SO_4), filtered, and concentrated under reduced pressure. The resulting residue was analyzed by crude NMR after the addition of 1,1,2,2-tetrachloroethane (3 μL) as an internal standard. The product yields were compared and the kinetic isotope effects (KIE) were measured to be 1.83.

2.9. Computational Details

General information

All calculations were carried out using DFT as implemented in the Gaussian 09[21] program packages. Gas-phase geometry optimizations were conducted with the B3LYP[22] hybrid functional, including Grimme's D3 dispersion correction[23] and the 6-31G** basis set and LanL2DZ[24,25] basis set for Ni and Ir. The energies of the optimized structures were reevaluated by additional single-point calculations using B3LYP hybrid functional, including Grimme's D3 dispersion correction and the 6-311++G** basis set and the SDD basis set for Ni and Ir. The integral equation formalism variant of the Polarizable Continuum Model (IEFPCM) was employed as implemented to account for the solvation effects for benzene (ε = 2.2706). All thermal corrections from the vibrational frequency calculations were performed at 25 °C (298.15 K).

Computation of Redox Potentials

The standard reduction potentials, E^{o}_{red} were obtained from the electron attachment energy in the solution phase, ΔG^{EA}(sol), and subsequent application of the following relationships, where n is the number of electrons, E is the absolute potential, and F is the Faraday constant. ΔG^{EA}(sol) was computed by subtracting the Gibbs free energies of the oxidized species from those of the reduced species.

$$\Delta G^{EA}(\text{sol}) = -\text{n}\text{F}E \quad (1)$$

$$E^{o}_{red}\ (\text{V vs SCE}) = -E - E^{o}\ (\text{SCE}) = -E - 4.51\ \text{V} \quad (2)$$

Here, we employed the absolute potential measured to be 4.27 V for the standard hydrogen electrode and added 0.24 V to obtain the absolute potential for the saturated calomel electrode.[26,27] The reduction potential of species denoted as "**2aa-lut**", and nickel-bound species "**^{3}IV-2aa**" was computed accordingly (Supplementary Fig. 58).

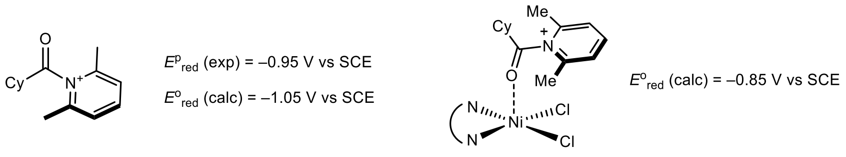

2aa-lut **^{3}IV-2aa**

Supplementary Fig. 58. Comparison of experimental and computed reduction potentials.

Computation of Activation Barrier of Single Electron Transfer Step

The redox properties of nickel(II) catalyst (**^{3}I**) and N-acyllutidinium (**2aa-lut**) under photoredox conditions were calculated according to the Marcus theory of electron transfer.[28-30]

$$\Delta G^{\ddagger} = \frac{\lambda}{4}\left(1 + \frac{\Delta G_{\text{rel}}}{\lambda}\right)^{2} \quad (3)$$

The reorganization energy λ is comprised of the internal reorganization energy λ_i and the external reorganization energy λ_o. In calculation of reorganization energy, the internal reorganization energy λ_i has more significant influence especially in solvents with low static dielectric constants. Thus, the equation can be reduced to following equation.

$$\Delta G^{\ddagger} = \frac{\lambda_i}{4}\left(1 + \frac{\Delta G_{\text{rel}}}{\lambda_i}\right)^{2} \quad (4)$$

The internal reorganization energy is calculated as follows:

$$\lambda_{\text{i}} = \frac{\lambda_i(\text{D}) + \lambda_i(\text{A})}{2} \quad (5)$$

where $\lambda_i(\mathrm{D})$ or $\lambda_\mathrm{i}(\mathrm{A})$ represents the internal reorganization energy for the electron donor or acceptor. The internal reorganization energy λ_i for each reaction component is calculated using Nelsen's four-point method,[31] where E_S is the electron configuration of starting material, E_P is the electron configuration of product, R_S is the optimized geometry of the starting material, and R_P is the optimized geometry of the product.

$$\lambda_i = \left(\mathrm{E_S}(\mathrm{R_P}) - \mathrm{E_S}(\mathrm{R_S})\right) + \left(\mathrm{E_P}(\mathrm{R_S}) - \mathrm{E_P}(\mathrm{R_P})\right) \quad (6)$$

Supplementary Table 2. Estimation of the Activation Barriers for the Single Electron Transfer Processes.

	**λ/(kcal/mol)**	**ΔG_{rel}/(kcal/mol)**	**Δ$G^‡$/(kcal/mol)**
**I-TS**	15.6	21.2	21.7
**II-TS**	21.5	4.3	7.8
**IV-TS**	18.2	-21.2	0.1
**VII-TS**	4.9	-17.0	7.4
**VIII-TS**	12.3	-21.0	1.5
**I-TS**	15.6	21.2	21.7

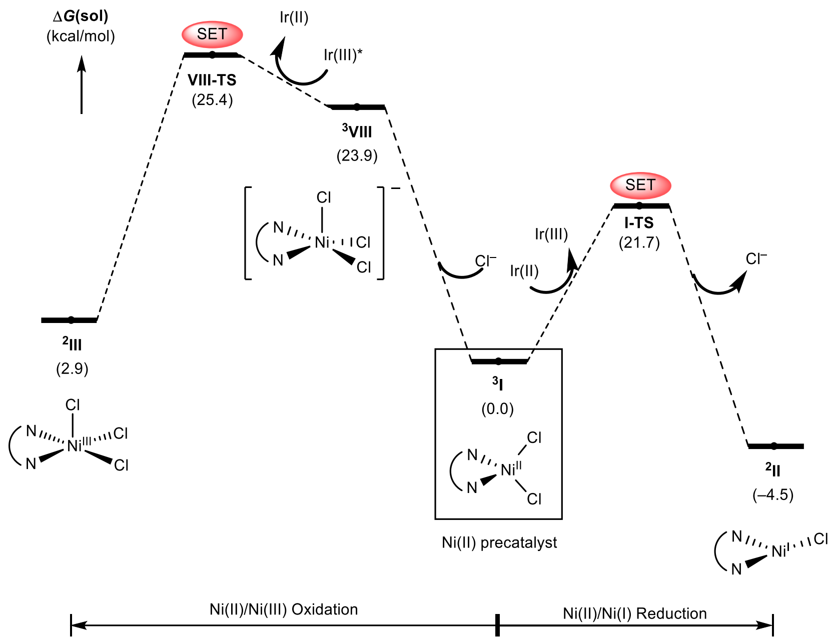

Supplementary Fig. 59. Comparison of computed nickel(II) redox free energy profile.

Compared to the N-acyllutidinium-mediated reduction-initiated-pathway which is nearly barrierless (0.7 kcal/mol), the redox processes of the initial nickel precatalyst (^{3}**I**) showed much higher reaction barriers (C–H addition initiated pathway: 25.4 kcal/mol, oxidative addition initiated pathway: 21.7 kcal/mol).

Free Energy Profile for Oxidative-Addition-Initiated Pathway

The initial free energy profile for the oxidative-addition-initiated pathway is shown in Supplementary Fig. 60.

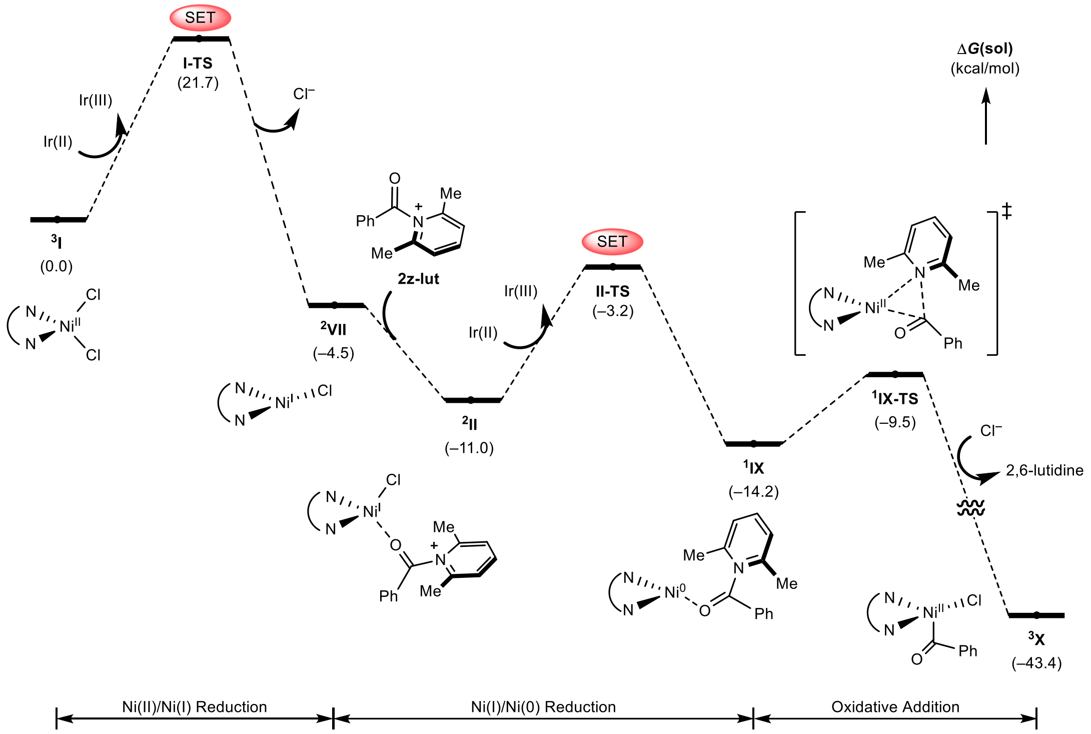

Supplementary Fig. 60. Free energy profile of oxidative addition pathway.

Supplementary Table 3. Energy Components of DFT Optimized Structures

	E(SCF)/(Hartree)	Thermal Corr. to *G*	*G*(sol)/(kcal/mol)
	6-311++G**/SDD	6-31G**/LanL2DZ	
cyclohexane	-235.9579039	0.142273	-147976.4307
2,6-lutidine	-327.0251066	0.110368	-205141.9407
2,6-lutidine•HCl	-787.8925306	0.118297	-494335.4216
2aa-lut	-675.5857832	0.275513	-423763.2724
2aa-lut-red	-675.711016	0.273569	-423843.0769
^{3}IV-2aa	-2577.360854	0.632756	-1616920.072
^{3}IV-2aa-red	-2577.493311	0.6308	-1617004.417
2z	-805.3202439	0.068085	-505302.977
2z-lut	-671.9230316	0.20653	-421508.1502
2z-lut-red	-672.0783228	0.204564	-421606.8305
3z	-580.439301	0.223013	-364090.9427
^{3}I	-1901.743393	0.329884	-1193154.089
^{2}II	-2113.410087	0.560352	-1325832.225
^{3}III	-2361.944014	0.332078	-1481932.744
^{3}IV	-2573.689124	0.559964	-1614661.706
^{2}V	-2246.802614	0.420019	-1409625.296
^{2}VI	-2021.863624	0.576651	-1268375.767
^{2}VI-TS	-2021.851175	0.578027	-1268367.092
^{2}VII	-1441.453372	0.330606	-904317.5055
^{3}VIII	-2362.122499	0.327901	-1482047.366
^{1}IX	-1653.124133	0.567115	-1036994.402
^{1}IX-TS	-1653.114771	0.565367	-1036989.624
^{3}X	-1786.52903	0.421124	-1120798.786

3. Supplementary Notes

3.1. ¹H, ¹³C, and ¹⁹F NMR Spectra

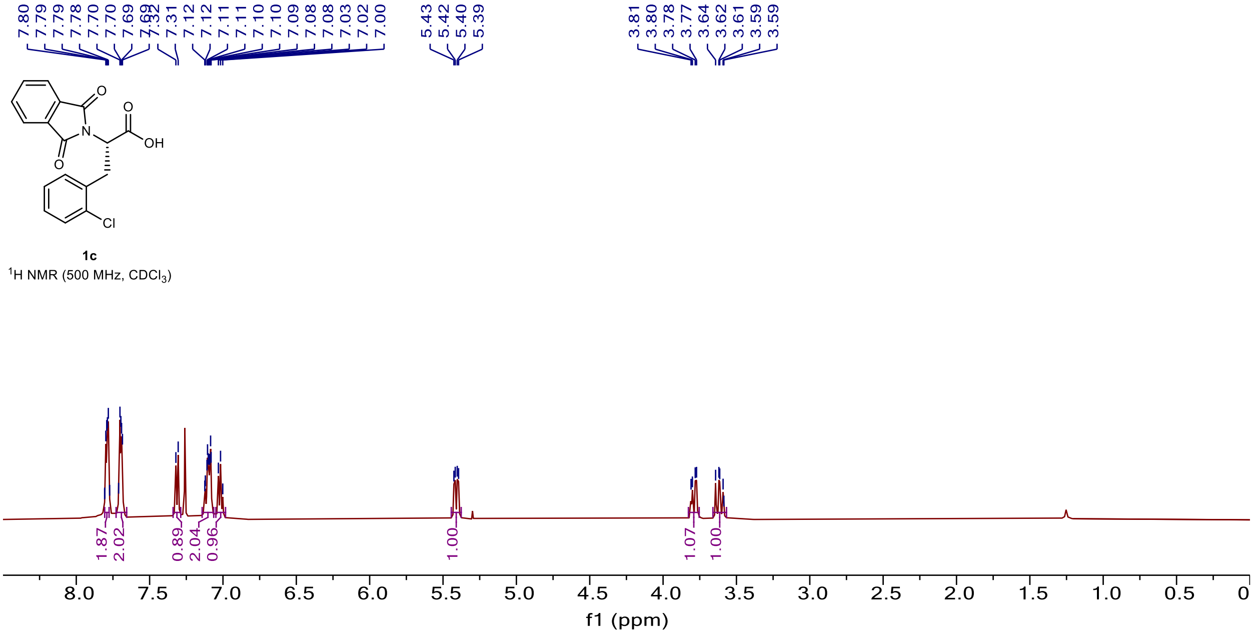

Supplementary Fig. 61. ¹H NMR spectrum of compound 1c.

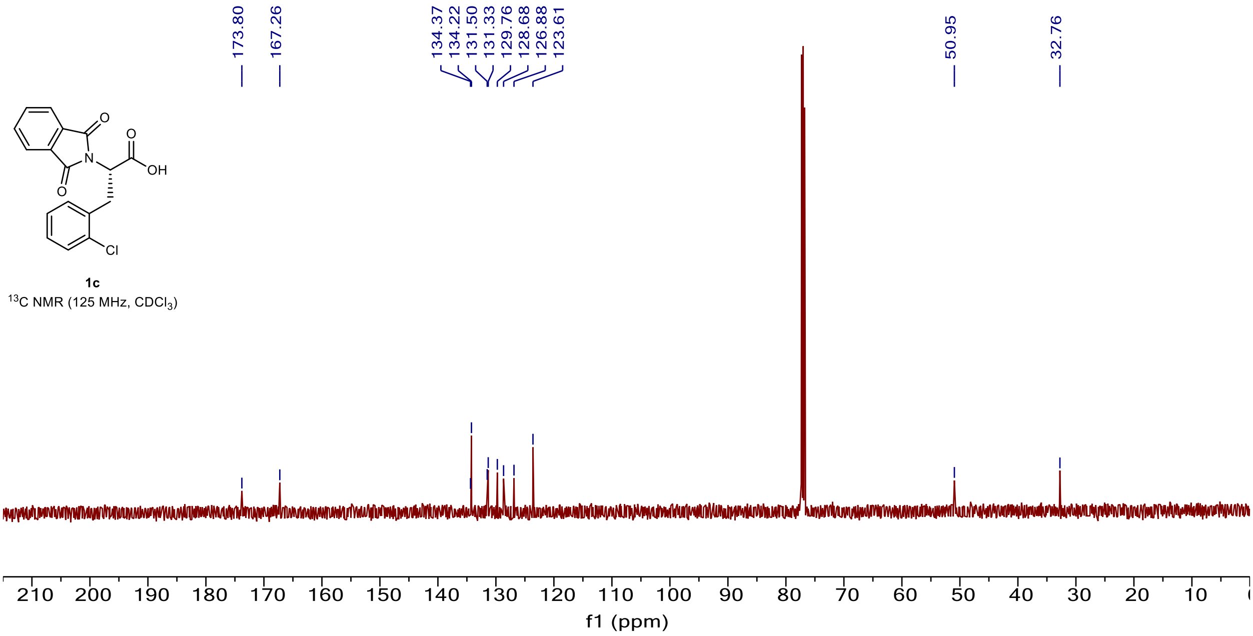

Supplementary Fig. 62. ¹³C NMR spectrum of compound 1c.

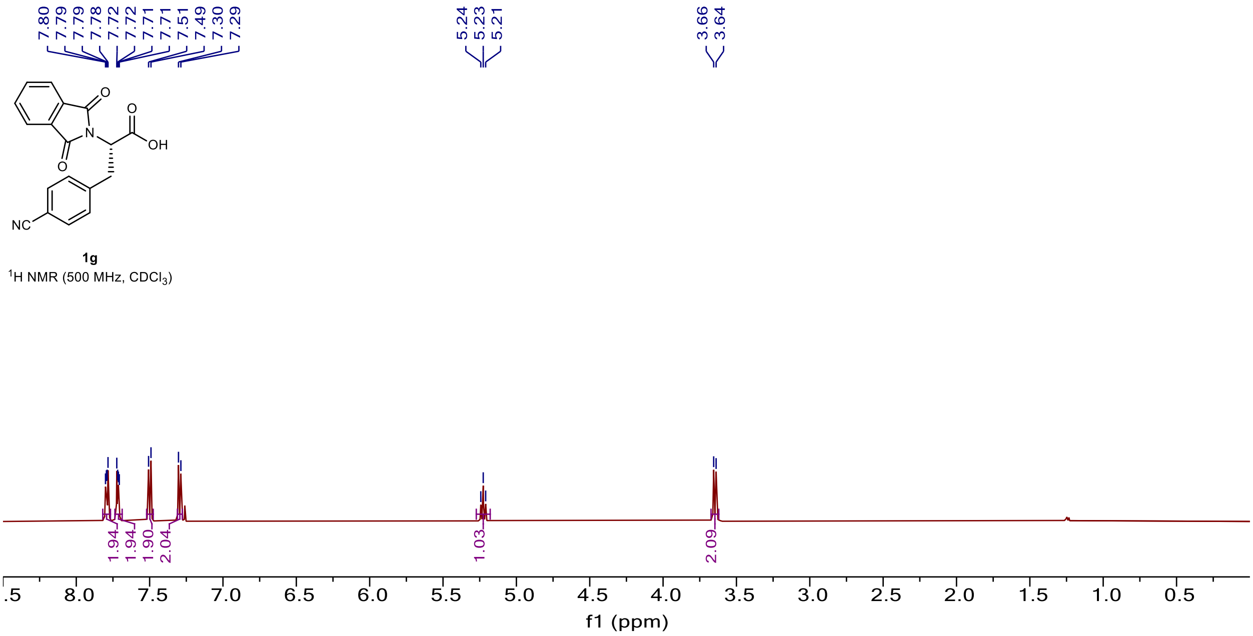

Supplementary Fig. 63. ^{1}H NMR spectrum of compound 1g.

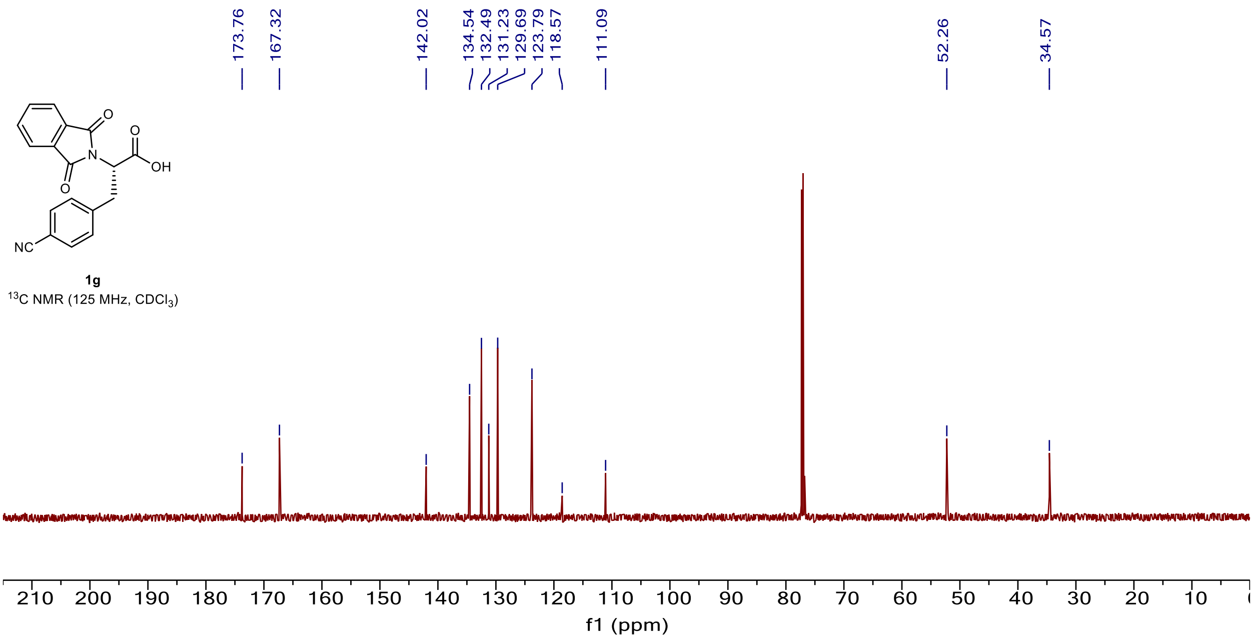

Supplementary Fig. 64. ^{13}C NMR spectrum of compound 1g.

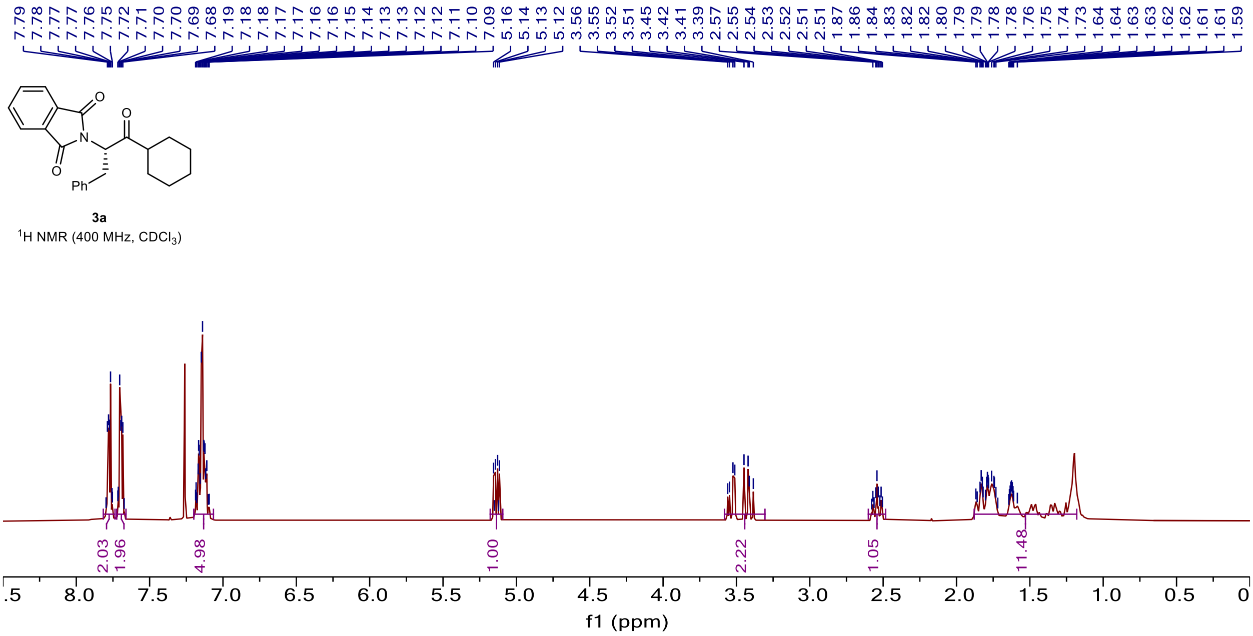

Supplementary Fig. 65. ^{1}H NMR spectrum of compound 3a.

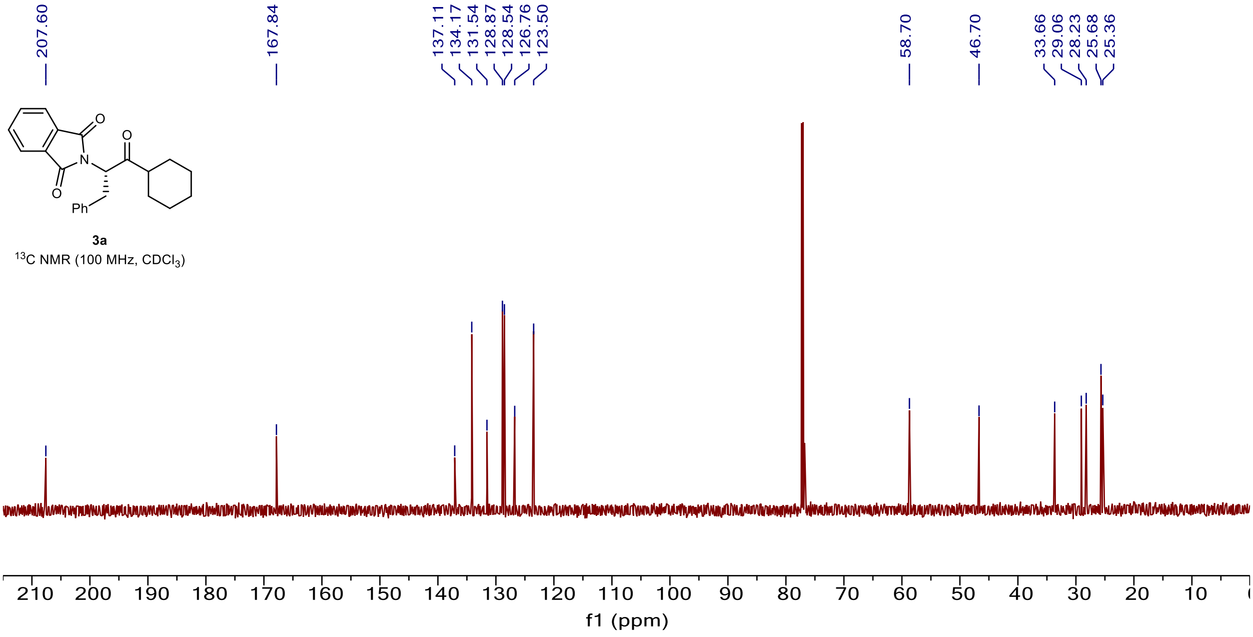

Supplementary Fig. 66. ^{13}C NMR spectrum of compound 3a.

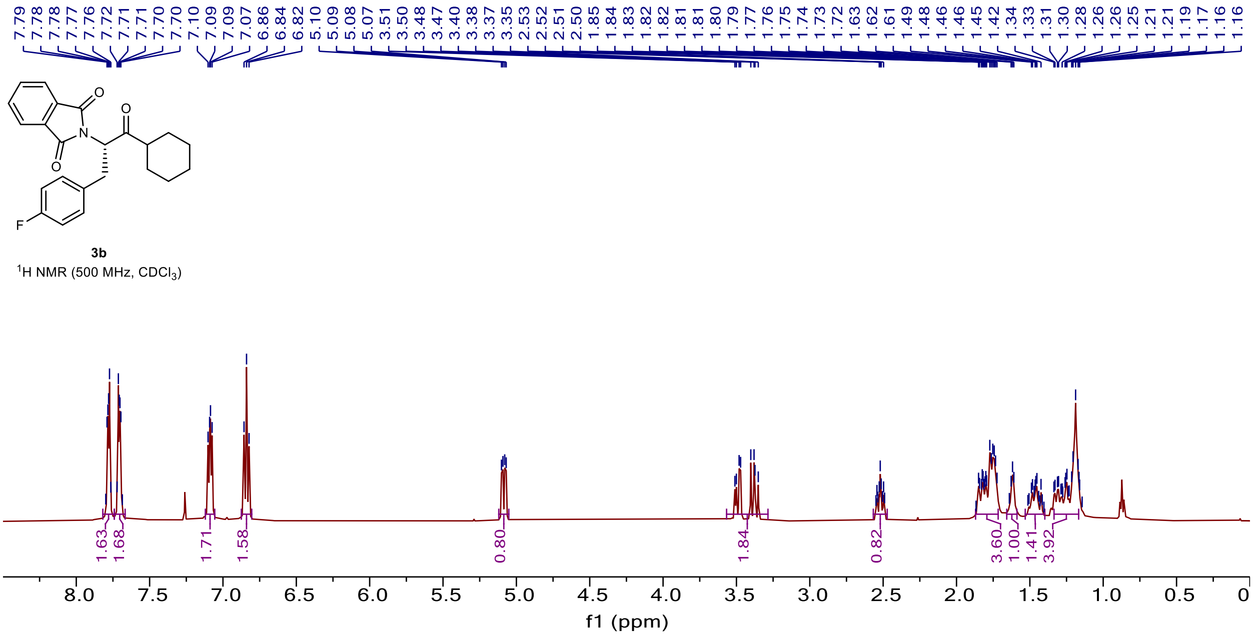

Supplementary Fig. 67. ^{1}H NMR spectrum of compound 3b.

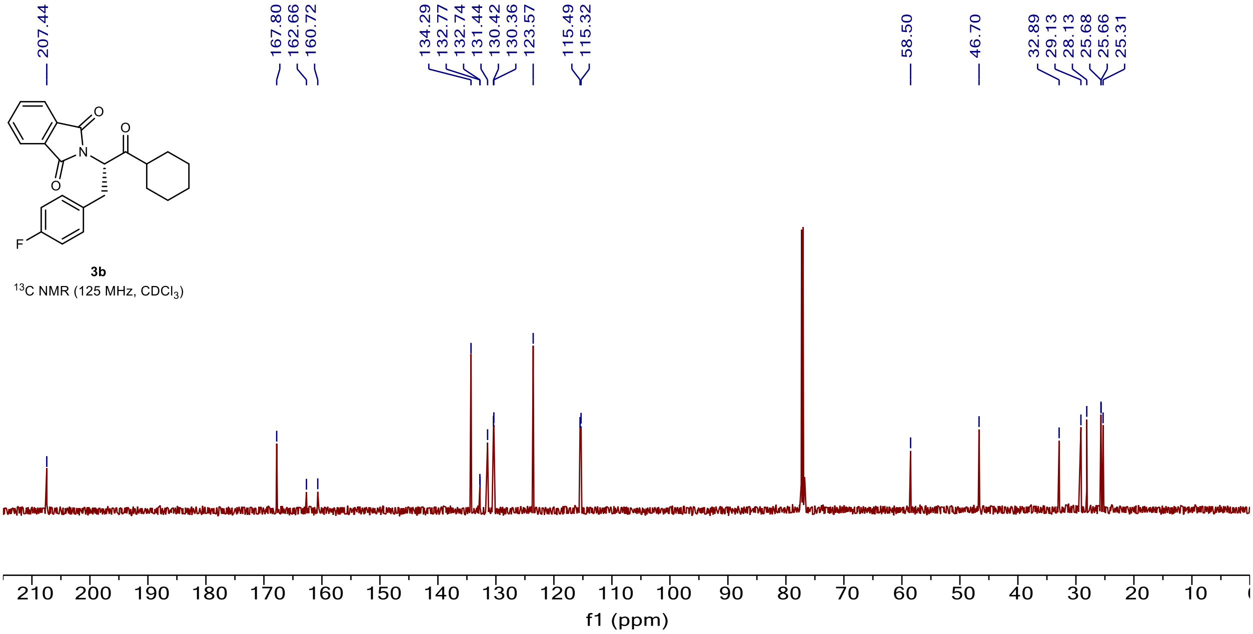

Supplementary Fig. 68. ^{13}C NMR spectrum of compound 3b.

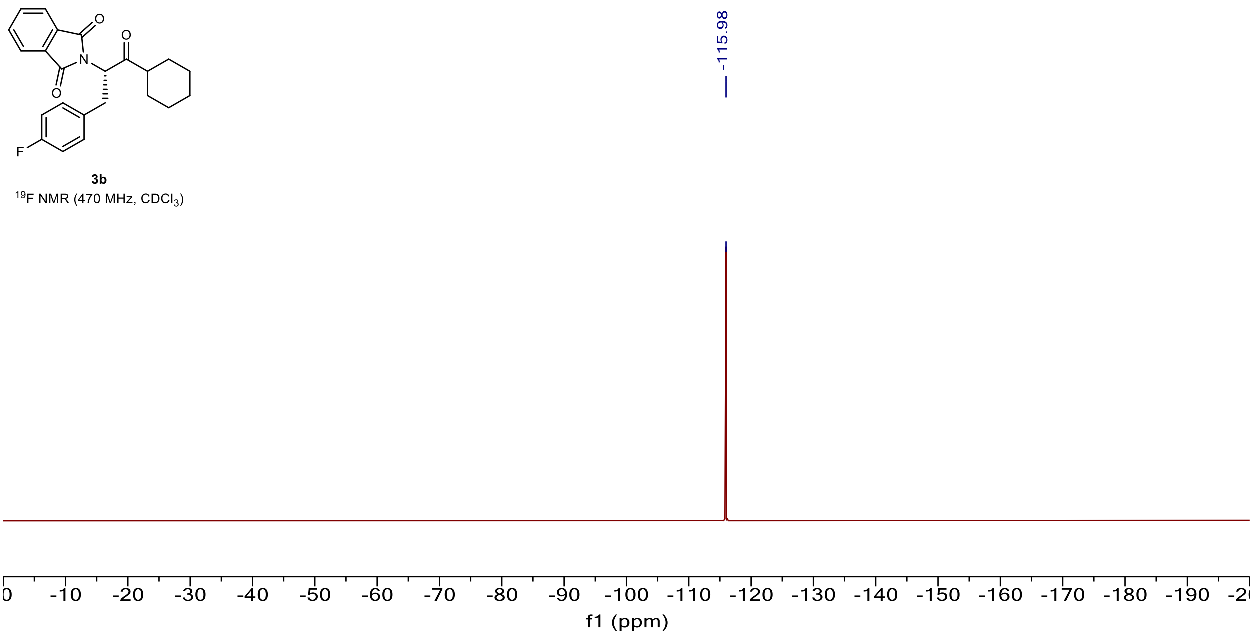

Supplementary Fig. 69. ^{19}F NMR spectrum of compound 3b.

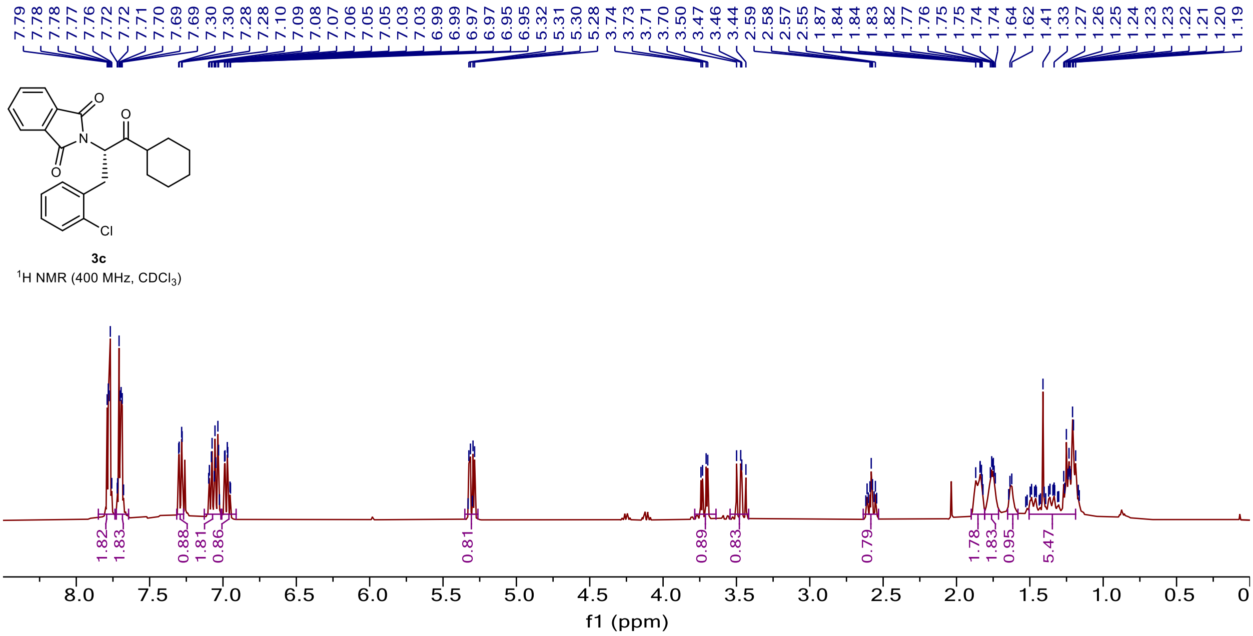

Supplementary Fig. 70. ^{1}H NMR spectrum of compound 3c.

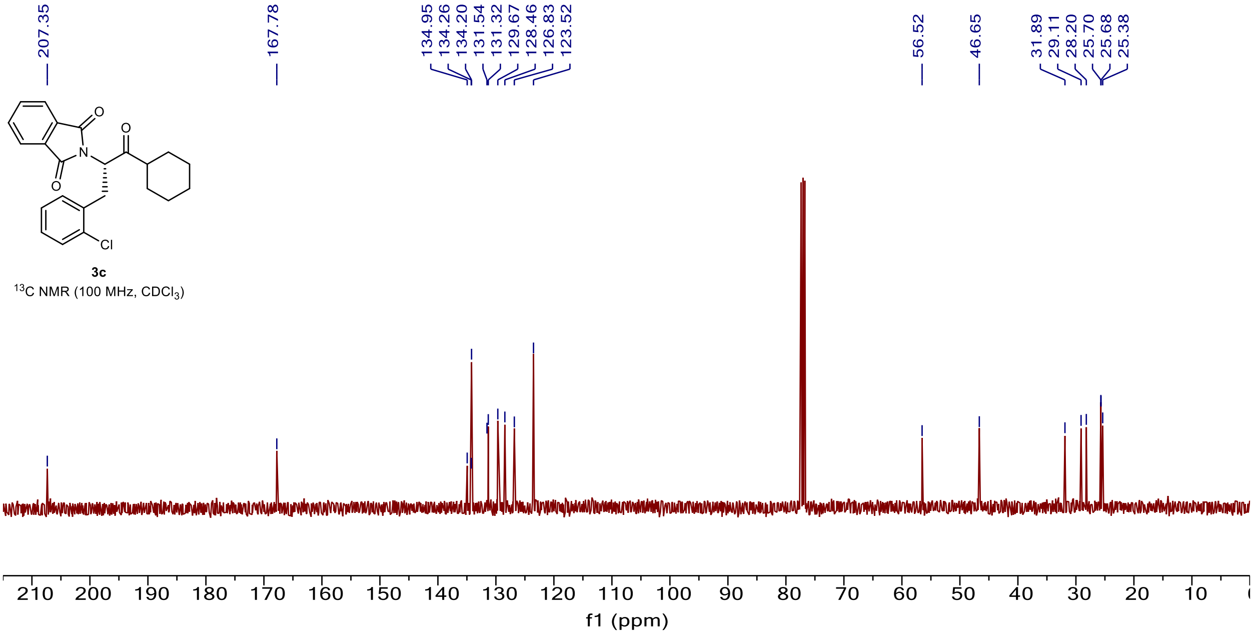

Supplementary Fig. 71. ^{13}C NMR spectrum of compound 3c.

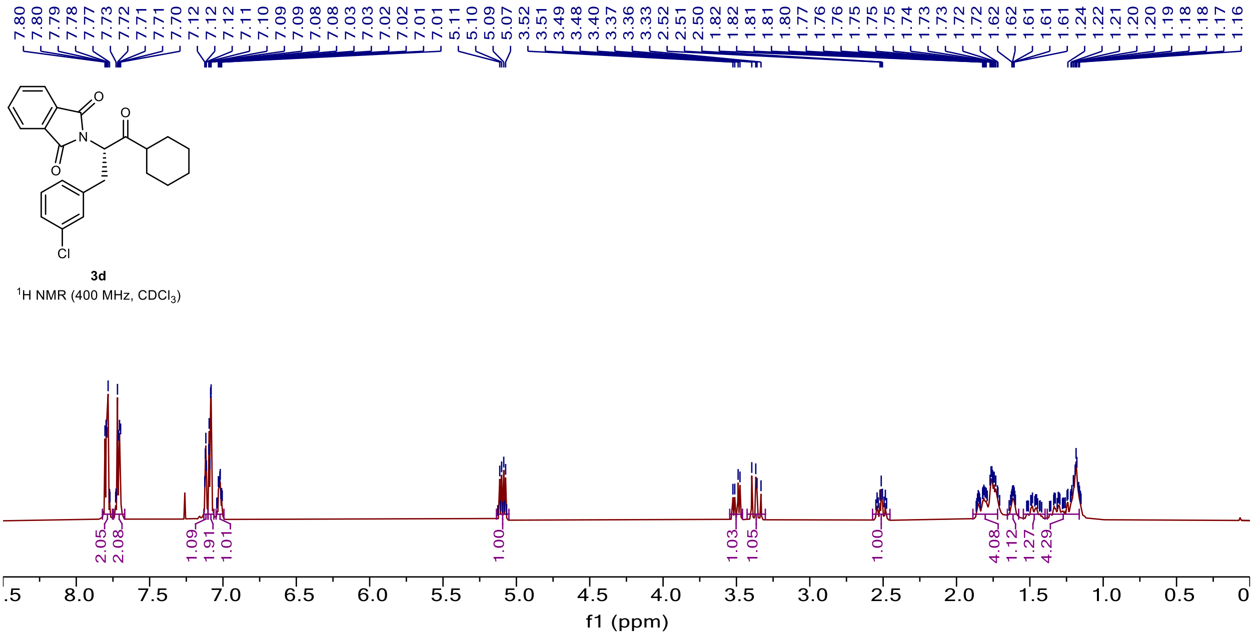

Supplementary Fig. 72. ^{1}H NMR spectrum of compound 3d.

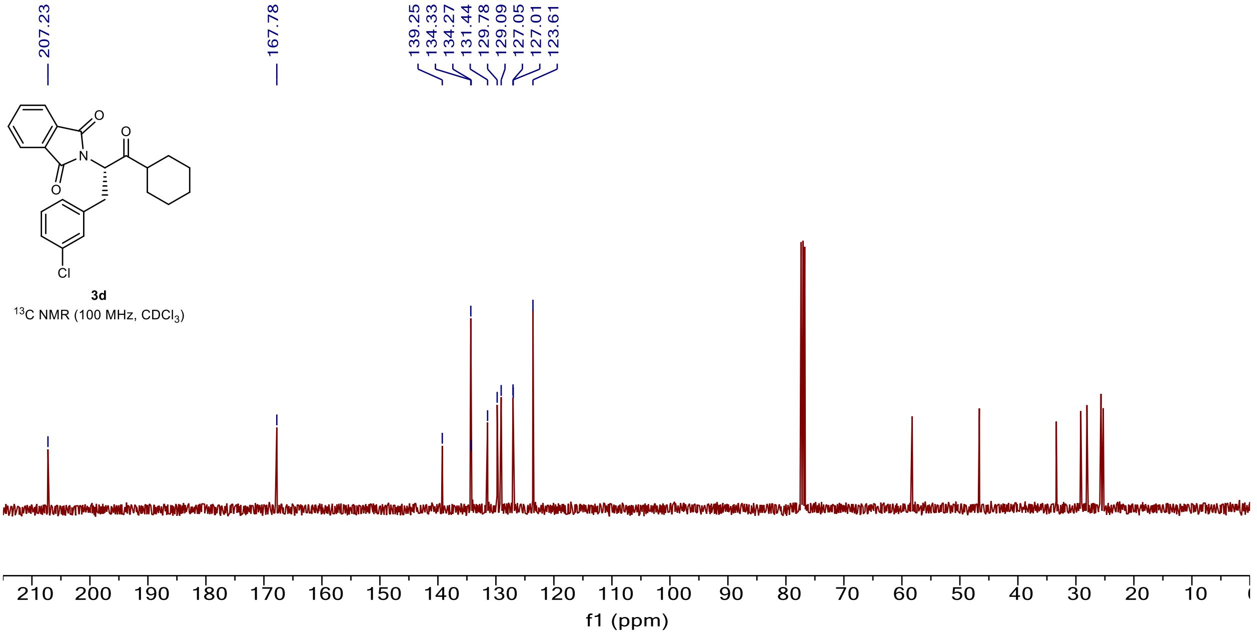

Supplementary Fig. 73. ^{13}C NMR spectrum of compound 3d.

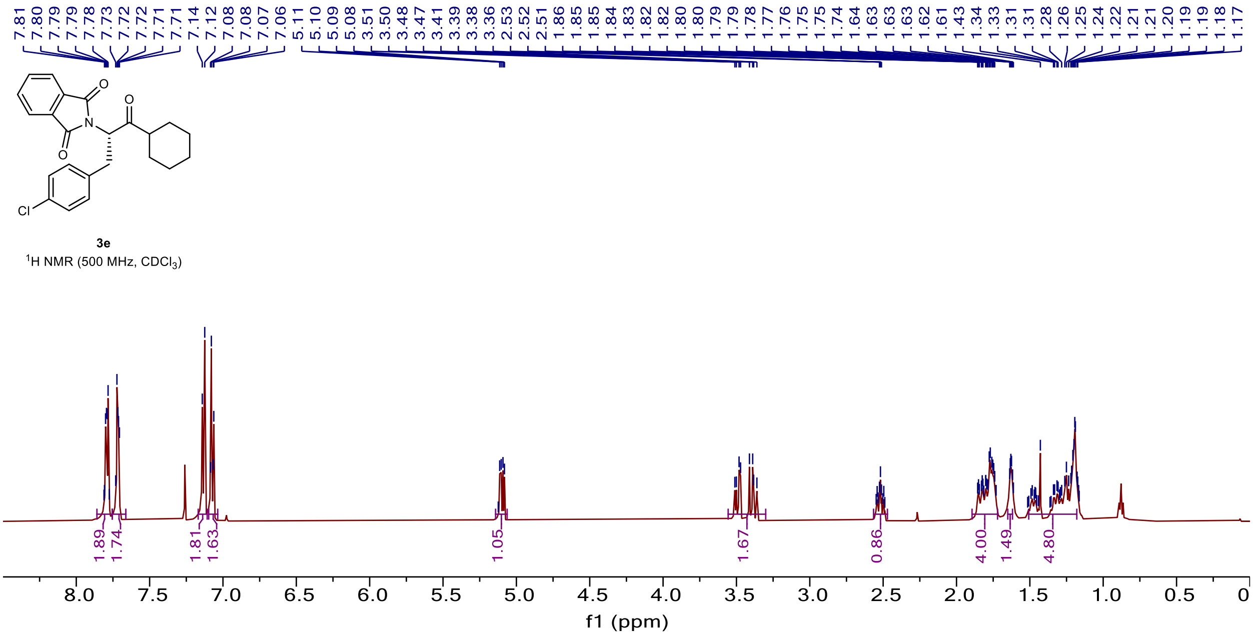

Supplementary Fig. 74. ^{1}H NMR spectrum of compound 3e.

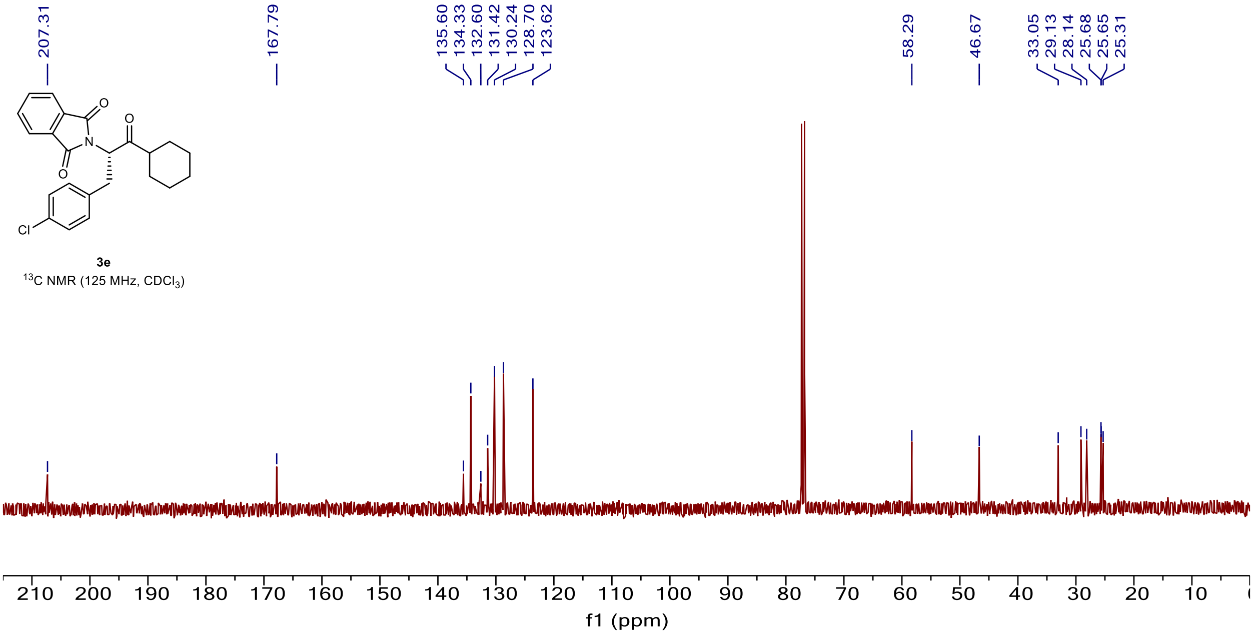

Supplementary Fig. 75. ^{13}C NMR spectrum of compound 3e.

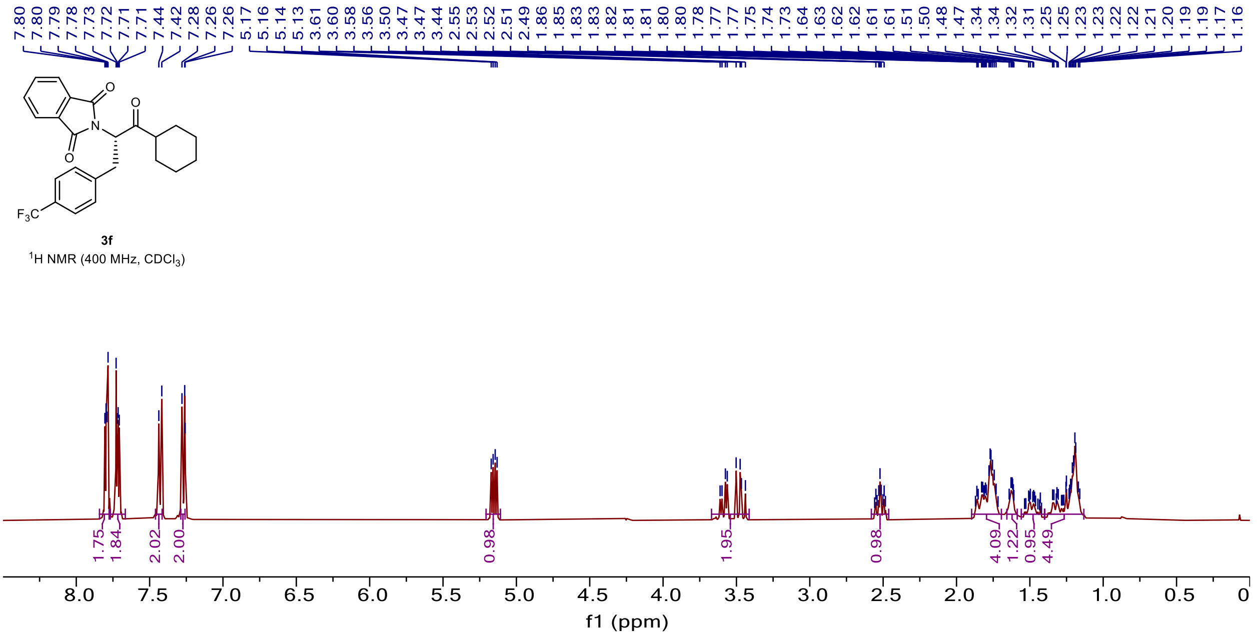

Supplementary Fig. 76. ^{1}H NMR spectrum of compound 3f.

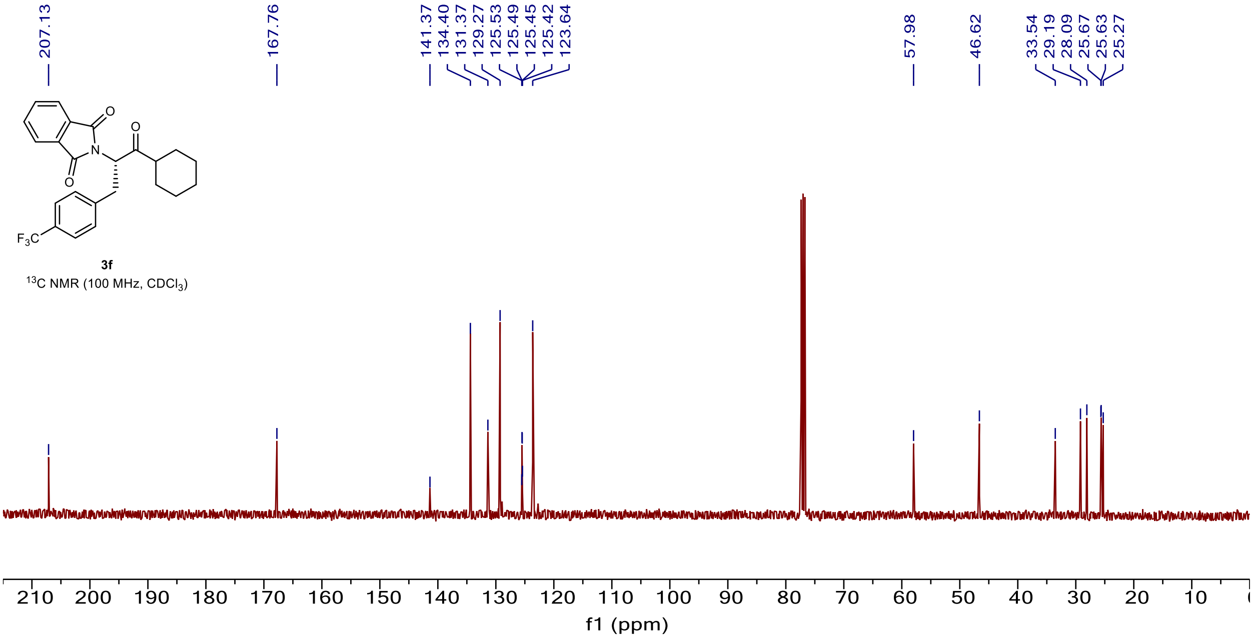

Supplementary Fig. 77. ^{13}C NMR spectrum of compound 3f.

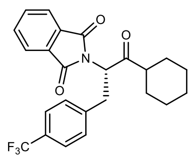

3f

^{19}F NMR (375 MHz, $CDCl_3$)

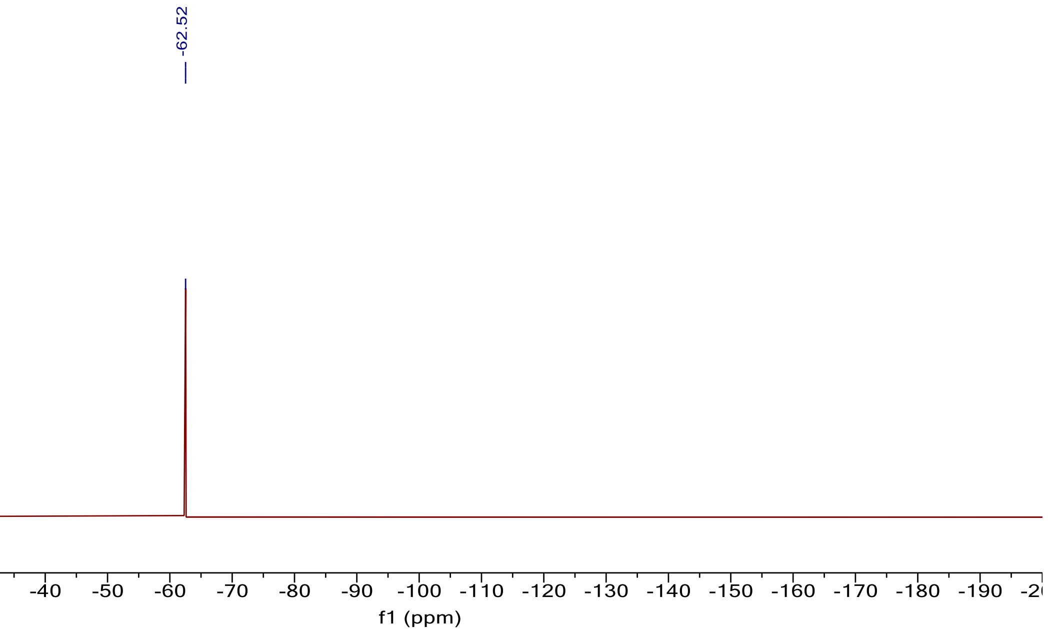

Supplementary Fig. 78. ^{19}F NMR spectrum of compound 3f.

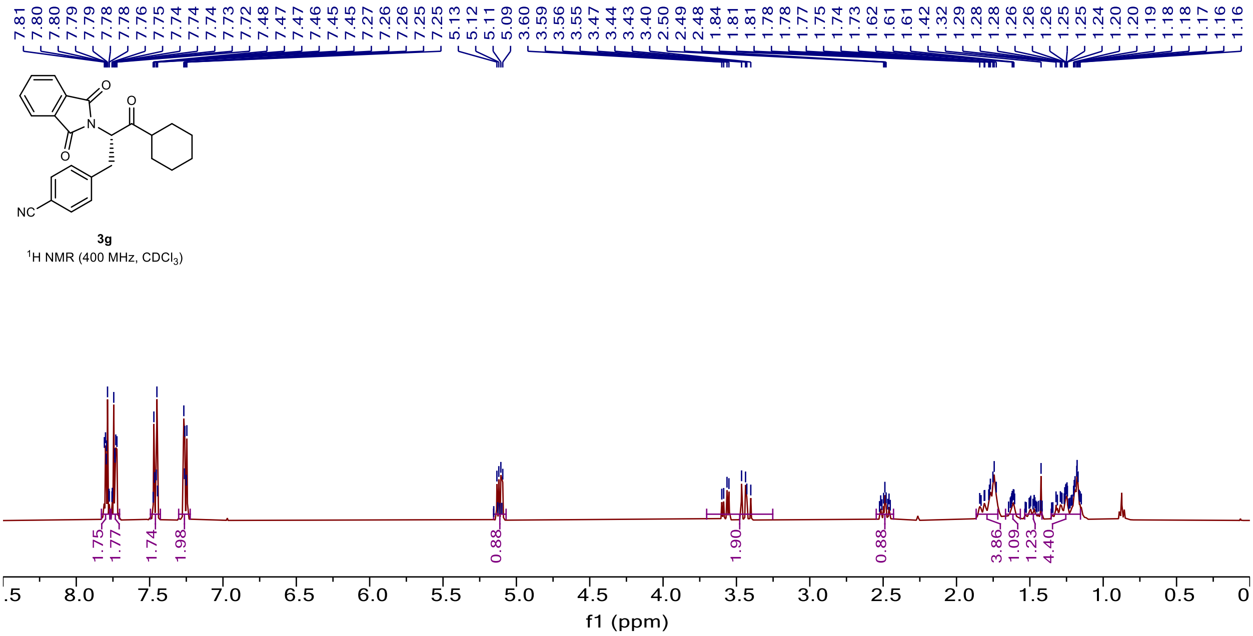

Supplementary Fig. 79. ^{1}**H NMR spectrum of compound 3g.**

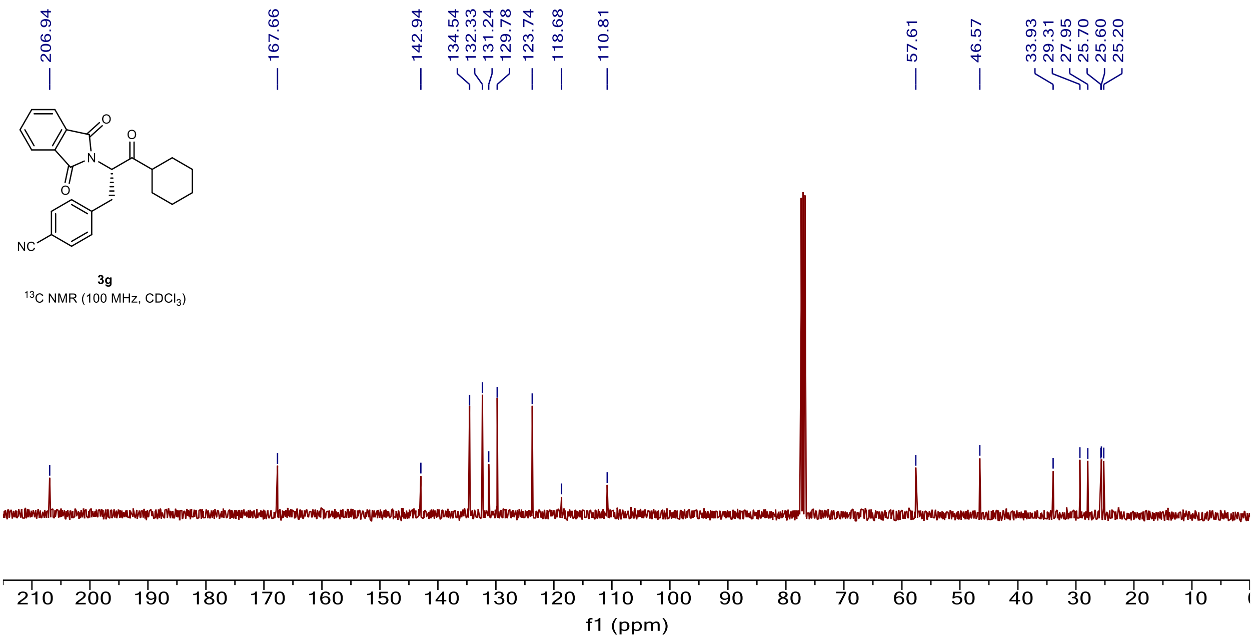

Supplementary Fig. 80. ^{13}**C NMR spectrum of compound 3g.**

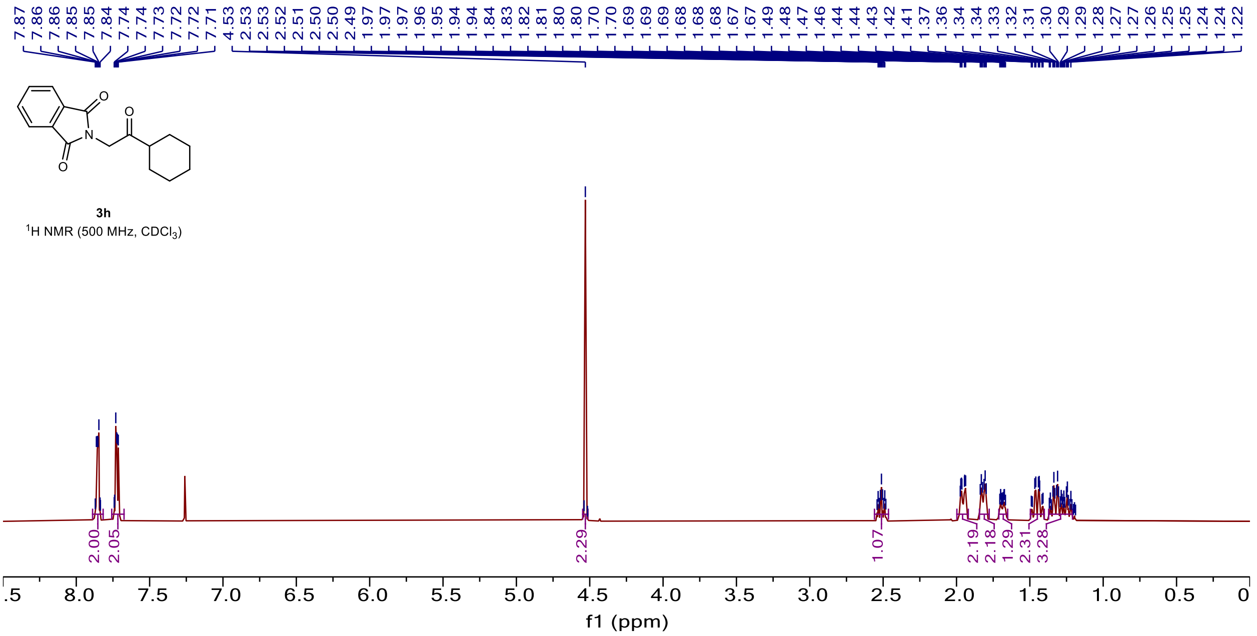

Supplementary Fig. 81. ^{1}H NMR spectrum of compound 3h.

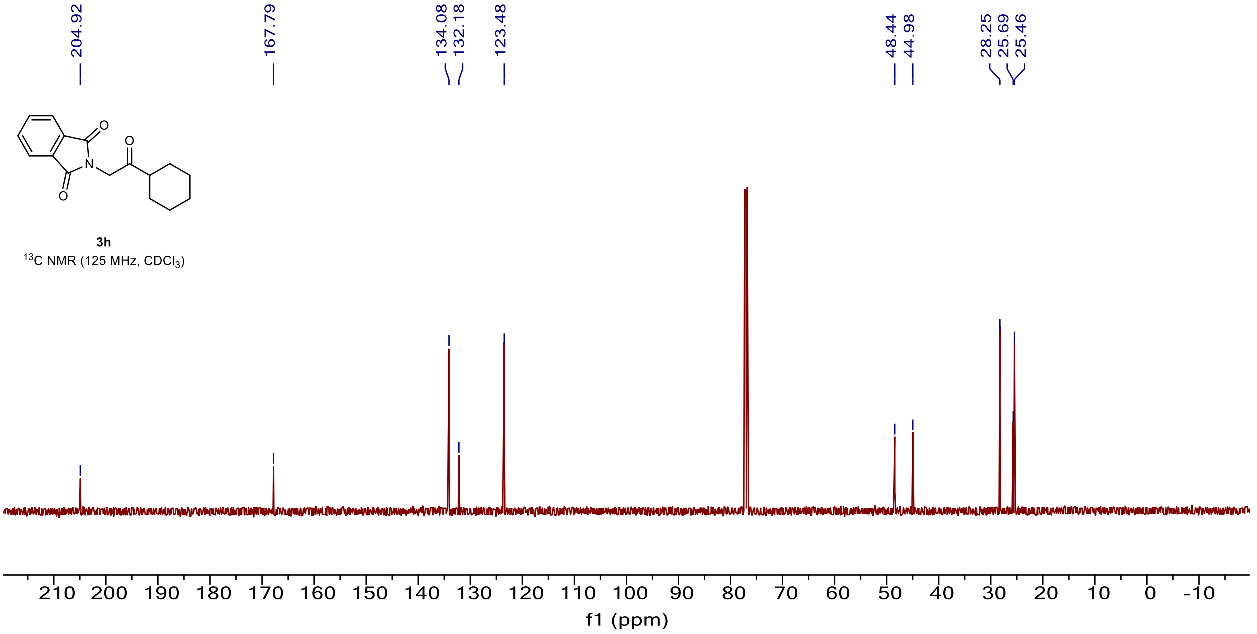

Supplementary Fig. 82. ^{13}C NMR spectrum of compound 3h.

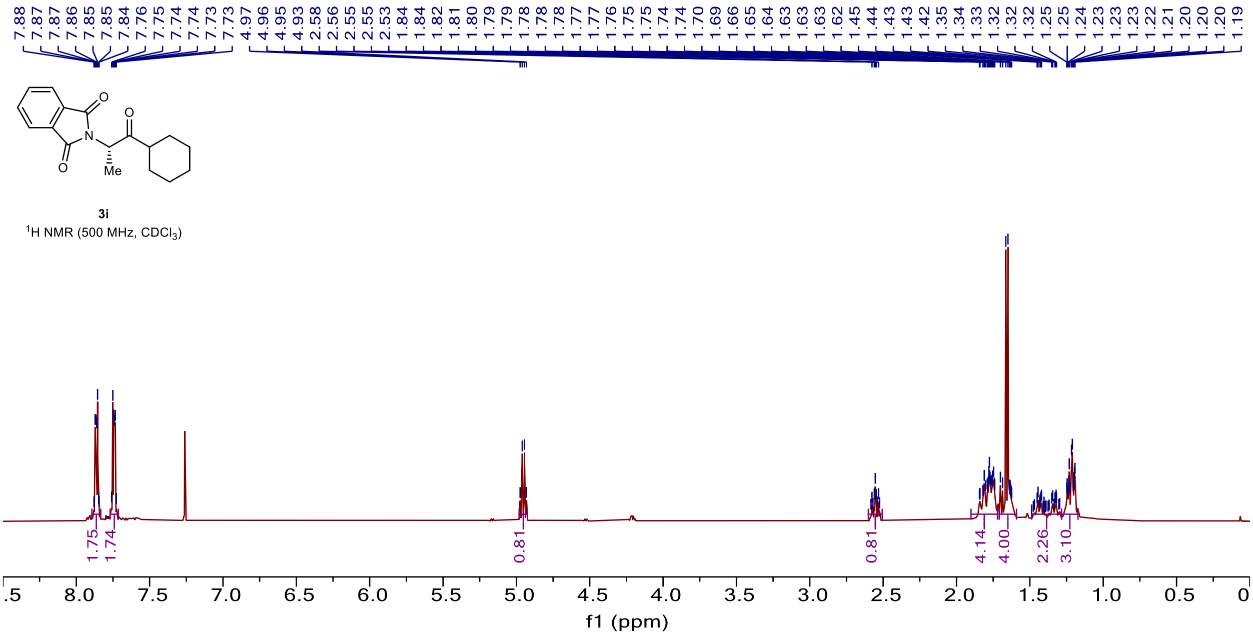

Supplementary Fig. 83. ^{1}H NMR spectrum of compound 3i.

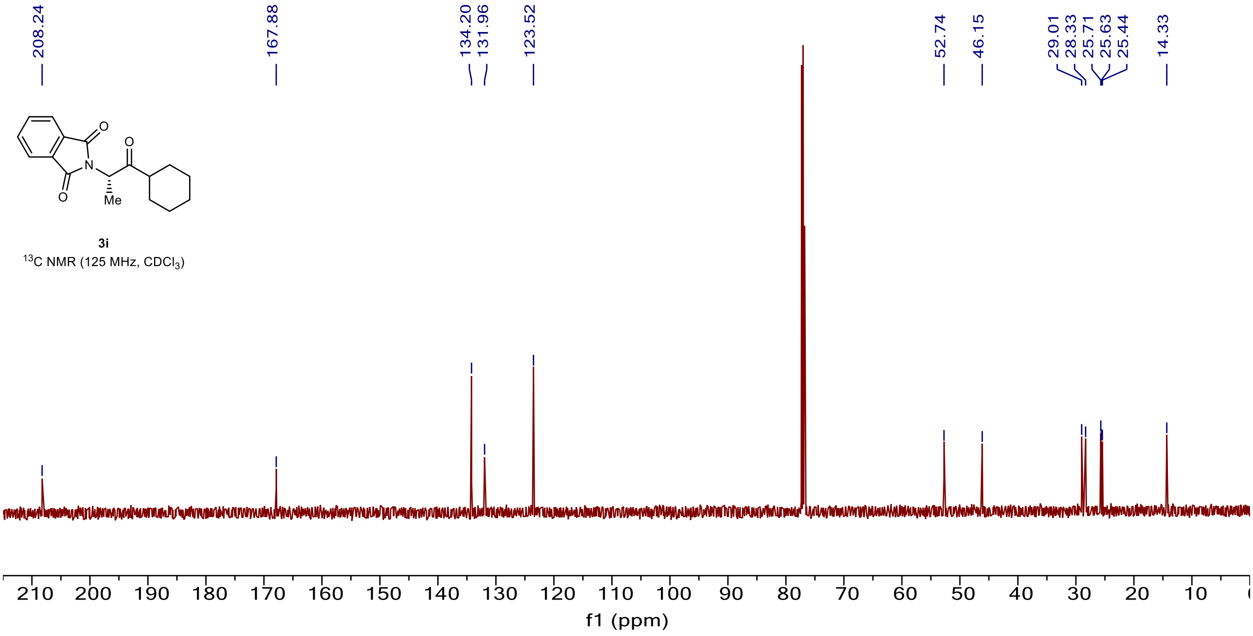

Supplementary Fig. 84. ^{13}C NMR spectrum of compound 3i.

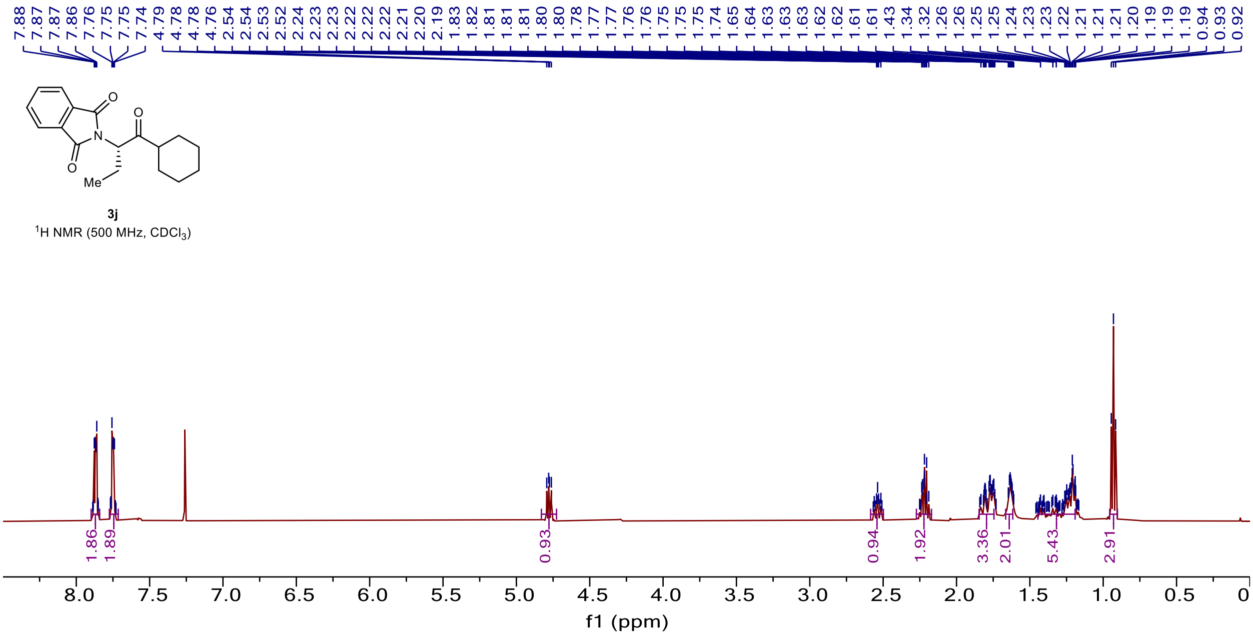

Supplementary Fig. 85. ^{1}H NMR spectrum of compound 3j.

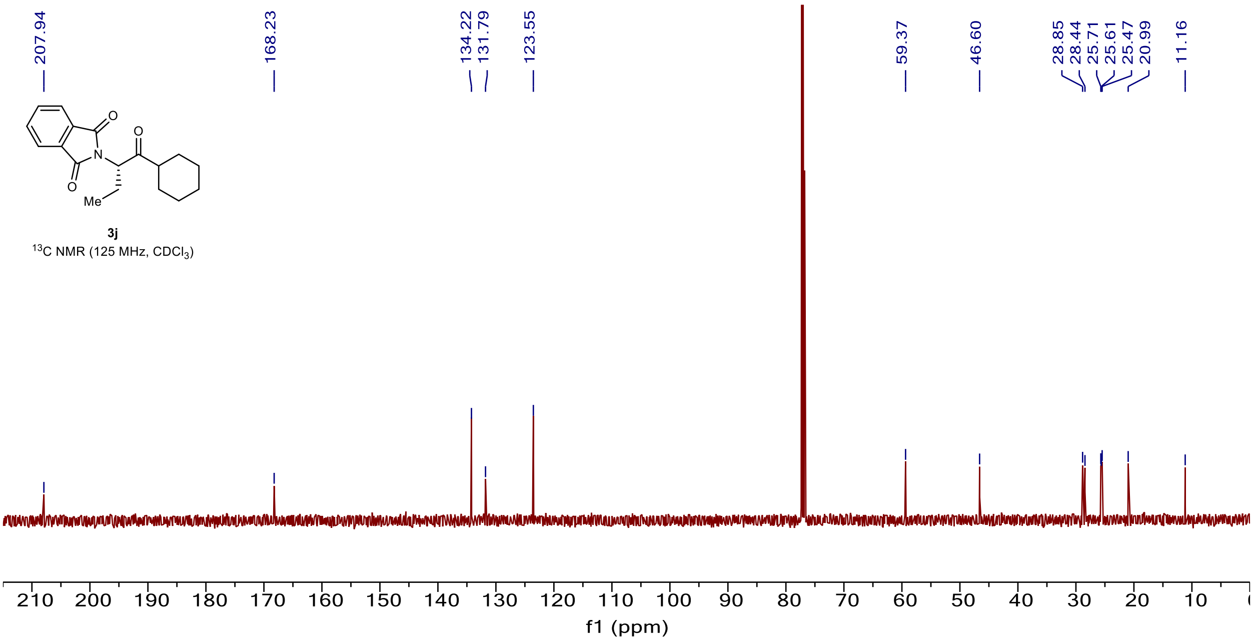

Supplementary Fig. 86. ^{13}C NMR spectrum of compound 3j.

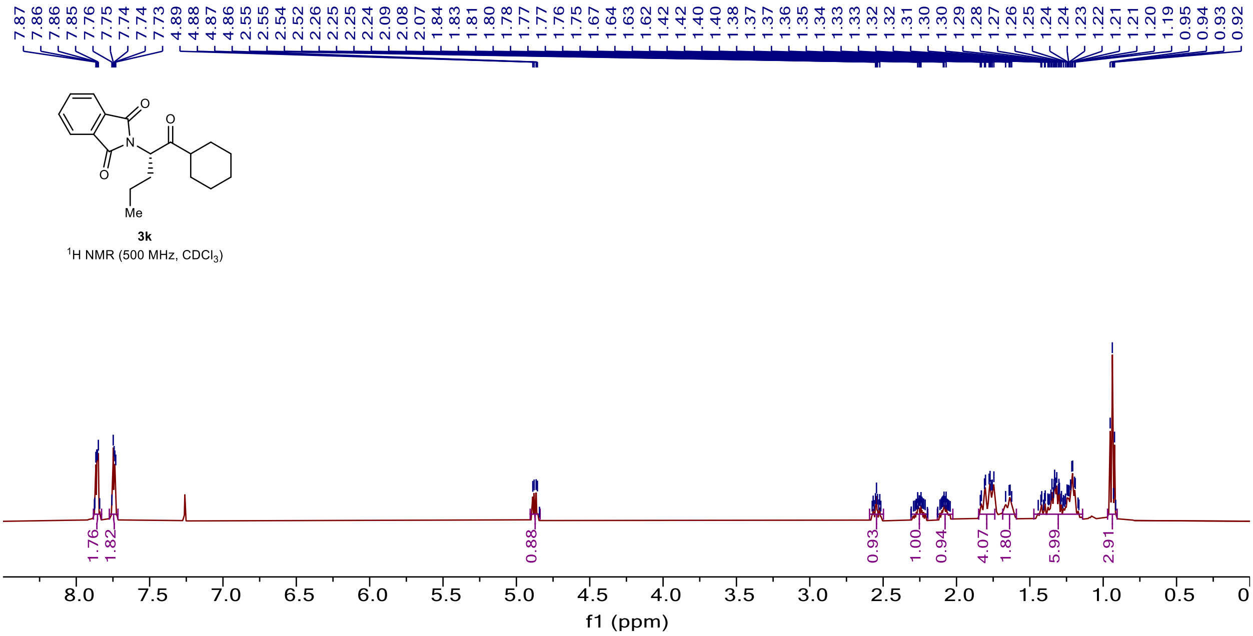

Supplementary Fig. 87. ^{1}H NMR spectrum of compound 3k.

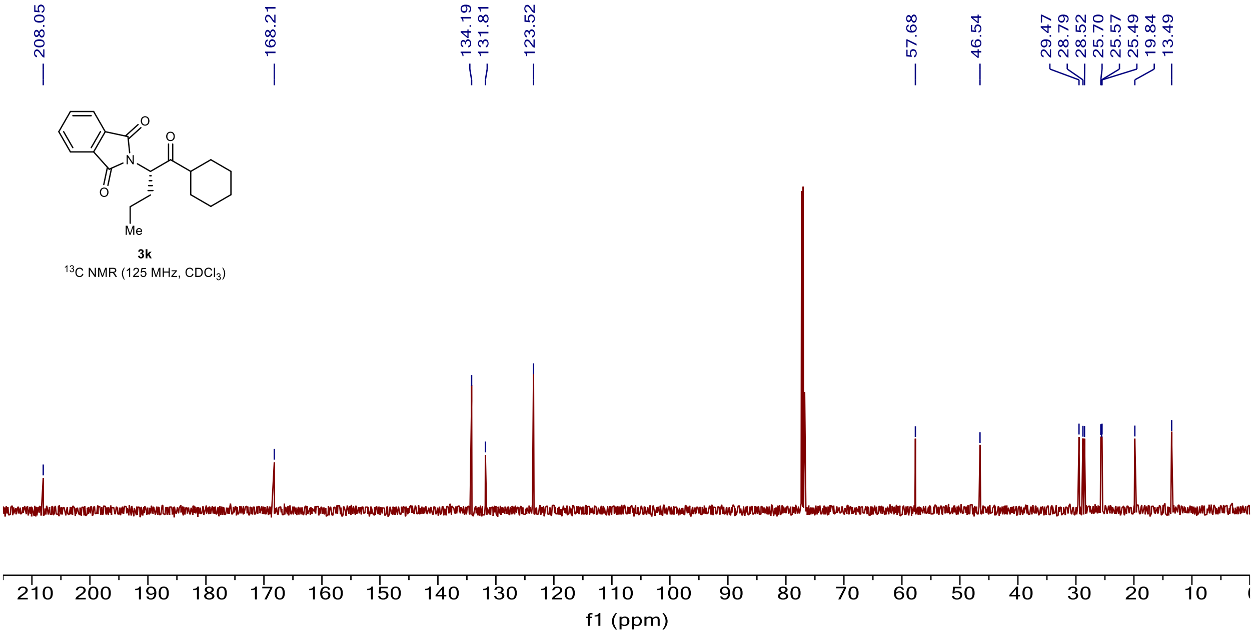

Supplementary Fig. 88. ^{13}C NMR spectrum of compound 3k.

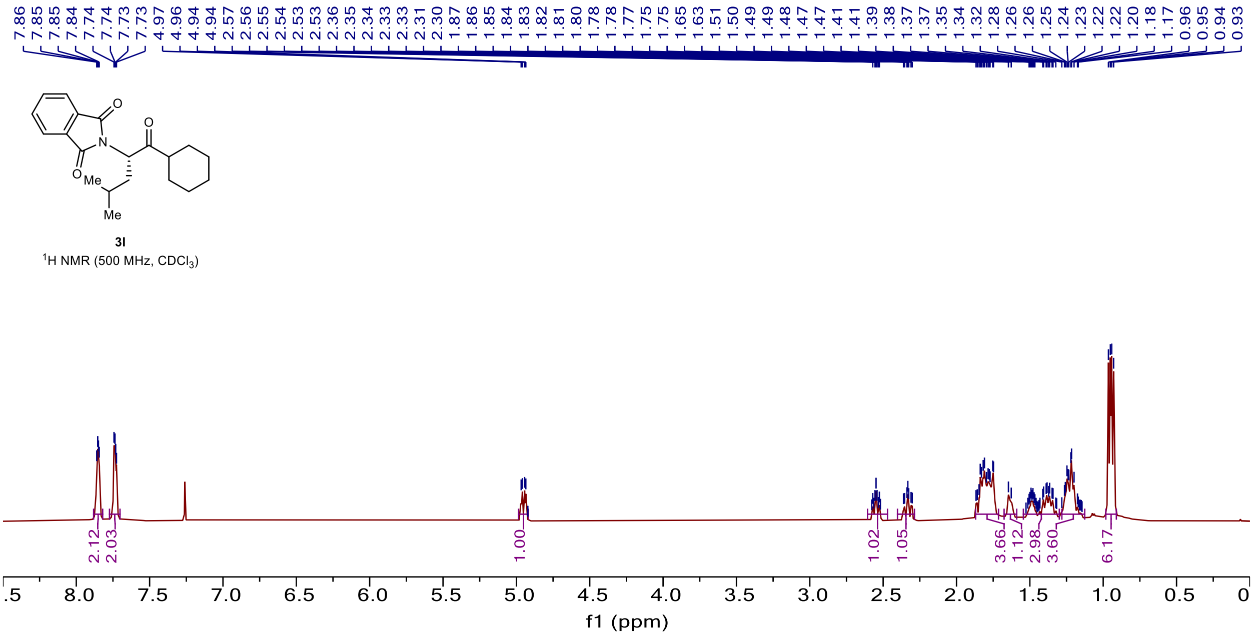

Supplementary Fig. 89. ^{1}H NMR spectrum of compound 3l.

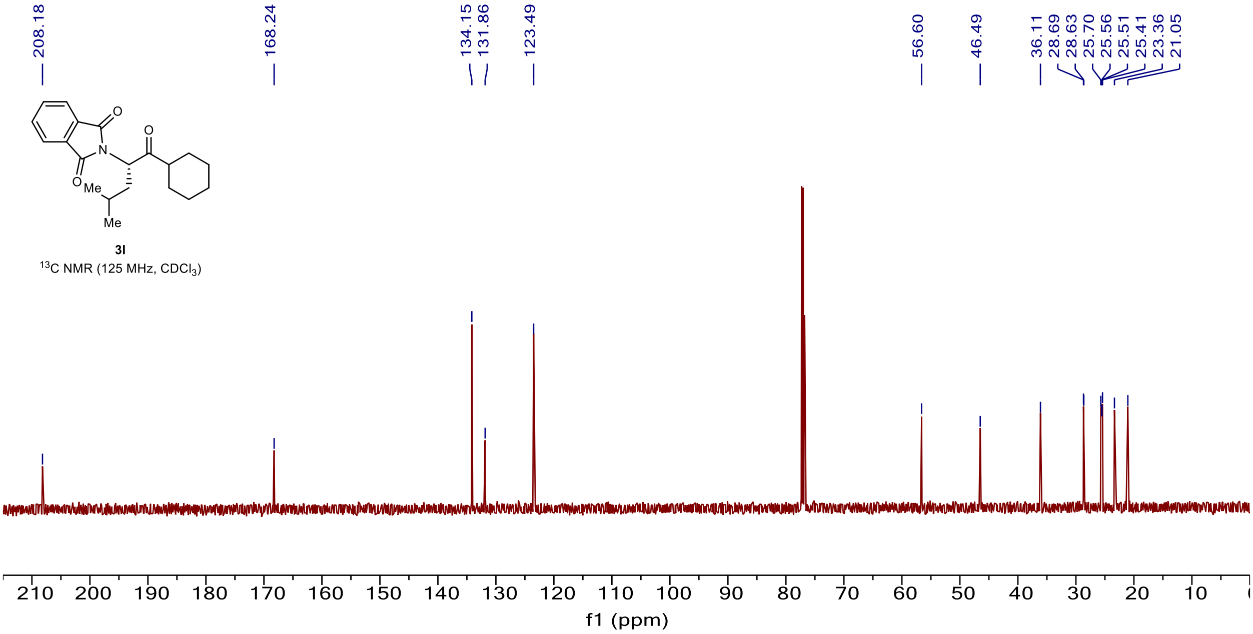

Supplementary Fig. 90. ^{13}C NMR spectrum of compound 3l.

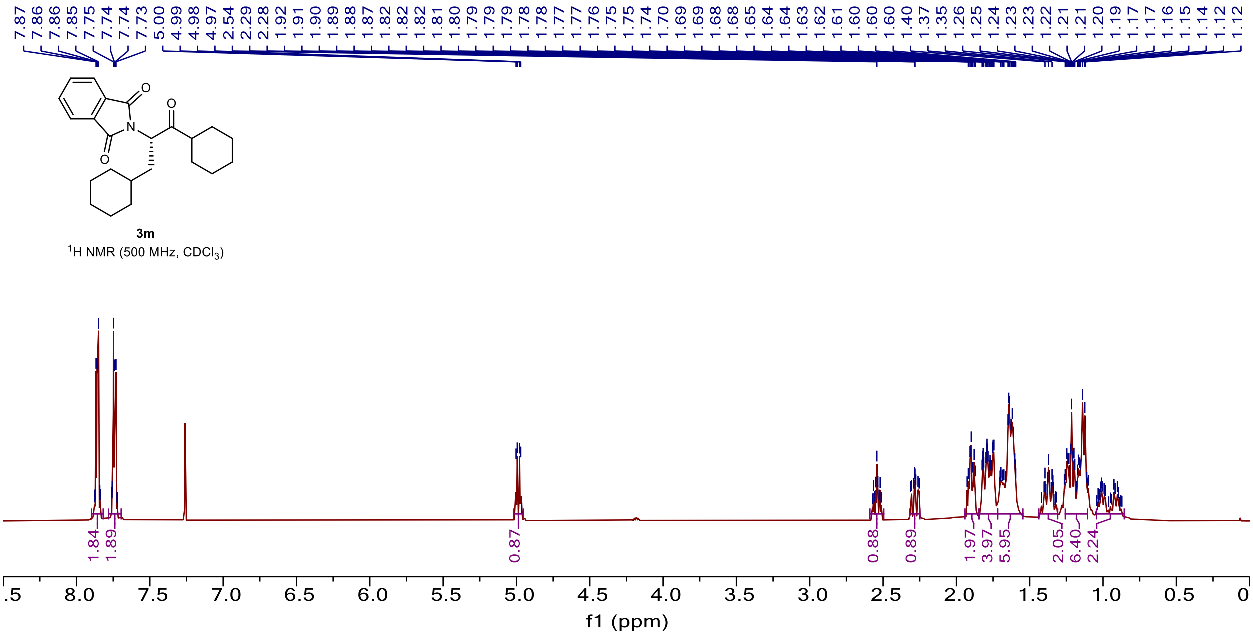

Supplementary Fig. 91. ^{1}H NMR spectrum of compound 3m.

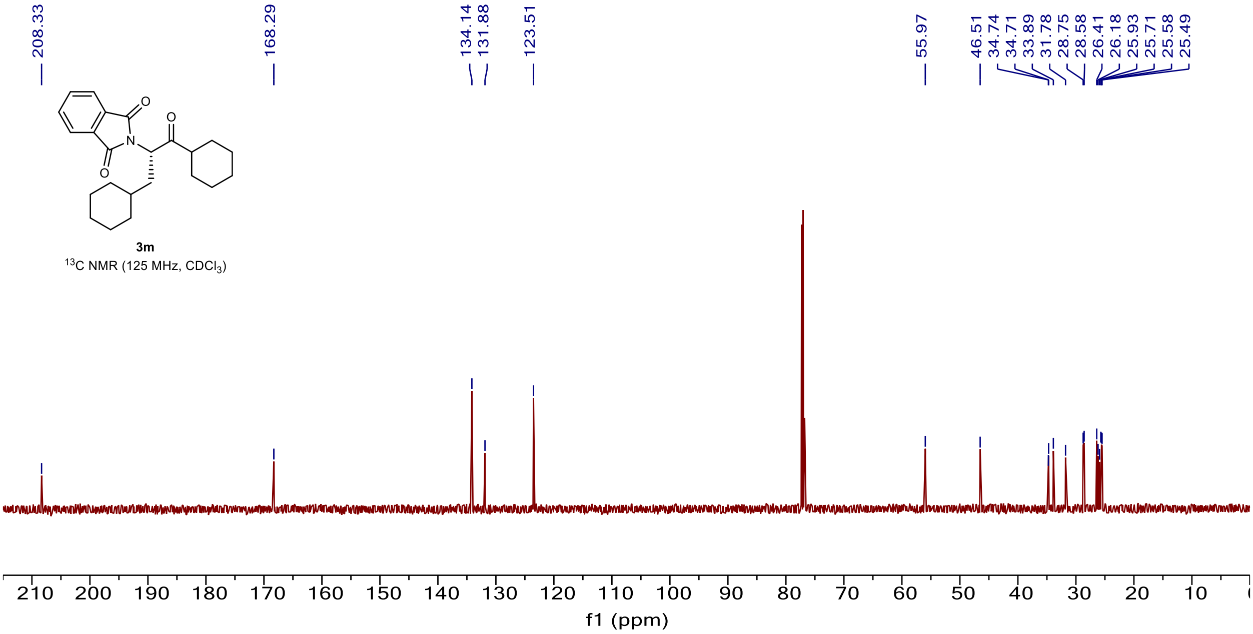

Supplementary Fig. 92. ^{13}C NMR spectrum of compound 3m.

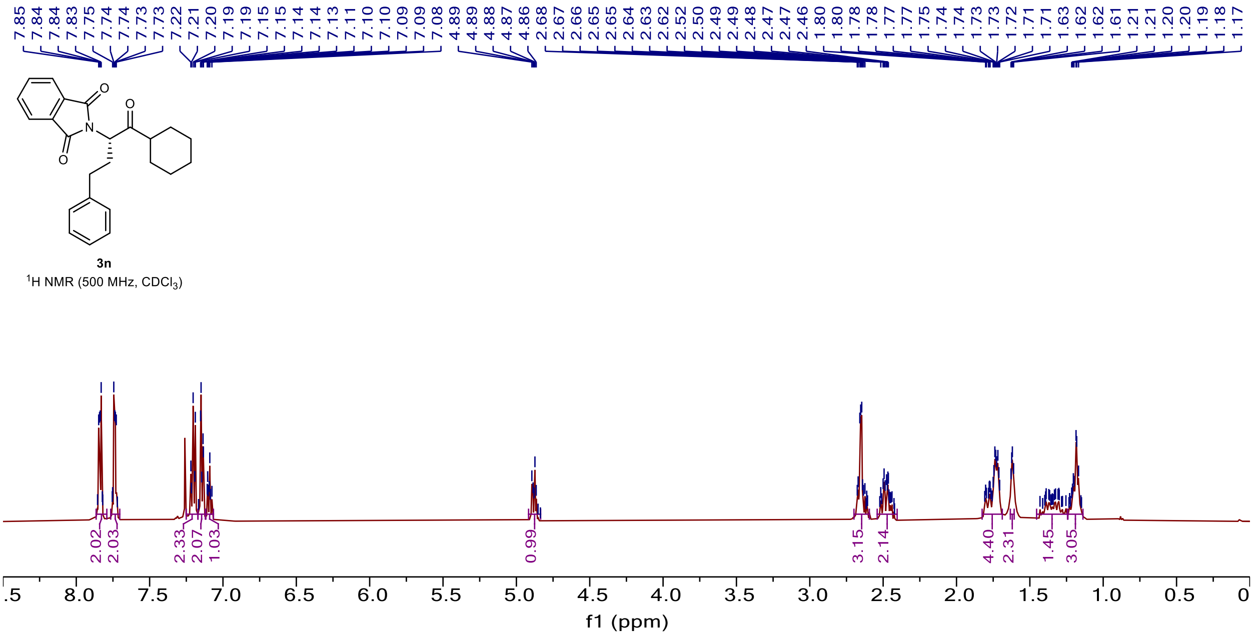

Supplementary Fig. 93. ^{1}H NMR spectrum of compound 3n.

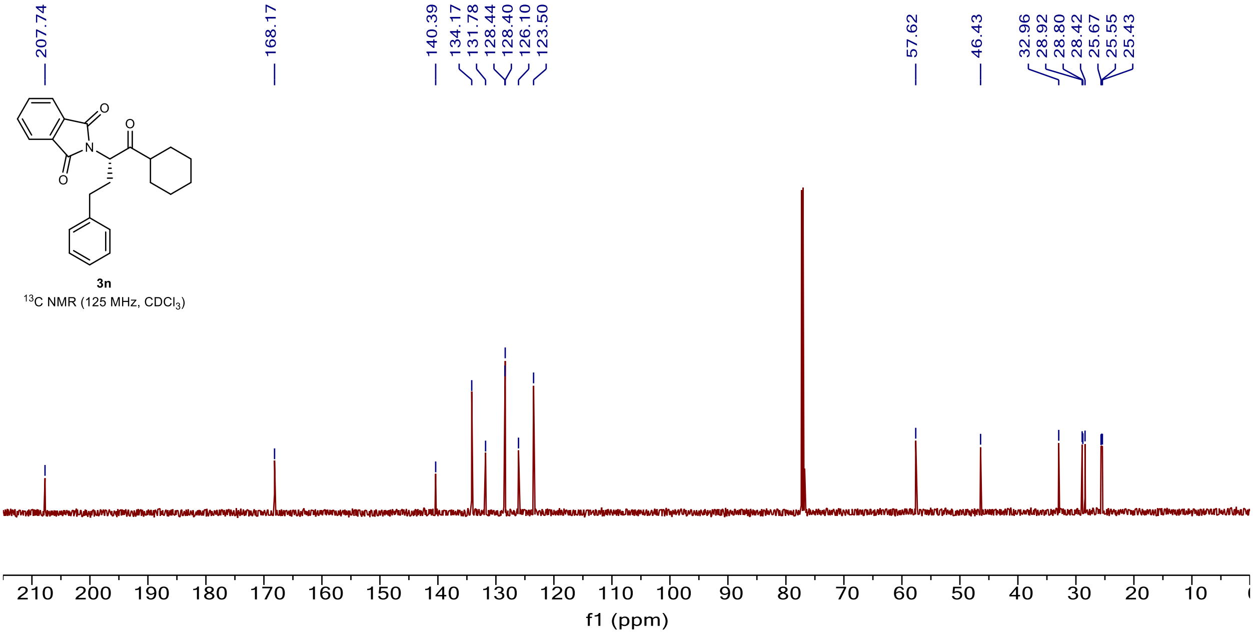

Supplementary Fig. 94. ^{13}C NMR spectrum of compound 3n.

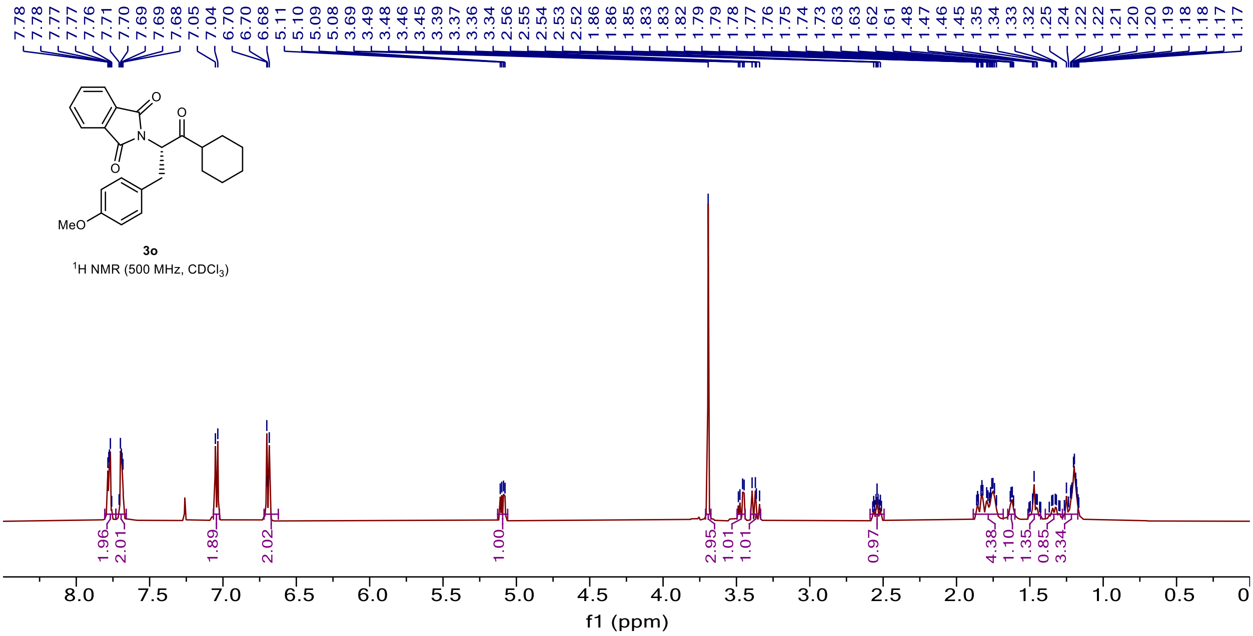

Supplementary Fig. 95. ^{1}H NMR spectrum of compound 3o.

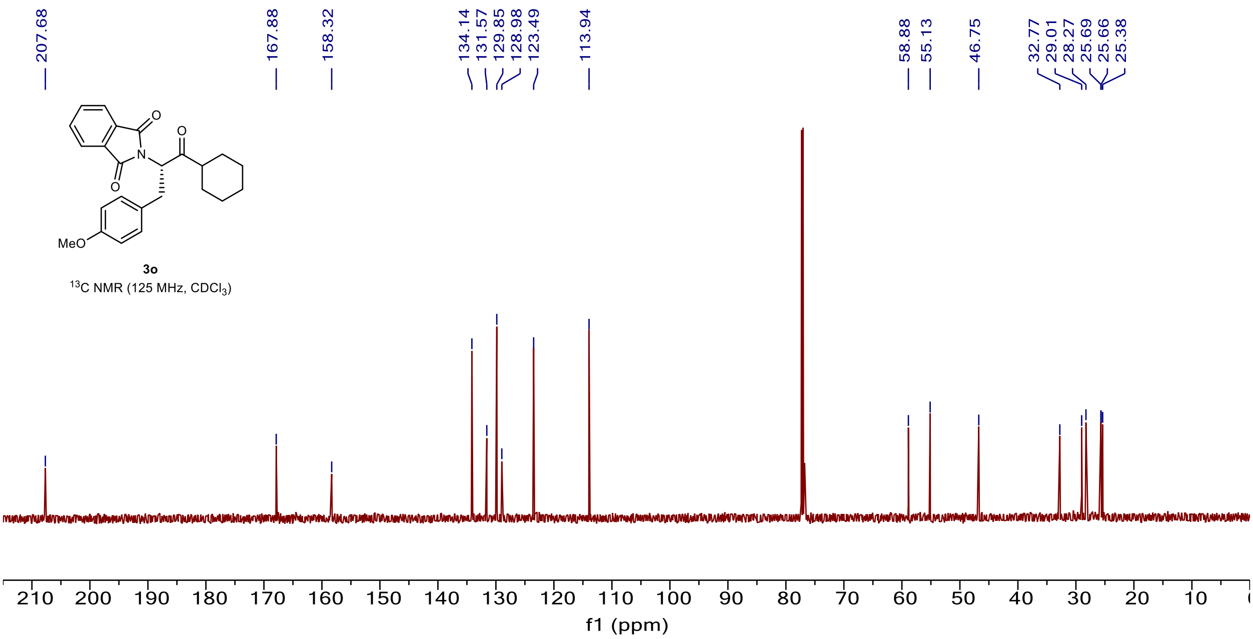

Supplementary Fig. 96. ^{13}C NMR spectrum of compound 3o.

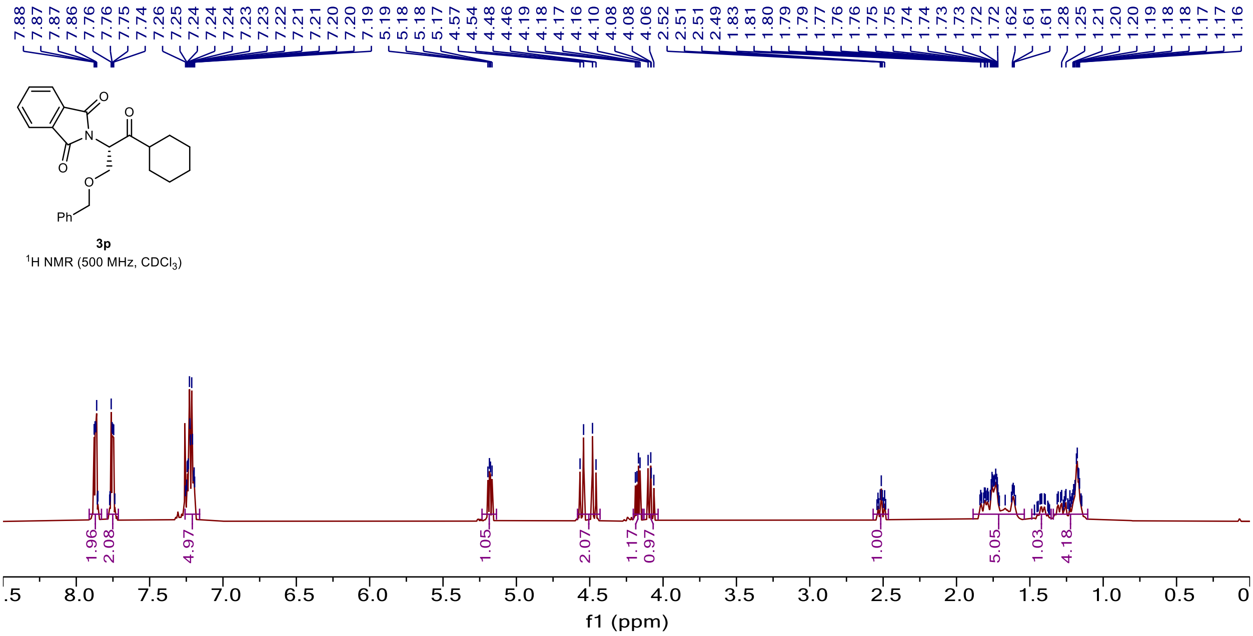

Supplementary Fig. 97. ^{1}H NMR spectrum of compound 3p.

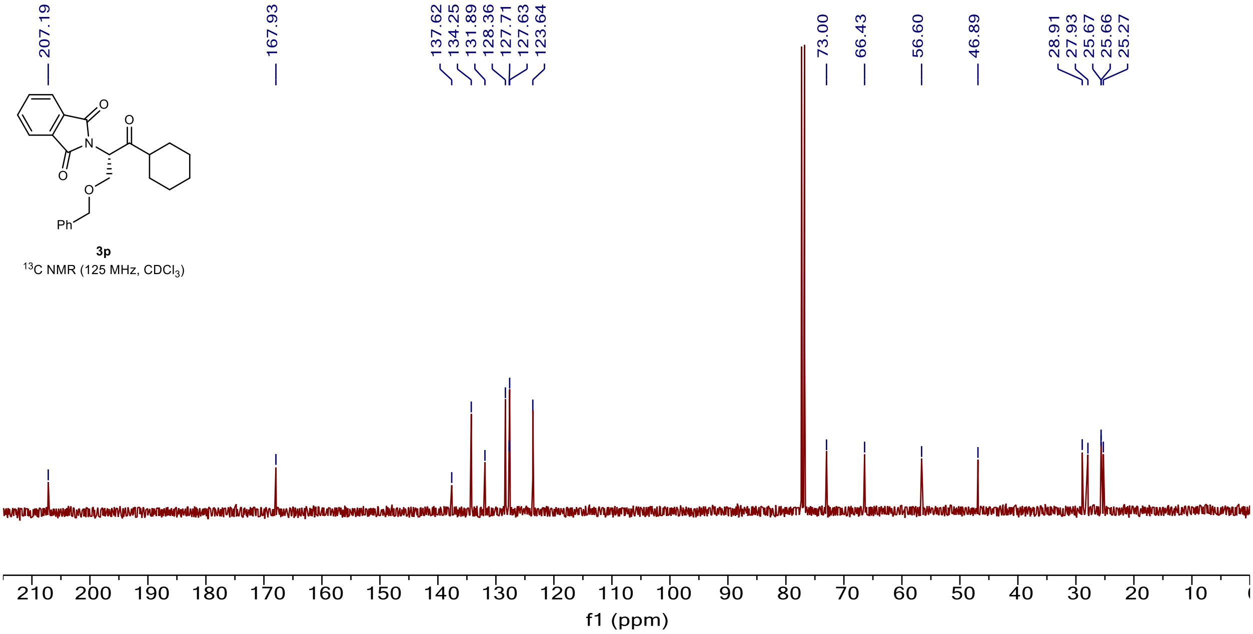

Supplementary Fig. 98. ^{13}C NMR spectrum of compound 3p.

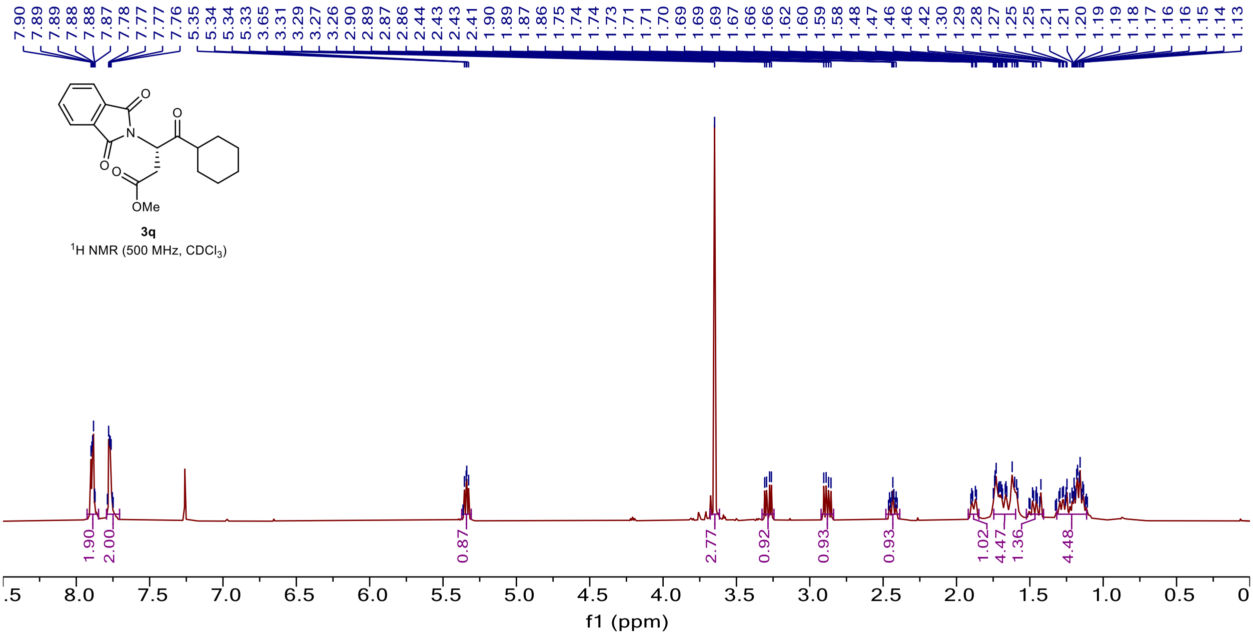

Supplementary Fig. 99. ^{1}H NMR spectrum of compound 3q.

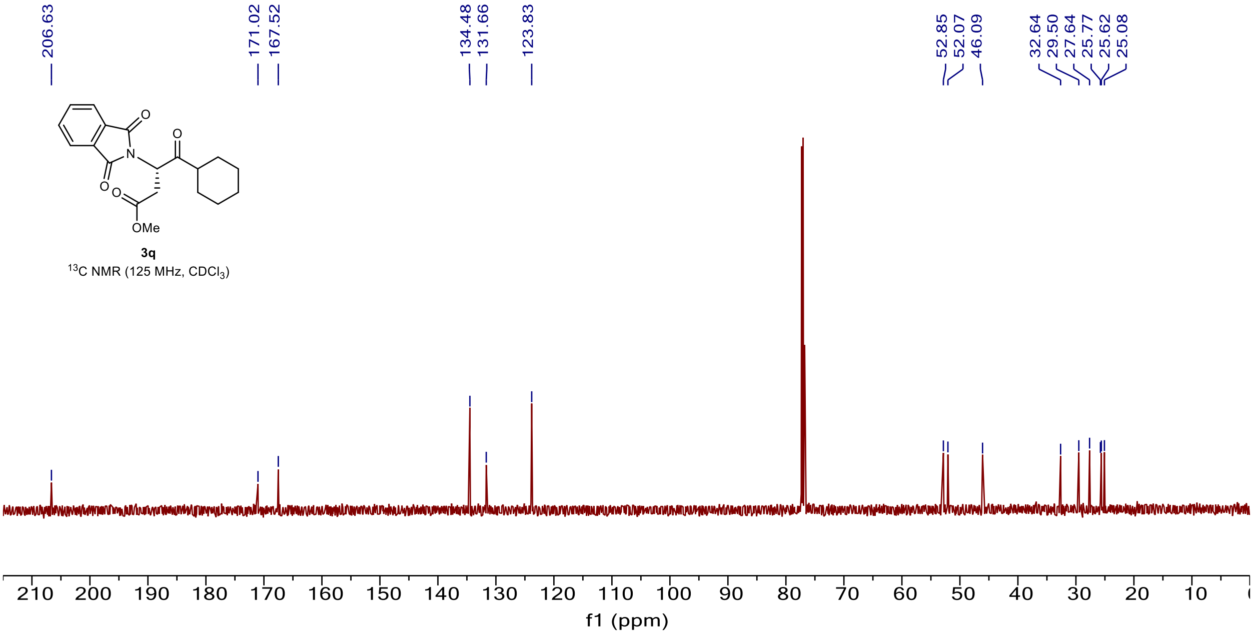

Supplementary Fig. 100. ^{13}C NMR spectrum of compound 3q.

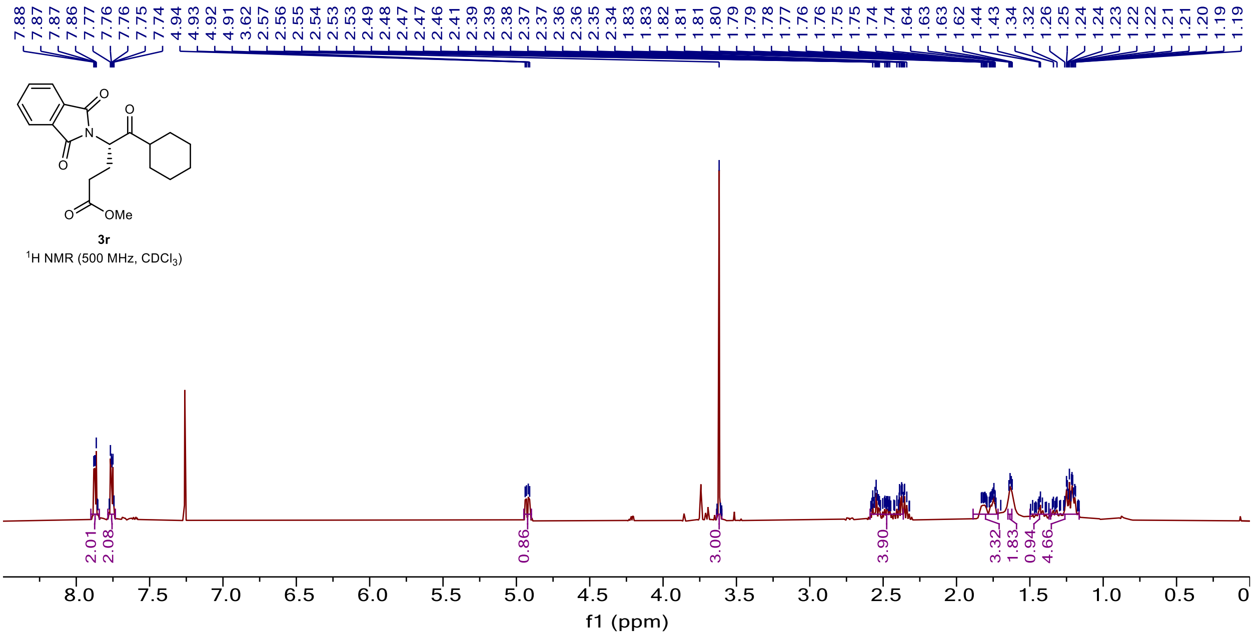

Supplementary Fig. 101. ^{1}H NMR spectrum of compound 3r.

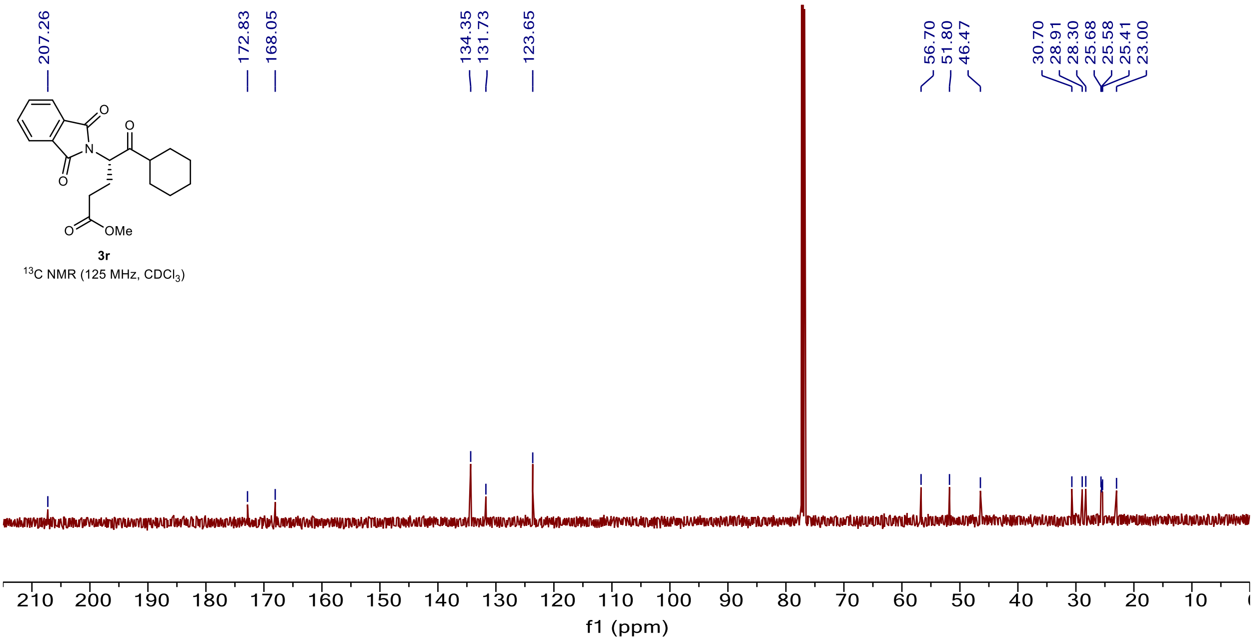

Supplementary Fig. 102. ^{13}C NMR spectrum of compound 3r.

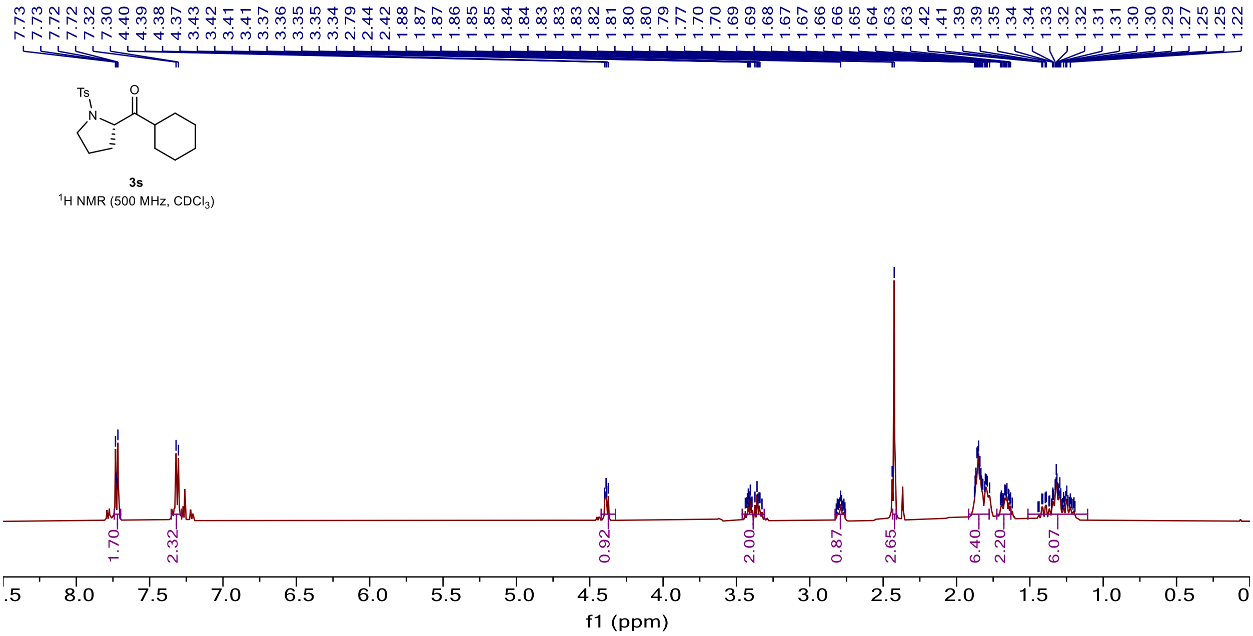

Supplementary Fig. 103. ^{1}H NMR spectrum of compound 3s.

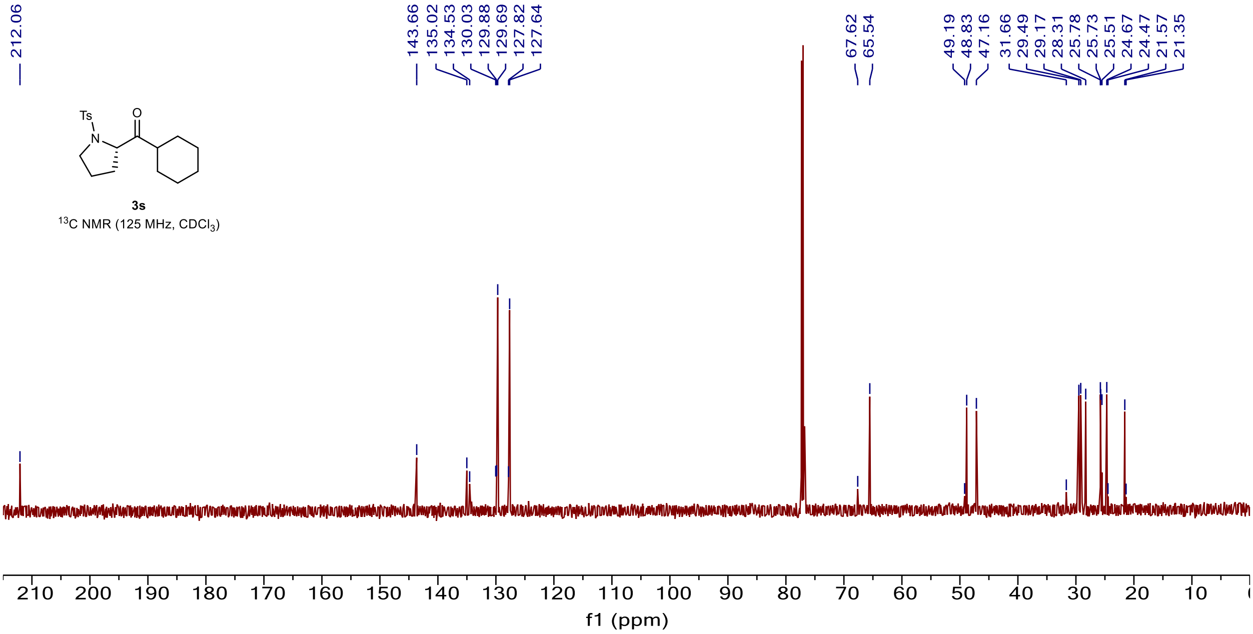

Supplementary Fig. 104. ^{13}C NMR spectrum of compound 3s.

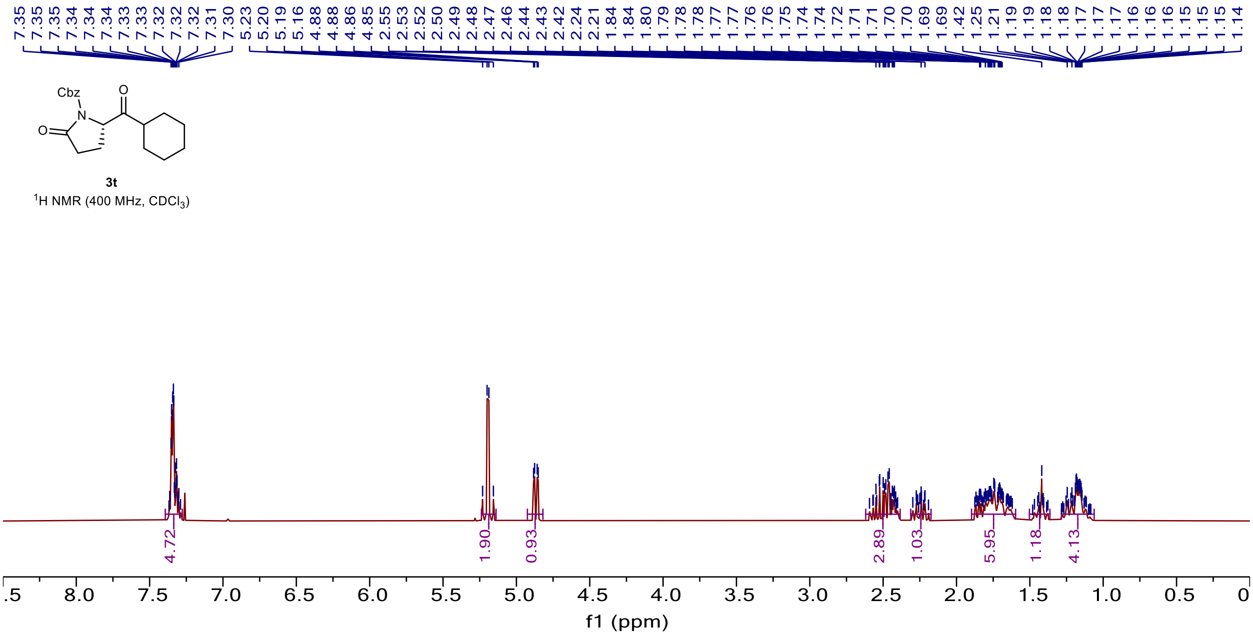

Supplementary Fig. 105. ^{1}H NMR spectrum of compound 3t.

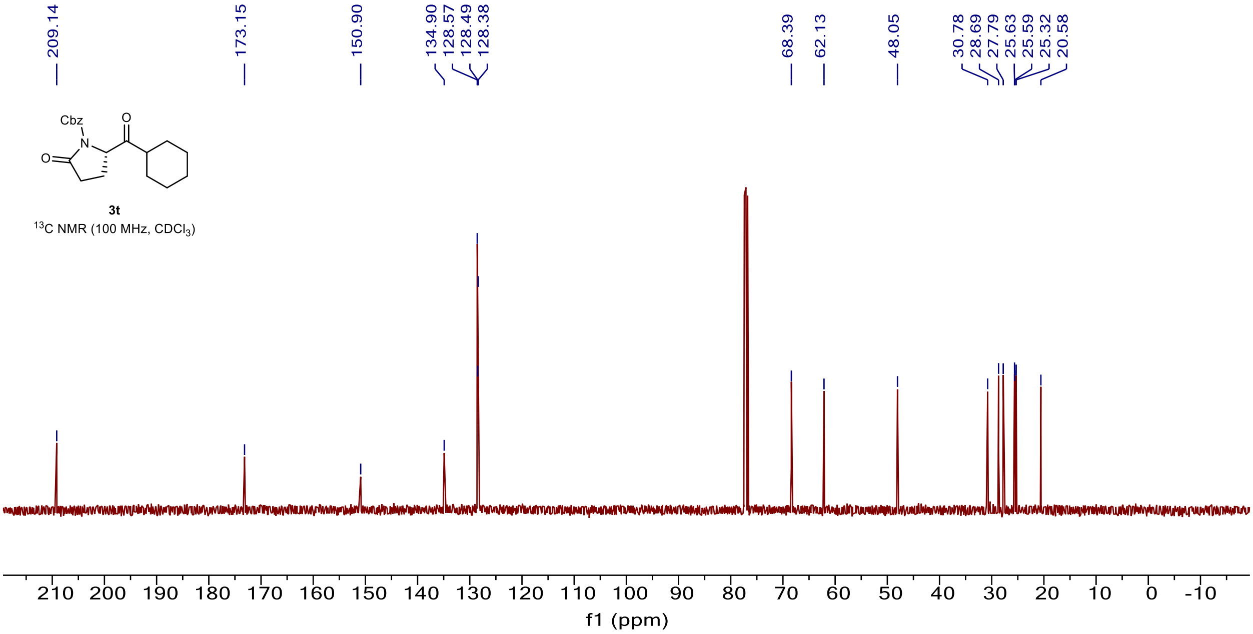

Supplementary Fig. 106. ^{13}C NMR spectrum of compound 3t.

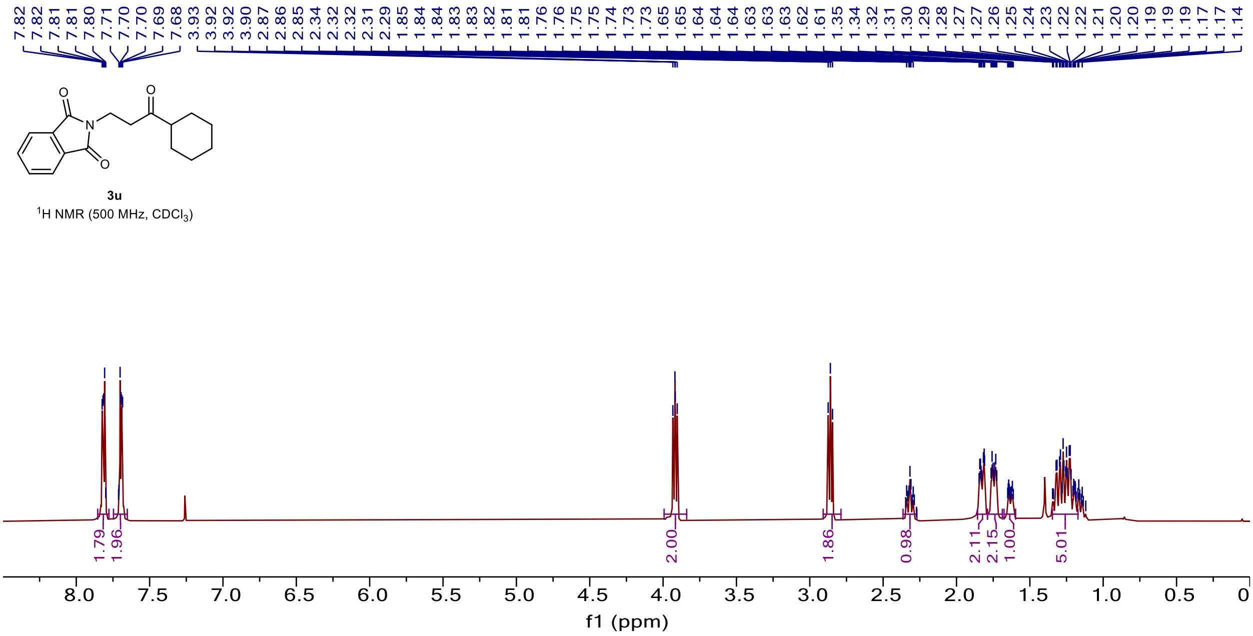

Supplementary Fig. 107. ^{1}H NMR spectrum of compound 3u.

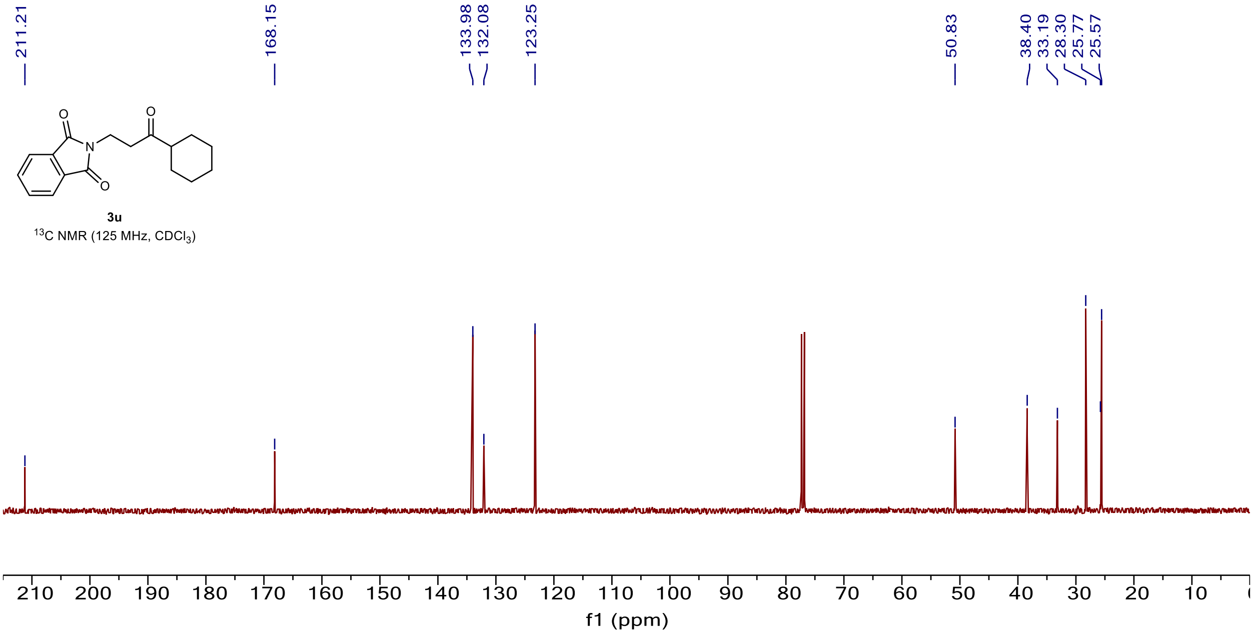

Supplementary Fig. 108. ^{13}C NMR spectrum of compound 3u.

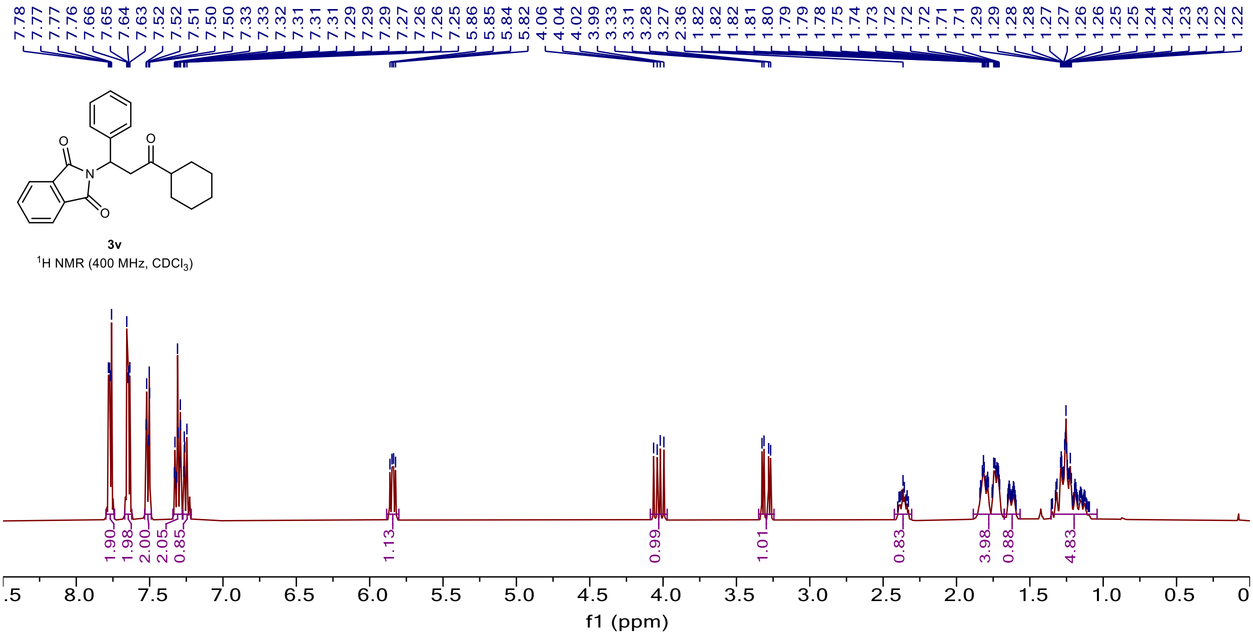

Supplementary Fig. 109. ^{1}H NMR spectrum of compound 3v.

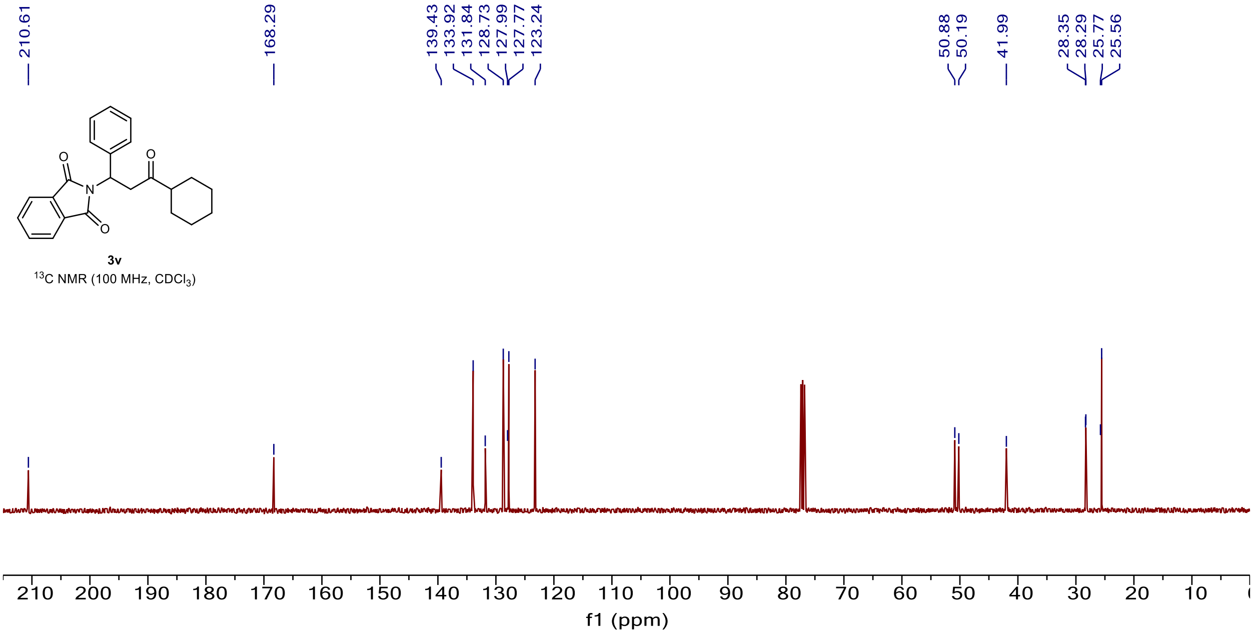

Supplementary Fig. 110. ^{13}C NMR spectrum of compound 3v.

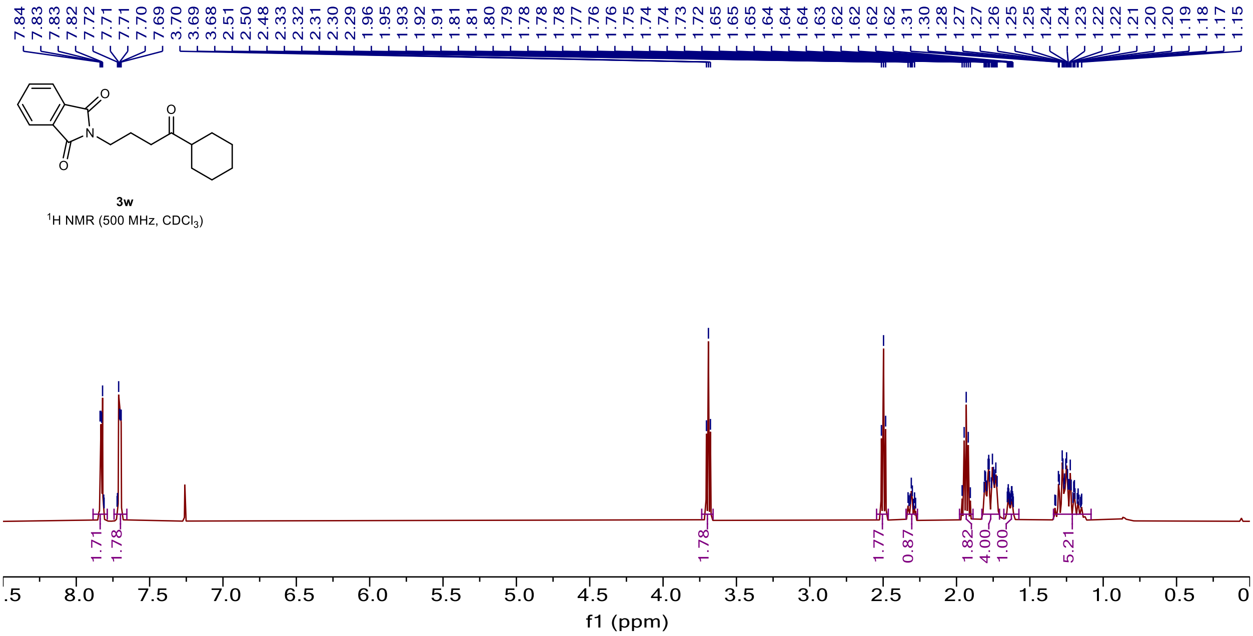

Supplementary Fig. 111. ^{1}H NMR spectrum of compound 3w.

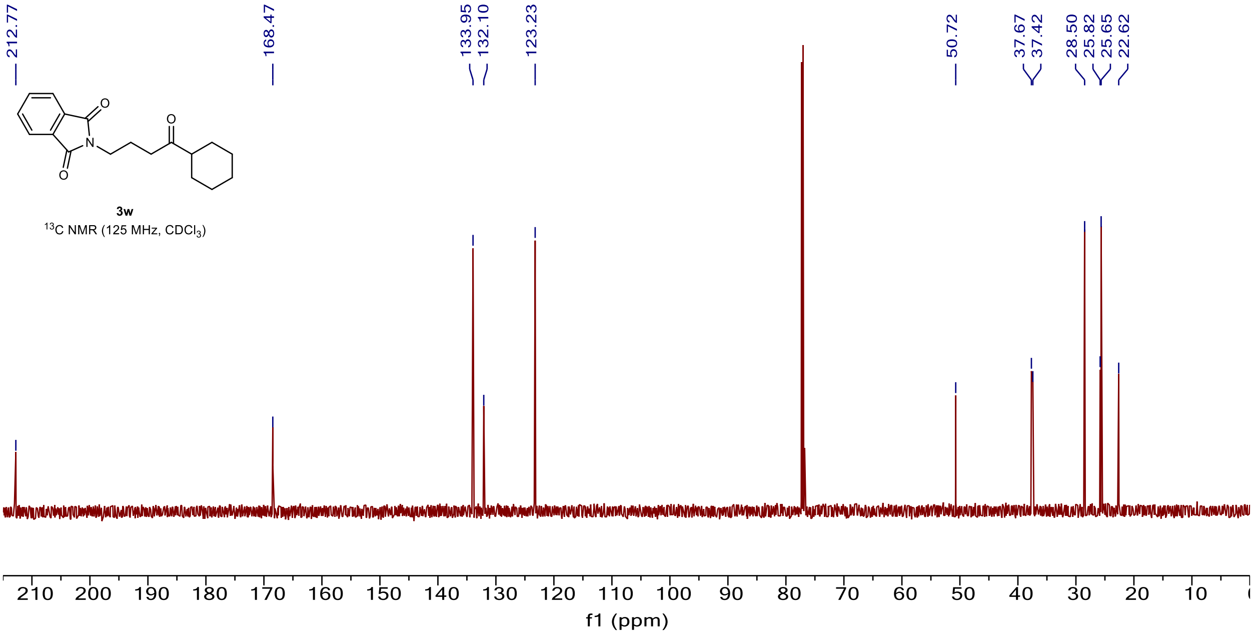

Supplementary Fig. 112. ^{13}C NMR spectrum of compound 3w.

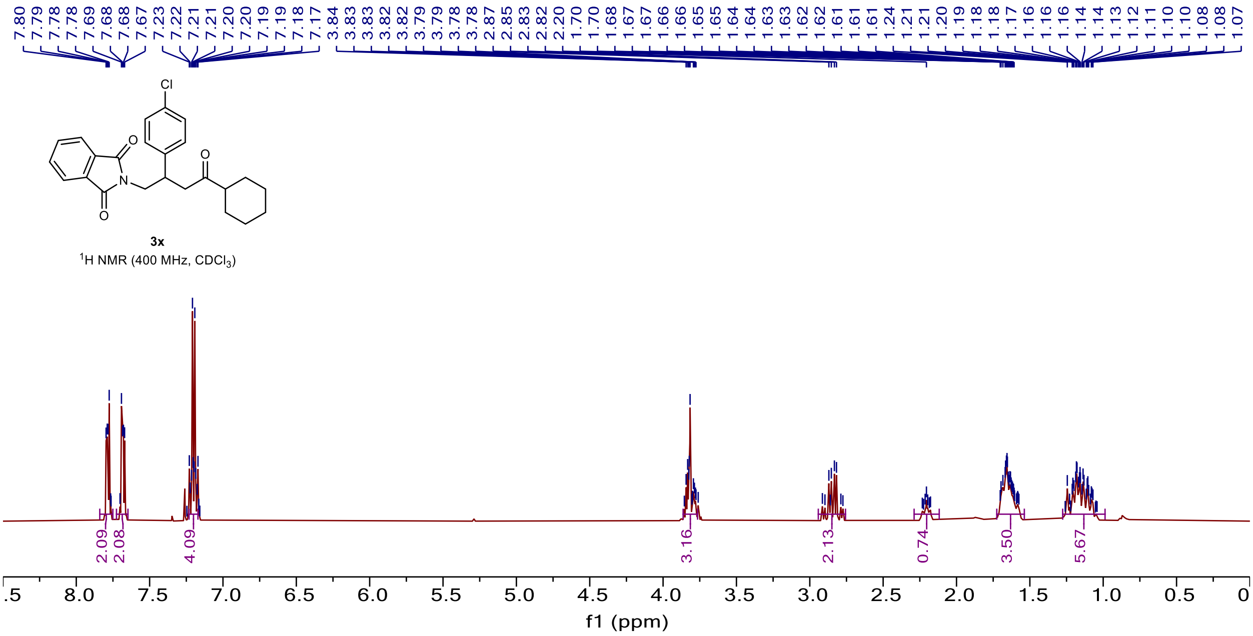

Supplementary Fig. 113. ^{1}H NMR spectrum of compound 3x.

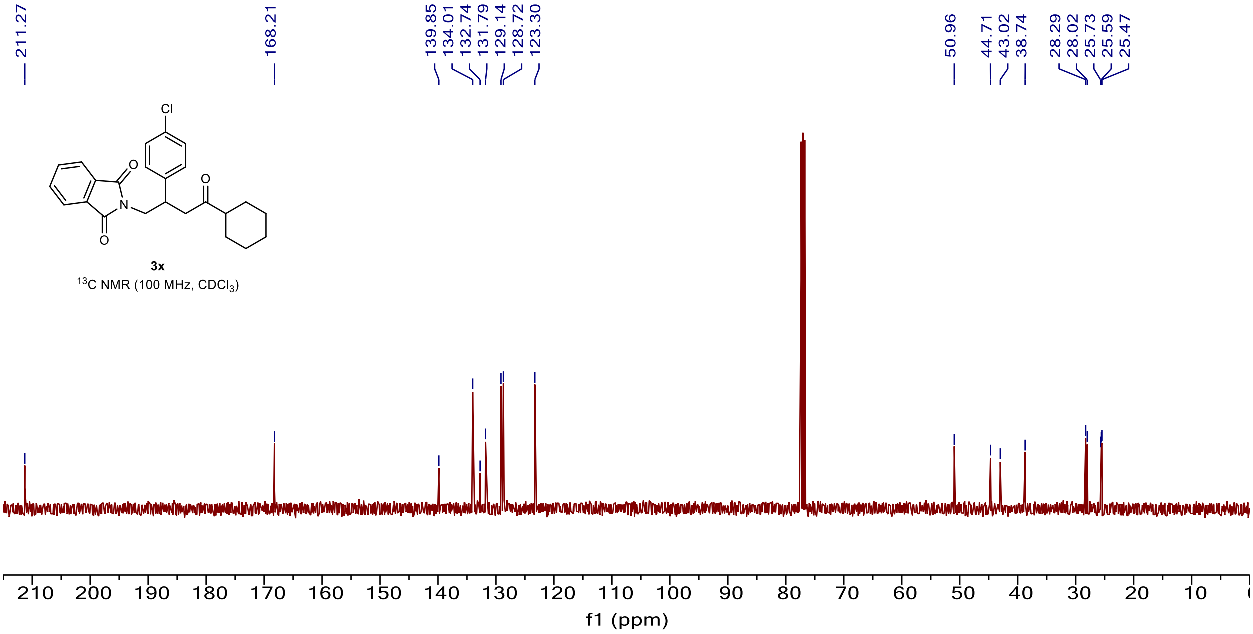

Supplementary Fig. 114. ^{13}C NMR spectrum of compound 3x.

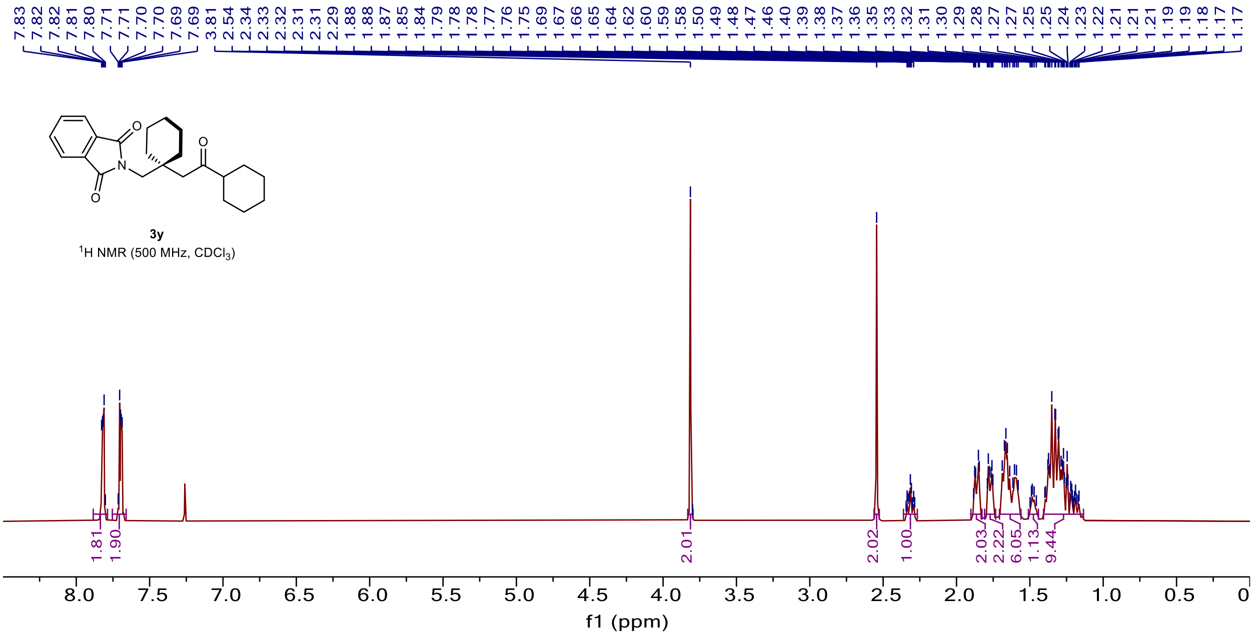

Supplementary Fig. 115. ^{1}H NMR spectrum of compound 3y.

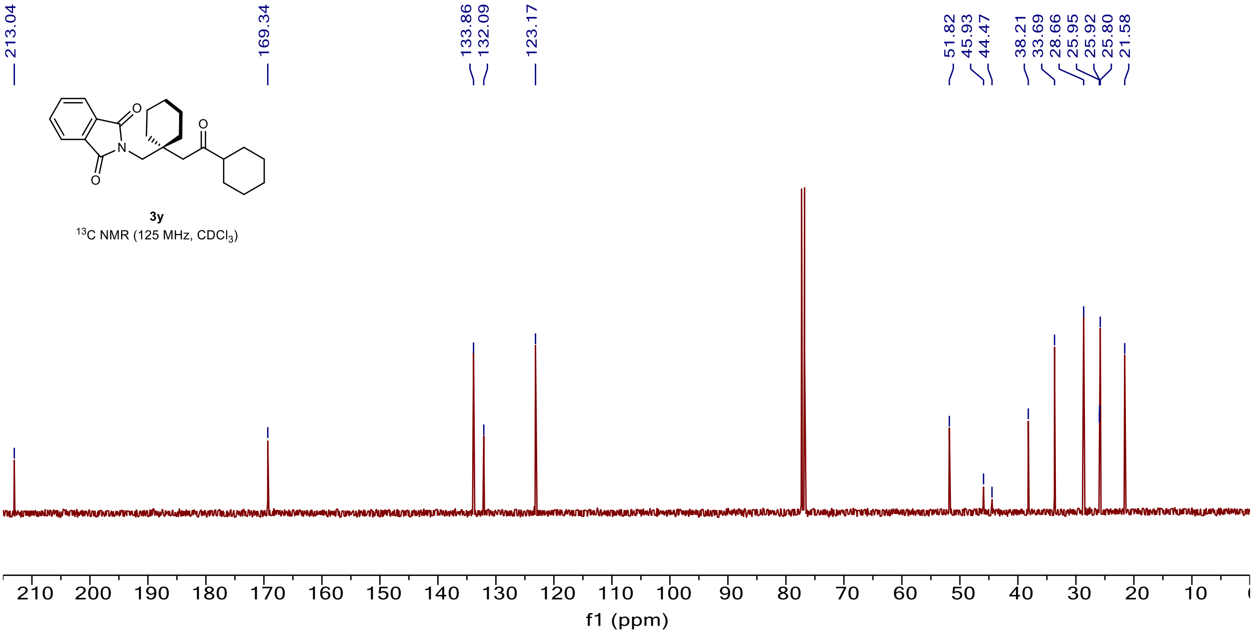

Supplementary Fig. 116. ^{13}C NMR spectrum of compound 3y.

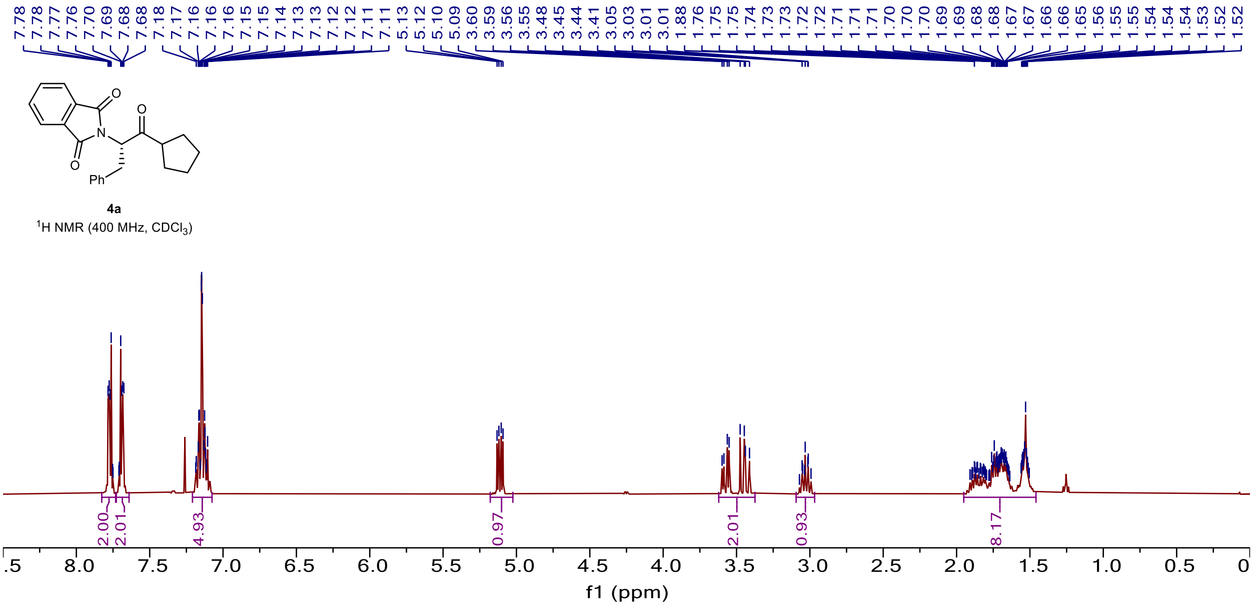

Supplementary Fig. 117. ^{1}H NMR spectrum of compound 4a.

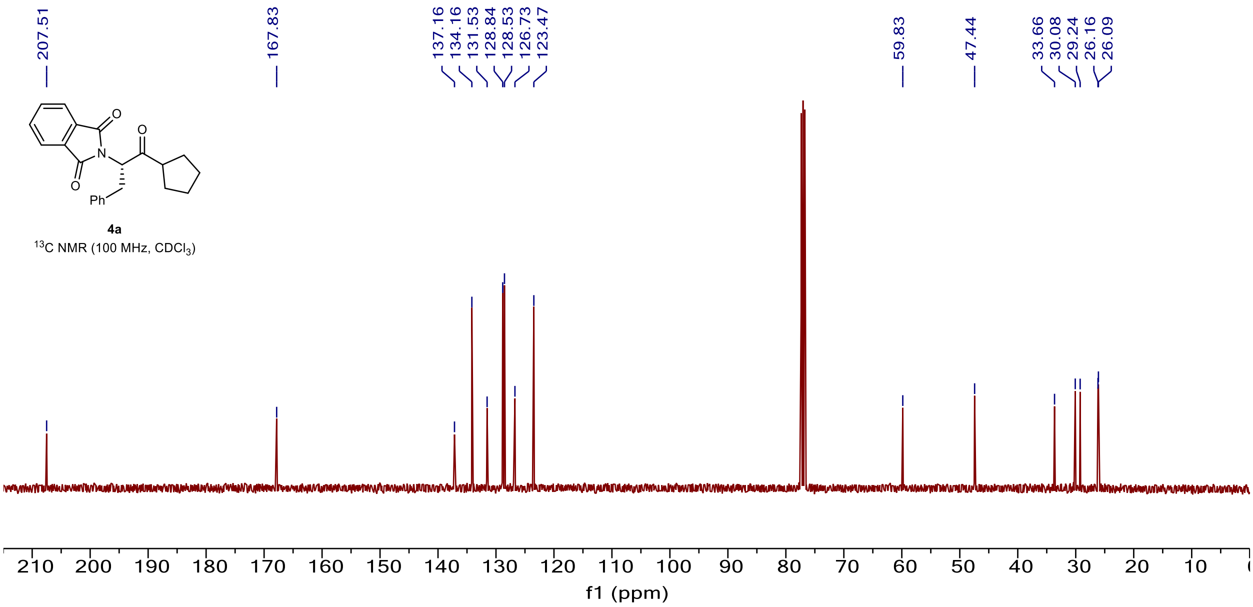

Supplementary Fig. 118. ^{13}C NMR spectrum of compound 4a.

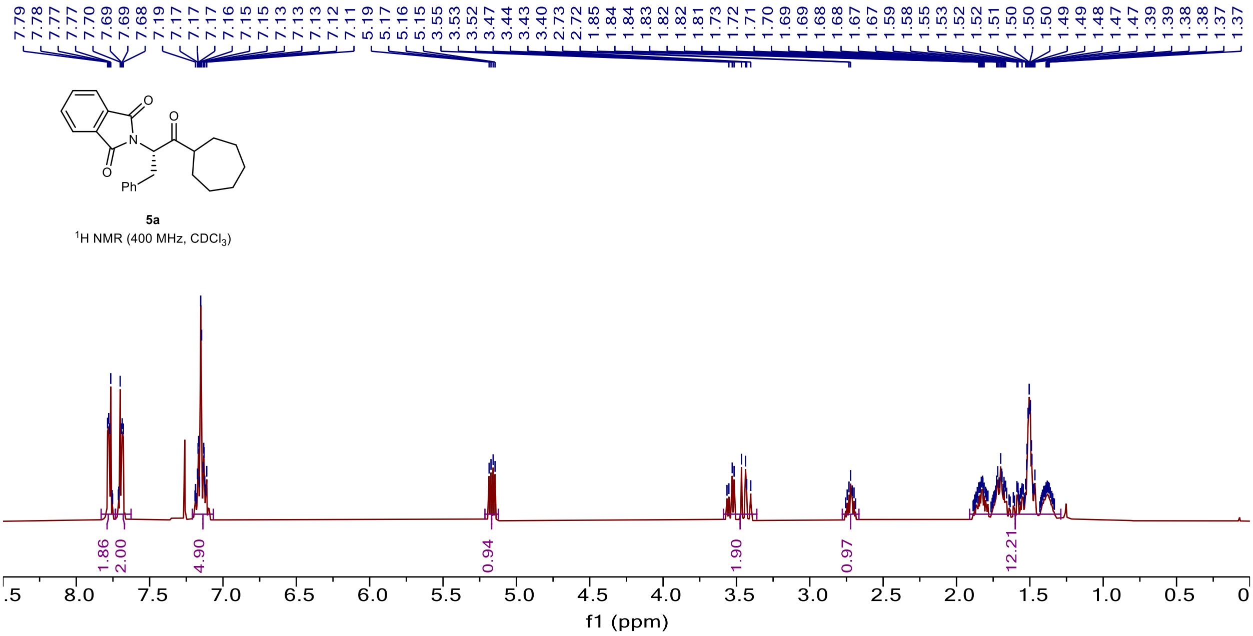

Supplementary Fig. 119. ^{1}H NMR spectrum of compound 5a.

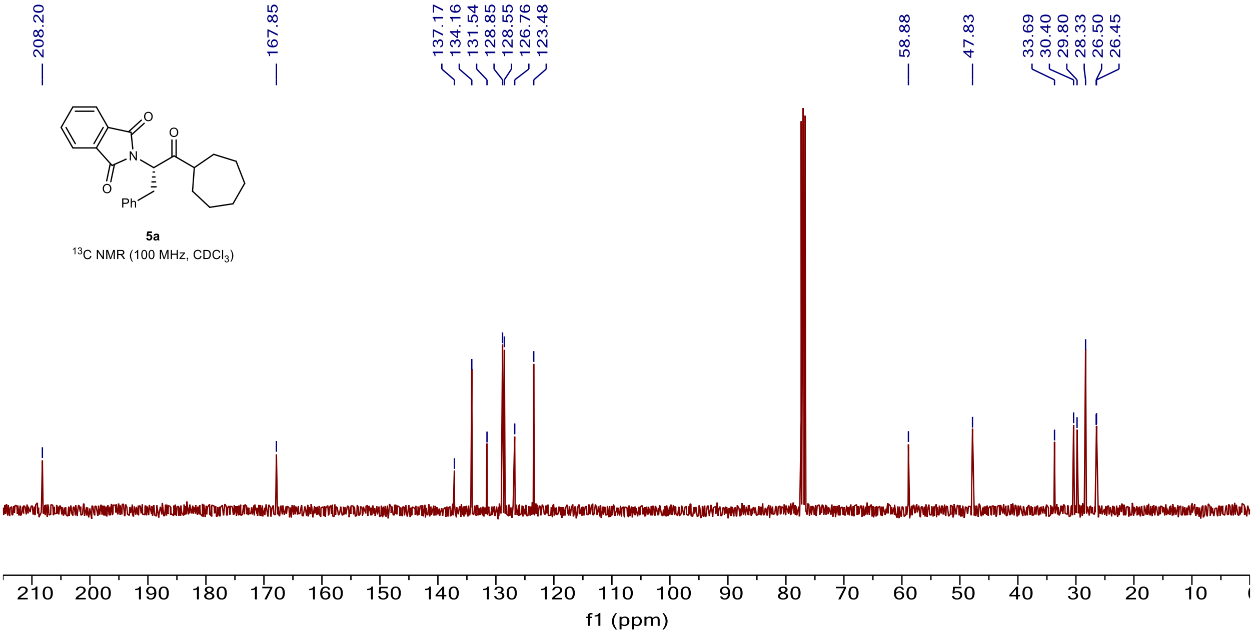

Supplementary Fig. 120. ^{13}C NMR spectrum of compound 5a.

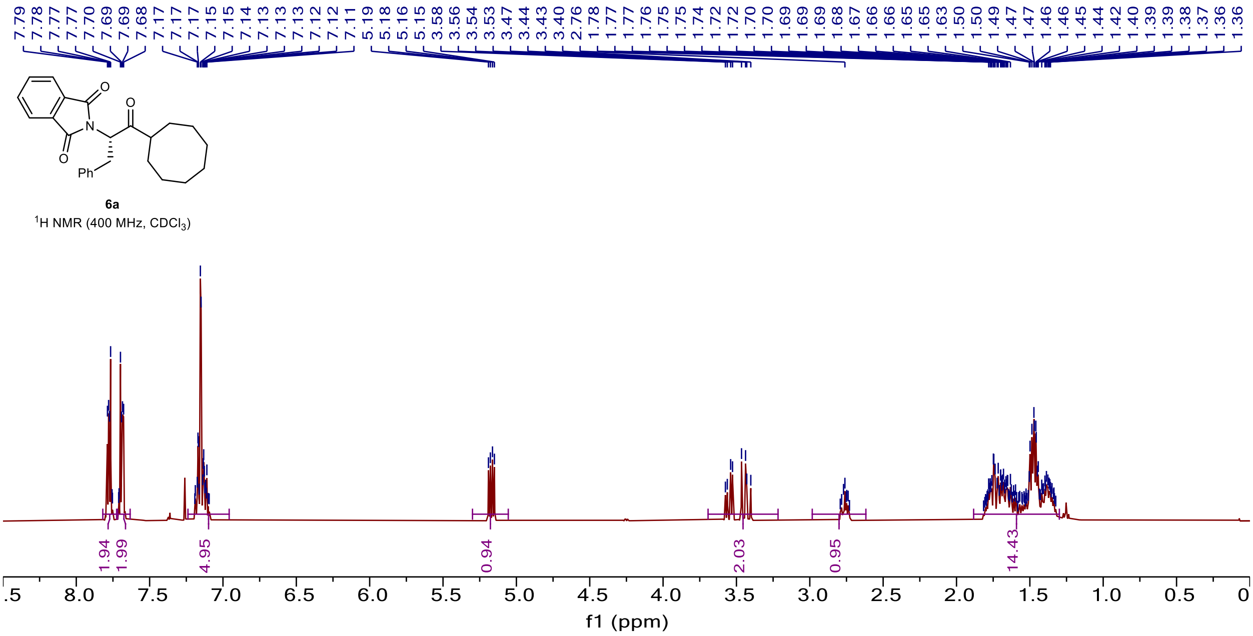

Supplementary Fig. 121. ^{1}H NMR spectrum of compound 6a.

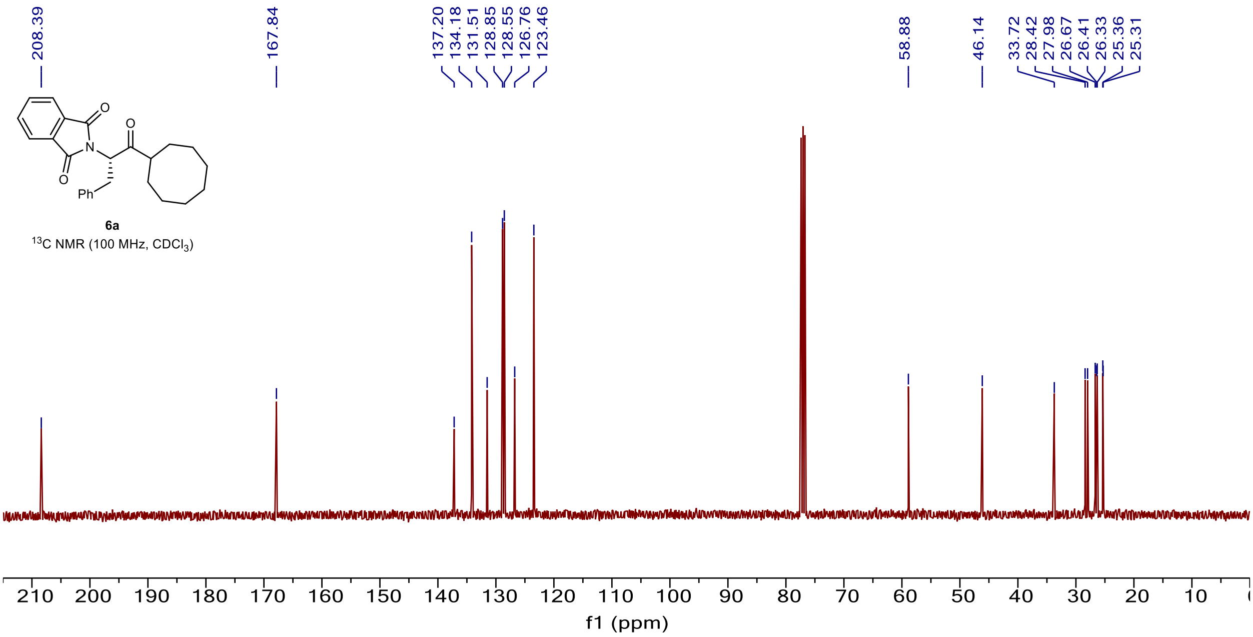

Supplementary Fig. 122. ^{13}C NMR spectrum of compound 6a.

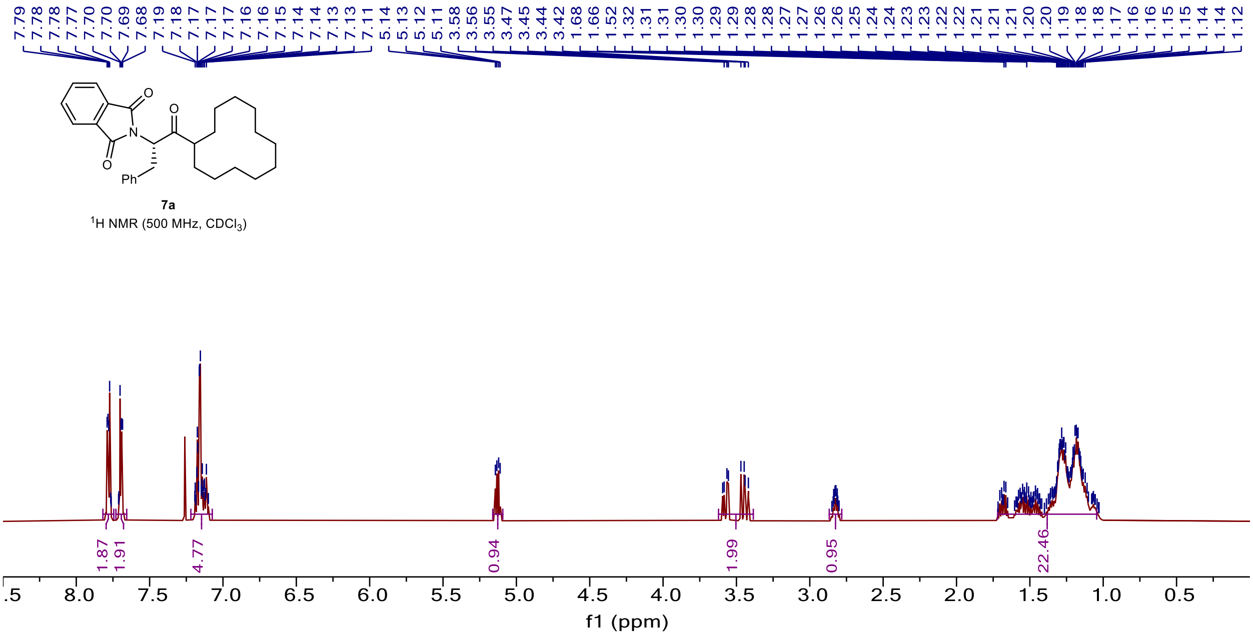

Supplementary Fig. 123. ^{1}H NMR spectrum of compound 7a.

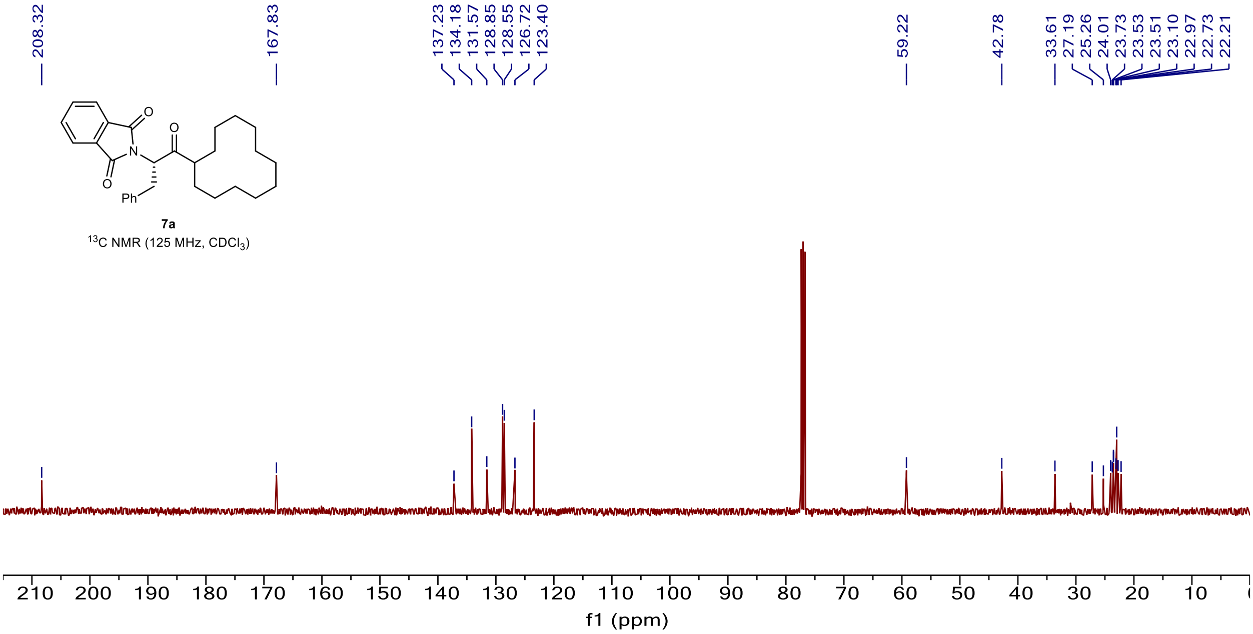

Supplementary Fig. 124. ^{13}C NMR spectrum of compound 7a.

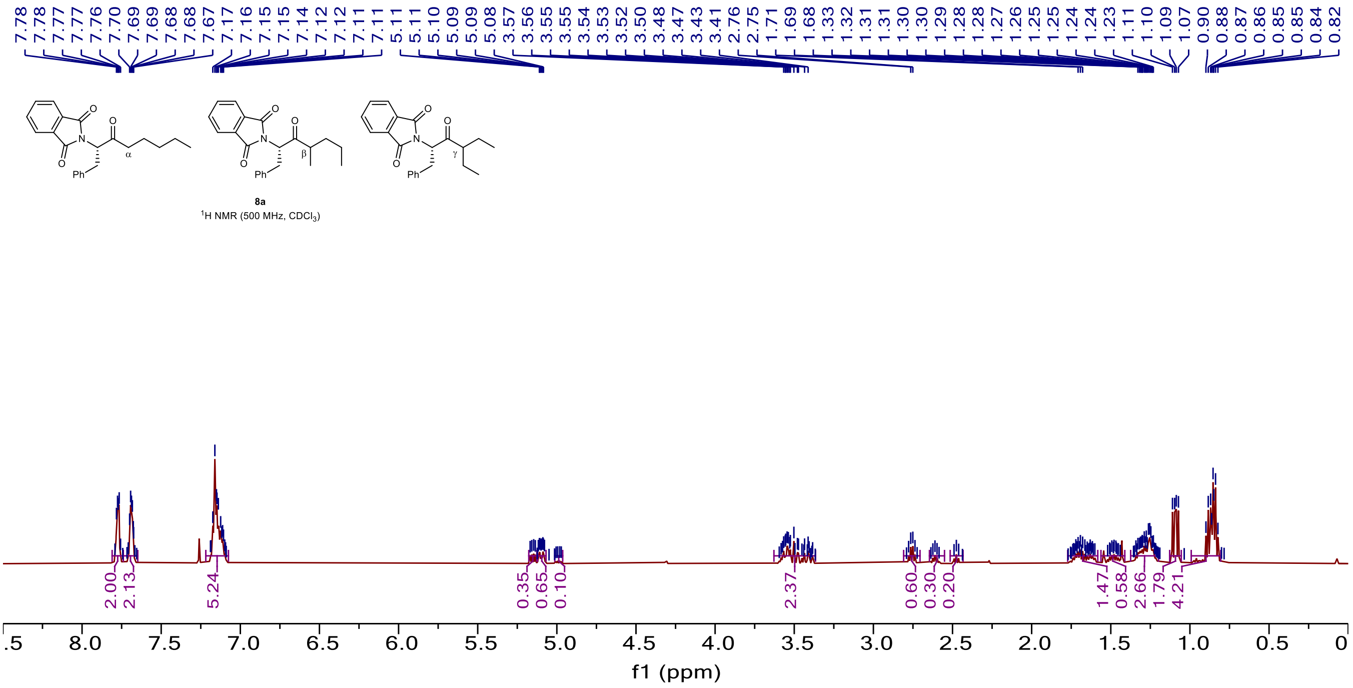

Supplementary Fig. 125. ^{1}H NMR spectrum of compound 8a.

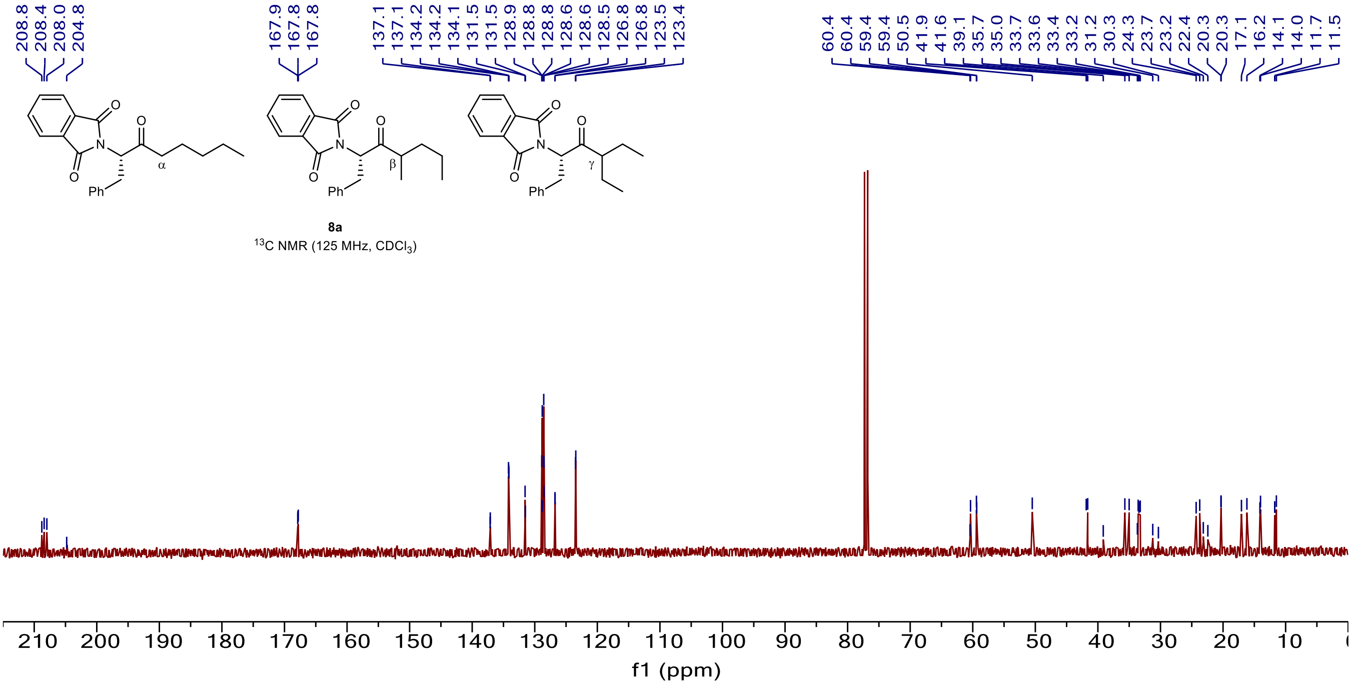

Supplementary Fig. 126. ^{1}H NMR spectrum of compound 8a.

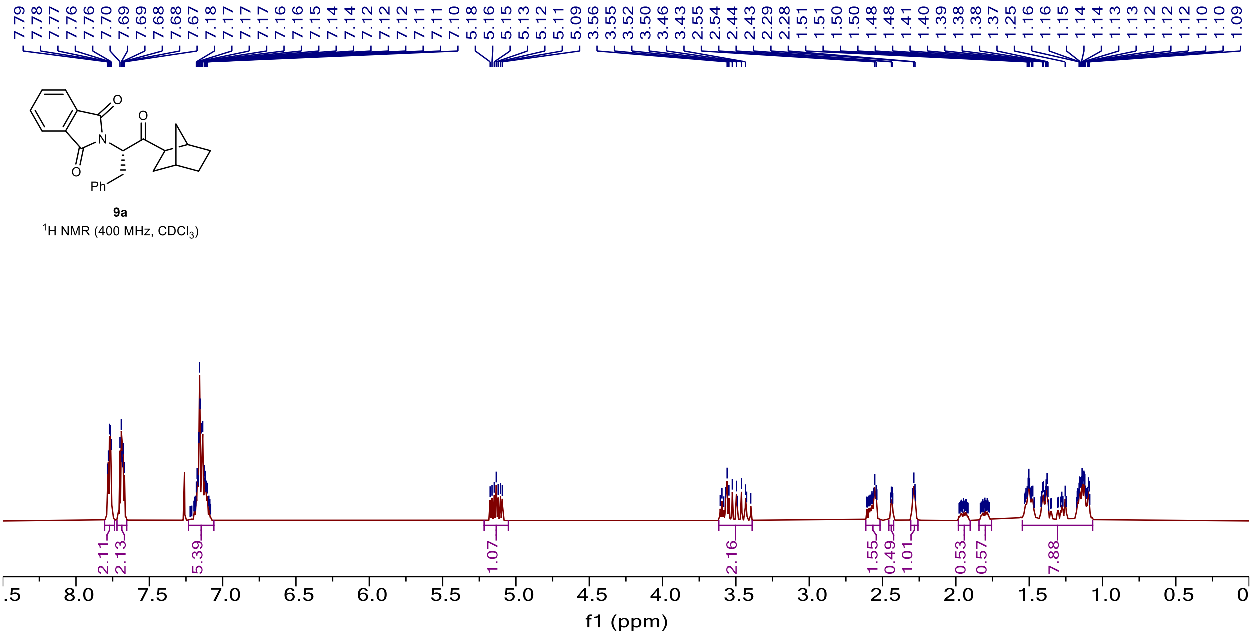

Supplementary Fig. 127. ^{1}H NMR spectrum of compound 9a.

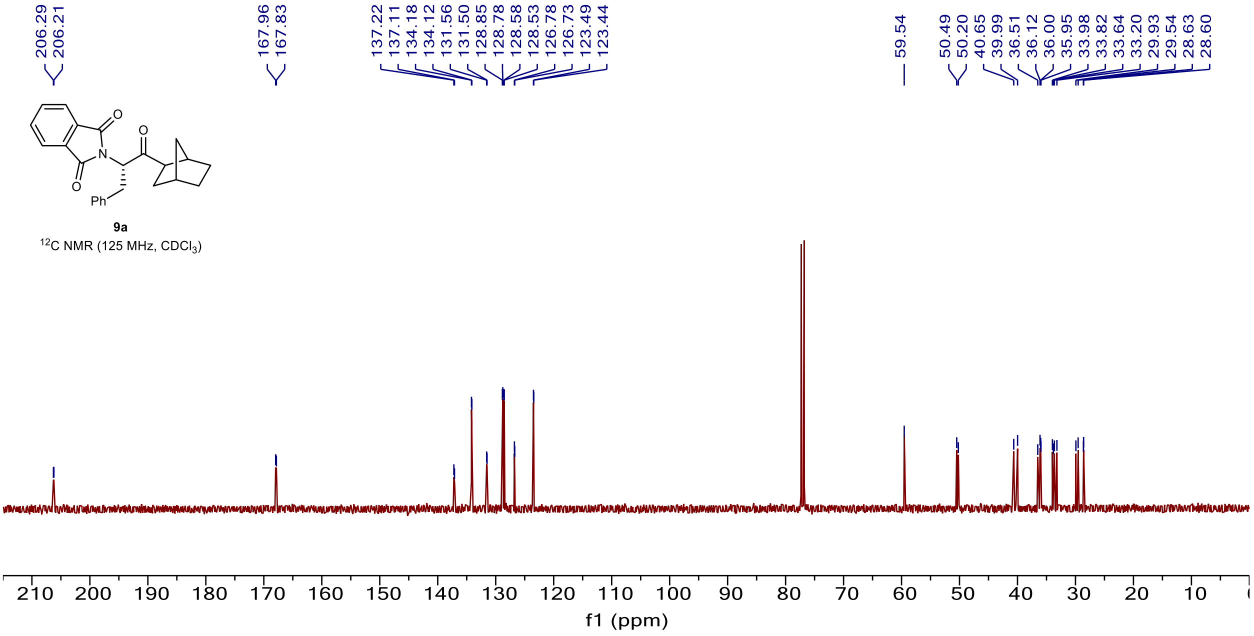

Supplementary Fig. 128. ^{13}C NMR spectrum of compound 9a.

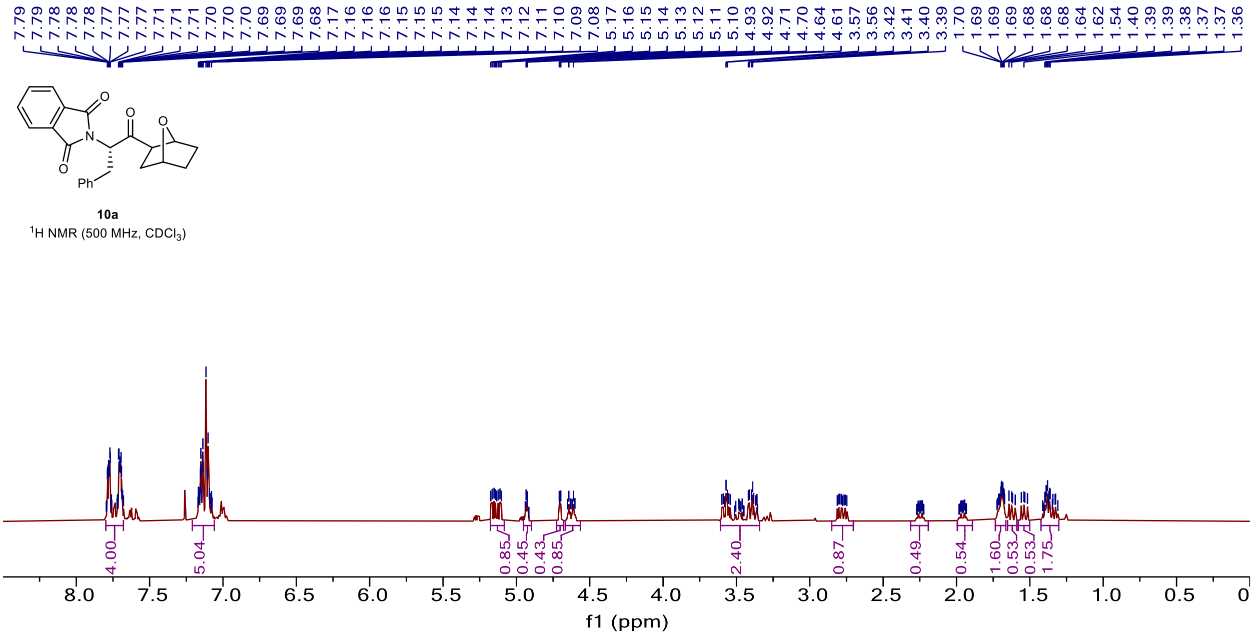

Supplementary Fig. 129. ^{1}H NMR spectrum of compound 10a.

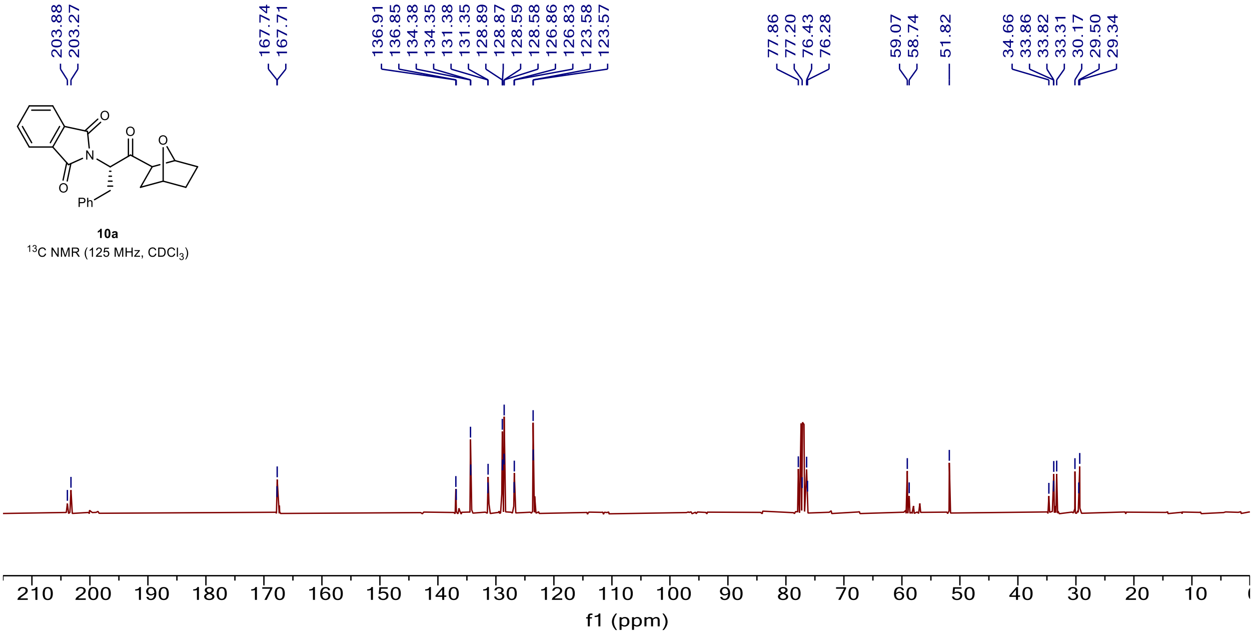

Supplementary Fig. 130. ^{13}C NMR spectrum of compound 10a.

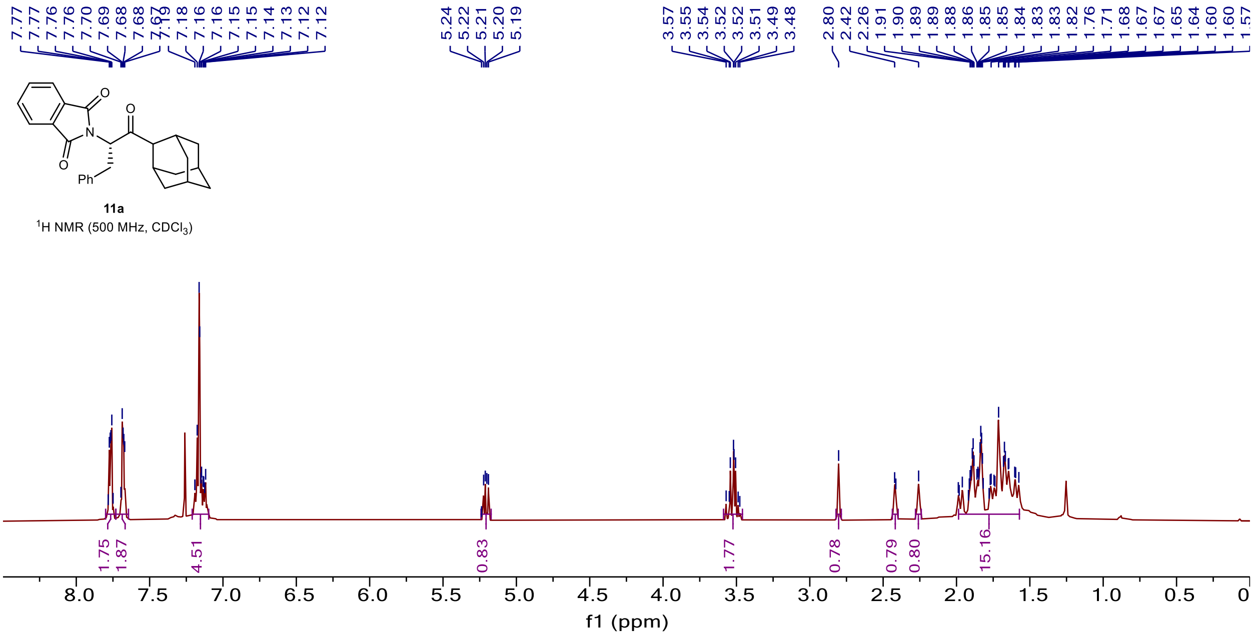

Supplementary Fig. 131. ^{1}H NMR spectrum of compound 11a.

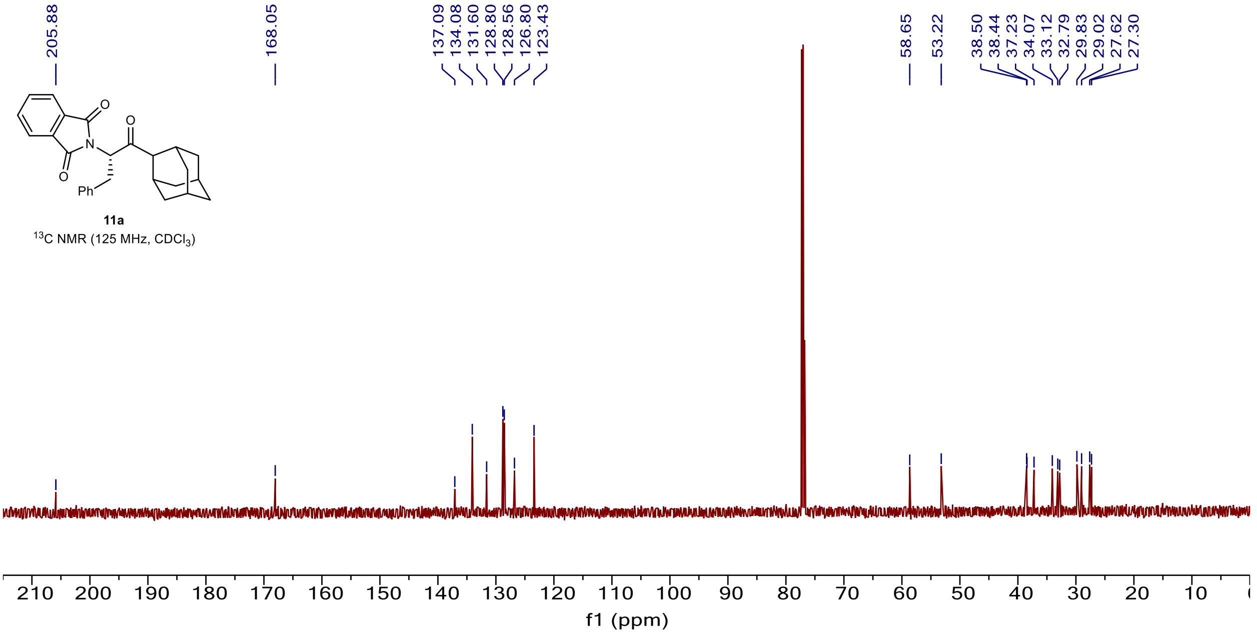

Supplementary Fig. 132. ^{13}C NMR spectrum of compound 11a.

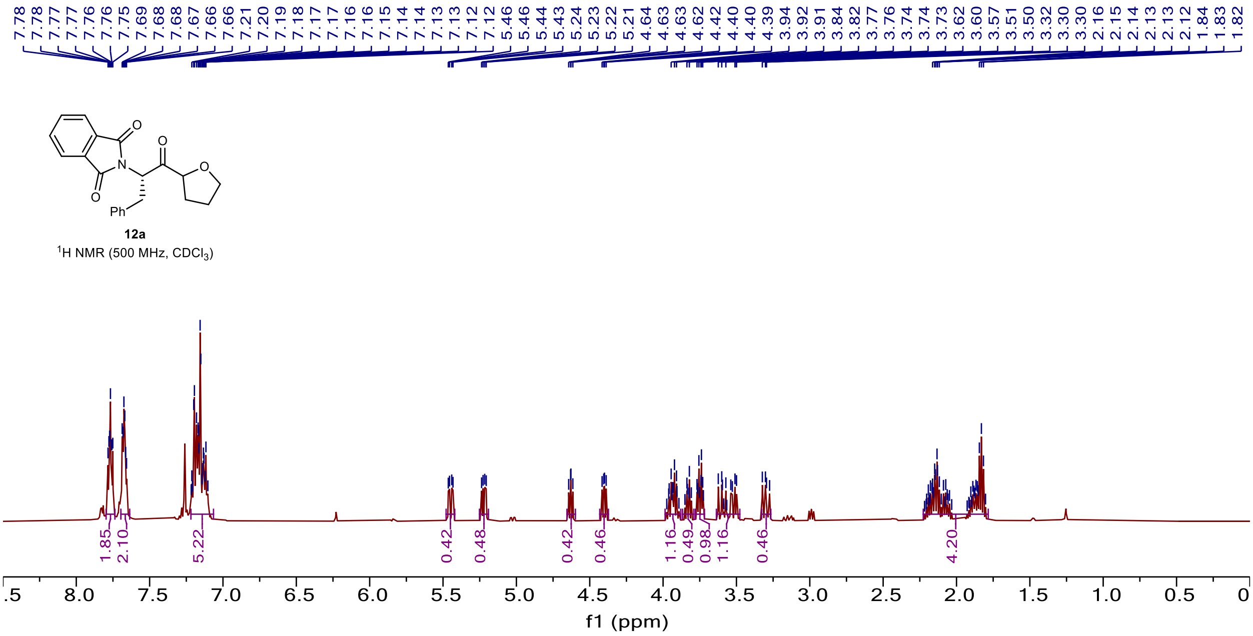

Supplementary Fig. 133. ^{1}H NMR spectrum of compound 12a.

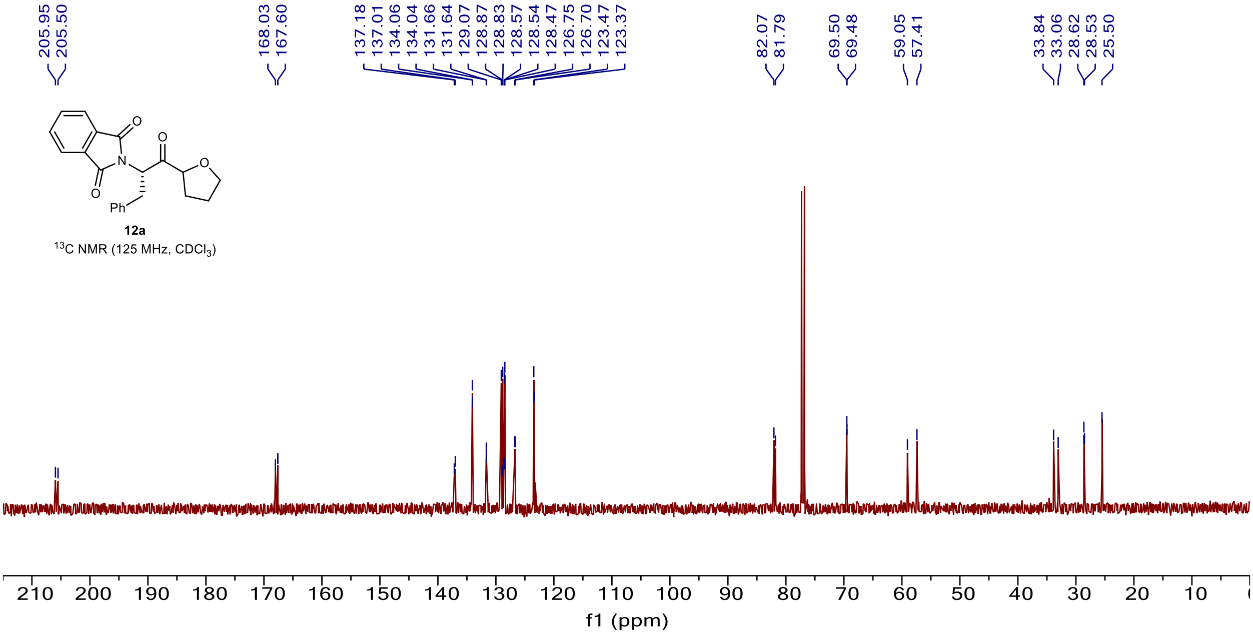

Supplementary Fig. 134. ^{13}C NMR spectrum of compound 12a.

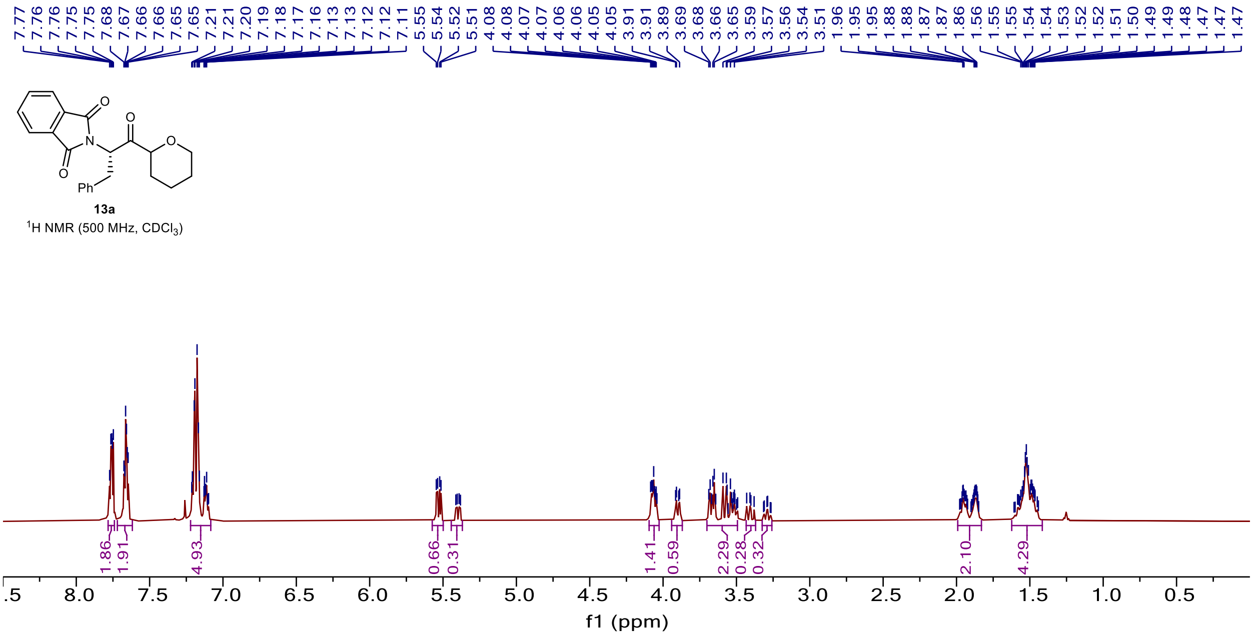

Supplementary Fig. 135. ^{1}H NMR spectrum of compound 13a.

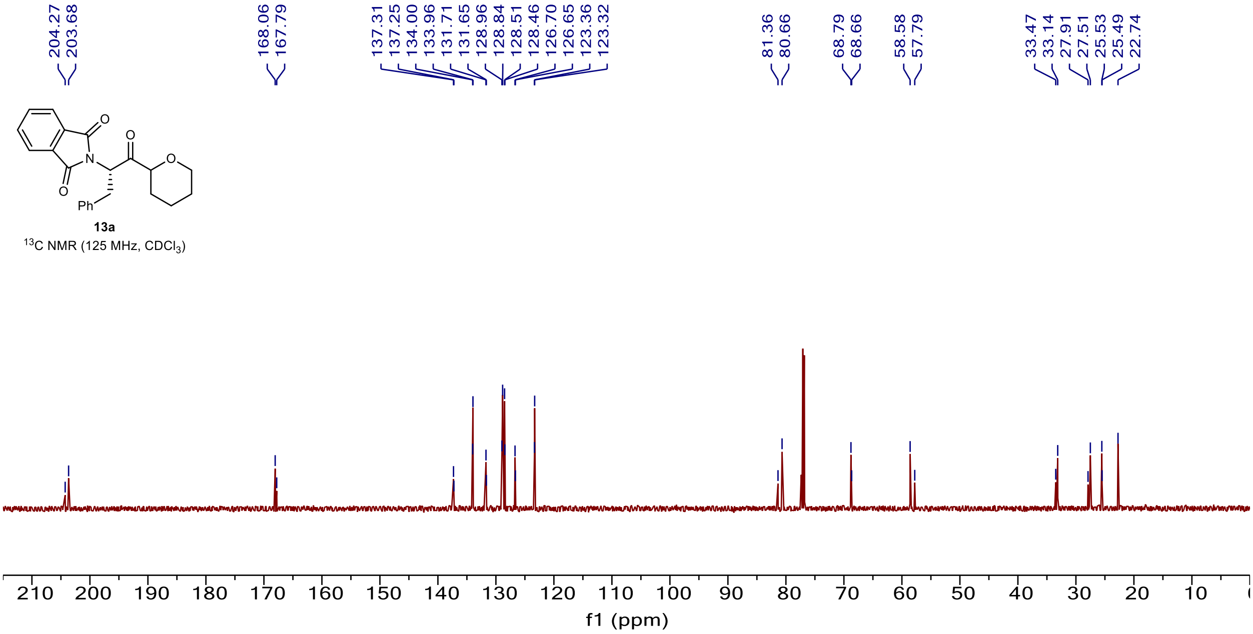

Supplementary Fig. 136. ^{13}C NMR spectrum of compound 13a.

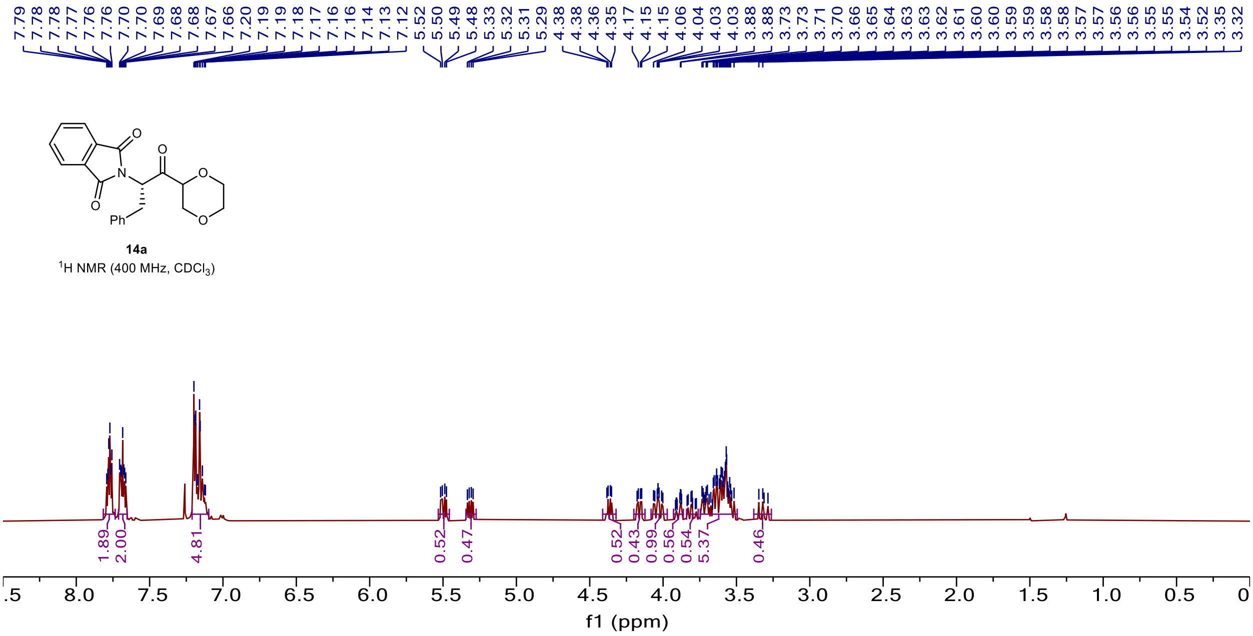

Supplementary Fig. 137. ^{1}H NMR spectrum of compound 14a.

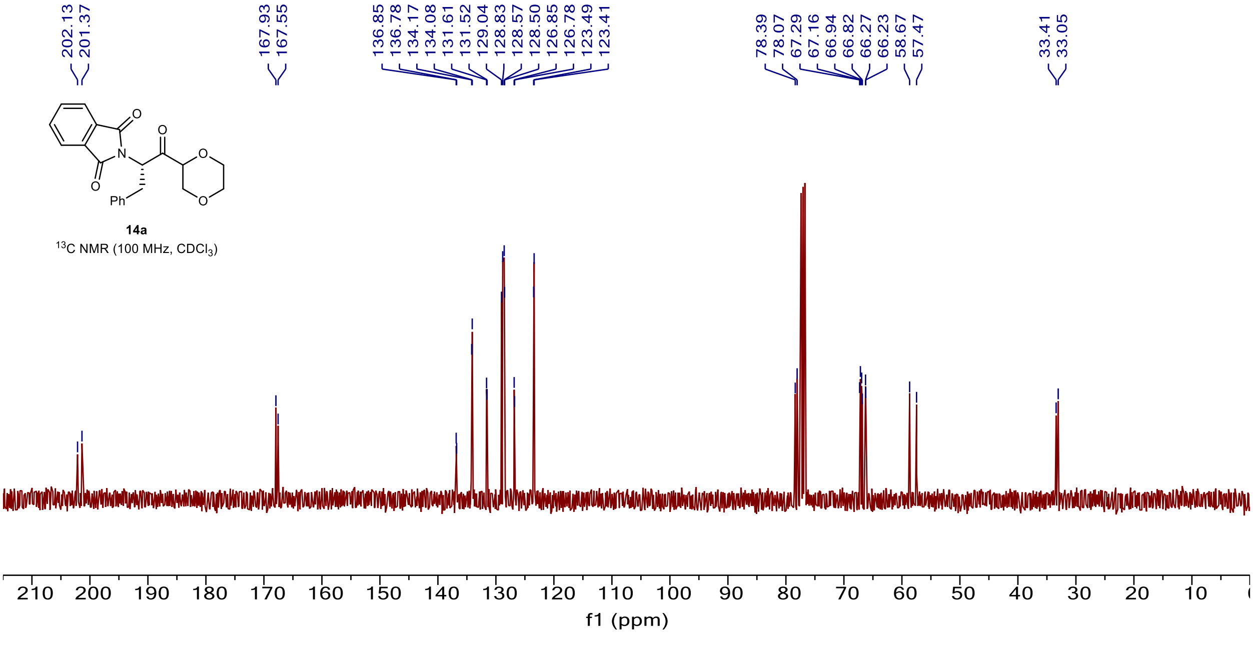

Supplementary Fig. 138. ^{13}C NMR spectrum of compound 14a.

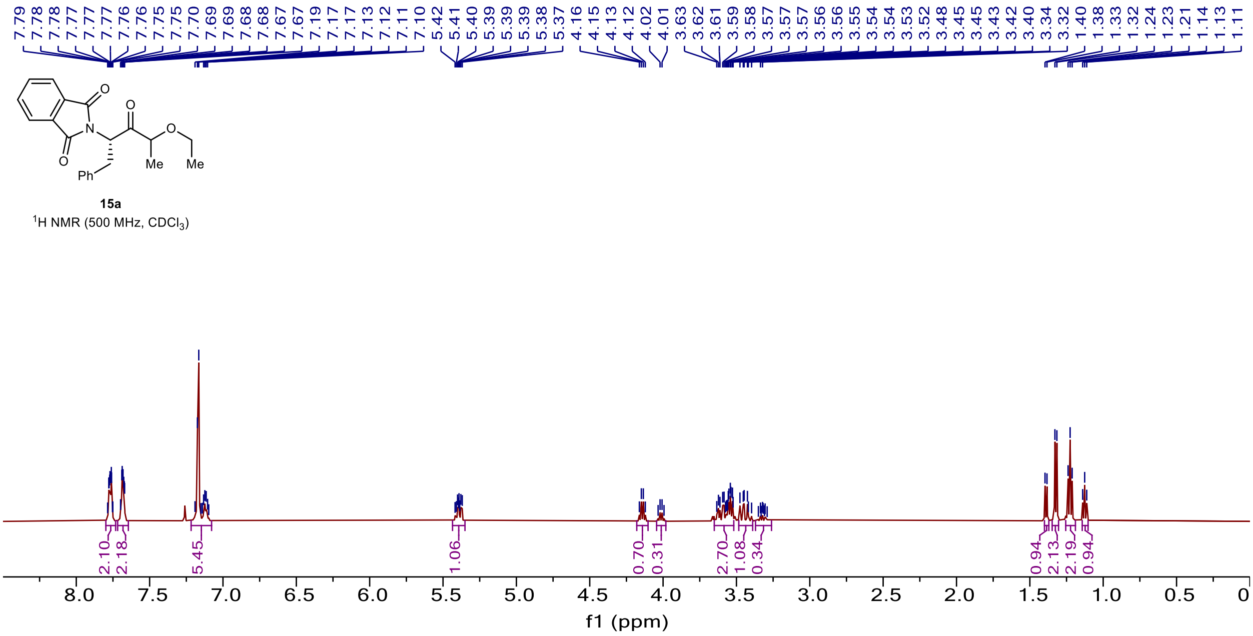

Supplementary Fig. 139. ^{1}H NMR spectrum of compound 15a.

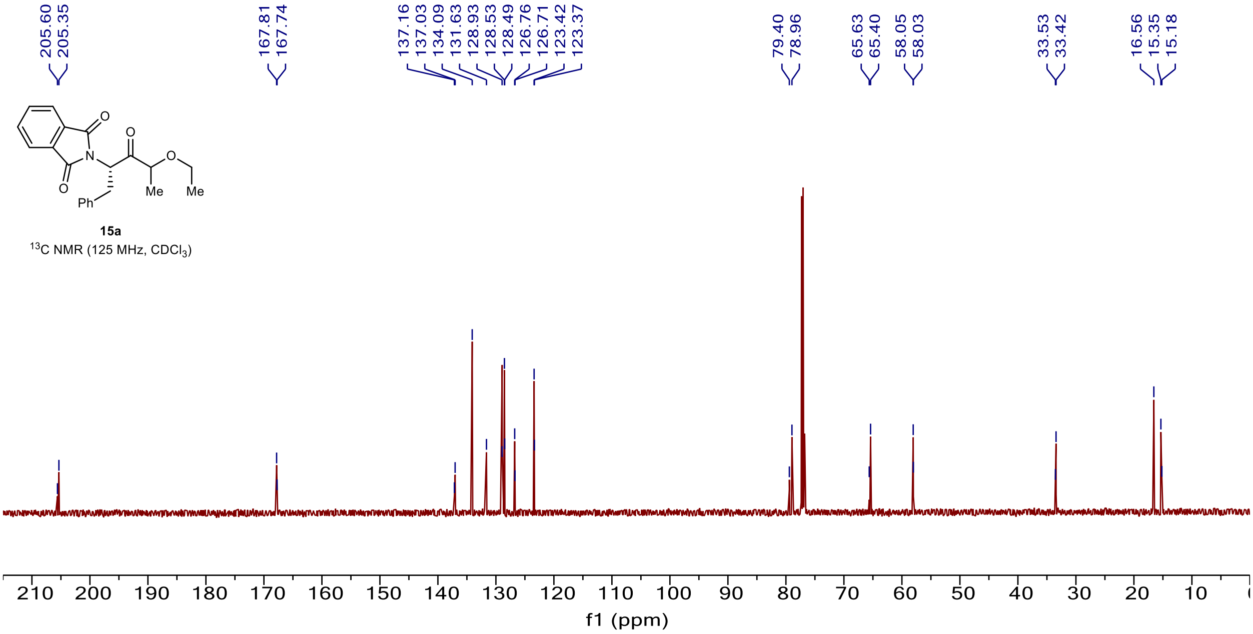

Supplementary Fig. 140. ^{13}C NMR spectrum of compound 15a.

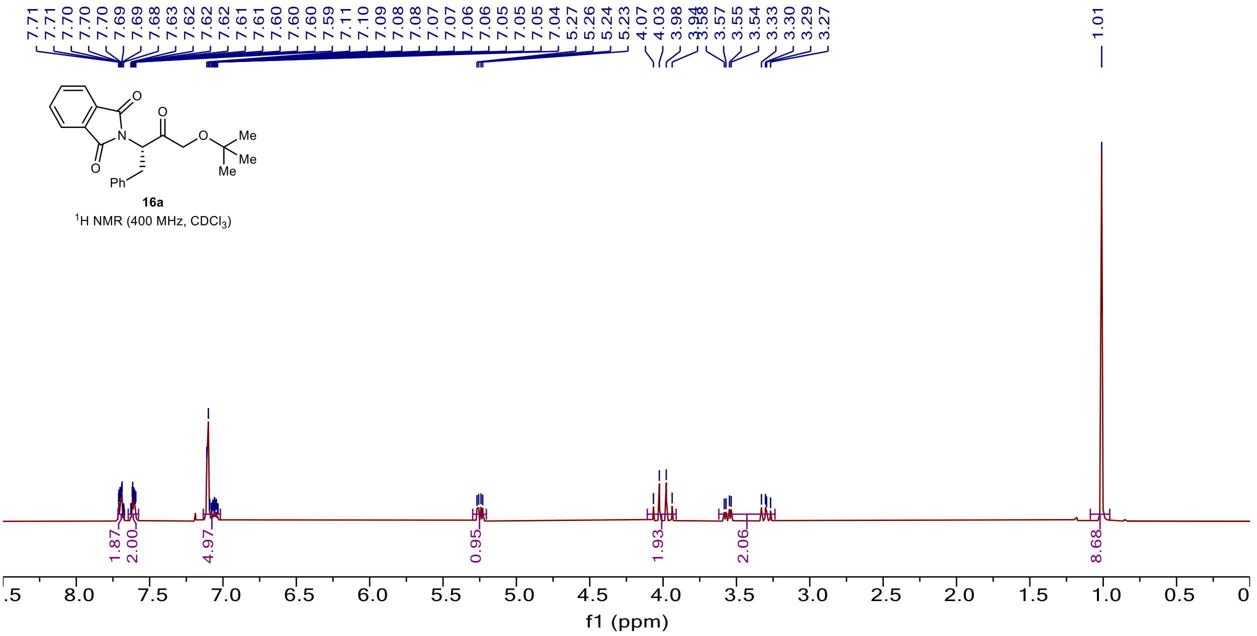

Supplementary Fig. 141. ^{1}H NMR spectrum of compound 16a.

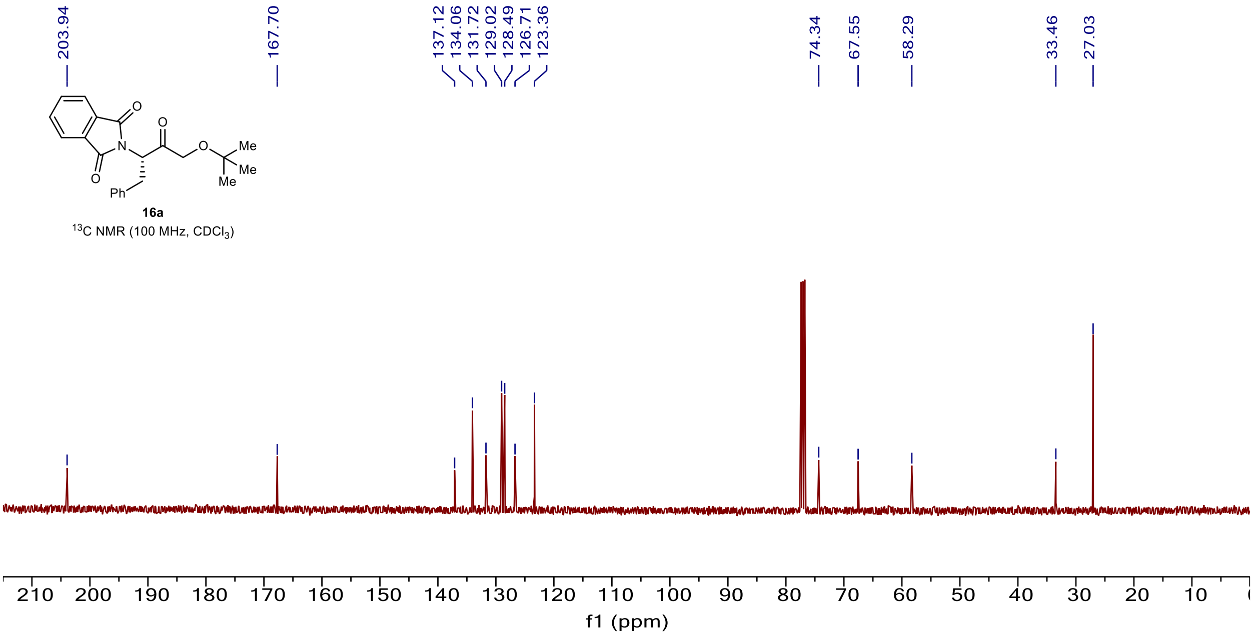

Supplementary Fig. 142. ^{13}C NMR spectrum of compound 16a.

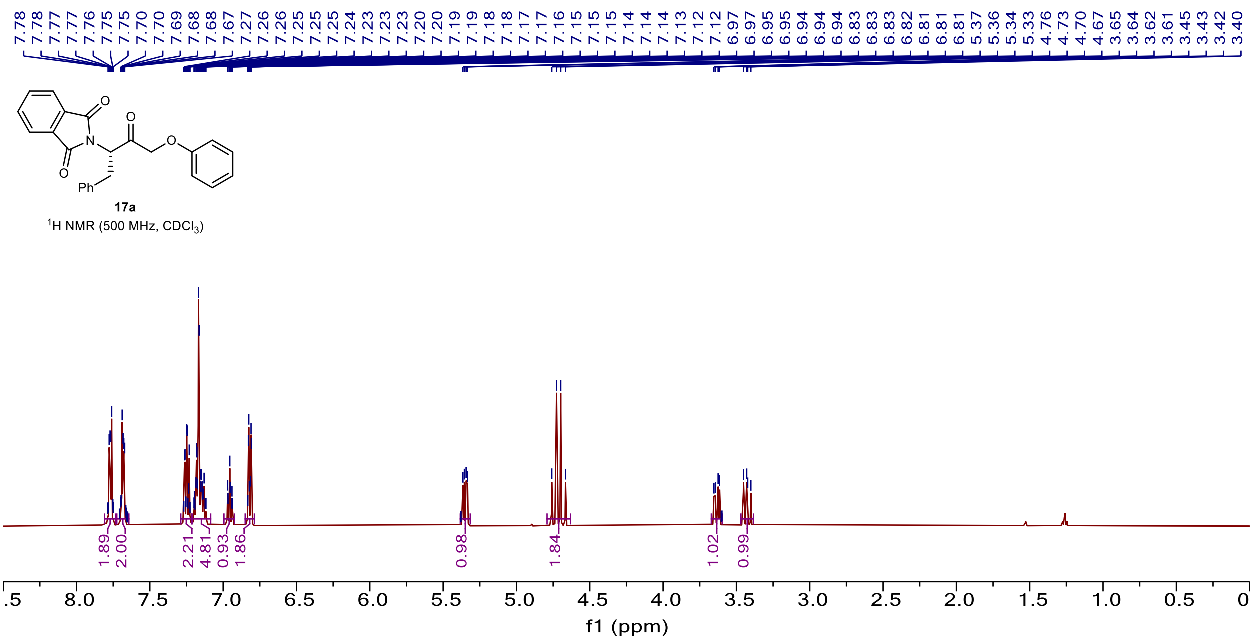

Supplementary Fig. 143. ^{1}H NMR spectrum of compound 17a.

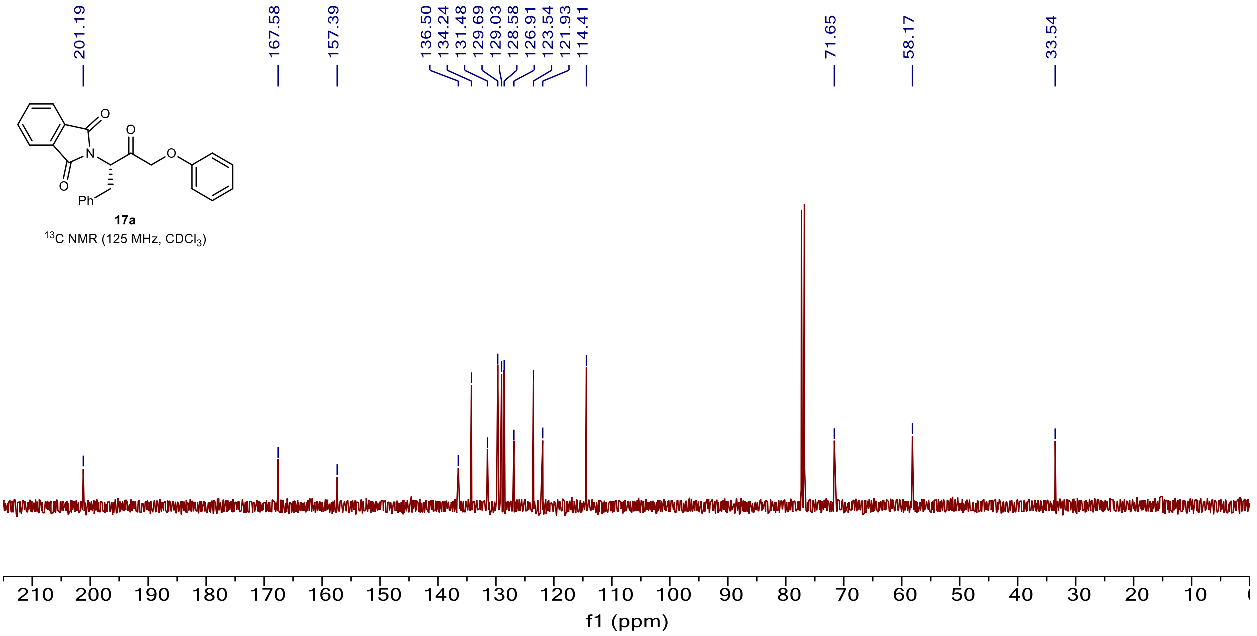

Supplementary Fig. 144. ^{13}C NMR spectrum of compound 17a.

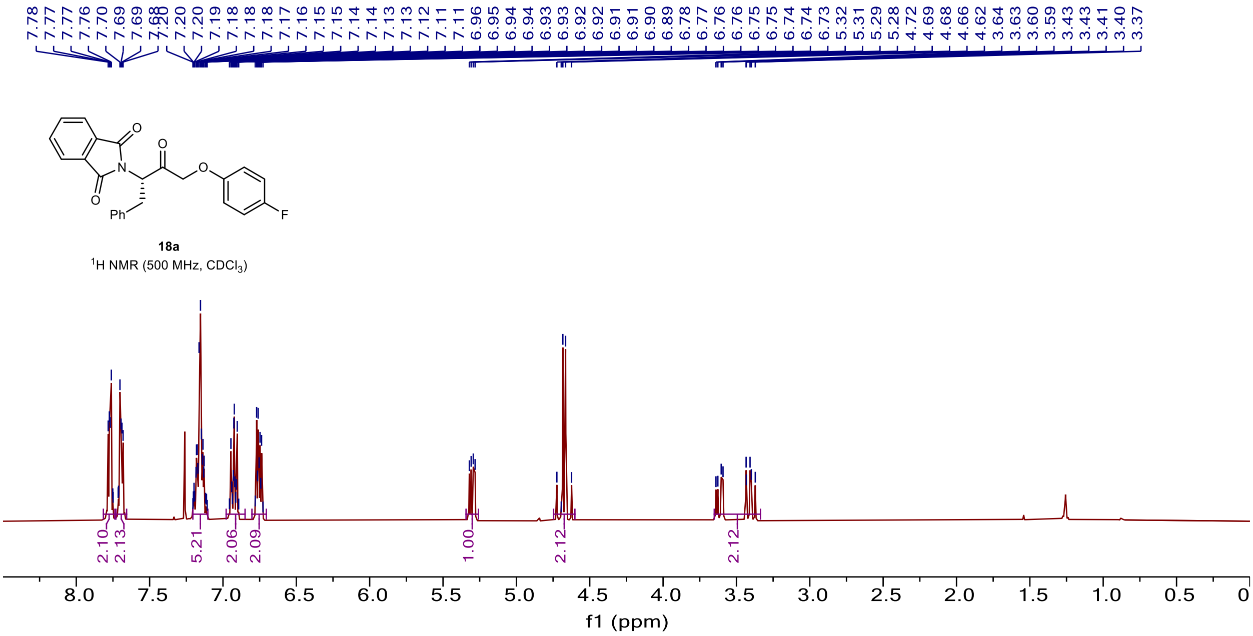

Supplementary Fig. 145. ^{1}**H NMR spectrum of compound 18a.**

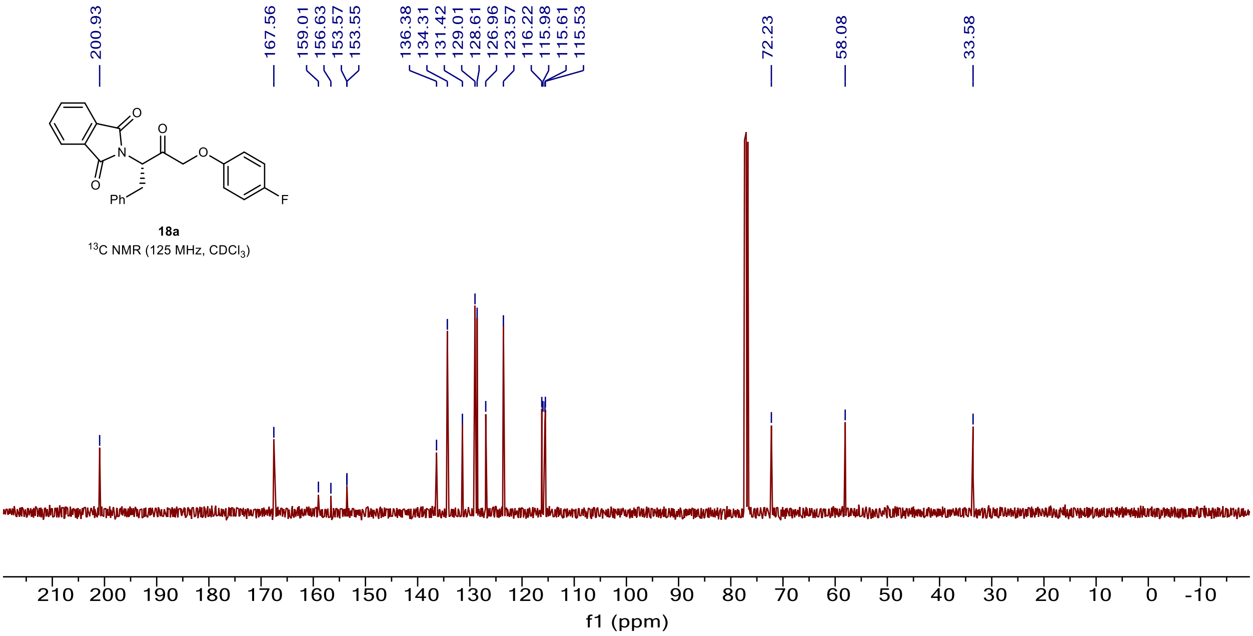

Supplementary Fig. 146. ^{13}**C NMR spectrum of compound 18a.**

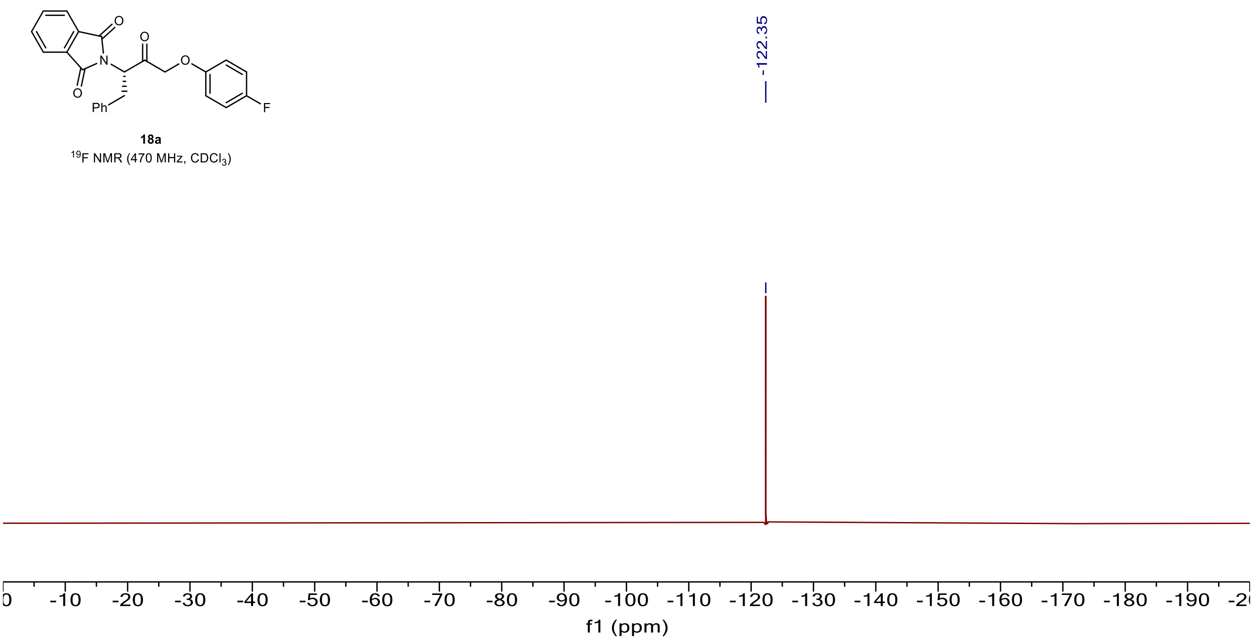

Supplementary Fig. 147. ^{19}F NMR spectrum of compound 18a.

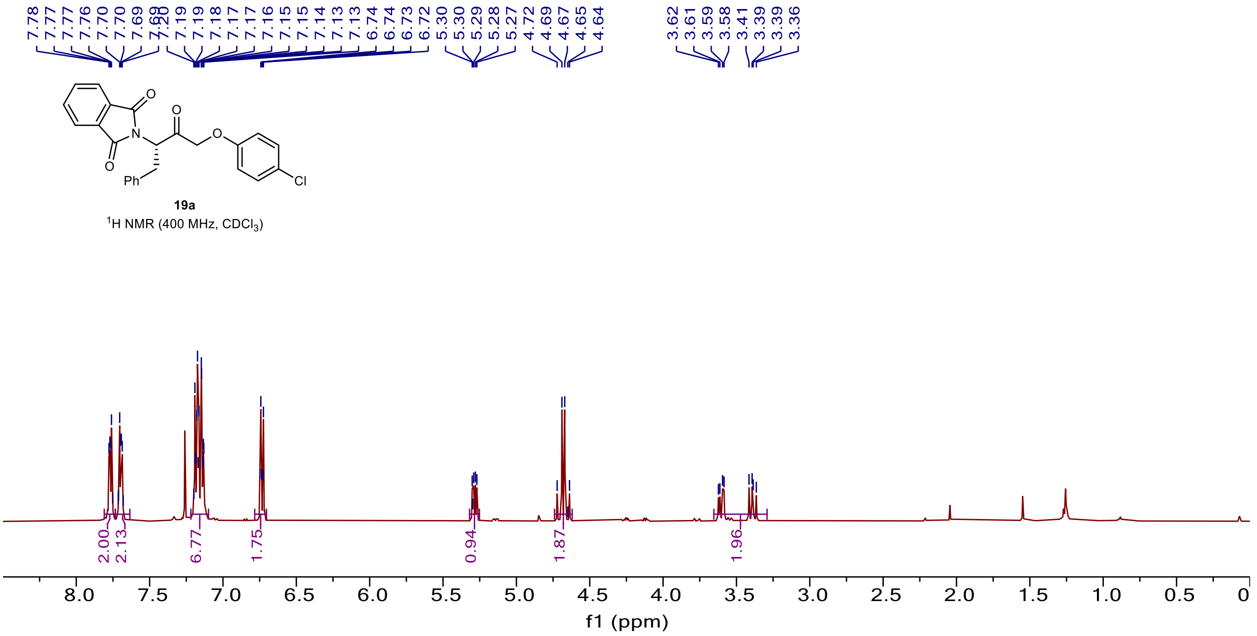

Supplementary Fig. 148. ^{1}H NMR spectrum of compound 19a.

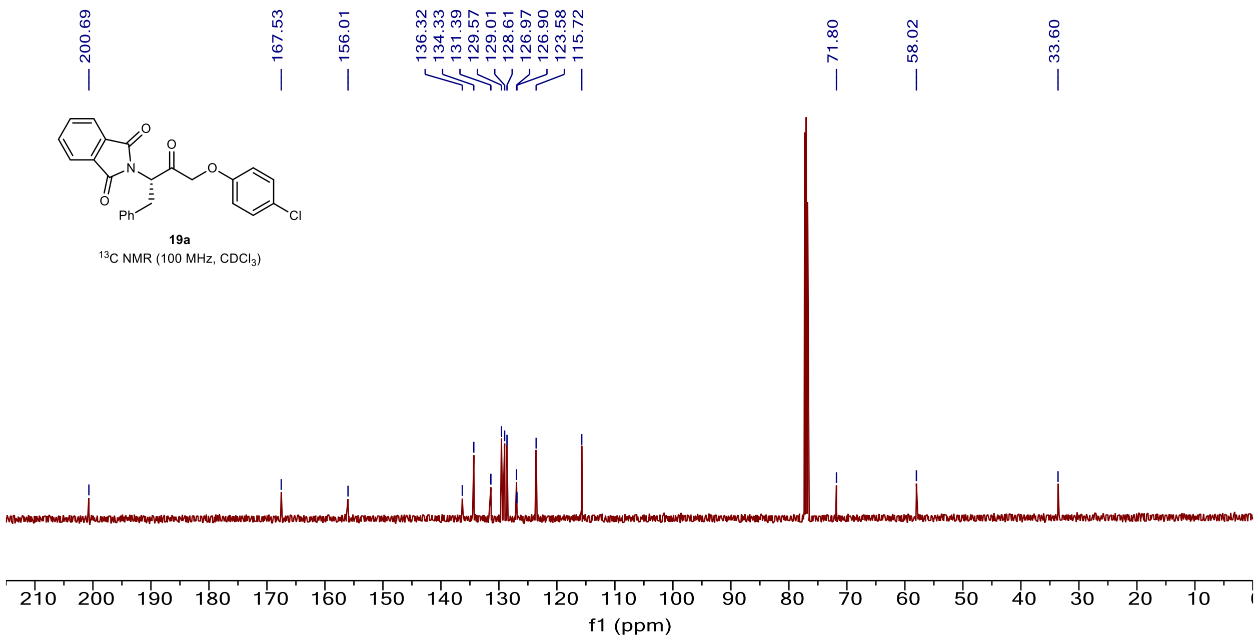

Supplementary Fig. 149. ^{13}C NMR spectrum of compound 19a.

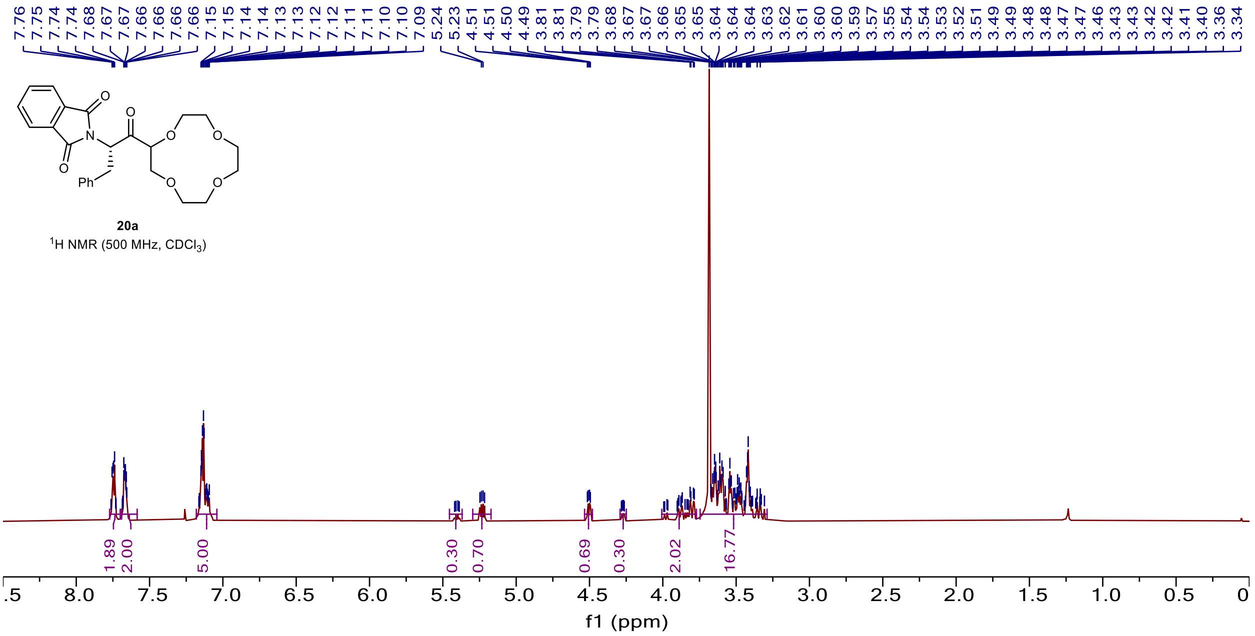

Supplementary Fig. 150. ^{1}H NMR spectrum of compound 20a.

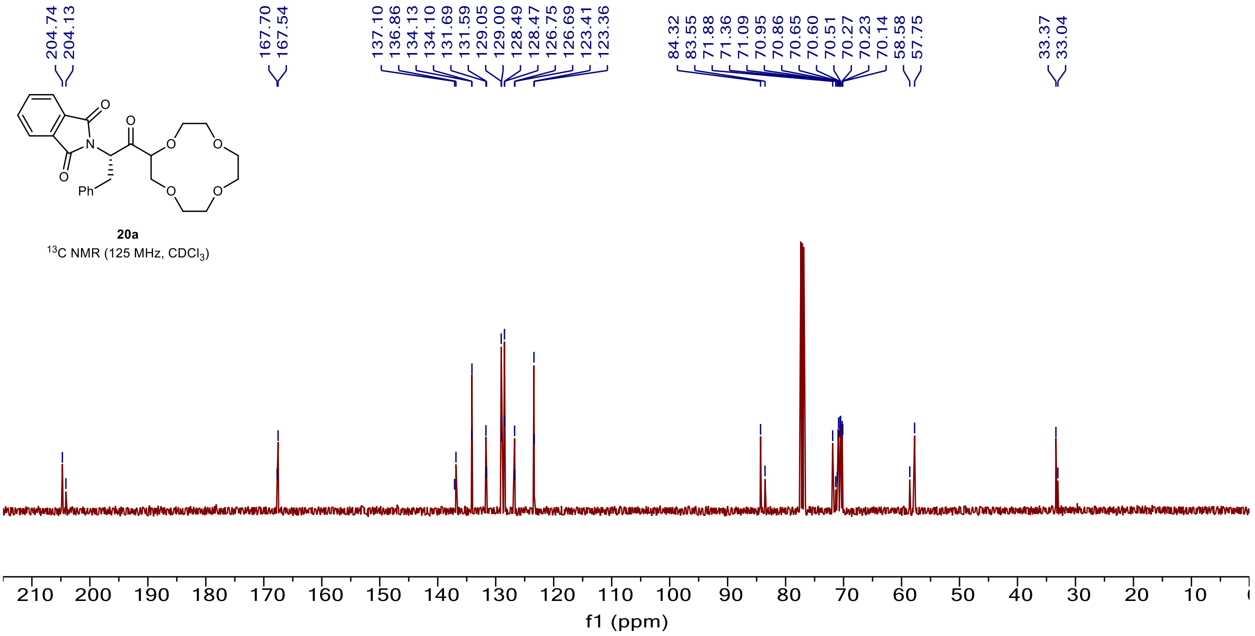

Supplementary Fig. 151. ^{13}C NMR spectrum of compound 20a.

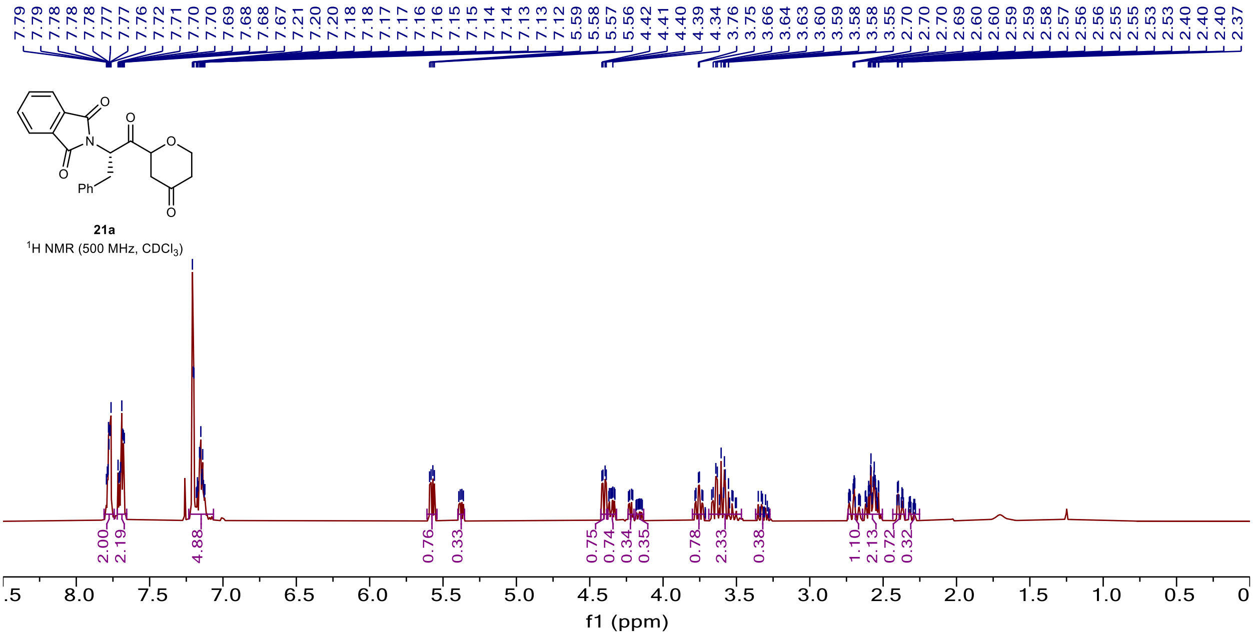

Supplementary Fig. 152. ^{1}H NMR spectrum of compound 21a.

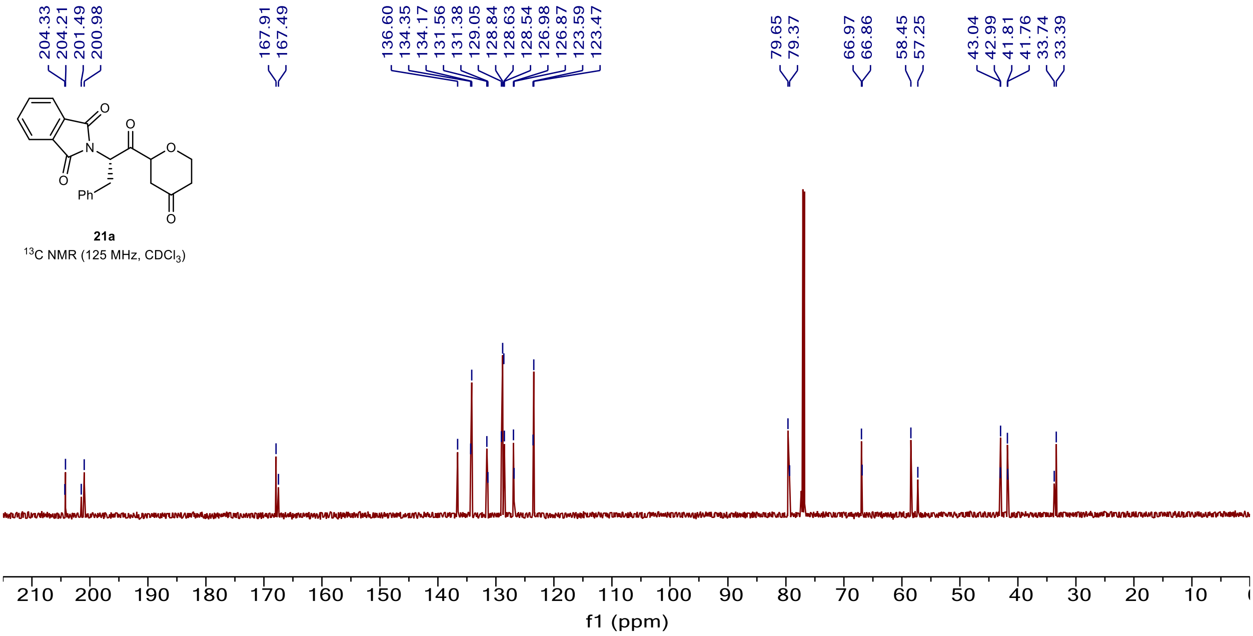

Supplementary Fig. 153. ^{13}C NMR spectrum of compound 21a.

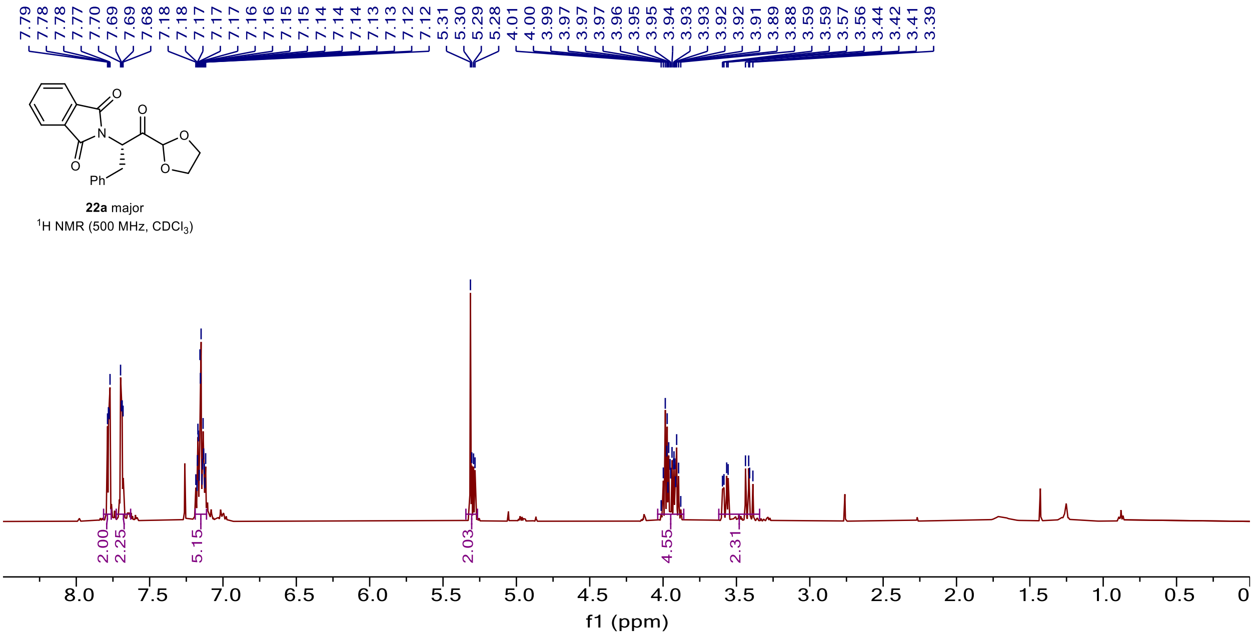

Supplementary Fig. 154. ^{1}H NMR spectrum of compound 22a.

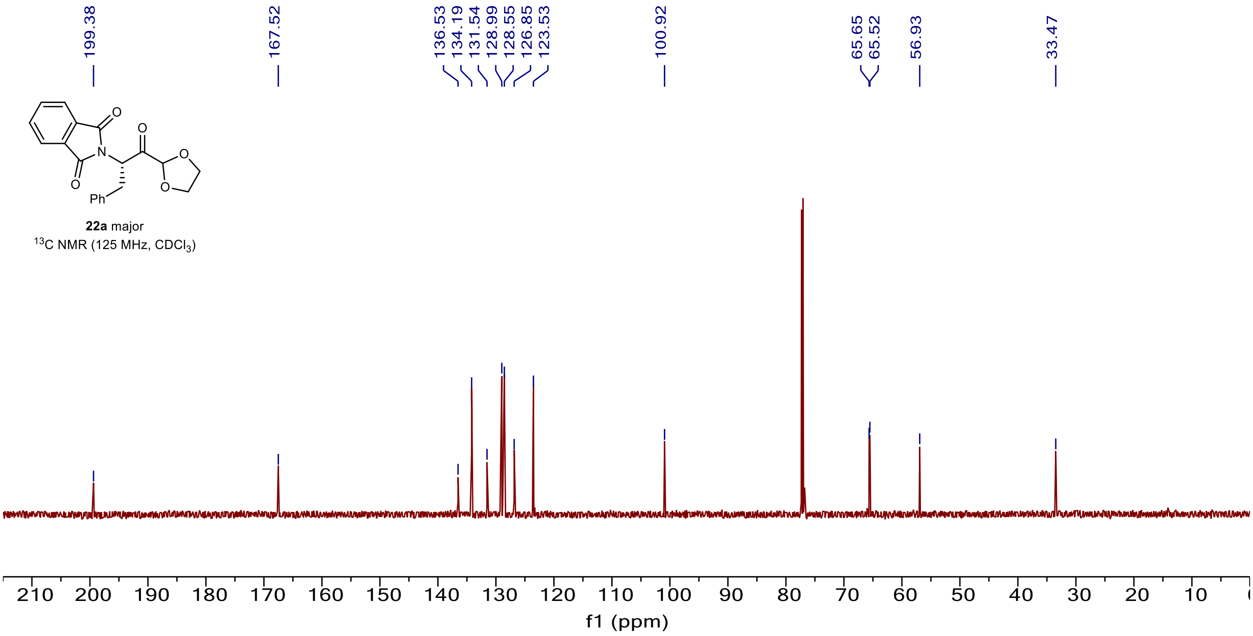

Supplementary Fig. 155. ^{1}H NMR spectrum of compound 22a.

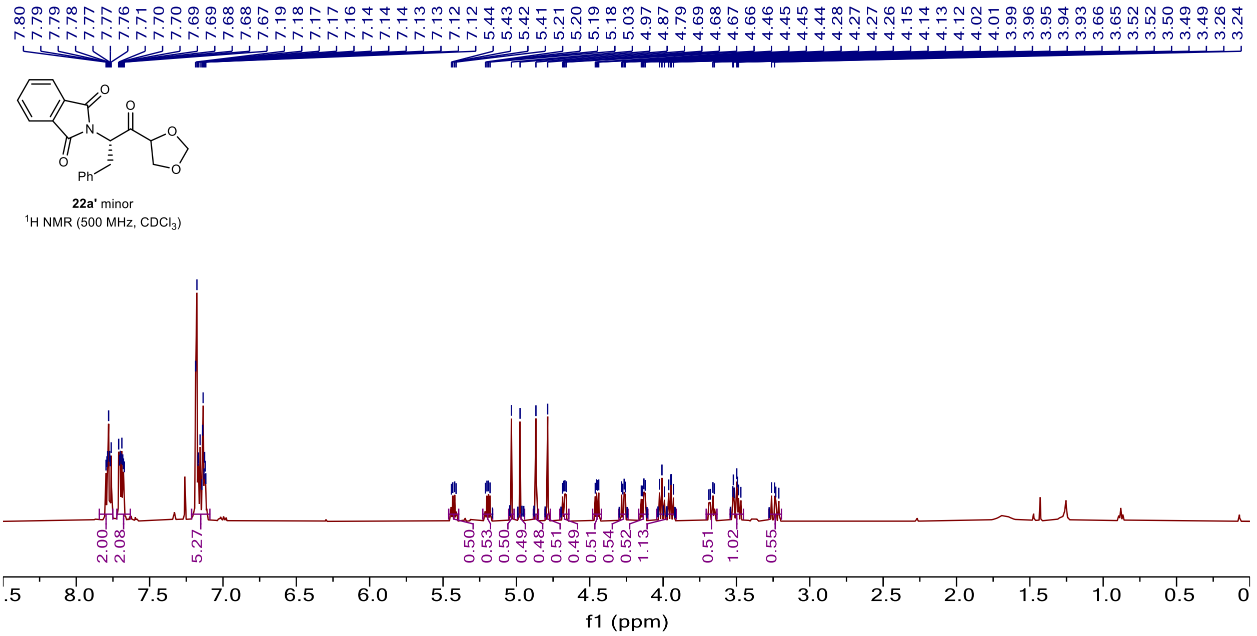

Supplementary Fig. 156. ^{1}H NMR spectrum of compound 22a'.

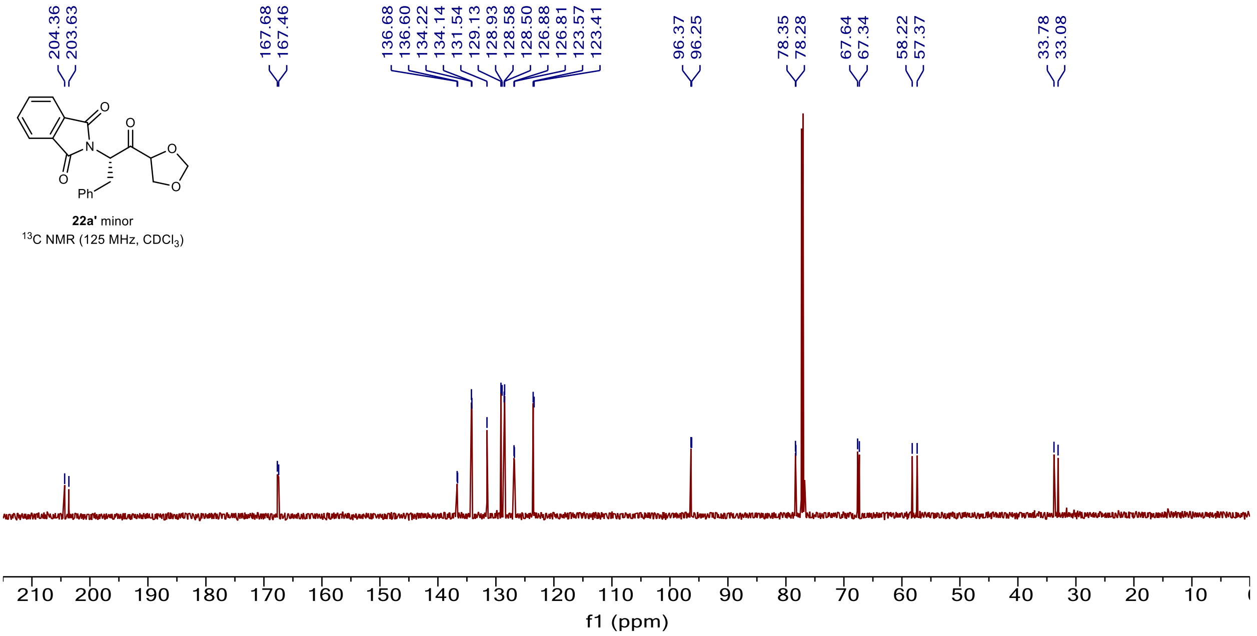

Supplementary Fig. 157. ^{13}C NMR spectrum of compound 22a'.

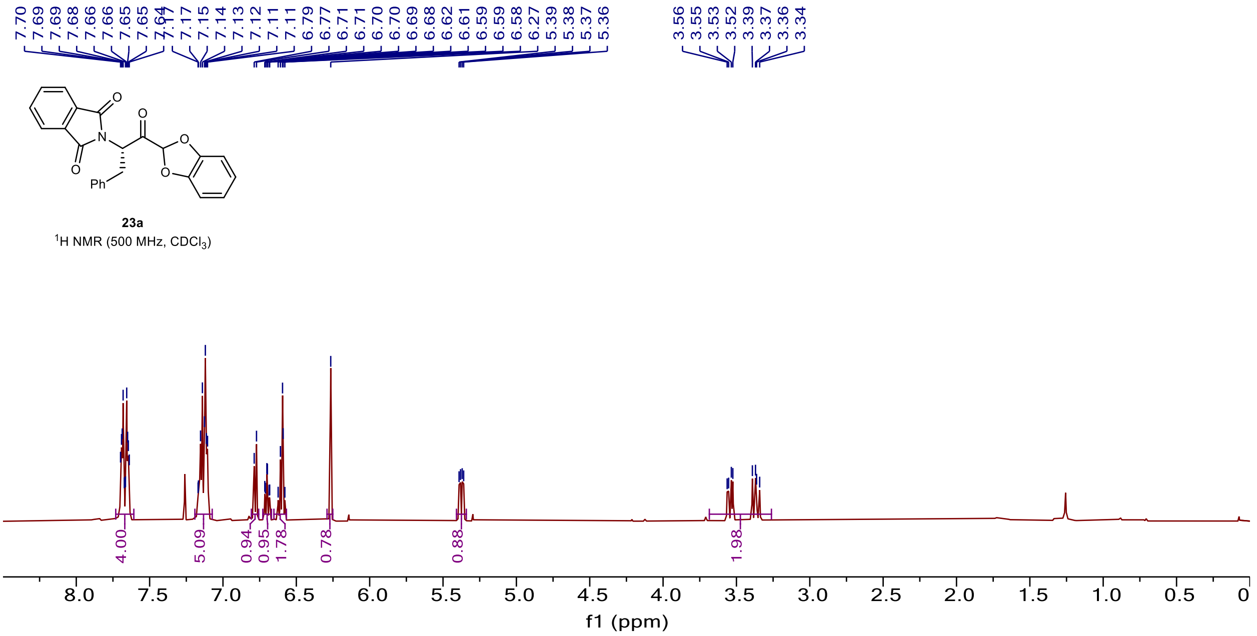

Supplementary Fig. 158. ^{1}H NMR spectrum of compound 23a.

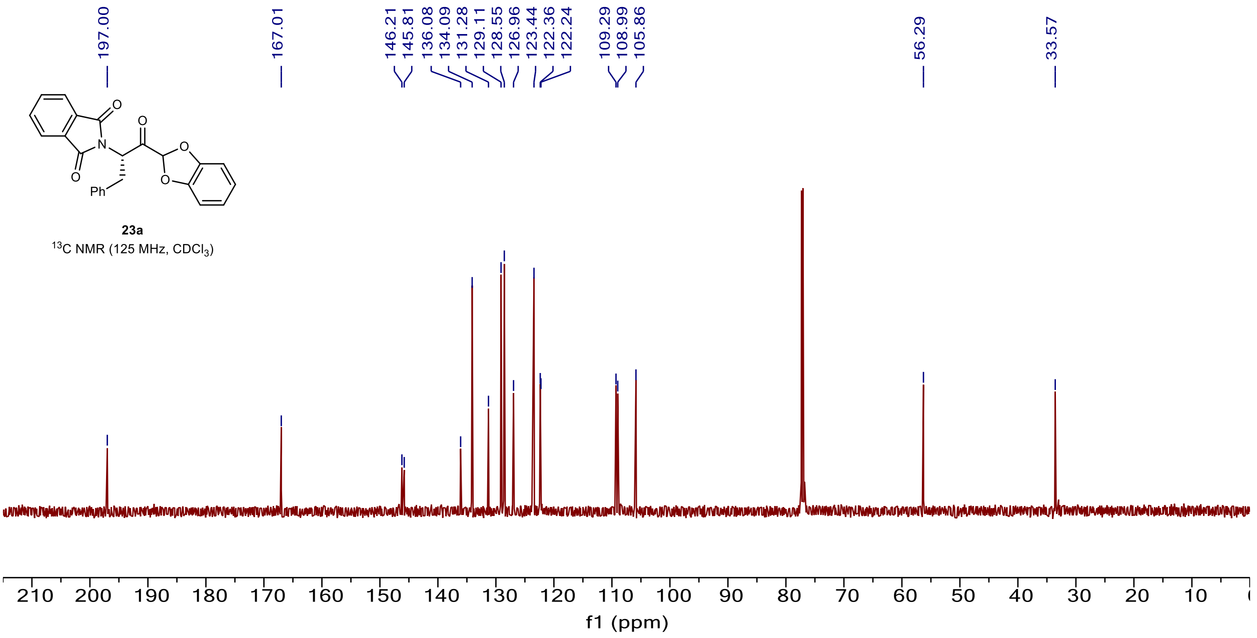

Supplementary Fig. 159. ^{13}C NMR spectrum of compound 23a.

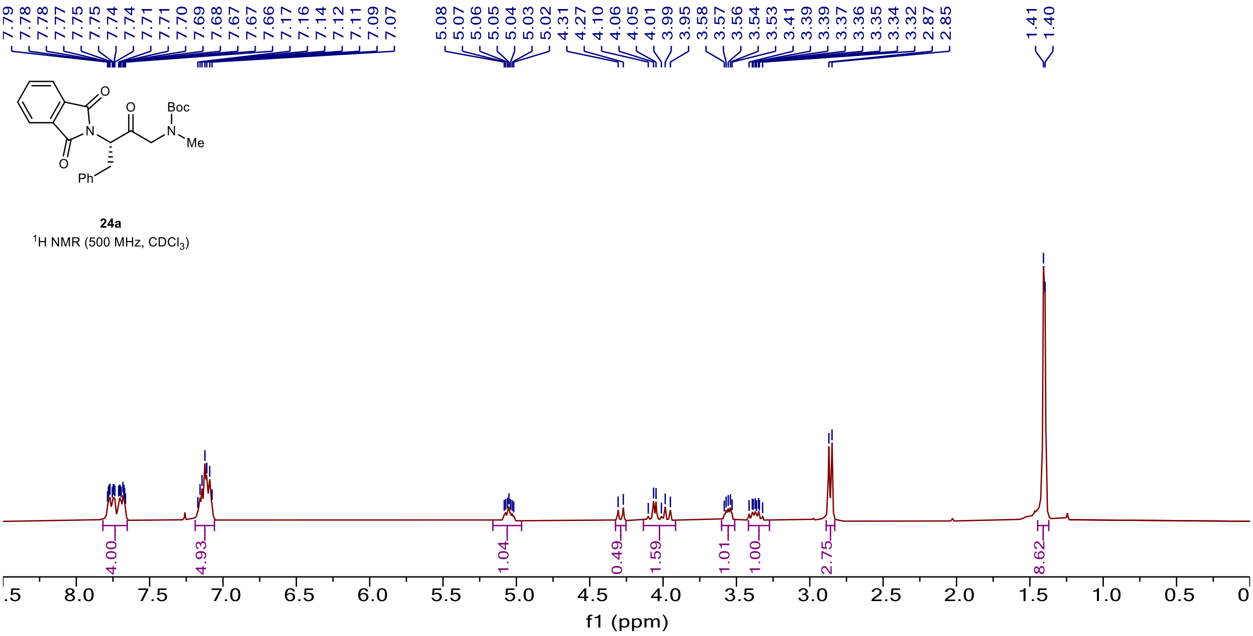

Supplementary Fig. 160. ^{1}H NMR spectrum of compound 24a.

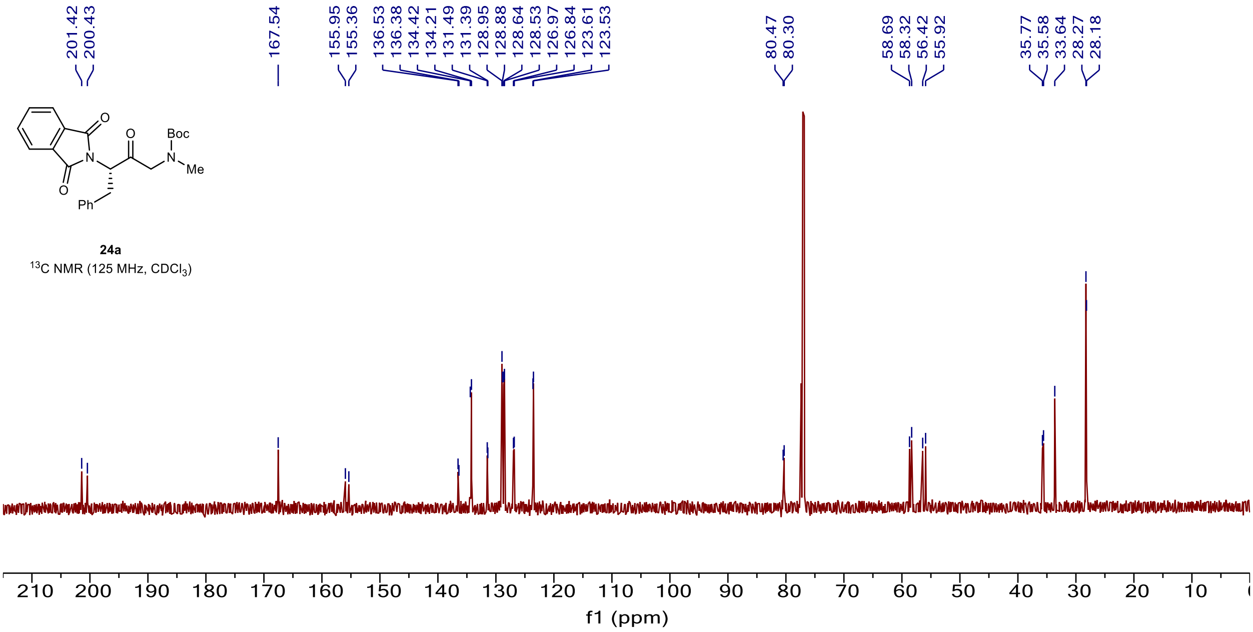

Supplementary Fig. 161. ^{13}C NMR spectrum of compound 24a.

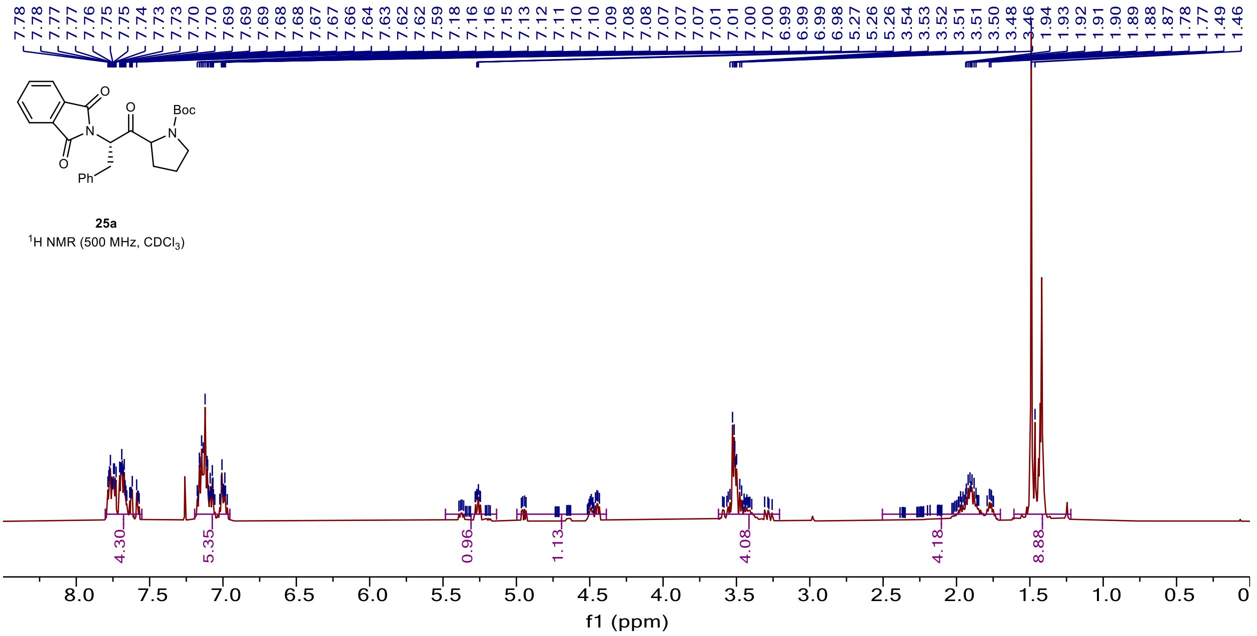

Supplementary Fig. 162. ^{1}H NMR spectrum of compound 25a.

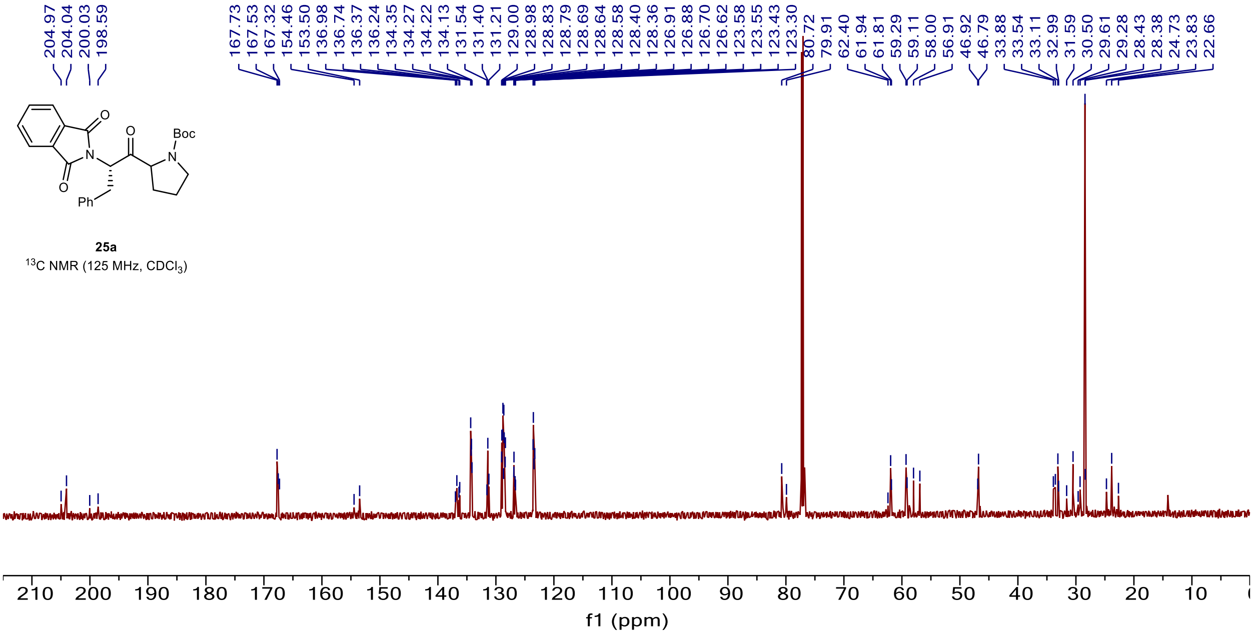

Supplementary Fig. 163. ^{13}C NMR spectrum of compound 25a.

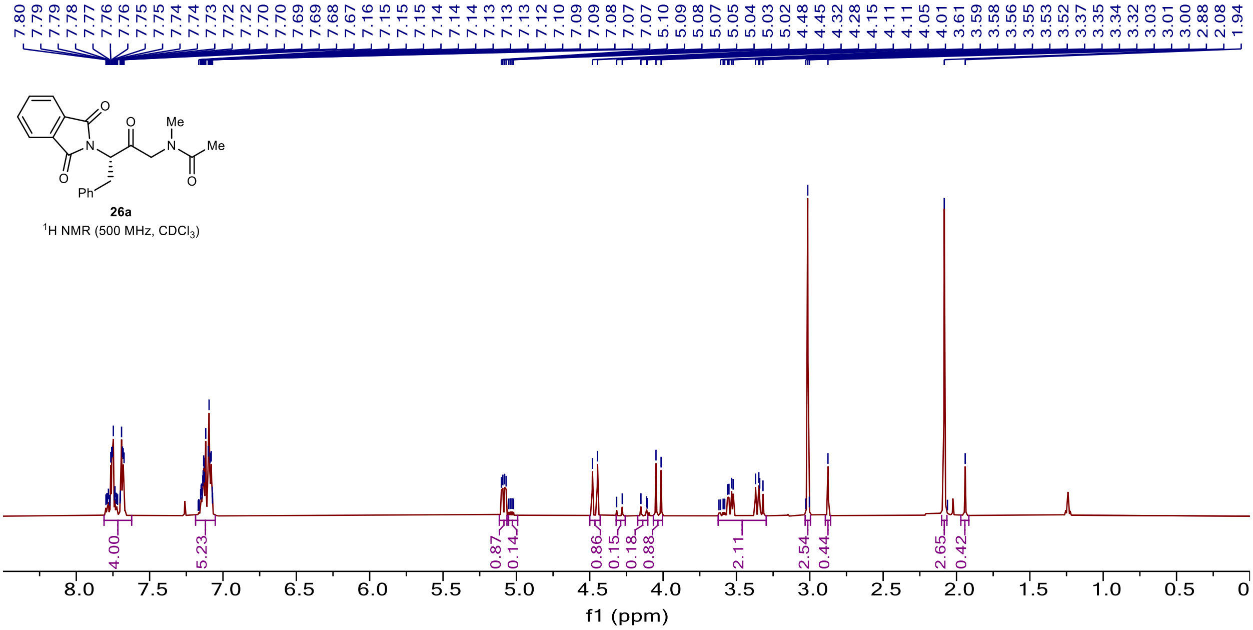

Supplementary Fig. 164. ^{1}H NMR spectrum of compound 26a.

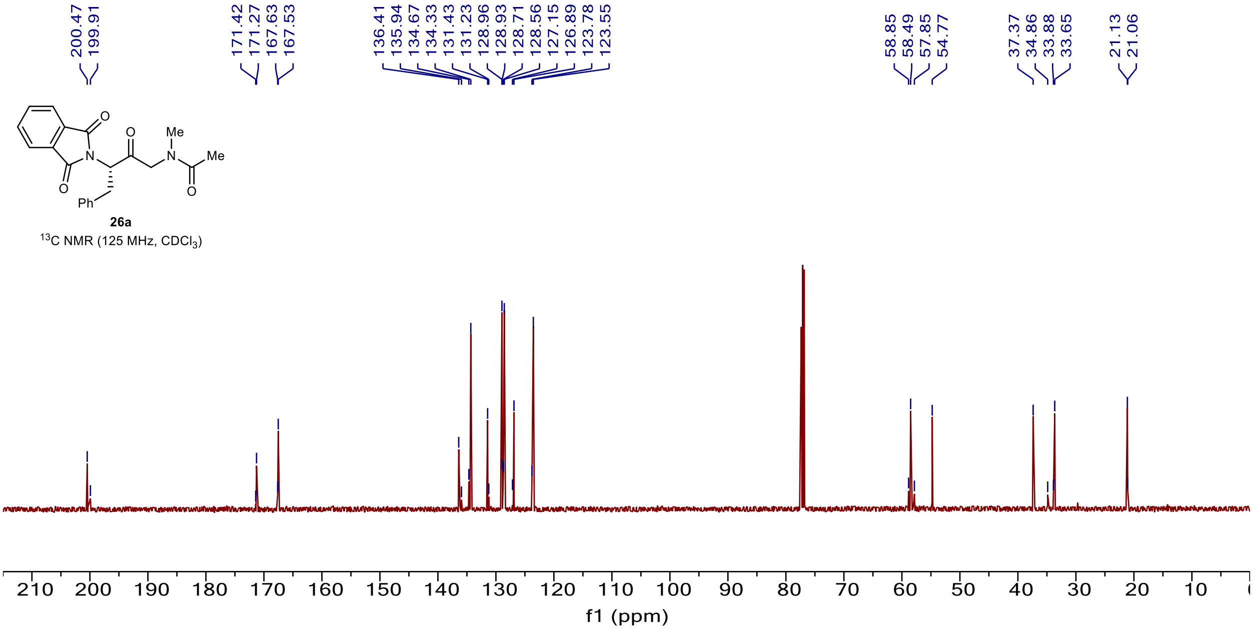

Supplementary Fig. 165. ^{13}C NMR spectrum of compound 26a.

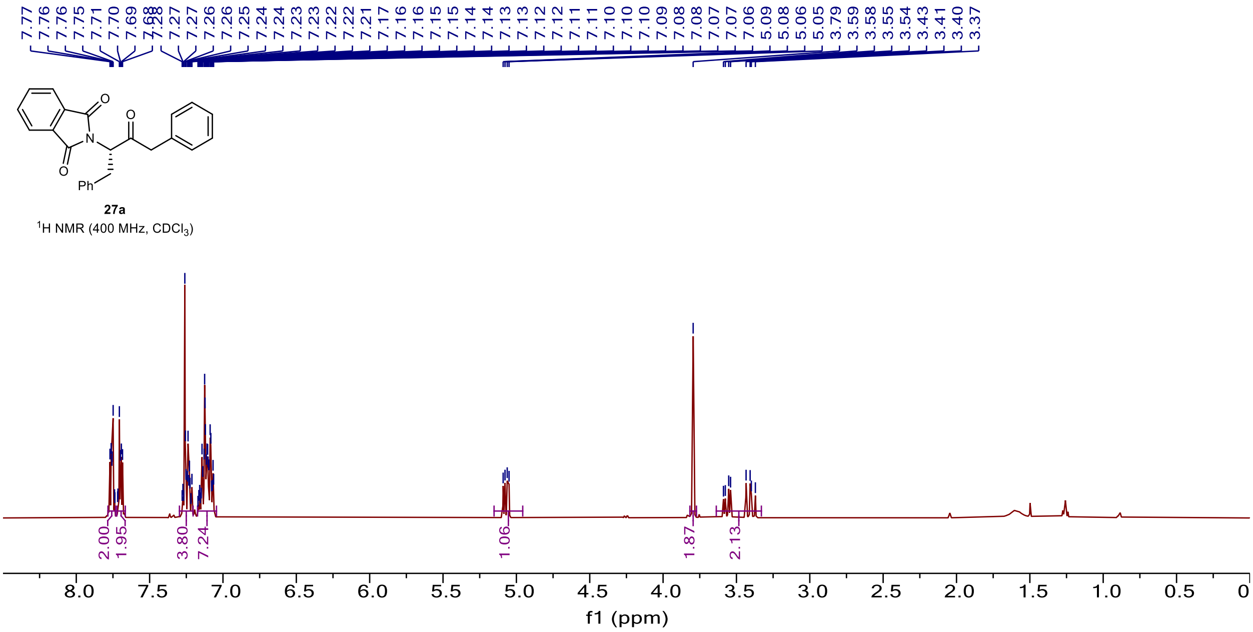

Supplementary Fig. 166. ^{1}H NMR spectrum of compound 27a.

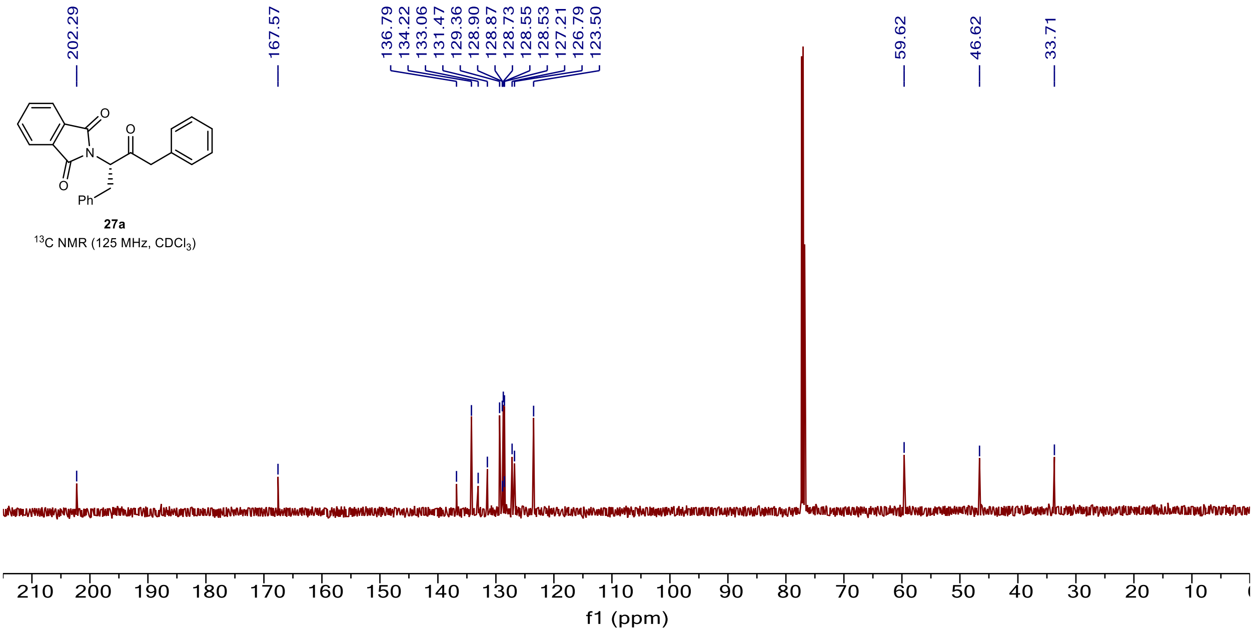

Supplementary Fig. 167. ^{13}C NMR spectrum of compound 27a.

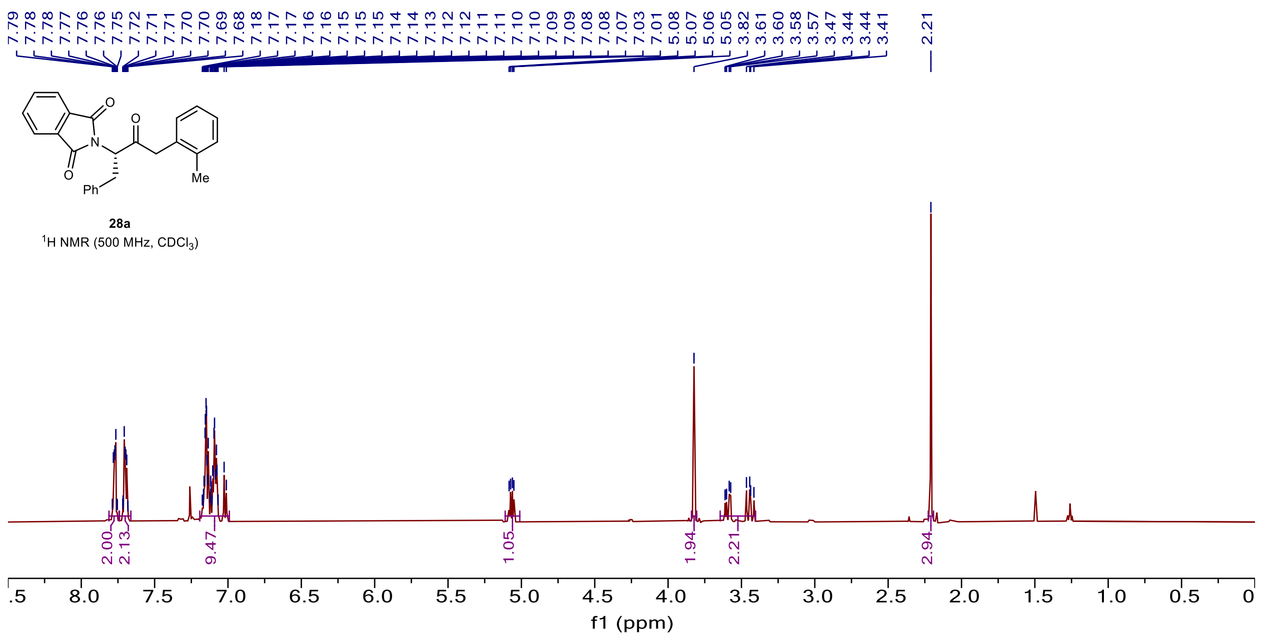

Supplementary Fig. 168. **^{1}H NMR spectrum of compound 28a.**

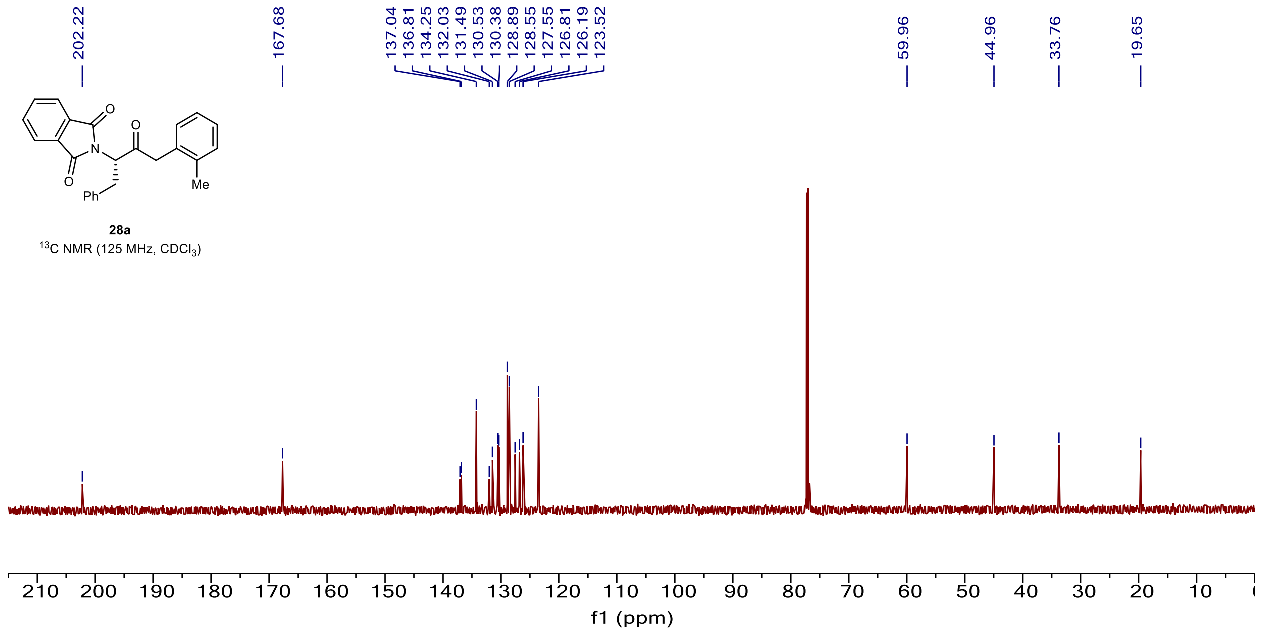

Supplementary Fig. 169. **^{13}C NMR spectrum of compound 28a.**

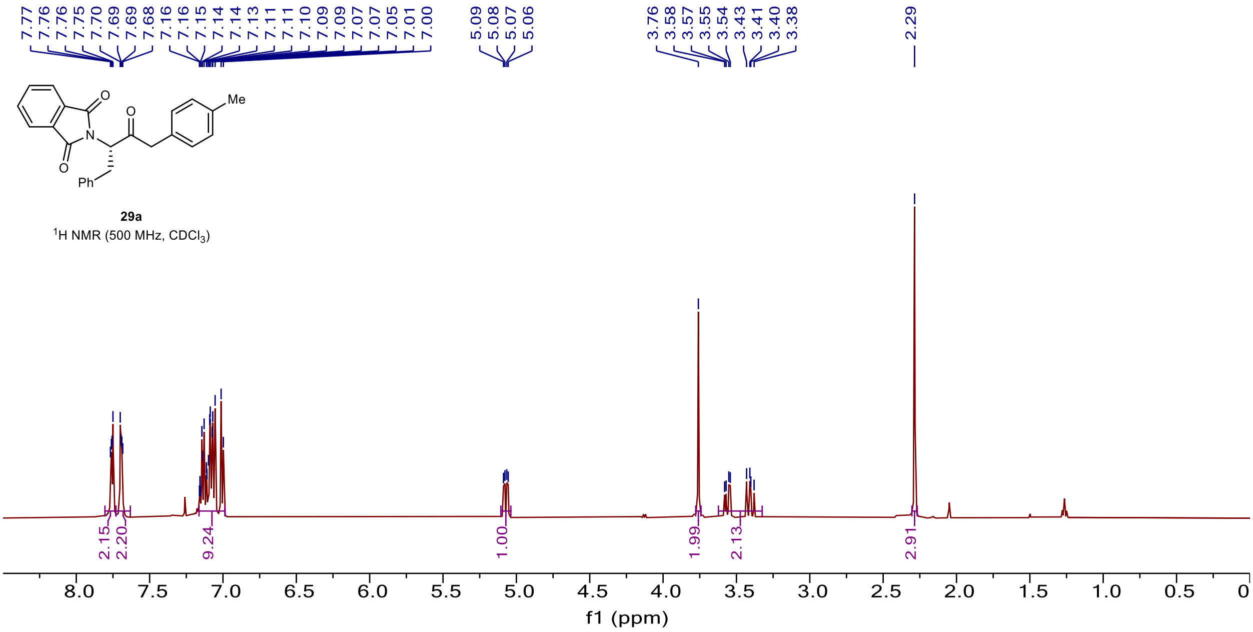

Supplementary Fig. 170. ^{1}H NMR spectrum of compound 29a.

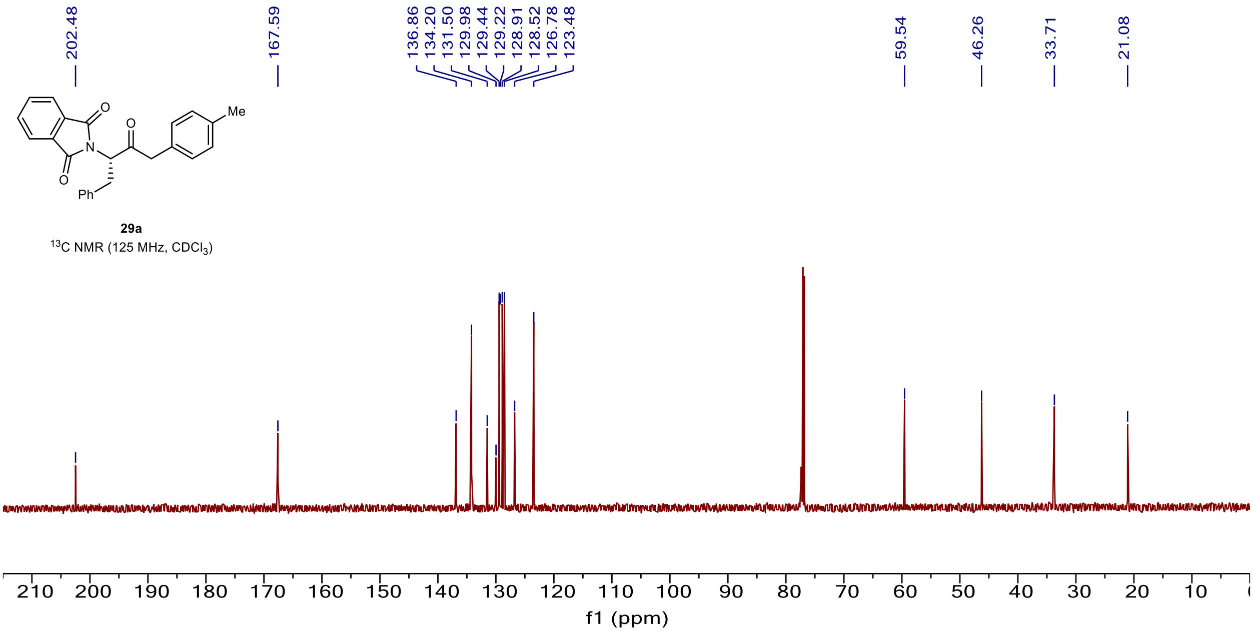

Supplementary Fig. 171. ^{13}C NMR spectrum of compound 29a.

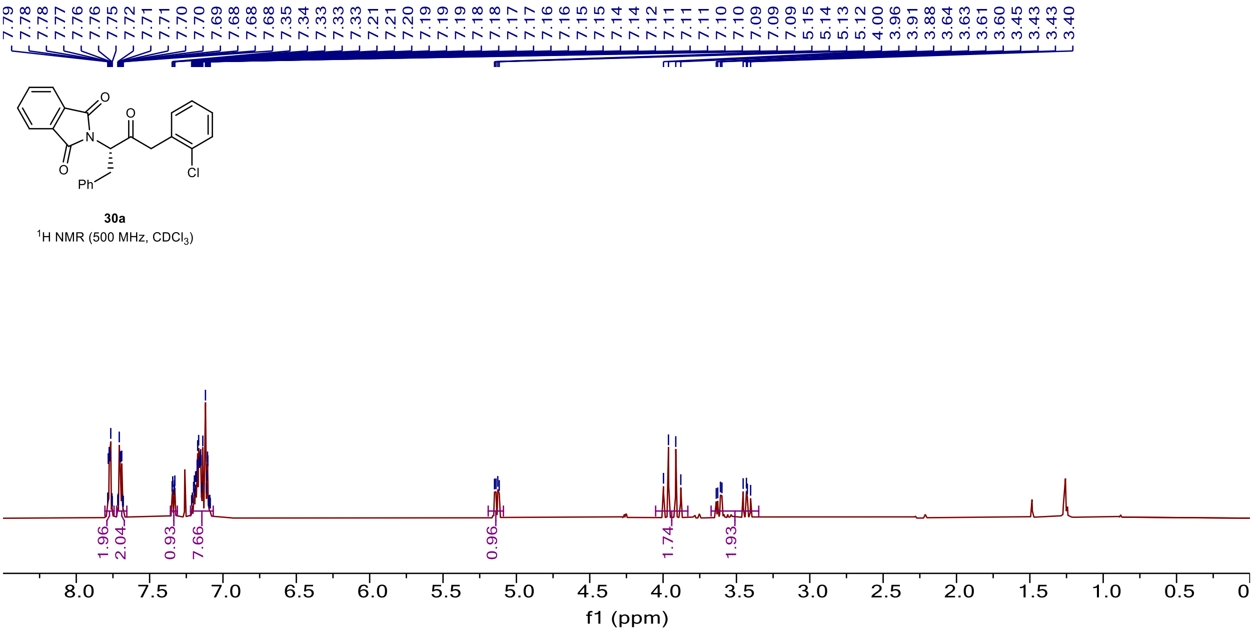

Supplementary Fig. 172. ^{1}H NMR spectrum of compound 30a.

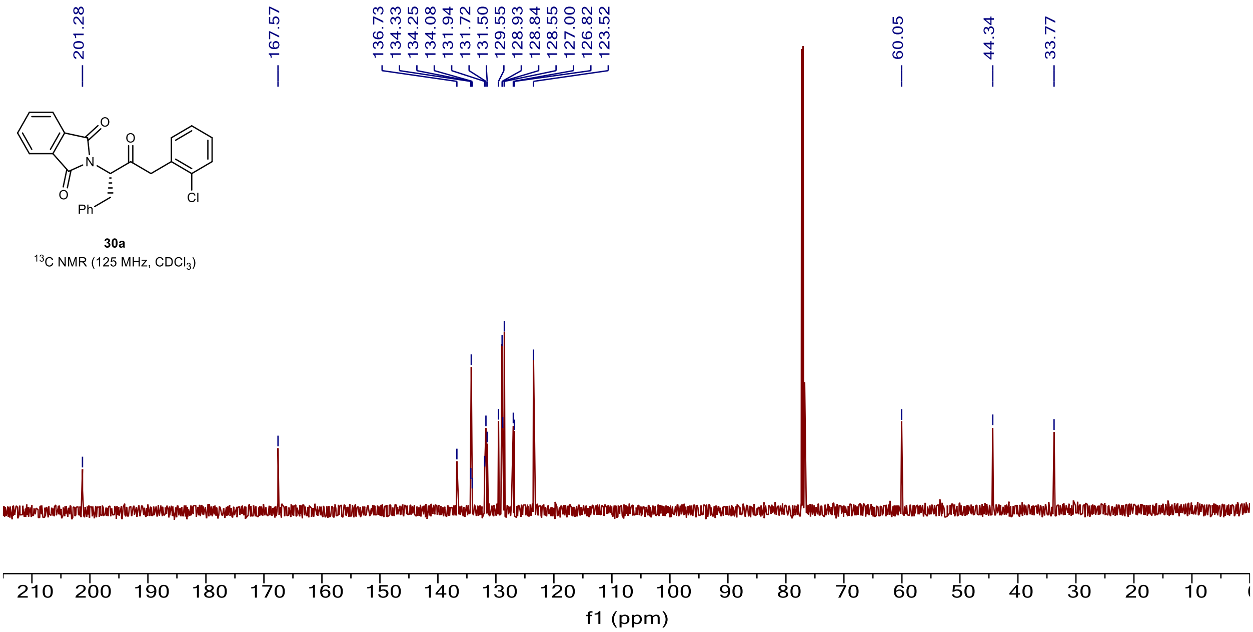

Supplementary Fig. 173. ^{13}C NMR spectrum of compound 30a.

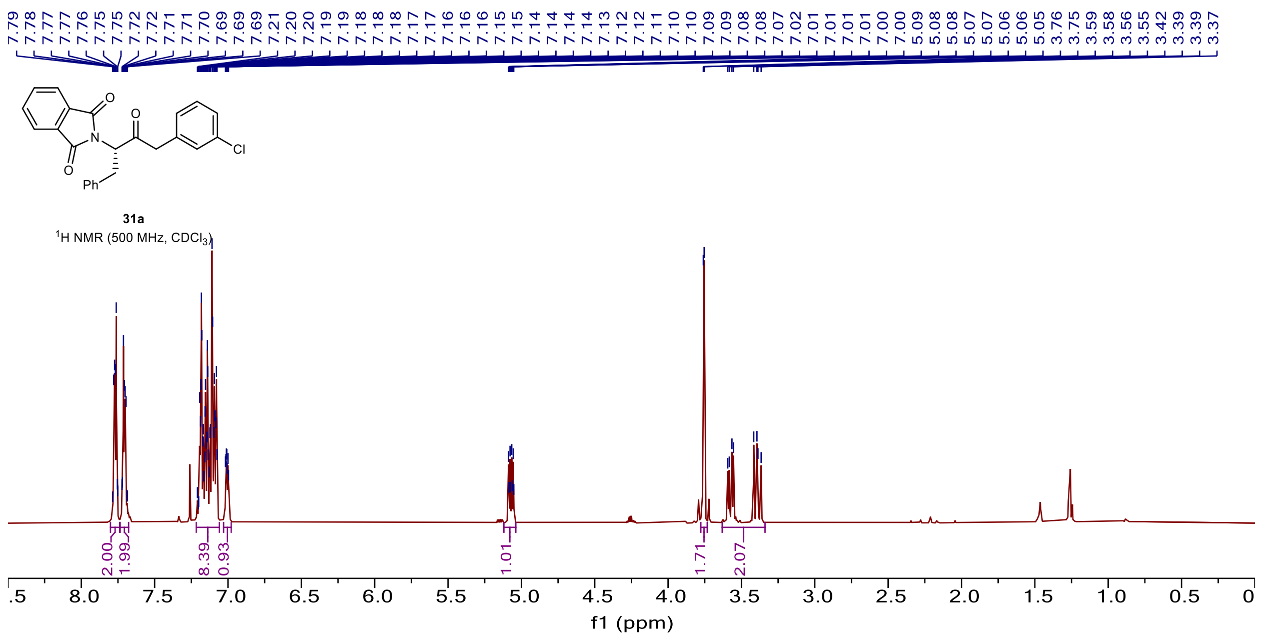

Supplementary Fig. 174. ^{1}H NMR spectrum of compound 31a.

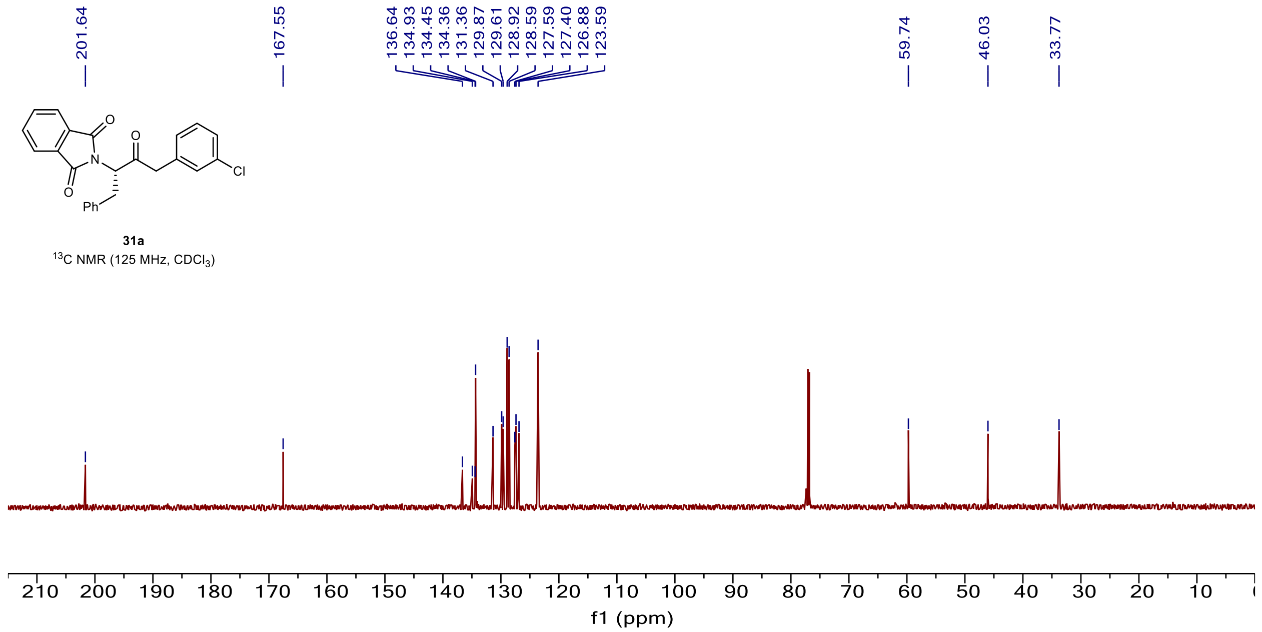

Supplementary Fig. 175. ^{13}C NMR spectrum of compound 31a.

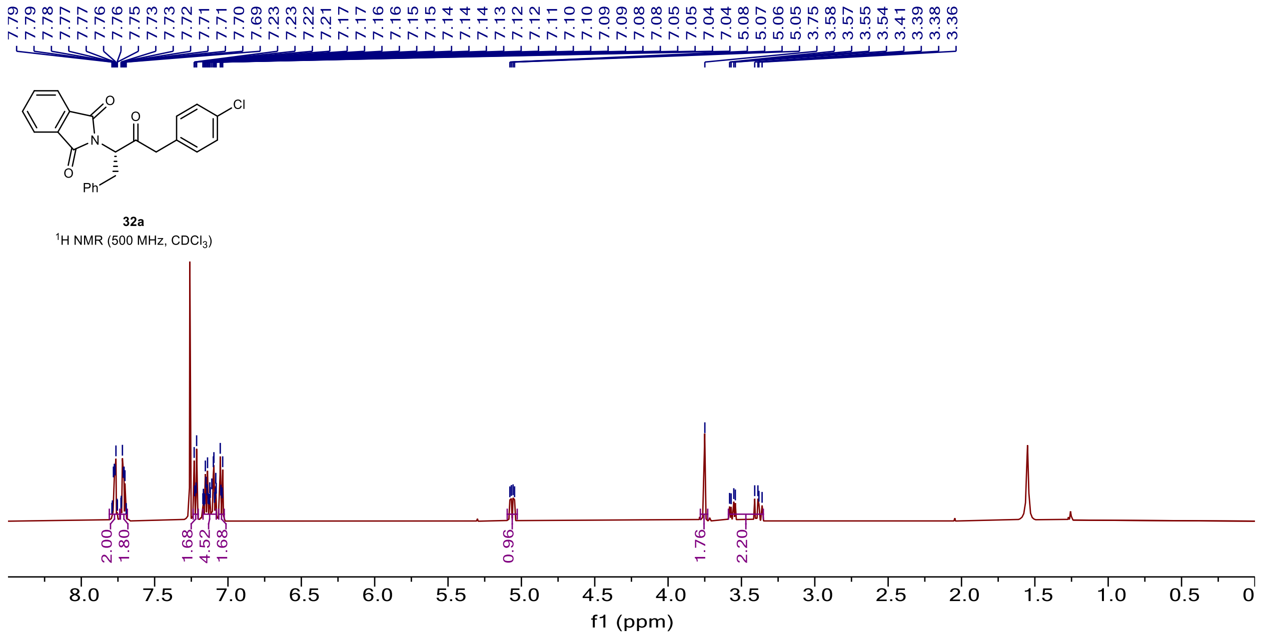

Supplementary Fig. 176. ^{1}H NMR spectrum of compound 32a.

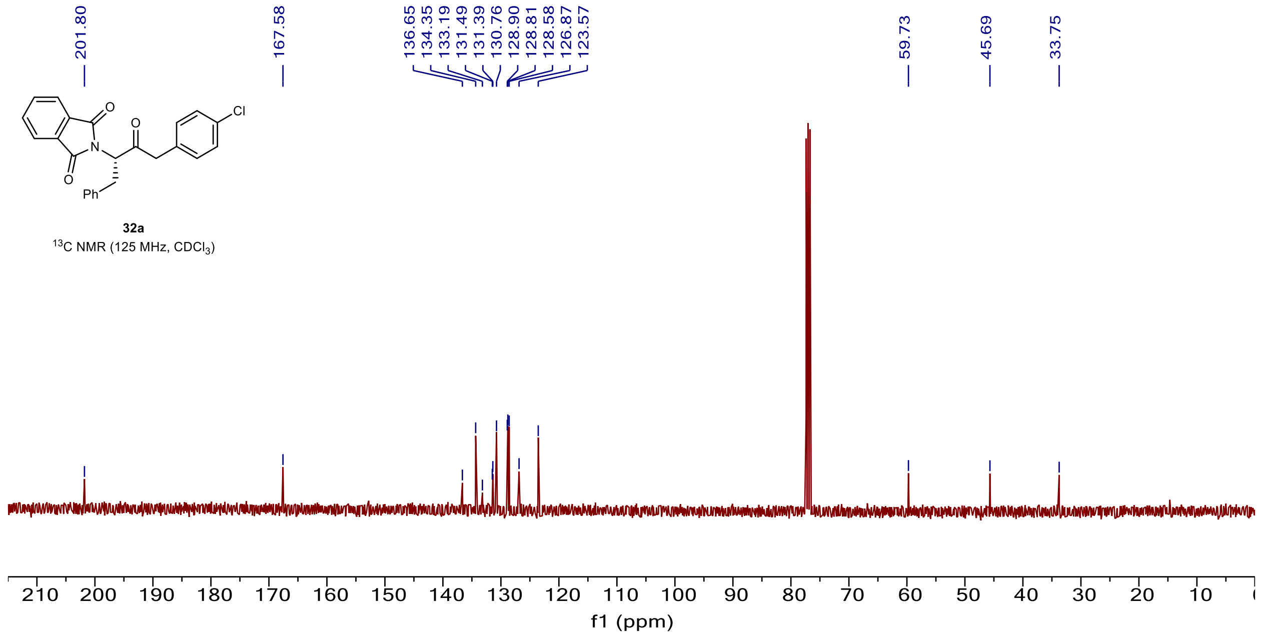

Supplementary Fig. 177. ^{13}C NMR spectrum of compound 32a.

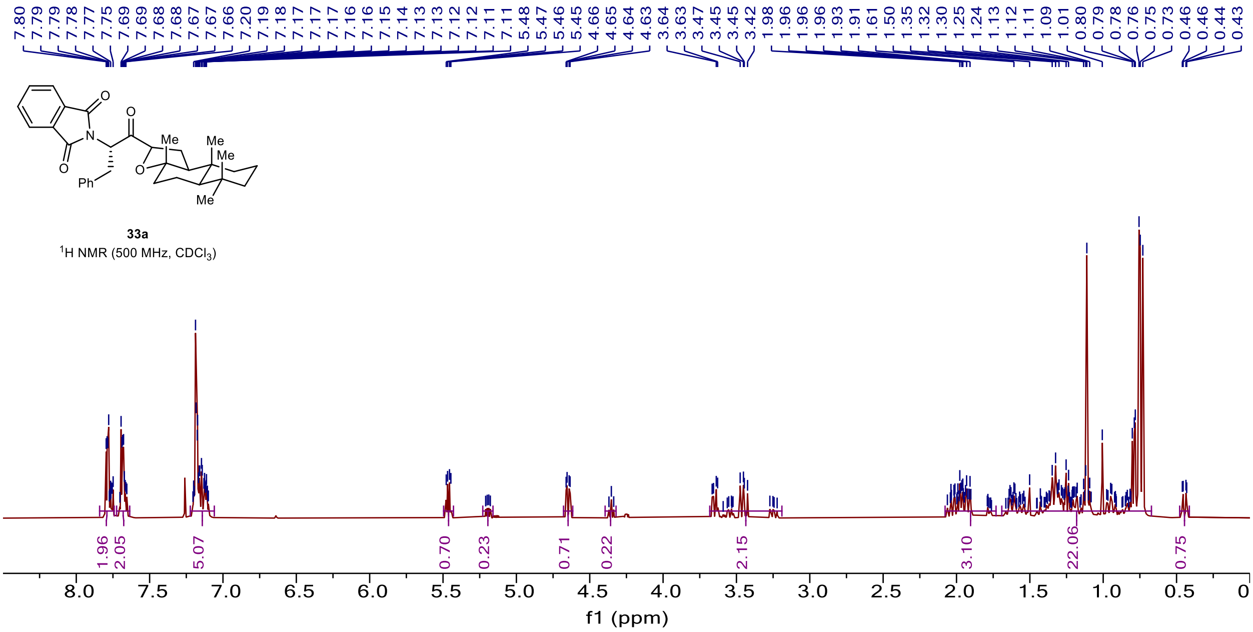

Supplementary Fig. 178. ^{1}H NMR spectrum of compound 33a.

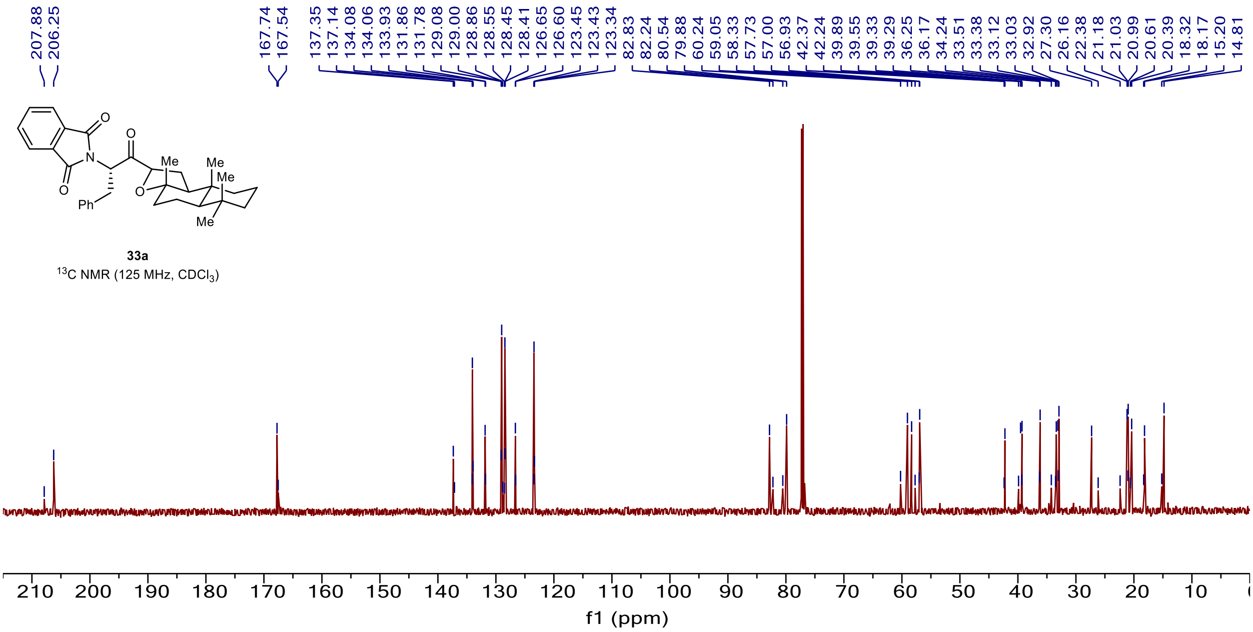

Supplementary Fig. 179. ^{13}C NMR spectrum of compound 33a.

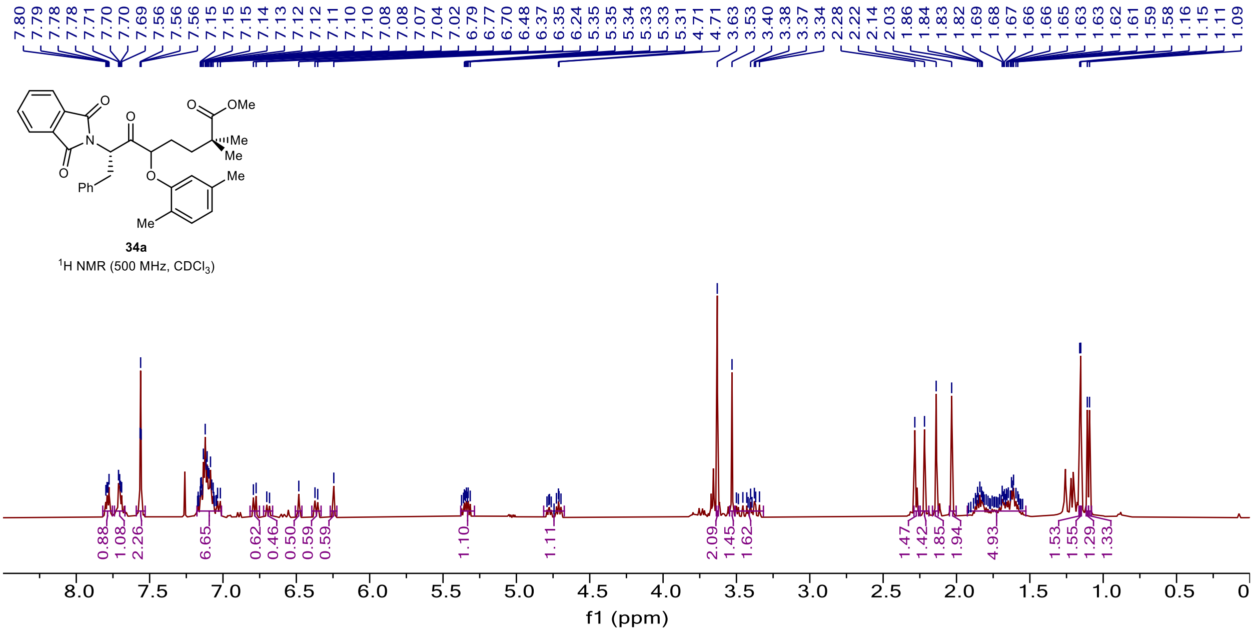

Supplementary Fig. 180. ^{1}H NMR spectrum of compound 34a.

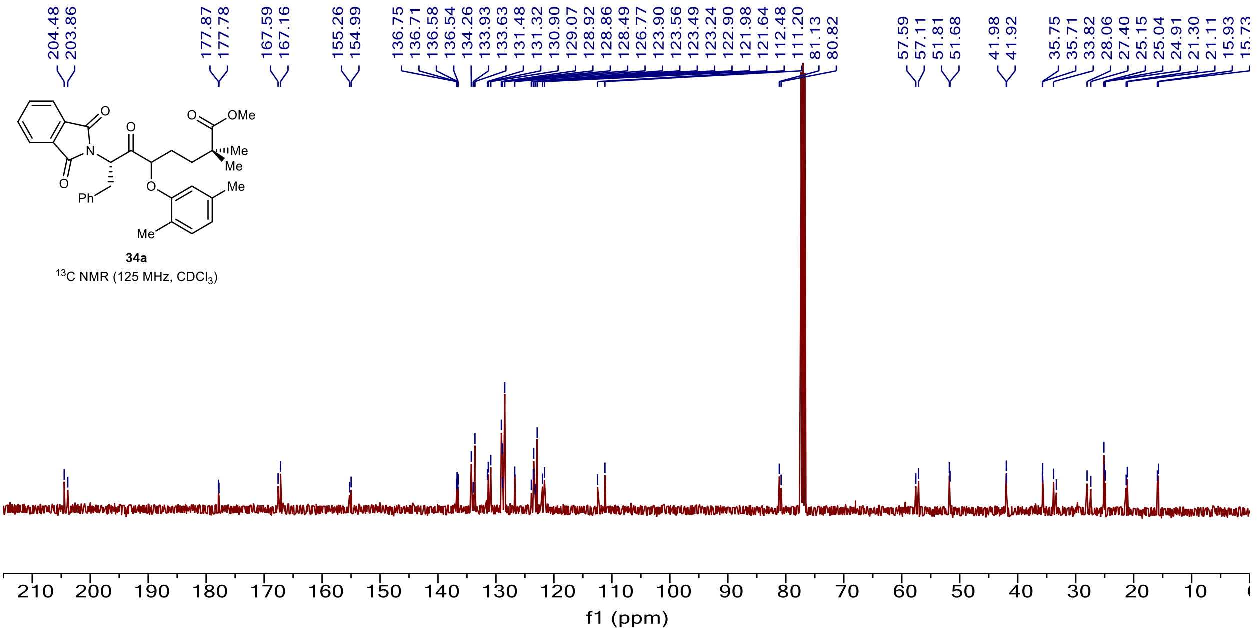

Supplementary Fig. 181. ^{13}C NMR spectrum of compound 34a.

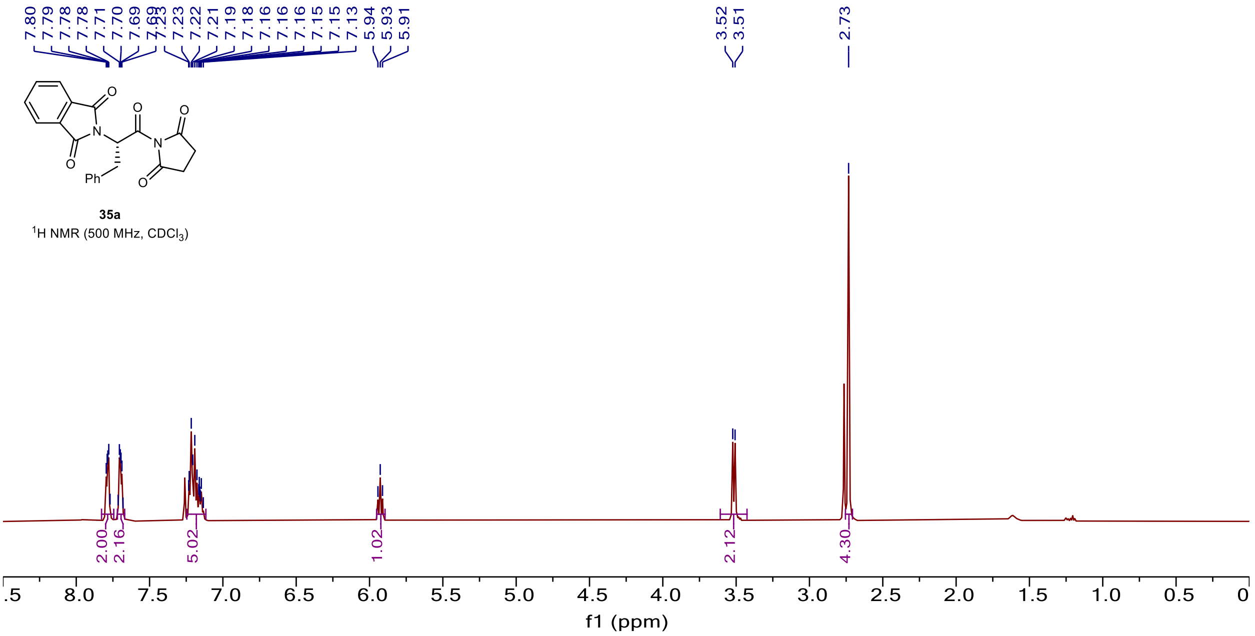

Supplementary Fig. 182. ^{1}H NMR spectrum of compound 35a.

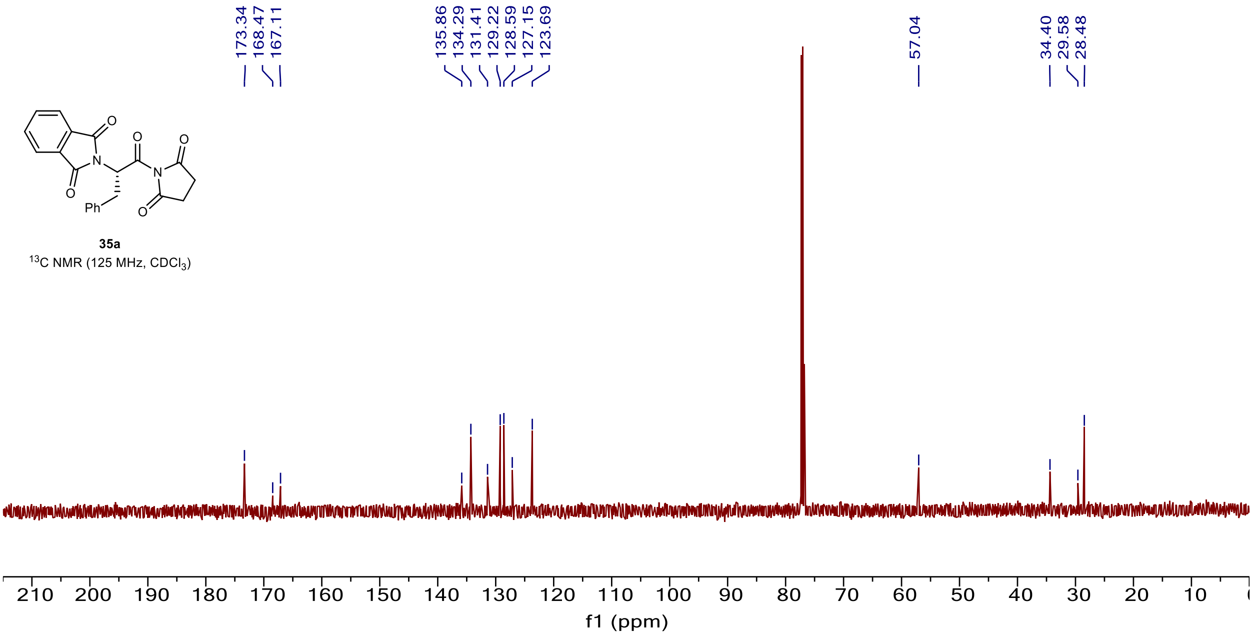

Supplementary Fig. 183. ^{13}C NMR spectrum of compound 35a.

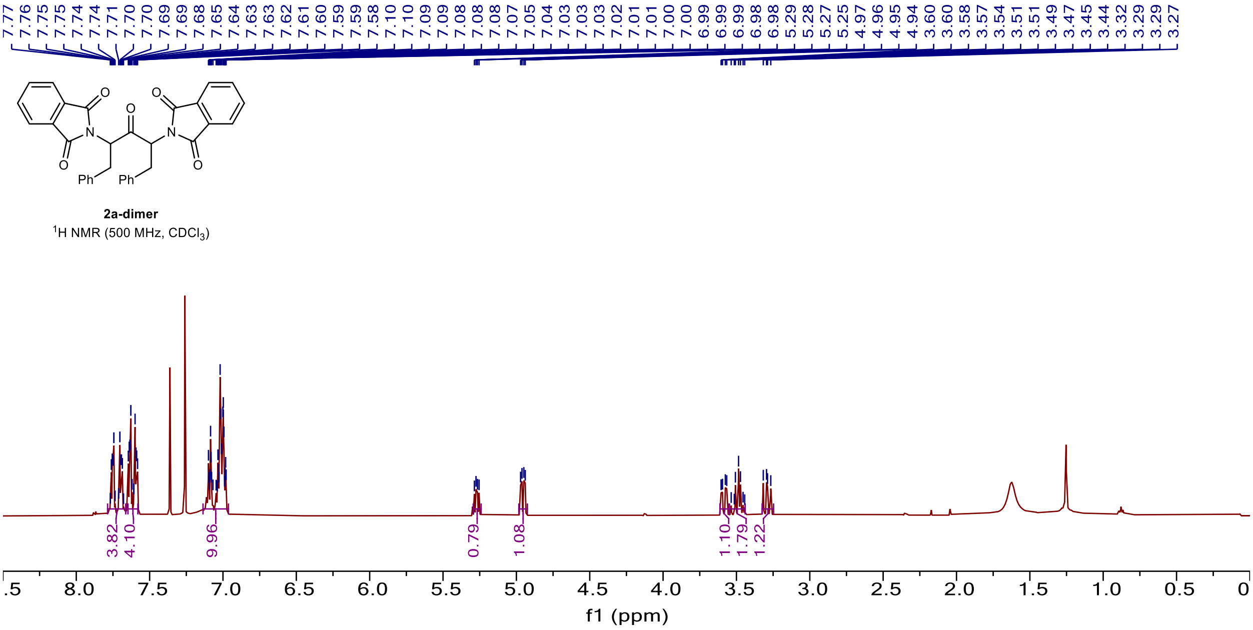

Supplementary Fig. 184. ^{1}**H NMR spectrum of compound 2a-dimer.**

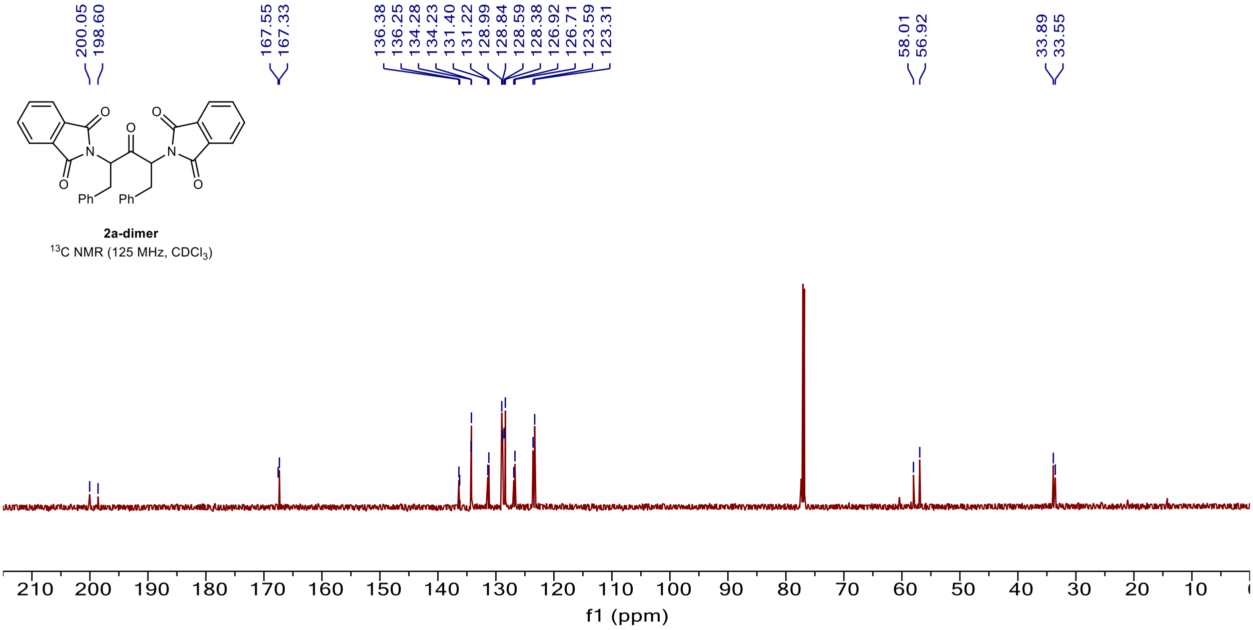

Supplementary Fig. 185. ^{13}**C NMR spectrum of compound 2a-dimer.**

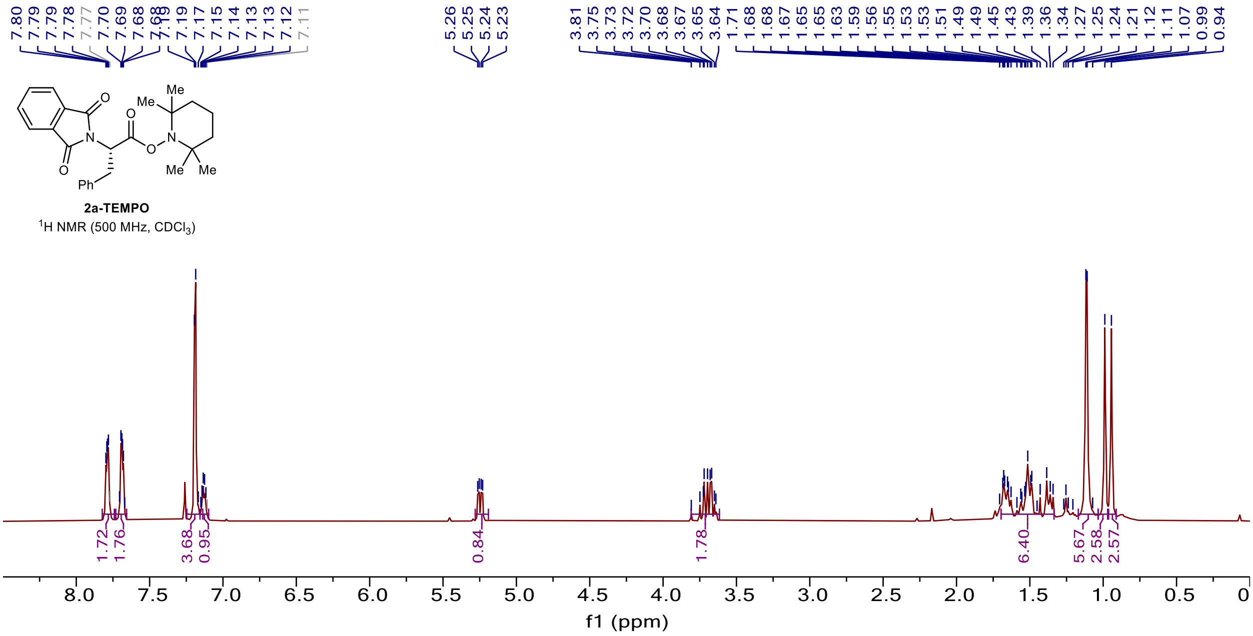

Supplementary Fig. 186. ^{1}H NMR spectrum of compound 2a-TEMPO.

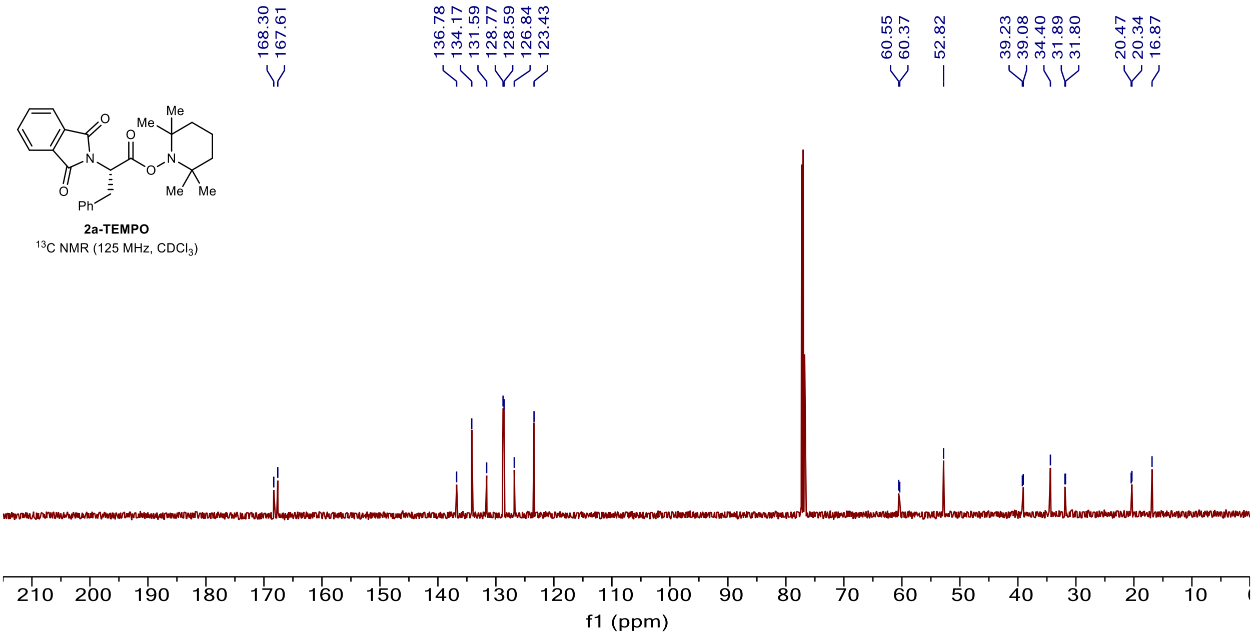

Supplementary Fig. 187. ^{13}C NMR spectrum of compound 2a-TEMPO.

4. Supplementary References

1. Lowry, M. S. *et al.* Single-Layer Electroluminescent Devices and Photoinduced Hydrogen Production from an Ionic Iridium(III) Complex. *Chem. Mater.* **17**, 5712-5719, (2005).
2. Zhang, Q. *et al.* Stereoselective Synthesis of Chiral α-Amino-β-Lactams through Palladium(II)-Catalyzed Sequential Monoarylation/Amidation of C(sp^3)–H Bonds. *Angew. Chem. Int. Ed.* **52**, 13588-13592, (2013).
3. Lee, G. S., Won, J., Choi, S., Baik, M.-H. & Hong, S. H. Synergistic Activation of Amides and Hydrocarbons for Direct C(sp^3)–H Acylation Enabled by Metallaphotoredox Catalysis. *Angew. Chem. Int. Ed.* **59**, 16933-16942, (2020).
4. Niu, L. *et al.* Visible-Light-Induced External Oxidant-Free Oxidative Phosphonylation of C(sp^2)–H Bonds. *ACS Catal.* **7**, 7412-7416, (2017).
5. Zhang, Q. *et al.* Stereoselective Synthesis of Chiral α-Amino-β-Lactams through Palladium(II)-Catalyzed Sequential Monoarylation/Amidation of C(sp^3)–H Bonds. *Angew. Chem. Int. Ed.* **52**, 13588-13592, (2013).
6. Zhu, Y. *et al.* Pd-catalysed ligand-enabled carboxylate-directed highly regioselective arylation of aliphatic acids. *Nat. Commun.* **8**, 14904, (2017).
7. Roy, I. L., Mouysset, D., Mignani, S., Vuilhorgne, M. & Stella, L. Solid phase β-lactams synthesis using the Staudinger reaction, monitored by ^{19}F NMR spectroscopy. *Tetrahedron* **59**, 3719-3727, (2003).
8. Uraguchi, D., Kinoshita, N. & Ooi, T. Catalytic Asymmetric Protonation of α-Amino Acid-Derived Ketene Disilyl Acetals Using *P*-Spiro Diaminodioxaphosphonium Barfates as Chiral Proton. *J. Am. Chem. Soc.* **132**, 12240-12242, (2010).
9. Mizrahi, D. M., Waner, T. & Segall, Y. α-Amino Acid Derived Bisphosphonates. Synthesis and Anti-Resorftive Activity. *Phosphorus Sulfur Silicon Relat. Elem.* **173**, 1-25, (2001).
10. Castaño, A. M. & Echavarren, A. M. Reactivity of a Nickelacycle Derived from Aspartic Acid: Alkylations, Insertions, and Oxidations. *Organometallics* **13**, 2262-2268, (1994).
11. Vicens, L., Bietti, M. & Costas, M. General Access to Modified α-Amino Acids by Bioinspired Stereoselective γ-C–H Bond Lactonization. *Angew. Chem. Int. Ed.* **60**, 4740-4746, (2021).
12. Bume, D. D., Pitts, C. R., Jokhai, R. T. & Lectka, T. Direct, visible light-sensitized benzylic C–H fluorination of peptides using dibenzosuberenone: selectivity for phenylalanine-like residues. *Tetrahedron* **72**, 6031-6036, (2016).
13. Estevan, F., Herbst, K. & Lahuerta, P. Chiral Dirhodium(II) Catalysts with Orthometalated Aryl Phosphine Ligands: Synthesis and Application for Enantioselective C–H Insertion of α-Diazo Ketones. *Organometallics* **20**, 950-957, (2001).
14. Dai, J., Ren, W., Wang, H. & Shi, Y. A facile approach to β-amino acid derivatives *via* palladium-catalyzed hydrocarboxylation of enimides with formic acid. *Org. Biomol. Chem.* **13**, 8429-8432, (2015).
15. Griesbeck, A. G. *et al.* Synthesis of Medium- and Large-Ring Compounds Initiated by Photochemical Decarboxylation of ω-Phthalimidoalkanoates. *Helv. Chim. Acta* **80**, 912-933, (1997).
16. Abdel-Hafez, A. A.-M. Synthesis and Anticonvulsant Evaluation of *N*-Substituted-Isoindolinedione Derivatives. *Arch. Pharm. Res.* **27**, 495-501, (2004).
17. Holmberg-Douglas, N., Choi, Y., Aquila, B., Huynh, H. & Nicewicz, D. A. *β*-Functionalization of Saturated Aza-Heterocycles Enabled by Organic Photoredox Catalysis. *ACS Catal.* **11**, 3153-3158, (2021).
18. Ackerman, L. K. G., Martinez Alvarado, J. I. & Doyle, A. G. Direct C–C Bond Formation from Alkanes Using Ni-Photoredox Catalysis. *J. Am. Chem. Soc.* **140**, 14059-14063, (2018).
19. Amani, J., Alam, R., Badir, S. & Molander, G. A. Synergistic Visible-Light Photoredox/Nickel-Catalyzed Synthesis of Aliphatic Ketones via N–C Cleavage of Imides. *Org. Lett.* **19**, 2426-2429, (2017).
20. Chen, J.-Q. *et al.* Efficient access to aliphatic esters by photocatalyzed alkoxycarbonylation of alkenes with alkyloxalyl chlorides. *Nat. Commun.* **12**, 5328, (2021).

21. Gaussian 09, Revision D.01, M. J. Frisch, G. W. Trucks, H. B. Schlegel, G. E. Scuseria, M. A. Robb, J. R. Cheeseman, G. Scalmani, V. Barone, B. Mennucci, G. A. Petersson, H. Nakatsuji, M. Caricato, X. Li, H. P. Hratchian, A. F. Izmaylov, J. Bloino, G. Zheng, J. L. Sonnenberg, M. Hada, M. Ehara, K. Toyota, R. Fukuda, J. Hasegawa, M. Ishida, T. Nakajima, Y. Honda, O. Kitao, H. Nakai, T. Vreven, J. A. Montgomery, Jr., J. E. Peralta, F. Ogliaro, M. Bearpark, J. J. Heyd, E. Brothers, K. N. Kudin, V. N. Staroverov, T. Keith, R. Kobayashi, J. Normand, K. Raghavachari, A. Rendell, J. C. Burant, S. S. Iyengar, J. Tomasi, M. Cossi, N. Rega, J. M. Millam, M. Klene, J. E. Knox, J. B. Cross, V. Bakken, C. Adamo, J. Jaramillo, R. Gomperts, R. E. Stratmann, O. Yazyev, A. J. Austin, R. Cammi, C. Pomelli, J. W. Ochterski, R. L. Martin, K. Morokuma, V. G. Zakrzewski, G. A. Voth, P. Salvador, J. J. Dannenberg, S. Dapprich, A. D. Daniels, O. Farkas, J. B. Foresman, J. V. Ortiz, J. Cioslowski, and D. J. Fox, Gaussian, Inc., Wallingford CT, 2013.

22. Lee, C., Yang, W. & Parr, R. G. Development of the Colle-Salvetti correlation-energy formula into a functional of the electron density. *Phys. Rev. B.* **37**, 785-789, (1988).

23. Grimme, S., Antony, J., Ehrlich, S. & Krieg, H. A consistent and accurate *ab initio* parametrization of density functional dispersion correction (DFT-D) for the 94 elements H-Pu. *J. Chem. Phys.* **132**, 154104, (2010).

24. Hay, P. J. & Wadt, W. R. *Ab initio* effective core potentials for molecular calculations. Potentials for K to Au including the outermost core orbitals. *J. Chem. Phys.* **82**, 299-310, (1985).

25. Wadt, W. R. & Hay, P. J. *Ab initio* effective core potentials for molecular calculations. Potentials for main group elements Na to Bi. *J. Chem. Phys.* **82**, 284-298, (1985).

26. Trasatti, S. The Absolute Electrode Potential: An Explanatory Note. *Pure Appl. Chem.* **58**, 955-966, (1986).

27. Isse, A. A. & Gennaro, A. Absolute Potential of the Standard Hydrogen Electrode and the Problem of Interconversion of Potentials in Different Solvents. *J. Phys. Chem. B* **114**, 7894-7899, (2010).

28. Marcus, R. A. On the Theory of Electron-Transfer Reactions. VI. Unified Treatment for Homogeneous and Electrode Reactions. *J. Chem. Phys.* **43**, 679-701, (1965).

29. Anderson, G. M., Cameron, I., Murphy, J. A. & Tuttle, T. Predicting the reducing power of organic super electron donors. *RSC Adv.* **6**, 11335-11343, (2016).

30. Ren, H. *et al.* How Does Iridium(III) Photocatalyst Regulate Nickel(II) Catalyst in Metallaphotoredox-Catalyzed C–S Cross-Coupling? Theoretical and Experimental Insights. *ACS Catal.* **9**, 3858-3865, (2019).

31. Nelsen, S. F., Blackstock, S. C. & Kim, Y. Estimation of Inner Shell Marcus Terms for Amino Nitrogen Compounds by Molecular Orbital Calculations. *J. Am. Chem. Soc.* **109**, 677-682, (1987).
