# Supplementary material for: Stereoretentive cross-coupling of chiral amino acid chlorides and hydrocarbons through mechanistically controlled Ni/Ir photoredox catalysis
Source: Nat Commun. 2022 Sep 3;13:5200. doi: 10.1038/s41467-022-32851-7 (PMC9440902; doi:10.1038/s41467-022-32851-7)
Supplement: Supplementary file 2 — Description of Additional Supplementary Files [file 41467_2022_32851_MOESM2_ESM.docx]

**Description of Additional Supplementary Files**

**File Name:** Supplementary Data 1

**Description:** Cartesian coordinates of DFT optimized structures.
