# Supplementary material for: Stereoretentive cross-coupling of chiral amino acid chlorides and hydrocarbons through mechanistically controlled Ni/Ir photoredox catalysis
Source: Nat Commun. 2022 Sep 3;13:5200. doi: 10.1038/s41467-022-32851-7 (PMC9440902; doi:10.1038/s41467-022-32851-7)
Supplement: Supplementary file 3 — Supplementary Data 1 [file 41467_2022_32851_MOESM3_ESM.docx]

## Cartesian Coordinates of DFT Optimized Structures

===============================

**cyclohexane**

===============================

C -0.002922000 -0.000282000 -1.483098000

C 0.228028000 1.293964000 -0.687976000

C -0.453055000 1.233412000 0.687963000

C 0.002922000 0.000282000 1.483098000

C -0.228028000 -1.293964000 0.687976000

C 0.453055000 -1.233412000 -0.687963000

H 0.518451000 0.046225000 -2.446833000

H -1.074538000 -0.095680000 -1.710374000

H -0.134704000 2.159753000 -1.255084000

H 1.308647000 1.441239000 -0.547601000

H -0.249111000 2.149640000 1.255151000

H -1.542681000 1.187292000 0.547528000

H -0.518451000 -0.046225000 2.446833000

H 1.074538000 0.095680000 1.710374000

H 0.134704000 -2.159753000 1.255084000

H -1.308647000 -1.441239000 0.547601000

H 0.249111000 -2.149640000 -1.255151000

H 1.542681000 -1.187292000 -0.547528000

===============================

**2,6-lutidine**

===============================

C 0.000009000 1.841851000 0.000009000

C -1.199773000 1.134881000 -0.000366000

C -1.156587000 -0.265188000 -0.000404000

N 0.000000000 -0.946637000 -0.000114000

C 1.156574000 -0.265203000 0.000254000

C 1.199777000 1.134882000 0.000376000

H 0.000002000 2.928344000 0.000060000

H -2.153905000 1.652403000 -0.000605000

H 2.153919000 1.652384000 0.000690000

C 2.415835000 -1.096167000 -0.000117000

H 2.442872000 -1.742732000 -0.883297000

H 3.315414000 -0.475025000 0.005976000

H 2.437047000 -1.752256000 0.876084000

C -2.415837000 -1.096164000 0.000230000

H -2.436953000 -1.752704000 -0.875618000

H -2.442953000 -1.742272000 0.883758000

H -3.315424000 -0.475037000 -0.006151000

===============================

**2,6-lutidine•HCl**

===============================

C 2.689994000 -0.000087000 -0.102533000

C 1.995052000 1.207471000 -0.055743000

C 0.604149000 1.189523000 0.038661000

N -0.029378000 0.000029000 0.084299000

C 0.604045000 -1.189521000 0.038656000

C 1.994952000 -1.207583000 -0.055750000

H 3.772971000 -0.000131000 -0.180576000

H 2.515411000 2.157606000 -0.095975000

H 2.515233000 -2.157761000 -0.095990000

C -0.251806000 -2.420162000 0.108274000

H -1.163229000 -2.287764000 -0.481080000

H 0.300886000 -3.296413000 -0.235967000

H -0.573051000 -2.594184000 1.141270000

C -0.251629000 2.420215000 0.108265000

H -1.162997000 2.287897000 -0.481195000

H -0.572984000 2.594191000 1.141233000

H 0.301143000 3.296454000 -0.235875000

H -1.193232000 0.000071000 0.065323000

Cl -2.873120000 0.000041000 -0.094720000

===============================

**2aa-lut**

===============================

C -0.037004000 0.022859000 0.969234000

O -0.014311000 -0.203723000 2.140258000

C 3.763081000 0.128802000 -1.001262000

C 3.043555000 1.304426000 -0.805220000

C 1.809733000 1.268633000 -0.166705000

N 1.326719000 0.058848000 0.249523000

C 2.029747000 -1.110873000 0.133453000

C 3.256544000 -1.075724000 -0.519903000

H 4.722965000 0.154028000 -1.506641000

H 3.433271000 2.260619000 -1.134183000

H 3.812840000 -1.998572000 -0.632186000

C 1.020471000 2.521489000 0.095977000

H 0.315654000 2.725298000 -0.716971000

H 0.459847000 2.473788000 1.032400000

H 1.703382000 3.370510000 0.157386000

C 1.487609000 -2.371692000 0.741482000

H 1.469996000 -2.285901000 1.831749000

H 0.468664000 -2.588056000 0.413081000

H 2.121684000 -3.213851000 0.464570000

C -1.227572000 0.220638000 0.074749000

C -2.410855000 0.808217000 0.869008000

C -3.645237000 0.965942000 -0.029289000

C -4.033517000 -0.364045000 -0.689091000

C -2.856591000 -0.957273000 -1.474488000

C -1.613973000 -1.136366000 -0.587795000

H -0.949849000 0.897795000 -0.740634000

H -2.122603000 1.771163000 1.305460000

H -2.633959000 0.138005000 1.706481000

H -4.476178000 1.356960000 0.565831000

H -3.437643000 1.714269000 -0.806692000

H -4.891432000 -0.221350000 -1.353304000

H -4.349030000 -1.076145000 0.085079000

H -3.126872000 -1.927390000 -1.903997000

H -2.604128000 -0.298518000 -2.316355000

H -0.778834000 -1.519901000 -1.185181000

H -1.826258000 -1.865100000 0.204490000

===============================

**2aa-lut-red**

===============================

C 0.020182000 -0.354265000 0.783869000

O -0.091831000 -1.135421000 1.721660000

C 3.775995000 0.618051000 -0.938309000

C 2.837411000 1.616920000 -0.639182000

C 1.635633000 1.335609000 -0.031829000

N 1.298752000 -0.018197000 0.247820000

C 2.279813000 -1.029513000 0.061775000

C 3.470018000 -0.697672000 -0.535061000

H 4.719051000 0.854945000 -1.415263000

H 3.069923000 2.660095000 -0.831078000

H 4.181880000 -1.498566000 -0.706815000

C 0.756705000 2.428222000 0.510137000

H -0.152868000 2.611961000 -0.071964000

H 0.438041000 2.193824000 1.533410000

H 1.317787000 3.364987000 0.538251000

C 1.936356000 -2.451510000 0.395731000

H 1.754808000 -2.593528000 1.462896000

H 1.020418000 -2.770734000 -0.116605000

H 2.752305000 -3.101056000 0.071392000

C -1.208623000 0.123876000 0.019581000

C -2.283882000 0.701565000 0.957451000

C -3.575296000 1.040021000 0.200010000

C -4.118247000 -0.177151000 -0.561949000

C -3.053285000 -0.759052000 -1.502201000

C -1.767644000 -1.108005000 -0.740711000

H -0.933055000 0.866091000 -0.735066000

H -1.899537000 1.592334000 1.466834000

H -2.483784000 -0.044245000 1.735651000

H -4.328806000 1.420514000 0.899479000

H -3.374310000 1.851590000 -0.514462000

H -5.017632000 0.095513000 -1.126447000

H -4.419533000 -0.948542000 0.160456000

H -3.436494000 -1.651641000 -2.010688000

H -2.822537000 -0.025275000 -2.287620000

H -1.006130000 -1.492710000 -1.430148000

H -1.966963000 -1.897395000 -0.006104000

===============================

**^3^IV-2aa**

===============================

Ni -0.577294000 -1.258306000 -1.439973000

Cl -1.543568000 -3.339055000 -1.093588000

Cl -2.304418000 -0.410216000 -2.738671000

C 0.829660000 2.866698000 -1.984185000

C 2.168674000 -0.817316000 -0.427292000

C 3.432890000 -1.056837000 0.116001000

C 3.794448000 -2.338854000 0.547740000

C 2.823774000 -3.341646000 0.398393000

C 1.585338000 -3.042593000 -0.153778000

N 1.258201000 -1.805387000 -0.557324000

H 4.142668000 -0.244319000 0.178572000

H 3.024026000 -4.363342000 0.699344000

H 0.810260000 -3.788896000 -0.291428000

C 1.738982000 0.510759000 -0.931527000

C 2.444589000 1.692143000 -0.685054000

C 1.995241000 2.912532000 -1.202255000

C 0.164833000 1.663255000 -2.177653000

N 0.597793000 0.509757000 -1.649237000

H 3.335719000 1.656558000 -0.074748000

H 0.424314000 3.763897000 -2.437521000

H -0.752452000 1.586266000 -2.751717000

C 2.699570000 4.245821000 -0.929355000

C 5.168879000 -2.664905000 1.141453000

C 4.975176000 -3.226226000 2.570603000

H 4.485263000 -2.492705000 3.219229000

H 4.372858000 -4.139095000 2.574722000

H 5.948452000 -3.469673000 3.007628000

C 6.080662000 -1.426986000 1.219395000

H 7.043472000 -1.710150000 1.653345000

H 6.281213000 -1.004267000 0.229253000

H 5.653318000 -0.643931000 1.855172000

C 5.856172000 -3.729810000 0.253072000

H 6.839744000 -3.976175000 0.664811000

H 5.276855000 -4.655854000 0.200543000

H 5.996883000 -3.360009000 -0.767382000

C 3.989755000 4.069460000 -0.108183000

H 3.792010000 3.638425000 0.879169000

H 4.718023000 3.437020000 -0.626470000

H 4.457595000 5.044838000 0.050949000

C 3.060154000 4.918701000 -2.274869000

H 3.733825000 4.286832000 -2.861763000

H 2.175597000 5.126352000 -2.883178000

H 3.563923000 5.872081000 -2.088586000

C 1.729712000 5.154581000 -0.135103000

H 0.803253000 5.341871000 -0.686246000

H 1.469286000 4.700224000 0.827104000

H 2.201474000 6.121810000 0.063652000

C -2.238332000 0.249632000 0.836841000

O -1.170974000 -0.151088000 0.435954000

N -3.431905000 -0.600062000 0.574688000

C -2.411196000 1.506006000 1.639433000

C -5.458085000 -2.299285000 -0.133863000

C -5.404351000 -1.015077000 -0.665854000

C -4.479231000 -2.715281000 0.762456000

H -6.254388000 -2.976634000 -0.424050000

C -4.380099000 -0.149818000 -0.312777000

H -6.143670000 -0.673449000 -1.379872000

C -4.323768000 1.251947000 -0.847839000

C -3.438120000 -1.867962000 1.108607000

C -2.341698000 -2.301514000 2.035770000

H -4.490705000 -3.714124000 1.180045000

H -4.847003000 1.943796000 -0.177316000

H -3.303312000 1.593836000 -1.015058000

H -4.817560000 1.273013000 -1.819308000

H -1.466358000 -2.570846000 1.438410000

H -2.064564000 -1.531149000 2.757723000

H -2.666149000 -3.186784000 2.583457000

C -1.458185000 2.607001000 1.122714000

C -2.131557000 1.195097000 3.141729000

H -3.450600000 1.839433000 1.568480000

C -1.573287000 3.876142000 1.975542000

H -1.673229000 2.824760000 0.071260000

H -0.433219000 2.220287000 1.154710000

C -1.302056000 3.579279000 3.456720000

H -0.873649000 4.629025000 1.598111000

H -2.579990000 4.301650000 1.864389000

C -2.236338000 2.479449000 3.978959000

H -1.421787000 4.487933000 4.055217000

H -0.258677000 3.256377000 3.577167000

H -2.007790000 2.240829000 5.022993000

H -3.274188000 2.837929000 3.957866000

H -2.841592000 0.447373000 3.511646000

H -1.123199000 0.771347000 3.227763000

===============================

**^3^IV-2aa-red**

===============================

Ni -0.643684000 -0.331390000 -1.915161000

Cl -1.274287000 -2.483241000 -2.575695000

Cl -2.161047000 0.862784000 -3.204678000

C 0.250038000 3.739494000 -0.447256000

C 2.103538000 -0.037216000 -0.768087000

C 3.373599000 -0.368768000 -0.284852000

C 3.914022000 -1.638121000 -0.515361000

C 3.123582000 -2.529826000 -1.255656000

C 1.865705000 -2.142625000 -1.701261000

N 1.362603000 -0.925537000 -1.457435000

H 3.940254000 0.370101000 0.263425000

H 3.470937000 -3.530883000 -1.484707000

H 1.201054000 -2.805701000 -2.246789000

C 1.488519000 1.305162000 -0.593122000

C 2.071442000 2.328778000 0.161482000

C 1.451874000 3.579171000 0.258757000

C -0.277778000 2.679018000 -1.174308000

N 0.320252000 1.484677000 -1.234223000

H 3.002623000 2.143120000 0.677510000

H -0.286506000 4.681533000 -0.435159000

H -1.202231000 2.752467000 -1.738347000

C 2.028018000 4.735471000 1.085886000

C 5.294101000 -2.065230000 -0.000237000

C 5.121257000 -3.285437000 0.935183000

H 4.488608000 -3.034716000 1.792810000

H 4.665606000 -4.134241000 0.417609000

H 6.096882000 -3.609111000 1.312819000

C 5.999452000 -0.944616000 0.785273000

H 6.974500000 -1.298752000 1.132901000

H 6.173729000 -0.059691000 0.164096000

H 5.425480000 -0.642706000 1.667794000

C 6.183728000 -2.455740000 -1.203812000

H 7.172374000 -2.769101000 -0.852262000

H 5.755742000 -3.283524000 -1.776018000

H 6.315740000 -1.608720000 -1.884582000

C 3.351003000 4.359439000 1.777683000

H 3.223281000 3.524023000 2.474230000

H 4.127321000 4.092191000 1.052962000

H 3.719302000 5.213804000 2.353386000

C 2.287807000 5.942814000 0.154569000

H 3.007048000 5.685044000 -0.629361000

H 1.371015000 6.288538000 -0.330988000

H 2.695882000 6.779633000 0.731176000

C 1.001464000 5.130836000 2.173716000

H 0.051084000 5.452871000 1.738839000

H 0.797598000 4.289039000 2.843518000

H 1.391619000 5.958999000 2.774732000

C -1.987420000 -0.676057000 0.957645000

O -1.033830000 -0.529840000 0.166166000

N -1.770358000 -1.403956000 2.151797000

C -3.396106000 -0.238383000 0.632426000

C -1.545861000 -2.938446000 4.501534000

C -0.956483000 -3.355026000 3.294560000

C -2.155184000 -1.679080000 4.519675000

H -1.487890000 -3.549023000 5.394702000

C -1.033865000 -2.616197000 2.136917000

H -0.431241000 -4.302868000 3.243097000

C -0.448896000 -3.113345000 0.851611000

C -2.241281000 -0.893433000 3.390352000

C -2.652103000 0.550262000 3.477830000

H -2.526295000 -1.262024000 5.450802000

H -1.163134000 -3.110303000 0.022410000

H 0.379309000 -2.484044000 0.520780000

H -0.087215000 -4.133759000 0.996509000

H -1.978582000 1.176816000 2.880786000

H -3.668355000 0.755204000 3.126652000

H -2.592241000 0.880250000 4.517597000

C -3.951920000 -1.183880000 -0.471529000

C -3.472271000 1.229052000 0.169847000

H -4.032776000 -0.384091000 1.509925000

C -5.390927000 -0.791382000 -0.832580000

H -3.909235000 -2.222009000 -0.122106000

H -3.319008000 -1.120764000 -1.359461000

C -5.470104000 0.672519000 -1.288211000

H -5.754042000 -1.456246000 -1.624272000

H -6.046957000 -0.944369000 0.038122000

C -4.908001000 1.614656000 -0.213324000

H -6.506823000 0.942712000 -1.522881000

H -4.881436000 0.790711000 -2.205437000

H -4.925316000 2.651594000 -0.569305000

H -5.548458000 1.577450000 0.681543000

H -3.091810000 1.895740000 0.953311000

H -2.826141000 1.340766000 -0.703869000

===============================

**2z**

===============================

C -1.937462000 -1.414132000 0.000349000

C -0.572443000 -1.136154000 0.000415000

C -2.867366000 -0.372331000 -0.000082000

H 0.148924000 -1.943753000 0.000723000

H -3.930259000 -0.595027000 -0.000160000

C -0.133164000 0.195150000 0.000080000

C -2.433019000 0.956341000 -0.000426000

H -3.155168000 1.766726000 -0.000777000

C -1.072393000 1.242284000 -0.000310000

H -0.715900000 2.266202000 -0.000533000

H -2.275002000 -2.445656000 0.000633000

C 1.293516000 0.600432000 0.000237000

O 1.706069000 1.720908000 0.000686000

Cl 2.506638000 -0.779135000 -0.000408000

===============================

**2z-lut**

===============================

C 1.422273000 0.001286000 0.282957000

C 1.401391000 -0.004690000 -1.125327000

C 2.650783000 0.004515000 0.973590000

C 2.599445000 -0.007581000 -1.830869000

C 3.841491000 0.001691000 0.258737000

C 3.816331000 -0.004412000 -1.140607000

H 0.460251000 -0.006979000 -1.665081000

H 2.645871000 0.009348000 2.058114000

H 2.588895000 -0.012203000 -2.915485000

H 4.789187000 0.004289000 0.786381000

H 4.748898000 -0.006616000 -1.695955000

C 0.206111000 0.004504000 1.091183000

O 0.097454000 0.009675000 2.287132000

C -3.528537000 -0.004133000 -0.959548000

C -2.911740000 -1.208476000 -0.627144000

C -1.681771000 -1.205338000 0.020026000

N -1.105428000 0.000989000 0.300434000

C -1.682564000 1.205066000 0.011783000

C -2.912484000 1.202904000 -0.635502000

H -4.489571000 -0.006188000 -1.463465000

H -3.377407000 -2.158524000 -0.860201000

H -3.378814000 2.150968000 -0.875178000

C -0.968376000 -2.458059000 0.434221000

H 0.029923000 -2.511896000 -0.011231000

H -0.851700000 -2.492308000 1.522012000

H -1.535440000 -3.333438000 0.117899000

C -0.970322000 2.461074000 0.417881000

H -0.855513000 2.503055000 1.505630000

H 0.028771000 2.512154000 -0.026099000

H -1.537168000 3.333913000 0.094286000

===============================

**2z-lut-red**

===============================

C 1.414440000 0.303099000 0.128771000

C 1.514012000 -0.585304000 -0.957034000

C 2.593769000 0.776958000 0.731296000

C 2.761959000 -1.006800000 -1.410309000

C 3.837289000 0.351166000 0.277083000

C 3.927365000 -0.544907000 -0.793221000

H 0.612636000 -0.933661000 -1.451462000

H 2.504613000 1.481024000 1.551372000

H 2.825743000 -1.689787000 -2.252369000

H 4.740688000 0.717572000 0.755921000

H 4.899112000 -0.875963000 -1.147356000

C 0.126105000 0.848105000 0.613780000

O 0.049324000 1.896818000 1.255846000

C -3.521623000 -1.061593000 -0.440782000

C -2.494167000 -1.777157000 0.191237000

C -1.299067000 -1.187566000 0.539591000

N -1.077699000 0.167954000 0.206584000

C -2.114288000 0.921984000 -0.385261000

C -3.300103000 0.301856000 -0.700818000

H -4.460328000 -1.536928000 -0.698380000

H -2.639038000 -2.817422000 0.464632000

H -4.064964000 0.898248000 -1.187271000

C -0.271099000 -1.919822000 1.351091000

H 0.600776000 -2.231554000 0.766257000

H 0.108013000 -1.289712000 2.164199000

H -0.724394000 -2.812394000 1.787651000

C -1.838011000 2.347328000 -0.756577000

H -1.689805000 2.977834000 0.123393000

H -0.917258000 2.423749000 -1.348816000

H -2.665971000 2.734679000 -1.353711000

===============================

**3z**

===============================

C 3.847331000 0.155100000 -0.000001000

C 2.699308000 0.939798000 0.000011000

C 3.742269000 -1.238879000 -0.000015000

H 2.760087000 2.022442000 0.000019000

H 4.637586000 -1.853891000 -0.000022000

C 1.422214000 0.351372000 0.000010000

C 2.482358000 -1.837639000 -0.000017000

H 2.392878000 -2.919902000 -0.000032000

C 1.331264000 -1.049695000 -0.000006000

H 0.366788000 -1.539304000 -0.000020000

H 4.825424000 0.627157000 -0.000001000

C 0.240786000 1.286797000 0.000019000

O 0.439138000 2.495274000 0.000006000

C -1.207892000 0.789435000 0.000029000

C -1.607142000 0.019912000 1.280908000

C -3.107848000 -0.309241000 1.267701000

C -3.501728000 -1.081904000 -0.000024000

C -3.107844000 -0.309182000 -1.267711000

C -1.607141000 0.019969000 -1.280898000

H -1.783982000 1.723187000 0.000044000

H -1.355862000 0.622305000 2.161530000

H -1.037308000 -0.911294000 1.369288000

H -3.371775000 -0.882881000 2.163827000

H -3.682241000 0.626454000 1.312132000

H -4.578259000 -1.288538000 -0.000034000

H -2.994884000 -2.057894000 -0.000044000

H -3.371775000 -0.882781000 -2.163863000

H -3.682236000 0.626517000 -1.312102000

H -1.355858000 0.622411000 -2.161484000

H -1.037301000 -0.911229000 -1.369314000

===============================

**Ir(II)**

===============================

N 2.137226000 0.461927000 -1.049515000

N -0.676194000 -1.504590000 1.217877000

C 1.437430000 -2.065152000 -0.903415000

C 1.018387000 -3.397256000 -0.792243000

C 1.629376000 -4.379981000 -1.559181000

C 2.663218000 -4.108288000 -2.454409000

C 3.076397000 -2.791884000 -2.556005000

C 2.498668000 -1.753068000 -1.808169000

C 2.882065000 -0.349217000 -1.870139000

C 3.904382000 0.217930000 -2.652562000

C 4.148250000 1.580324000 -2.599386000

C 3.364786000 2.382007000 -1.762188000

C 2.369474000 1.784135000 -1.005677000

C 1.842450000 -0.874571000 1.659003000

C 3.188280000 -0.530123000 1.839407000

C 3.830719000 -0.848943000 3.028432000

C 3.195473000 -1.512577000 4.076685000

C 1.868422000 -1.857219000 3.889853000

C 1.162508000 -1.562392000 2.712281000

C -0.231376000 -1.901650000 2.455134000

C -1.118242000 -2.569744000 3.319953000

C -2.424196000 -2.818702000 2.927426000

C -2.846312000 -2.400178000 1.662037000

C -1.939339000 -1.751310000 0.838310000

H 0.221002000 -3.683939000 -0.116067000

H 3.126697000 -4.888359000 -3.043885000

H 4.496237000 -0.421882000 -3.289115000

H 4.941686000 2.023755000 -3.191409000

H 1.739656000 2.357325000 -0.338563000

H 3.747954000 -0.014219000 1.067329000

H 3.712695000 -1.753439000 4.996066000

H -0.770437000 -2.879468000 4.293481000

H -3.115680000 -3.322867000 3.593562000

H -2.225788000 -1.407770000 -0.145291000

C -1.697961000 1.036659000 -0.968136000

C -2.951187000 1.183566000 -1.628417000

C -3.310881000 0.389558000 -2.695465000

C -2.342498000 -0.558803000 -3.156115000

C -1.155453000 -0.698878000 -2.472111000

N -0.828200000 0.035605000 -1.388419000

H -3.650792000 1.904240000 -1.227433000

H -2.537635000 -1.196896000 -4.009199000

H -0.425736000 -1.449385000 -2.759314000

C -1.300059000 1.793916000 0.175465000

C -1.971579000 2.961099000 0.638578000

C 0.234455000 2.021401000 1.950860000

C -1.548047000 3.653030000 1.753071000

H -2.821978000 3.312874000 0.070532000

H 1.113698000 1.614490000 2.441132000

C -0.396534000 3.147528000 2.433112000

H -0.004250000 3.632176000 3.318726000

N -0.175985000 1.348631000 0.857065000

Ir 0.681118000 -0.466807000 0.063130000

C 3.553642000 3.870798000 -1.721218000

F 4.081573000 -2.531790000 -3.429001000

F 1.214260000 -5.656259000 -1.444780000

F 1.256563000 -2.504794000 4.912790000

F 5.123240000 -0.505398000 3.190009000

C -4.231686000 -2.668392000 1.149242000

F 4.861646000 4.200185000 -1.810983000

F 2.923869000 4.478605000 -2.752223000

F 3.066732000 4.407492000 -0.582186000

F -4.279497000 -3.789943000 0.395834000

F -5.112229000 -2.827695000 2.160121000

F -4.674563000 -1.653119000 0.368789000

C -4.697749000 0.429998000 -3.343358000

C -5.605592000 1.506700000 -2.722551000

H -5.782918000 1.320380000 -1.658263000

H -5.177125000 2.508709000 -2.831615000

H -6.578172000 1.504705000 -3.224790000

C -5.369641000 -0.950530000 -3.139376000

H -6.379756000 -0.949169000 -3.564236000

H -4.803165000 -1.749905000 -3.626145000

H -5.440360000 -1.193701000 -2.074492000

C -4.559433000 0.721453000 -4.855390000

H -4.094903000 1.698857000 -5.022334000

H -3.948566000 -0.031003000 -5.362304000

H -5.545762000 0.725375000 -5.332486000

C -2.238196000 4.919661000 2.270189000

C -1.228389000 6.091639000 2.250933000

H -0.353593000 5.885011000 2.874108000

H -0.876082000 6.283644000 1.232251000

H -1.700026000 7.005796000 2.628479000

C -2.717862000 4.678241000 3.721205000

H -3.204006000 5.577878000 4.114809000

H -3.438610000 3.854916000 3.759887000

H -1.887864000 4.428541000 4.388316000

C -3.456995000 5.316079000 1.417788000

H -3.173474000 5.532105000 0.382457000

H -4.217841000 4.528539000 1.409879000

H -3.918514000 6.219094000 1.829807000

===============================

**Ir(III)**

===============================

N 2.126257000 0.343480000 -1.079756000

N -0.765180000 -1.528917000 1.202380000

C 1.233481000 -2.129278000 -0.985072000

C 0.702644000 -3.419268000 -0.904530000

C 1.223084000 -4.428700000 -1.709565000

C 2.265304000 -4.215805000 -2.608626000

C 2.785899000 -2.935521000 -2.679124000

C 2.303350000 -1.874312000 -1.893648000

C 2.799412000 -0.505583000 -1.923139000

C 3.862313000 -0.004470000 -2.696691000

C 4.219069000 1.331795000 -2.610531000

C 3.509462000 2.173910000 -1.748387000

C 2.472401000 1.639047000 -1.002801000

C 1.801535000 -1.063035000 1.570858000

C 3.161102000 -0.776386000 1.718156000

C 3.833183000 -1.196099000 2.863080000

C 3.209074000 -1.900898000 3.889376000

C 1.863127000 -2.181937000 3.733361000

C 1.128439000 -1.784756000 2.602619000

C -0.288242000 -2.041864000 2.384034000

C -1.170031000 -2.732342000 3.236048000

C -2.503909000 -2.889007000 2.888353000

C -2.958319000 -2.354941000 1.679599000

C -2.054472000 -1.690534000 0.867586000

H -0.102278000 -3.665894000 -0.222389000

H 2.657385000 -5.014123000 -3.225211000

H 4.399290000 -0.673927000 -3.351226000

H 5.044576000 1.722175000 -3.195964000

H 1.908738000 2.252176000 -0.313526000

H 3.719043000 -0.241525000 0.958874000

H 3.749258000 -2.220988000 4.770816000

H -0.796895000 -3.135867000 4.164922000

H -3.189187000 -3.412262000 3.546157000

H -2.373177000 -1.271781000 -0.075480000

C -1.687757000 1.198898000 -0.905224000

C -2.934000000 1.469309000 -1.476128000

C -3.421341000 0.701551000 -2.539771000

C -2.560580000 -0.293741000 -3.034798000

C -1.338616000 -0.526790000 -2.423885000

N -0.920107000 0.175603000 -1.355274000

H -3.545462000 2.252314000 -1.051031000

H -2.850896000 -0.918714000 -3.871005000

H -0.679666000 -1.324130000 -2.749069000

C -1.157231000 1.939038000 0.266067000

C -1.671215000 3.167739000 0.689630000

C 0.401734000 1.968431000 1.989984000

C -1.139794000 3.826696000 1.803713000

H -2.478623000 3.617856000 0.129680000

H 1.228419000 1.454302000 2.468500000

C -0.076599000 3.181575000 2.459150000

H 0.391718000 3.621518000 3.331715000

N -0.117021000 1.356608000 0.909957000

Ir 0.617445000 -0.511628000 0.041473000

C 3.812331000 3.645161000 -1.661880000

F 3.794049000 -2.728680000 -3.553493000

F 0.702189000 -5.660770000 -1.621985000

F 1.258041000 -2.862940000 4.730344000

F 5.136645000 -0.911681000 2.989801000

C -4.376506000 -2.510962000 1.202009000

F 5.128712000 3.881592000 -1.802420000

F 3.169507000 4.331428000 -2.630551000

F 3.413244000 4.158380000 -0.475479000

F -4.497184000 -3.534839000 0.333938000

F -5.221774000 -2.730035000 2.222310000

F -4.787418000 -1.391405000 0.547343000

C -4.830981000 0.866205000 -3.112434000

C -5.602585000 2.015448000 -2.439316000

H -5.742474000 1.840945000 -1.367174000

H -5.102536000 2.980167000 -2.577780000

H -6.596666000 2.096454000 -2.886759000

C -5.597410000 -0.458484000 -2.865081000

H -6.628018000 -0.360558000 -3.219264000

H -5.141800000 -1.297081000 -3.399967000

H -5.620245000 -0.710074000 -1.800198000

C -4.742896000 1.148134000 -4.630449000

H -4.202878000 2.079342000 -4.828776000

H -4.240297000 0.341694000 -5.171970000

H -5.750417000 1.244614000 -5.045807000

C -1.657163000 5.178954000 2.303058000

C -0.497988000 6.203871000 2.254904000

H 0.341845000 5.904721000 2.888545000

H -0.124964000 6.330465000 1.233683000

H -0.850834000 7.176394000 2.611020000

C -2.149376000 5.016343000 3.761581000

H -2.519219000 5.976094000 4.134647000

H -2.965748000 4.289828000 3.824159000

H -1.349709000 4.687741000 4.431554000

C -2.820721000 5.710236000 1.446691000

H -2.521275000 5.874504000 0.406082000

H -3.682327000 5.034108000 1.462602000

H -3.154945000 6.672210000 1.844111000

===============================

**Ir(III)***

===============================

N 1.714185000 0.911711000 1.149993000

N -1.714377000 0.911474000 -1.149961000

C -0.589381000 2.175332000 1.385882000

C -1.835767000 2.824634000 1.445258000

C -2.088493000 3.719587000 2.471932000

C -1.141850000 4.001378000 3.464181000

C 0.084762000 3.355755000 3.402911000

C 0.402681000 2.432433000 2.397427000

C 1.662004000 1.715663000 2.268349000

C 2.766997000 1.762885000 3.133811000

C 3.890483000 0.986983000 2.877859000

C 3.905833000 0.171135000 1.739650000

C 2.802278000 0.167969000 0.902187000

C 0.588879000 2.175677000 -1.385736000

C 1.835131000 2.825233000 -1.445089000

C 2.087629000 3.720363000 -2.471671000

C 1.140881000 4.002099000 -3.463828000

C -0.085600000 3.356221000 -3.402574000

C -0.403281000 2.432709000 -2.397195000

C -1.662453000 1.715651000 -2.268155000

C -2.767533000 1.762833000 -3.133505000

C -3.890842000 0.986648000 -2.877601000

C -3.905919000 0.170562000 -1.739565000

C -2.802287000 0.167456000 -0.902206000

H -2.603528000 2.643746000 0.702659000

H -1.354846000 4.705814000 4.258804000

H 2.731661000 2.402122000 4.003371000

H 4.746725000 1.014453000 3.541954000

H 2.793806000 -0.433576000 0.004461000

H 2.602963000 2.644421000 -0.702545000

H 1.353690000 4.706676000 -4.258375000

H -2.732412000 2.402258000 -4.002934000

H -4.747158000 1.014077000 -3.541601000

H -2.793593000 -0.434269000 -0.004600000

C -0.496997000 -2.067410000 0.524883000

C -1.283613000 -3.191451000 0.846994000

C -2.281005000 -3.121226000 1.808488000

C -2.401320000 -1.885351000 2.504538000

C -1.623893000 -0.807878000 2.143780000

N -0.704449000 -0.863069000 1.149610000

H -1.136872000 -4.093061000 0.266761000

H -3.122135000 -1.762145000 3.303470000

H -1.738451000 0.156495000 2.625330000

C 0.497558000 -2.067241000 -0.525062000

C 1.284485000 -3.191043000 -0.847263000

C 1.624016000 -0.807310000 -2.143959000

C 2.281790000 -3.120495000 -1.808814000

H 1.138042000 -4.092725000 -0.267062000

H 1.738279000 0.157118000 -2.625464000

C 2.401709000 -1.884557000 -2.504829000

H 3.122421000 -1.761112000 -3.303816000

N 0.704659000 -0.862814000 -1.149722000

Ir -0.000072000 0.882304000 -0.000011000

C 5.049163000 -0.750037000 1.405049000

F 0.980709000 3.650280000 4.363652000

F -3.272661000 4.338240000 2.535764000

F -0.981656000 3.650704000 -4.363227000

F 3.271672000 4.339257000 -2.535479000

C -5.049050000 -0.750873000 -1.404995000

F 6.168095000 -0.414278000 2.066198000

F 4.746612000 -2.031304000 1.721403000

F 5.312523000 -0.731613000 0.078255000

F -5.312514000 -0.732407000 -0.078224000

F -6.168015000 -0.415457000 -2.066256000

F -4.746135000 -2.032089000 -1.721211000

C -3.280922000 -4.248559000 2.068769000

C -2.942493000 -5.522633000 1.274410000

H -2.982669000 -5.352813000 0.193487000

H -1.950474000 -5.909337000 1.531322000

H -3.671519000 -6.303591000 1.507501000

C -4.679284000 -3.745045000 1.627614000

H -5.425174000 -4.530597000 1.785163000

H -4.992725000 -2.865960000 2.198767000

H -4.684312000 -3.472255000 0.567775000

C -3.301130000 -4.598402000 3.574746000

H -2.319576000 -4.947540000 3.910712000

H -3.588486000 -3.745076000 4.195523000

H -4.027159000 -5.395962000 3.759777000

C 3.281988000 -4.247560000 -2.069177000

C 4.680280000 -3.743671000 -1.628260000

H 4.993452000 -2.864556000 -2.199512000

H 4.685391000 -3.470813000 -0.568441000

H 5.426337000 -4.529055000 -1.785856000

C 3.302048000 -4.597561000 -3.575129000

H 4.028180000 -5.395022000 -3.760172000

H 2.320500000 -4.946897000 -3.910911000

H 3.589169000 -3.744253000 -4.196035000

C 2.943982000 -5.521632000 -1.274637000

H 2.984067000 -5.351618000 -0.193740000

H 1.952101000 -5.908719000 -1.531509000

H 3.673279000 -6.302374000 -1.507599000

===============================

**^3^I**

===============================

C -2.899191000 -1.219463000 -0.000148000

C -1.502677000 -1.296253000 -0.000065000

C -0.725955000 -0.134094000 -0.000142000

N -1.301694000 1.083550000 -0.000227000

C -2.634746000 1.184185000 -0.000265000

C -3.459795000 0.068343000 -0.000248000

H -1.011918000 -2.258973000 0.000100000

H -3.031906000 2.193611000 -0.000285000

H -4.533806000 0.212504000 -0.000290000

C 0.759734000 -0.129754000 -0.000087000

C 1.545644000 -1.279695000 -0.000140000

N 1.329177000 1.095377000 0.000047000

C 2.946643000 -1.193904000 0.000079000

C 2.657601000 1.204143000 0.000226000

C 3.497215000 0.092790000 0.000292000

H 1.068962000 -2.251587000 -0.000378000

H 3.048859000 2.216089000 0.000270000

H 4.567173000 0.253189000 0.000494000

C 3.792666000 -2.473443000 0.000076000

C 3.457526000 -3.299082000 -1.264757000

C 3.457048000 -3.299394000 1.264587000

H 3.677201000 -2.731676000 2.174045000

H 2.403019000 -3.591116000 1.296428000

H 4.055511000 -4.216071000 1.280421000

H 2.403507000 -3.590788000 -1.297073000

H 4.055988000 -4.215759000 -1.280590000

H 3.678028000 -2.731145000 -2.173994000

C -3.803617000 -2.457972000 -0.000045000

C -4.694349000 -2.428467000 -1.264775000

C -4.694433000 -2.428165000 1.264610000

H -4.085657000 -2.440686000 2.174215000

H -5.329314000 -1.538273000 1.296656000

H -5.349747000 -3.305160000 1.279419000

H -5.329264000 -1.538611000 -1.297071000

H -5.349635000 -3.305484000 -1.279423000

H -4.085508000 -2.441182000 -2.174333000

C -2.998434000 -3.770169000 0.000129000

H -2.365871000 -3.860008000 0.889642000

H -3.686302000 -4.620668000 0.000227000

H -2.365856000 -3.860229000 -0.889353000

C 5.301912000 -2.170235000 0.000408000

H 5.603541000 -1.606172000 -0.888068000

H 5.603188000 -1.606376000 0.889133000

H 5.863358000 -3.109115000 0.000410000

Ni 0.006609000 2.650650000 0.000034000

Cl 0.003611000 3.372312000 -2.129634000

Cl 0.002405000 3.372204000 2.129710000

===============================

**^2^II**

===============================

C -5.257651000 2.242225000 2.561053000

C -4.455935000 3.331273000 2.918855000

C -4.795215000 1.300361000 1.648521000

H -4.822099000 4.063407000 3.631476000

H -5.429012000 0.463903000 1.371798000

C -3.181152000 3.476867000 2.356038000

C -3.509696000 1.438852000 1.077044000

H -2.559229000 4.321636000 2.635485000

C -2.706958000 2.544608000 1.443859000

H -1.721035000 2.643138000 1.002476000

H -6.246995000 2.130736000 2.993338000

C -2.979565000 0.498802000 0.118794000

O -1.898246000 0.621805000 -0.495224000

C -5.218326000 -2.973283000 -0.739900000

C -4.441396000 -2.911155000 0.413414000

C -3.693091000 -1.775437000 0.687008000

N -3.783573000 -0.712622000 -0.168880000

C -4.480901000 -0.761093000 -1.342580000

C -5.221237000 -1.900693000 -1.626197000

H -5.797158000 -3.864192000 -0.960304000

H -4.382493000 -3.751496000 1.094364000

H -5.784257000 -1.939643000 -2.550771000

C -2.760472000 -1.672668000 1.855368000

H -2.743638000 -2.617700000 2.399049000

H -1.747439000 -1.462526000 1.495004000

H -3.055163000 -0.875817000 2.544002000

C -4.392491000 0.408105000 -2.272692000

H -4.608139000 1.349846000 -1.758602000

H -3.376676000 0.468171000 -2.676208000

H -5.094477000 0.286324000 -3.097789000

Ni -0.429904000 -0.601793000 -1.037715000

Cl -1.580218000 -2.446029000 -1.720623000

C 1.470719000 3.306869000 -1.143820000

C 2.319118000 -0.700865000 -0.106452000

C 3.549808000 -1.207234000 0.317764000

C 3.694872000 -2.566465000 0.622367000

C 2.551827000 -3.368129000 0.467956000

C 1.358996000 -2.807132000 0.031963000

N 1.239836000 -1.500001000 -0.241355000

H 4.397596000 -0.541967000 0.398391000

H 2.583754000 -4.431802000 0.673388000

H 0.462027000 -3.394669000 -0.134578000

C 2.088384000 0.725452000 -0.458232000

C 3.045341000 1.731286000 -0.293096000

C 2.759036000 3.056161000 -0.640736000

C 0.563692000 2.265484000 -1.275271000

N 0.861684000 1.000037000 -0.949094000

H 4.012989000 1.477182000 0.115250000

H 1.168165000 4.306446000 -1.433470000

H -0.444932000 2.426833000 -1.640840000

C 3.770219000 4.198077000 -0.489608000

C 5.018832000 -3.180766000 1.090614000

C 4.808703000 -3.816303000 2.485898000

H 4.498696000 -3.064410000 3.218754000

H 4.050703000 -4.604530000 2.467119000

H 5.744483000 -4.264669000 2.833706000

C 6.145004000 -2.136007000 1.192156000

H 7.063500000 -2.622133000 1.532258000

H 6.360703000 -1.672602000 0.223590000

H 5.906676000 -1.346864000 1.913399000

C 5.448592000 -4.272434000 0.081191000

H 6.392907000 -4.722831000 0.402418000

H 4.708404000 -5.073775000 0.003611000

H 5.595155000 -3.849310000 -0.917555000

C 5.115876000 3.715317000 0.081453000

H 5.004223000 3.283456000 1.081735000

H 5.593102000 2.974706000 -0.568921000

H 5.800685000 4.563404000 0.167078000

C 4.022011000 4.828096000 -1.880645000

H 4.431497000 4.090953000 -2.578426000

H 3.106413000 5.236339000 -2.318101000

H 4.740998000 5.648279000 -1.791146000

C 3.183319000 5.264625000 0.465891000

H 2.247626000 5.685221000 0.086222000

H 2.988838000 4.841379000 1.456637000

H 3.893408000 6.089161000 0.582009000

===============================

**^3^III**

===============================

C -2.922246000 -1.492056000 -0.102184000

C -3.466071000 -0.204137000 -0.115335000

C -2.631096000 0.908542000 -0.180582000

N -1.302551000 0.797755000 -0.235577000

C -0.737022000 -0.432133000 -0.207500000

C -1.522452000 -1.578405000 -0.150775000

H -4.534126000 -0.037157000 -0.071681000

H -3.012156000 1.923120000 -0.187706000

H -1.042306000 -2.548312000 -0.132346000

C 2.921516000 -1.493286000 -0.102319000

C 1.521682000 -1.579024000 -0.150652000

C 0.736730000 -0.432436000 -0.207617000

N 1.302795000 0.797190000 -0.236100000

C 2.631398000 0.907413000 -0.181348000

C 3.465904000 -0.205608000 -0.115950000

H 1.041107000 -2.548712000 -0.131762000

H 3.012888000 1.921827000 -0.188782000

H 4.534040000 -0.039086000 -0.072527000

C -3.768553000 -2.768017000 -0.028864000

C -5.276198000 -2.460473000 0.016206000

C -3.386996000 -3.550907000 1.250154000

H -2.333306000 -3.845624000 1.252249000

H -3.570456000 -2.950937000 2.146757000

H -3.987119000 -4.463929000 1.320029000

H -5.544092000 -1.868898000 0.897401000

H -5.838858000 -3.397312000 0.065203000

H -5.608450000 -1.922714000 -0.877669000

C 3.767245000 -2.769612000 -0.028656000

C 3.477977000 -3.638371000 -1.275783000

C 3.385556000 -3.551797000 1.250762000

H 3.569453000 -2.951540000 2.147083000

H 2.331729000 -3.846016000 1.253160000

H 3.985267000 -4.465067000 1.320904000

H 2.425595000 -3.931706000 -1.336882000

H 4.075694000 -4.554781000 -1.237546000

H 3.733213000 -3.102766000 -2.195619000

C -3.479894000 -3.636379000 -1.276416000

H -2.427645000 -3.930122000 -1.337830000

H -4.078001000 -4.552548000 -1.238460000

H -3.735076000 -3.100269000 -2.195973000

C 5.275031000 -2.462718000 0.016009000

H 5.543350000 -1.870921000 0.896926000

H 5.837301000 -3.399777000 0.065261000

H 5.607335000 -1.925437000 -0.878134000

Ni 0.000486000 2.321975000 -0.159165000

Cl 0.001169000 2.139791000 2.197129000

Cl -1.539229000 3.867695000 -0.567927000

Cl 1.540661000 3.866937000 -0.569120000

===============================

**^3^IV**

===============================

C -4.108410000 0.001762000 4.315916000

C -2.994595000 0.002285000 5.162638000

C -3.930947000 0.001063000 2.936779000

H -3.137724000 0.002834000 6.238810000

H -4.794403000 0.000657000 2.280088000

C -1.697337000 0.002110000 4.634488000

C -2.627927000 0.000878000 2.400800000

H -0.840021000 0.002521000 5.299399000

C -1.509376000 0.001410000 3.258886000

H -0.514875000 0.001259000 2.826079000

H -5.109826000 0.001900000 4.733036000

C -2.385975000 0.000164000 0.963916000

O -1.288171000 -0.000020000 0.436857000

C -5.605932000 -0.001349000 -1.739709000

C -5.093034000 -1.206931000 -1.268873000

C -4.055824000 -1.211677000 -0.349049000

N -3.578825000 -0.000340000 0.080232000

C -4.055821000 1.210516000 -0.350393000

C -5.093039000 1.204749000 -1.270211000

H -6.403538000 -0.001758000 -2.475206000

H -5.471671000 -2.156290000 -1.626057000

H -5.471668000 2.153713000 -1.628453000

C -3.480848000 -2.462175000 0.245156000

H -3.907996000 -3.331121000 -0.254336000

H -2.400453000 -2.496794000 0.093301000

H -3.705884000 -2.523330000 1.315760000

C -3.480909000 2.461680000 0.242473000

H -3.706210000 2.524079000 1.312954000

H -2.400478000 2.496095000 0.090865000

H -3.907902000 3.330059000 -0.258134000

Ni -0.535127000 -0.000990000 -1.650102000

Cl -1.836763000 -1.724909000 -2.516522000

Cl -1.837062000 1.721685000 -2.518554000

C 1.970343000 3.473233000 -0.629065000

C 2.101357000 -0.742543000 -0.488338000

C 3.162180000 -1.509741000 0.001485000

C 3.122727000 -2.907454000 -0.056586000

C 1.971817000 -3.473440000 -0.626608000

C 0.948929000 -2.658574000 -1.092222000

N 1.004452000 -1.320058000 -1.021565000

H 4.025276000 -1.013075000 0.420270000

H 1.863790000 -4.547732000 -0.720837000

H 0.048906000 -3.054074000 -1.550667000

C 2.101029000 0.742490000 -0.488831000

C 3.161350000 1.510494000 0.000817000

C 3.121299000 2.908149000 -0.058242000

C 0.947936000 2.657596000 -1.094385000

N 1.004009000 1.319155000 -1.022738000

H 4.024497000 1.014504000 0.420300000

H 1.861900000 4.547411000 -0.724110000

H 0.047865000 3.052378000 -1.553354000

C 4.258315000 3.800170000 0.451725000

C 4.260350000 -3.798612000 0.453532000

C 3.707123000 -4.737259000 1.552373000

H 3.313920000 -4.164279000 2.398562000

H 2.906656000 -5.381041000 1.176856000

H 4.506882000 -5.385515000 1.923552000

C 5.423300000 -2.981948000 1.045611000

H 6.204333000 -3.661603000 1.397263000

H 5.878132000 -2.320112000 0.301082000

H 5.104713000 -2.378676000 1.902667000

C 4.799827000 -4.641681000 -0.727045000

H 5.613006000 -5.286919000 -0.380407000

H 4.027143000 -5.283959000 -1.158754000

H 5.189835000 -3.999741000 -1.523062000

C 5.421398000 2.984429000 1.044813000

H 5.102729000 2.381564000 1.902124000

H 5.876841000 2.322321000 0.300900000

H 6.201975000 3.664667000 1.396350000

C 4.797880000 4.642711000 -0.729183000

H 5.188493000 4.000430000 -1.524629000

H 4.025085000 5.284367000 -1.161619000

H 5.610633000 5.288538000 -0.382641000

C 3.704222000 4.739291000 1.549732000

H 2.903619000 5.382473000 1.173476000

H 3.310936000 4.166685000 2.396136000

H 4.503545000 5.388141000 1.920812000

===============================

**^2^V**

===============================

Ni 1.411456000 -0.085169000 -0.065632000

Cl 1.977806000 -0.621855000 -2.206624000

C -1.152583000 3.464616000 -0.103932000

C -1.443481000 -0.744280000 -0.032619000

C -2.628696000 -1.488736000 -0.008560000

C -2.588969000 -2.885834000 -0.003744000

C -1.316636000 -3.479867000 -0.032051000

C -0.182105000 -2.680760000 -0.059677000

N -0.244680000 -1.347357000 -0.053878000

H -3.578767000 -0.974234000 0.001868000

H -1.200672000 -4.557559000 -0.035867000

H 0.815724000 -3.105647000 -0.094040000

C -1.397240000 0.739519000 -0.049237000

C -2.543466000 1.538302000 0.007334000

C -2.446944000 2.932460000 -0.018352000

C -0.051784000 2.618885000 -0.152414000

N -0.165281000 1.285983000 -0.126257000

H -3.511606000 1.063649000 0.076195000

H -0.985663000 4.535366000 -0.129460000

H 0.961608000 2.995667000 -0.211382000

C -3.668180000 3.857811000 0.044584000

C -3.850815000 -3.757717000 0.025985000

C -3.875279000 -4.650764000 -1.236918000

H -3.892480000 -4.042487000 -2.146831000

H -3.002916000 -5.308329000 -1.288793000

H -4.769365000 -5.283082000 -1.230799000

C -5.141439000 -2.918802000 0.055211000

H -6.009764000 -3.583960000 0.078165000

H -5.192218000 -2.281045000 0.944026000

H -5.235912000 -2.286008000 -0.833596000

C -3.816276000 -4.647575000 1.290954000

H -4.709323000 -5.280312000 1.327769000

H -2.942078000 -5.304638000 1.303936000

H -3.791640000 -4.036994000 2.199142000

C -4.991255000 3.075717000 0.134314000

H -5.144010000 2.432670000 -0.738951000

H -5.037126000 2.455713000 1.035861000

H -5.829359000 3.777719000 0.175754000

C -3.547500000 4.762952000 1.293313000

H -3.514989000 4.164630000 2.209360000

H -2.645651000 5.380825000 1.263319000

H -4.410448000 5.434502000 1.353253000

C -3.701148000 4.733325000 -1.230331000

H -2.803271000 5.350543000 -1.324603000

H -3.779801000 4.113932000 -2.129504000

H -4.565770000 5.404717000 -1.200682000

C 3.079904000 1.048322000 -0.086714000

O 2.933996000 2.229682000 -0.224248000

C 4.383778000 0.349174000 0.034478000

C 4.482087000 -1.023641000 0.288920000

C 5.546372000 1.120962000 -0.126669000

C 5.735298000 -1.621482000 0.382197000

C 6.796910000 0.516850000 -0.035518000

C 6.892030000 -0.854117000 0.218570000

H 3.584422000 -1.613477000 0.429333000

H 5.448945000 2.183045000 -0.324341000

H 5.811104000 -2.685475000 0.583504000

H 7.695976000 1.112612000 -0.162191000

H 7.868342000 -1.325115000 0.290112000

Cl 1.716795000 -0.189454000 2.199407000

===============================

**^2^VI**

===============================

C -3.881120000 -0.962375000 -0.673686000

C -3.288013000 -2.229621000 -0.634364000

C -1.913284000 -2.342326000 -0.445050000

N -1.117912000 -1.287800000 -0.256296000

C -1.660251000 -0.054916000 -0.279727000

C -3.025154000 0.133813000 -0.497660000

H -3.867803000 -3.133158000 -0.770692000

H -1.418530000 -3.307386000 -0.464391000

H -3.420397000 1.140504000 -0.555350000

C -0.178873000 3.359223000 0.528897000

C -1.114519000 2.343913000 0.289159000

C -0.704078000 1.058817000 -0.066460000

N 0.604552000 0.741112000 -0.186565000

C 1.508466000 1.693486000 0.062034000

C 1.167246000 2.998896000 0.409359000

H -2.171645000 2.547062000 0.407536000

H 2.546116000 1.395605000 -0.015550000

H 1.965271000 3.707988000 0.587710000

C -5.379336000 -0.737171000 -0.914682000

C -6.147344000 -2.062336000 -1.069122000

C -5.562137000 0.089835000 -2.209232000

H -5.079109000 1.069065000 -2.140662000

H -5.137605000 -0.434198000 -3.071078000

H -6.627706000 0.256592000 -2.399458000

H -5.789777000 -2.640983000 -1.926745000

H -7.209154000 -1.854040000 -1.231622000

H -6.066290000 -2.685471000 -0.172461000

C -0.649364000 4.764973000 0.921973000

C -1.440114000 4.678510000 2.248781000

C -1.565679000 5.322295000 -0.192671000

H -1.030013000 5.385669000 -1.145139000

H -2.450487000 4.697219000 -0.345503000

H -1.910586000 6.327034000 0.073175000

H -2.321408000 4.036622000 2.158130000

H -1.782969000 5.675077000 2.546427000

H -0.813696000 4.278194000 3.052228000

C -5.974621000 0.037378000 0.284685000

H -5.499334000 1.013974000 0.417193000

H -7.044871000 0.207594000 0.127309000

H -5.851805000 -0.526057000 1.215260000

C 0.528772000 5.736432000 1.116646000

H 1.119478000 5.845758000 0.201356000

H 0.147729000 6.726411000 1.384816000

H 1.195187000 5.410678000 1.921815000

Ni 0.972744000 -1.378146000 -0.394202000

C 5.578832000 0.920727000 -0.818052000

C 4.841472000 -0.203949000 -0.446392000

C 5.171323000 1.693942000 -1.908909000

H 6.475381000 1.187043000 -0.265033000

H 5.155123000 -0.825653000 0.386613000

C 3.672117000 -0.539029000 -1.145879000

H 5.750222000 2.565005000 -2.203388000

C 4.023943000 1.340151000 -2.624882000

C 3.263833000 0.235944000 -2.239677000

C 2.869269000 -1.699183000 -0.631298000

H 3.714586000 1.930906000 -3.482536000

H 2.358240000 -0.042401000 -2.767525000

O 3.378213000 -2.763762000 -0.370898000

Cl 0.664943000 -1.984684000 -2.629586000

C 1.384176000 -1.672684000 1.560146000

C 1.083490000 -3.161478000 1.714926000

C 1.278528000 -3.619617000 3.176609000

C 0.429410000 -2.769858000 4.133631000

C 0.725426000 -1.272423000 3.963823000

C 0.533075000 -0.821539000 2.498743000

H 2.441588000 -1.490457000 1.781221000

H 1.723531000 -3.744558000 1.044754000

H 0.036573000 -3.355999000 1.436132000

H 1.026425000 -4.682824000 3.278575000

H 2.339925000 -3.517440000 3.441357000

H 0.599193000 -3.076555000 5.173337000

H -0.635389000 -2.949907000 3.923311000

H 0.085339000 -0.676962000 4.628455000

H 1.764068000 -1.074552000 4.264408000

H 0.786950000 0.240030000 2.406452000

H -0.528347000 -0.919775000 2.237380000

===============================

**^2^VI-TS**

===============================

C -3.945314000 -1.168101000 -0.395314000

C -3.282960000 -2.377671000 -0.631181000

C -1.890757000 -2.409701000 -0.624624000

N -1.134157000 -1.336493000 -0.386850000

C -1.743317000 -0.152630000 -0.170766000

C -3.135374000 -0.046787000 -0.168665000

H -3.823357000 -3.293732000 -0.831727000

H -1.352907000 -3.330617000 -0.828174000

H -3.598916000 0.918908000 -0.013191000

C -0.438519000 3.382325000 0.427050000

C -1.309705000 2.288394000 0.335541000

C -0.839758000 1.016305000 0.006148000

N 0.477238000 0.788429000 -0.200688000

C 1.317455000 1.824514000 -0.137384000

C 0.909543000 3.121694000 0.166180000

H -2.365611000 2.433150000 0.524516000

H 2.356987000 1.605846000 -0.339000000

H 1.659210000 3.901794000 0.195646000

C -5.472925000 -1.025165000 -0.396292000

C -6.180279000 -2.368619000 -0.650367000

C -5.885619000 -0.035012000 -1.511031000

H -5.453236000 0.958013000 -1.356677000

H -5.560114000 -0.393373000 -2.492472000

H -6.975098000 0.075234000 -1.529631000

H -5.917954000 -2.786905000 -1.627356000

H -7.264370000 -2.221072000 -0.635465000

H -5.936703000 -3.107811000 0.119833000

C -0.980566000 4.770662000 0.789707000

C -1.648092000 4.704218000 2.183518000

C -2.026938000 5.197494000 -0.266661000

H -1.580068000 5.240943000 -1.264773000

H -2.872610000 4.504566000 -0.307682000

H -2.421474000 6.189884000 -0.024009000

H -2.480979000 3.995003000 2.204797000

H -2.042316000 5.688368000 2.458073000

H -0.927118000 4.399500000 2.948890000

C -5.932339000 -0.481680000 0.977093000

H -5.497152000 0.498079000 1.195890000

H -7.021804000 -0.371735000 0.991661000

H -5.647396000 -1.164413000 1.784096000

C 0.132524000 5.833166000 0.831321000

H 0.625744000 5.941762000 -0.139924000

H -0.296638000 6.804736000 1.094331000

H 0.893701000 5.594127000 1.581136000

Ni 0.961607000 -1.201900000 -0.743351000

C 5.082988000 1.237407000 0.429935000

C 4.288062000 0.091988000 0.517911000

C 5.325179000 1.830723000 -0.810669000

H 5.510915000 1.665014000 1.332381000

H 4.101268000 -0.355344000 1.487645000

C 3.740536000 -0.473057000 -0.641270000

H 5.935847000 2.726861000 -0.875616000

C 4.781674000 1.265403000 -1.969024000

C 3.998197000 0.115552000 -1.890252000

C 2.814647000 -1.653673000 -0.644860000

H 4.963139000 1.724944000 -2.936336000

H 3.528490000 -0.310477000 -2.770635000

O 3.045289000 -2.700335000 -1.227651000

Cl 0.684806000 -0.855906000 -3.005680000

C 1.910951000 -2.007408000 1.061538000

C 1.280774000 -3.400749000 1.105208000

C 1.462022000 -4.023260000 2.502857000

C 0.890542000 -3.101310000 3.593127000

C 1.498580000 -1.690721000 3.523649000

C 1.316093000 -1.070306000 2.123260000

H 2.962570000 -2.144211000 1.343975000

H 1.725699000 -4.028466000 0.326867000

H 0.205645000 -3.329939000 0.899718000

H 0.980191000 -5.007937000 2.546021000

H 2.533022000 -4.187350000 2.687715000

H 1.053088000 -3.536501000 4.586566000

H -0.198517000 -3.024304000 3.458663000

H 1.045173000 -1.042018000 4.284040000

H 2.571925000 -1.747155000 3.756630000

H 1.781346000 -0.081003000 2.075645000

H 0.245979000 -0.923597000 1.938205000

===============================

**^2^VII**

===============================

C -2.931781000 -0.982497000 -0.000020000

C -1.538615000 -1.093230000 -0.000027000

C -0.731165000 0.048703000 -0.000149000

N -1.262121000 1.293122000 -0.000317000

C -2.599792000 1.415009000 -0.000395000

C -3.454627000 0.322707000 -0.000254000

H -1.070477000 -2.067806000 0.000069000

H -2.974341000 2.433607000 -0.000599000

H -4.524676000 0.497000000 -0.000314000

C 0.749855000 0.019418000 -0.000030000

C 1.515933000 -1.144811000 -0.000010000

N 1.328728000 1.245554000 -0.000115000

C 2.917371000 -1.084956000 -0.000063000

C 2.665269000 1.319557000 -0.000130000

C 3.486600000 0.195156000 -0.000105000

H 1.020566000 -2.108064000 0.000068000

H 3.075535000 2.324585000 -0.000130000

H 4.559328000 0.339106000 -0.000158000

C 3.742882000 -2.378310000 0.000022000

C 3.396127000 -3.200597000 -1.263647000

C 3.396106000 -3.200367000 1.263837000

H 3.627285000 -2.636627000 2.173175000

H 2.336877000 -3.472506000 1.296932000

H 3.978102000 -4.127907000 1.279380000

H 2.336915000 -3.472802000 -1.296686000

H 3.978171000 -4.128109000 -1.279044000

H 3.627265000 -2.637004000 -2.173086000

C -3.870526000 -2.195369000 0.000110000

C -4.761354000 -2.143403000 -1.263614000

C -4.761169000 -2.143339000 1.263955000

H -4.152722000 -2.174829000 2.173450000

H -5.369119000 -1.234789000 1.297381000

H -5.442375000 -3.000701000 1.278328000

H -5.369237000 -1.234805000 -1.297000000

H -5.442610000 -3.000726000 -1.277818000

H -4.153037000 -2.174996000 -2.173191000

C -3.101990000 -3.529366000 0.000102000

H -2.471227000 -3.635450000 0.889074000

H -3.812133000 -4.361638000 0.000099000

H -2.471236000 -3.635456000 -0.888877000

C 5.256874000 -2.099574000 0.000003000

H 5.566644000 -1.539530000 -0.888242000

H 5.566632000 -1.539335000 0.888131000

H 5.804428000 -3.046863000 0.000113000

Ni 0.057001000 2.804945000 -0.000039000

Cl 0.203364000 4.975180000 0.000326000

===============================

**^3^VIII**

===============================

C -2.910922000 -1.540171000 -0.133153000

C -3.475478000 -0.263456000 -0.196492000

C -2.648608000 0.858347000 -0.276943000

N -1.318780000 0.770423000 -0.296768000

C -0.743210000 -0.444807000 -0.231359000

C -1.511089000 -1.608057000 -0.156471000

H -4.547592000 -0.112303000 -0.179319000

H -3.027351000 1.876687000 -0.323388000

H -1.020159000 -2.572237000 -0.105130000

C 2.910618000 -1.540677000 -0.133140000

C 1.510772000 -1.608319000 -0.156385000

C 0.743099000 -0.444940000 -0.231388000

N 1.318881000 0.770178000 -0.296950000

C 2.648725000 0.857869000 -0.277212000

C 3.475400000 -0.264074000 -0.196693000

H 1.019674000 -2.572404000 -0.104894000

H 3.027648000 1.876137000 -0.323781000

H 4.547543000 -0.113111000 -0.179599000

C -3.738483000 -2.829916000 -0.033651000

C -5.252232000 -2.549573000 -0.023620000

C -3.371513000 -3.568193000 1.274781000

H -2.311640000 -3.837162000 1.305563000

H -3.580391000 -2.940164000 2.146400000

H -3.955205000 -4.491710000 1.366415000

H -5.541936000 -1.930588000 0.831326000

H -5.802534000 -3.493900000 0.046812000

H -5.575348000 -2.042919000 -0.938770000

C 3.737955000 -2.830562000 -0.033557000

C 3.418550000 -3.737564000 -1.244587000

C 3.371394000 -3.568354000 1.275263000

H 3.580647000 -2.940044000 2.146589000

H 2.311511000 -3.837227000 1.306533000

H 3.955040000 -4.491891000 1.367000000

H 2.359321000 -4.008893000 -1.280888000

H 3.999926000 -4.665253000 -1.188403000

H 3.666915000 -3.233695000 -2.184364000

C -3.419788000 -3.736609000 -1.245101000

H -2.360663000 -4.008276000 -1.281868000

H -4.001445000 -4.664127000 -1.189029000

H -3.668319000 -3.232339000 -2.184619000

C 5.251766000 -2.550527000 -0.024252000

H 5.541892000 -1.931003000 0.830161000

H 5.801896000 -3.494921000 0.046641000

H 5.574672000 -2.044582000 -0.939868000

Ni 0.000225000 2.525531000 -0.036907000

Cl 0.000414000 2.030703000 2.282217000

Cl -1.782535000 3.973779000 -0.597823000

Cl 1.783130000 3.973421000 -0.598296000

===============================

**^1^IX**

===============================

C -2.598365000 -3.288720000 -0.672312000

C -1.734345000 0.758176000 0.124615000

C -2.640341000 1.800347000 0.291469000

C -2.203289000 3.128184000 0.426984000

C -0.819721000 3.334125000 0.360996000

C 0.038066000 2.250533000 0.199636000

N -0.391791000 0.984346000 0.107501000

H -3.700858000 1.582288000 0.294942000

H -0.391040000 4.325454000 0.427711000

H 1.110821000 2.389810000 0.124391000

C -2.119176000 -0.652723000 -0.113862000

C -3.422520000 -1.144927000 -0.027146000

C -3.695784000 -2.489171000 -0.308988000

C -1.323796000 -2.741869000 -0.729434000

N -1.082946000 -1.451214000 -0.455594000

H -4.221034000 -0.477747000 0.266208000

H -2.727399000 -4.338105000 -0.910333000

H -0.454397000 -3.331695000 -1.000691000

Ni 0.702839000 -0.589991000 -0.294886000

C -5.104312000 -3.089246000 -0.235480000

C -3.219964000 4.259812000 0.611516000

C -4.044171000 3.989153000 1.893117000

H -4.776795000 4.788962000 2.038107000

H -4.593426000 3.044464000 1.837380000

H -3.399053000 3.954804000 2.776755000

C -4.162669000 4.294068000 -0.615621000

H -4.893271000 5.100308000 -0.498377000

H -3.601876000 4.474253000 -1.538036000

H -4.718750000 3.359266000 -0.734102000

C -2.538440000 5.633459000 0.746120000

H -1.869727000 5.673519000 1.612253000

H -1.965744000 5.894361000 -0.149764000

H -3.299494000 6.406387000 0.883838000

C -6.160364000 -2.050116000 0.182840000

H -5.958419000 -1.640004000 1.178147000

H -6.224368000 -1.222485000 -0.531645000

H -7.144462000 -2.525194000 0.219734000

C -5.483851000 -3.644401000 -1.629444000

H -6.487487000 -4.079259000 -1.594049000

H -5.483015000 -2.850742000 -2.383276000

H -4.795468000 -4.427016000 -1.960867000

C -5.106362000 -4.238853000 0.800546000

H -6.105891000 -4.680154000 0.862238000

H -4.408645000 -5.036421000 0.529789000

H -4.833907000 -3.872919000 1.795637000

C 4.711074000 2.388664000 -1.505058000

C 4.344931000 2.458858000 -2.849165000

C 4.191585000 1.385441000 -0.683382000

H 5.410677000 3.109736000 -1.092529000

H 4.752013000 3.237226000 -3.486866000

C 3.457444000 1.511483000 -3.371140000

H 4.512791000 1.335381000 0.352780000

C 3.289068000 0.440010000 -1.196933000

H 3.177979000 1.549030000 -4.419948000

C 2.932325000 0.513419000 -2.555734000

C 2.628669000 -0.601414000 -0.351894000

H 2.242782000 -0.226060000 -2.948878000

O 2.054937000 -1.671144000 -0.877318000

N 3.493352000 -0.979779000 0.866194000

C 5.196574000 -2.394223000 1.750217000

C 4.981965000 -1.873699000 3.020724000

C 4.029590000 -0.874495000 3.185264000

H 5.559116000 -2.230234000 3.867796000

C 4.454545000 -1.938125000 0.663634000

H 5.950513000 -3.153524000 1.579240000

C 4.710459000 -2.476063000 -0.712997000

C 3.286394000 -0.425111000 2.097464000

C 2.273002000 0.663217000 2.264947000

H 3.851241000 -0.427967000 4.156286000

H 5.625907000 -3.068918000 -0.708369000

H 3.872001000 -3.093277000 -1.042681000

H 4.813139000 -1.666692000 -1.439812000

H 2.438253000 1.465739000 1.544983000

H 1.261024000 0.281360000 2.099017000

H 2.337627000 1.076749000 3.272653000

===============================

**^1^IX-TS**

===============================

C -2.812337000 -3.197646000 -0.785925000

C -1.712484000 0.795185000 0.000399000

C -2.549882000 1.876382000 0.252273000

C -2.033260000 3.173809000 0.407104000

C -0.645439000 3.309349000 0.277007000

C 0.144119000 2.190354000 0.030756000

N -0.364504000 0.956148000 -0.086764000

H -3.618534000 1.714391000 0.313902000

H -0.160890000 4.273292000 0.359557000

H 1.219453000 2.270508000 -0.089154000

C -2.177453000 -0.591663000 -0.240085000

C -3.503042000 -1.011843000 -0.126405000

C -3.856127000 -2.339387000 -0.400688000

C -1.511788000 -2.720450000 -0.873430000

N -1.193284000 -1.445042000 -0.606101000

H -4.258452000 -0.301882000 0.179993000

H -3.002858000 -4.238700000 -1.018649000

H -0.685559000 -3.360074000 -1.165685000

Ni 0.618827000 -0.641444000 -0.540950000

C -5.293494000 -2.860669000 -0.297386000

C -2.975566000 4.349635000 0.685476000

C -3.740580000 4.077105000 2.003045000

H -4.419191000 4.908827000 2.215206000

H -4.343093000 3.165363000 1.948682000

H -3.050231000 3.978098000 2.846682000

C -3.981475000 4.474947000 -0.484289000

H -4.659903000 5.313373000 -0.299548000

H -3.464044000 4.658086000 -1.431112000

H -4.593024000 3.575135000 -0.600342000

C -2.214653000 5.680467000 0.824553000

H -1.499748000 5.656869000 1.653581000

H -1.677587000 5.941395000 -0.093128000

H -2.924708000 6.486683000 1.027737000

C -6.281357000 -1.765248000 0.143190000

H -6.035877000 -1.367668000 1.133819000

H -6.315937000 -0.935014000 -0.570322000

H -7.288826000 -2.185837000 0.201812000

C -5.731640000 -3.392566000 -1.683373000

H -6.756688000 -3.771358000 -1.626484000

H -5.703154000 -2.599448000 -2.437208000

H -5.094791000 -4.211824000 -2.028953000

C -5.336163000 -4.009773000 0.738552000

H -6.356933000 -4.395319000 0.820994000

H -4.689567000 -4.844416000 0.453056000

H -5.023405000 -3.660757000 1.727828000

C 4.413889000 1.309883000 -3.273757000

C 4.970814000 2.353065000 -2.529776000

C 3.627403000 0.343292000 -2.648782000

H 4.597181000 1.244457000 -4.341920000

H 5.586535000 3.101735000 -3.018375000

C 4.739746000 2.426880000 -1.152957000

H 3.189308000 -0.471912000 -3.215074000

C 3.393836000 0.416665000 -1.265851000

H 5.180775000 3.229560000 -0.569764000

C 3.951019000 1.466752000 -0.521951000

C 2.449350000 -0.555686000 -0.677538000

H 3.797510000 1.507660000 0.550899000

O 2.082167000 -1.658998000 -1.176937000

N 3.403235000 -1.076967000 1.052768000

C 5.030595000 -2.522816000 2.035776000

C 4.568301000 -2.202522000 3.306765000

C 3.516229000 -1.301461000 3.429562000

H 5.024384000 -2.643028000 4.187910000

C 4.433216000 -1.946576000 0.909828000

H 5.855716000 -3.213084000 1.900442000

C 4.945278000 -2.279911000 -0.465793000

C 2.943989000 -0.749000000 2.280948000

C 1.798574000 0.216958000 2.401609000

H 3.132413000 -1.018928000 4.403649000

H 5.776913000 -2.982841000 -0.397943000

H 4.157523000 -2.720695000 -1.080315000

H 5.294254000 -1.380065000 -0.979033000

H 1.943516000 1.089103000 1.763573000

H 0.860159000 -0.252869000 2.089075000

H 1.687012000 0.553552000 3.434241000

===============================

**^3^X**

===============================

C 0.148186000 3.325004000 -0.063727000

C -1.169007000 3.119690000 -0.494686000

C -1.577887000 1.850098000 -0.894183000

N -0.759868000 0.794358000 -0.870975000

C 0.519487000 0.956325000 -0.465965000

C 0.992903000 2.204750000 -0.065676000

H -1.885302000 3.929812000 -0.535117000

H -2.577705000 1.648633000 -1.264930000

H 2.027478000 2.310638000 0.236642000

C 3.316358000 -1.594323000 0.032141000

C 2.615003000 -0.379120000 0.055893000

C 1.336422000 -0.281832000 -0.488860000

N 0.730324000 -1.353983000 -1.051654000

C 1.371088000 -2.527137000 -1.057583000

C 2.655206000 -2.686055000 -0.542922000

H 3.063543000 0.491362000 0.518512000

H 0.814808000 -3.363399000 -1.464831000

H 3.112726000 -3.665552000 -0.590982000

C 0.685947000 4.689816000 0.386241000

C -0.392552000 5.786724000 0.326814000

C 1.856117000 5.101929000 -0.537934000

H 2.679920000 4.382868000 -0.501157000

H 1.524422000 5.180892000 -1.578010000

H 2.251087000 6.075798000 -0.230115000

H -0.768197000 5.932733000 -0.691026000

H 0.033400000 6.737747000 0.660315000

H -1.240914000 5.557746000 0.979830000

C 4.724211000 -1.680362000 0.635495000

C 4.652289000 -1.318566000 2.137978000

C 5.650069000 -0.679468000 -0.095309000

H 5.712906000 -0.911579000 -1.163208000

H 5.298300000 0.351743000 0.004757000

H 6.660161000 -0.727957000 0.324777000

H 4.275945000 -0.303615000 2.297092000

H 5.650087000 -1.377171000 2.585425000

H 3.995046000 -2.009194000 2.675557000

C 1.190856000 4.577826000 1.844307000

H 1.997559000 3.845312000 1.943216000

H 1.577374000 5.545125000 2.182030000

H 0.380959000 4.279502000 2.517509000

C 5.327340000 -3.090335000 0.501273000

H 5.418160000 -3.394919000 -0.546410000

H 6.330937000 -3.102842000 0.936913000

H 4.729407000 -3.839928000 1.029427000

Ni -1.278093000 -1.089351000 -1.480558000

C -3.792998000 -1.802631000 3.315158000

C -2.878617000 -2.275649000 2.377570000

C -4.707021000 -0.804988000 2.957008000

H -3.802106000 -2.210800000 4.322408000

H -2.168019000 -3.059415000 2.622787000

C -2.860017000 -1.740448000 1.079759000

H -5.423897000 -0.439916000 3.687719000

C -4.703457000 -0.282080000 1.661145000

C -3.774920000 -0.739857000 0.725924000

C -1.832834000 -2.213744000 0.092916000

H -5.425432000 0.479756000 1.380189000

H -3.769862000 -0.362807000 -0.293041000

O -1.254476000 -3.280031000 0.251827000

Cl -2.973918000 -0.730267000 -2.922562000
